# Multifunctional activities of ERF109 as affected by salt stress in Arabidopsis

**DOI:** 10.1038/s41598-018-24452-6

**Published:** 2018-04-23

**Authors:** Ahmed Bahieldin, Ahmed Atef, Sherif Edris, Nour O. Gadalla, Ahmed M. Ramadan, Sabah M. Hassan, Sanaa G. Al Attas, Magdy A. Al-Kordy, Abdulrahman S. M. Al-Hajar, Jamal S. M. Sabir, Mahmoud E. Nasr, Gamal H. Osman, Fotouh M. El-Domyati

**Affiliations:** 1 Department of Biological Sciences, Faculty of Science, King Abdulaziz University (KAU), P.O. Box 80141, Jeddah, 21589 Saudi Arabia; 2 Department of Genetics, Faculty of Agriculture, Ain Shams University, Cairo, Egypt; 3 Princess Al-Jawhara Al-Brahim Centre of Excellence in Research of Hereditary Disorders (PACER-HD), Faculty of Medicine, King Abdulaziz University (KAU), Jeddah, Saudi Arabia; 4 Department of Arid Land Agriculture, Faculty of Meteorology, Environment and Arid Land Agriculture, King Abdulaziz University, Jeddah, Saudi Arabia; 5 Genetics and Cytology Department, Genetic Engineering and Biotechnology Division, National Research Center, Dokki, Egypt; 6 Agricultural Genetic Engineering Research Institute (AGERI), Agriculture Research Center (ARC), Giza, Egypt; 7 Faculty of Agriculture, Menofia University, Shebeen Elkom, Egypt; 8 Department of Biology, Umm Al-Qura University, Makkah, Saudi Arabia

## Abstract

Transcriptomic analysis was conducted in leaves of *Arabidopsis* T-DNA insertion *ERF109*-knocked out (KO) mutant or plants overexpressing (OE) the gene to detect its role in driving expression of programmed cell death- (PCD-) or growth-related genes under high salt (200 mM NaCl) stress. The analysis yielded ~22–24 million reads, of which 90% mapped to the *Arabidopsis* reference nuclear genome. Hierarchical cluster analysis of gene expression and principal component analysis (PCA) successfully separated transcriptomes of the two stress time points. Analysis indicated the occurrence of 65 clusters of gene expression with transcripts of four clusters differed at the genotype (e.g., WT (wild type), KO^*ERF109*^ or OE^*ERF109*^) level. Regulated transcripts involved *DIAP1*-like gene encoding a death-associated inhibitor of reactive oxygen species (ROS). Other ERF109-regulated transcripts belong to gene families encoding ROS scavenging enzymes and a large number of genes participating in three consecutive pathways, e.g., phenylalanine, tyrosine and tryptophan biosynthesis, tryptophan metabolism and plant hormone signal transduction. We investigated the possibility that ERF109 acts as a “master switch” mediator of a cascade of consecutive events across these three pathways initially by driving expression of *ASA1* and *YUC2* genes and possibly driving *GST*, *IGPS* and *LAX2* genes. Action of downstream auxin-regulator, auxin-responsive as well as auxin carrier genes promotes plant cell growth under adverse conditions.

## Introduction

Programmed cell death (or PCD) represents a cascade of events that lead to the programmed destruction of cells^1^. This process is accurate and genetically controlled due to the regulation of a large number of genes and related processes. Plant experiences a variety of responses to orchestrate PCD events such as the accumulation of reactive oxygen species (ROS), release of mitochondrial cytochrome c and the activation of caspase-like proteases^2^. Under unfavorable conditions, H_2_O_2_ accumulates, hence, stimulates the production of ROS or the occurrence of oxidative burst^2^. ROS comprise free radicals, e.g., superoxide (O_2_^−^), hydroxyl radical (OH^•^), and non-radicals, e.g., hydrogen peroxide (H_2_O_2_) and singlet oxygen (^1^O_2_)^3^. Detoxification of oxygen species, generated under adverse condition, is mandatory for the protection of plant cells and organelles against the toxic effects^4^.

Cells exposed to harsh conditions, e.g., pathogen attack or abiotic stresses, induce several processes including PCD. In our recent work, we confirmed that manipulating PCD-related genes resulted in differential levels of salt stress tolerance in plant^5,6^. Of which, knockout mutants of *Bax Inhibitor 1 (BI-1)* gene and its driving transcription factor namely *ethylene responsive factor 109* (*ERF109*) gene in *Arabidopsis* confirmed the role of either gene in standing salt stress. Bax inhibitor-1 (BI-1) is a cell death suppressor conserved among eukaryotes^7,8^. BI-1 blocks Bax-induced cell death downstream of Bax action in the mitochondria. The protein (25–27 kDa) is trans-membrane residing in endoplasmic reticulum that exists in several tissues including leaf, root and stem and largely enhanced under stress conditions such as heat, cold, drought and salt. BI-1 serves in increasing the capacity of cellular homeostasis under oxidative stress condition, thus, blocks cell death^2^. Bahieldin *et al*.^5,6^ indicated that expression of such a protein is due to the action of ERF109. In tobacco (*Nicotiana benthamiana*), *BI-1* gene was upregulated early before the onset of PCD (2 h of treatment with a PCD-inducing agent namely oxalic acid; Bahieldin *et al*.^5^. Action of *BI-1* gene was also previously confirmed in *Arabidopsis*^9^. These results supports the idea that *BI-1* gene overexpression is vital for basal suppression of cell death progression under adverse conditions.

*Arabidopsis* ERF109 was recently proposed to mediate cross-talking of jasmonic acid and auxins such as indole acetic acid^10^. The TF drives expression of two genes, e.g., *Anthranilate synthase alpha subunit 1* (or *ASA1*) and *YUC2* gene of the *YUCCA* gene family encoding flavin monooxygenase-like enzyme, important in two pathways namely tryptophan biosynthesis and tryptophan metabolism, respectively. Plants overexpressing *ERF109* gene resulted in the overproduction of auxins, thus, overgrowth of roots, hairy roots and hypocotyls. Upon JA treatment, the gene is also overexpressed in shoots and roots, especially in the lateral root primordia^11^.

In the present study, transcriptomic analysis via RNA-Seq was conducted in leaves of *Arabidopsis* T-DNA insertion mutant knocked out for *ERF109* gene (loss-of-function) and transgenic plants overexpressing *ERF109* gene (gain-of-function) in a trial to detect the possible role of this TF in driving expression of other PCD- or plant growth-related genes under salt stress.

## Results and Discussion

RNA-Seq of cDNA samples of *Arabidopsis* leaves of three genotypes, e.g., WT, KO^*ERF109*^ and OE^*ERF109*^, treated with NaCl (200 mM) for 2 and 12 h as well as the untreated control was done in order to search transcripts that might be driven by ERF109 other than those recently detected^5,6,9^. Two time points were selected based on previous evidence that this TF is upregulated after 2 h of exposure to PCD inducer (e.g., oxalic acid) or salt stress^5,6^. The analysis yielded ~22–24 million reads corresponding to an average of >2 billion nucleotides of cDNA per sample (Table 1). The raw RNA-Seq data indicated that over 90% of the reads mapped to the *Arabidopsis* reference nuclear genome in the exonic regions (Table 1).

**Table 1:** Statistics of *Arabidopsis* RNA-Seq numerical data analysis for three genotypes (WT, KO^*ERF109*^ and OE^*ERF109*^) across time of salt stress treatment (2 and 12 h as well as the untreated controls).

Genotype-time point	Total no. reads^1^	% Mapped reads^2^	% Unique matches^3^	% Multi-position matches^4^	% unmapped reads^5^
WT-C	22,444,772	92.25	81.3	10.95	7.75
WT-2h	24,080,924	91.49	83.03	8.47	8.51
WT-12h	23,921,946	91.34	83.92	7.41	8.66
KO-C	24,108,677	92.98	83.36	9.62	7.02
KO-2h	24,114,677	90.42	81.73	8.69	9.58
KO-12h	24,122,423	92.01	84.1	7.91	7.99
OE-C	23,948,590	93.32	83.81	9.5	6.68
OE-2h	24,119,974	92.87	84.35	8.52	7.13
OE-12h	22,229,894	91.6	84.54	7.06	8.4

WT = wild type, C = control untreated, KO^*ERF109*^ = *ERF109* knocked out mutant, OE^*ERF109*^ = *ORF109* overexpressed plant.

^1^Total number of reads recovered from RNA-Seq.

^2^Percentage of reads aligned with Arabidopsis genome over total reads.

^3^Percentage of reads with unique matches.

^4^Percentage of reads with multi-position matches.

^5^Percentage of unmapped reads.

### Cluster analysis

**Figure 1:**
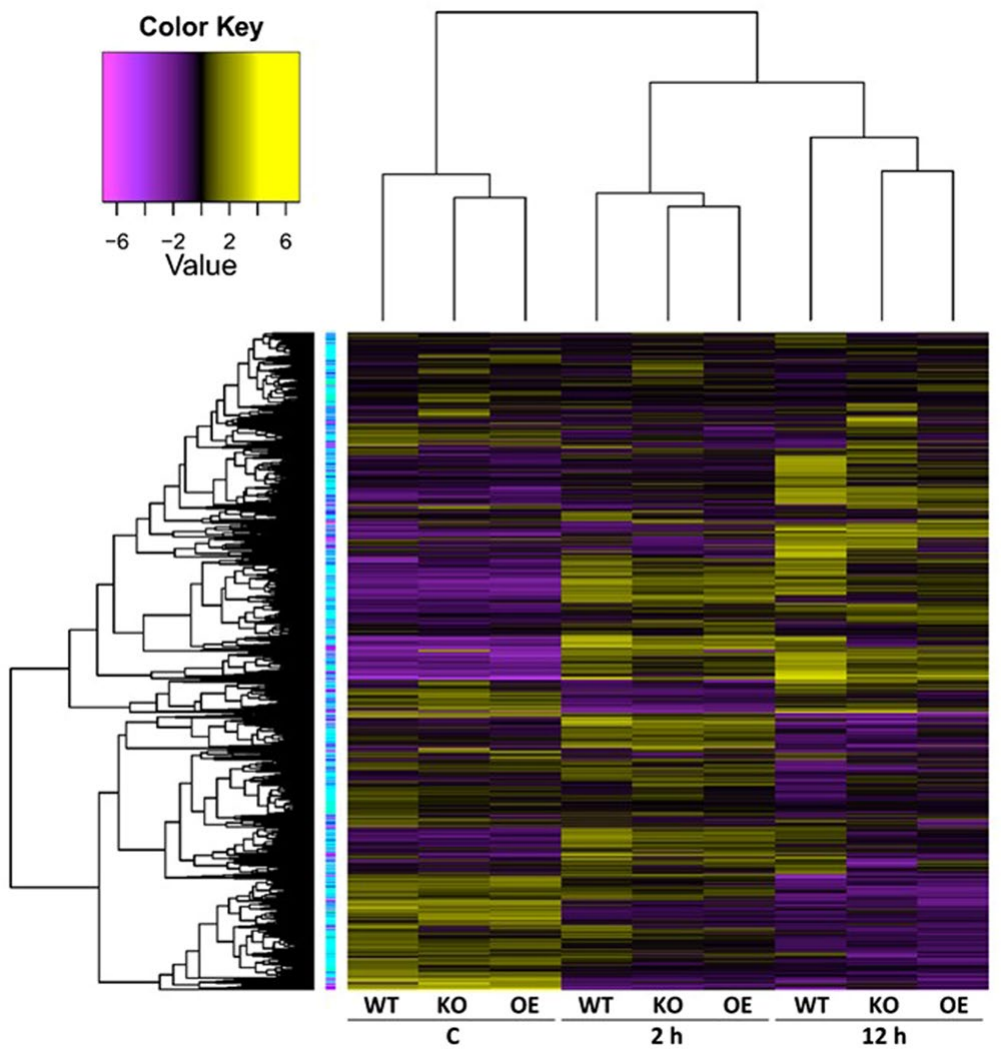
Hierarchical cluster analysis of gene expression based on log ratio RPKM data for leaf transcriptome of *Arabidopsis* genotypes (e.g., WT, KO^*ERF109*^ and OE^*ERF109*^) under high salt (200 mM NaCl) treatment for 2 and 12 h as well as the untreated control. WT = wild type, C = control untreated, KO = *ERF109* knocked out mutant, OE = *ORF109* overexpressed.

**Figure 2:**
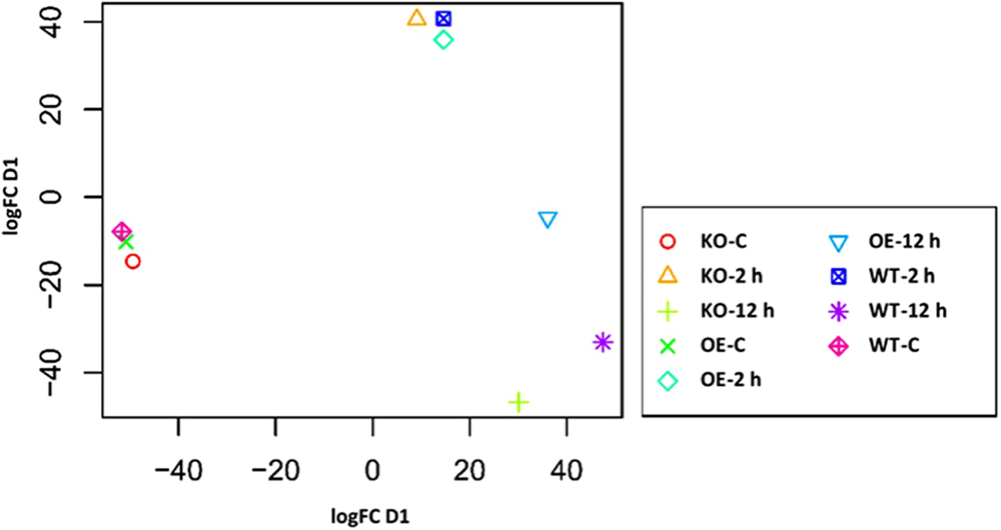
Multi-dimensional scaling plot of raw sequence counts of RNA-seq data for leaf transcriptome of *Arabidopsis* genotypes (e.g., WT, KO^*ERF109*^ and OE^*ERF109*^) under high salt (200 mM NaCl) treatment for 2 and 12 h as well as the untreated control. WT = wild type, C = control untreated, KO = *ERF109* knocked out mutant, OE = *ORF109* overexpressed. Each sample is displayed as a different color.

**Figure 3:**
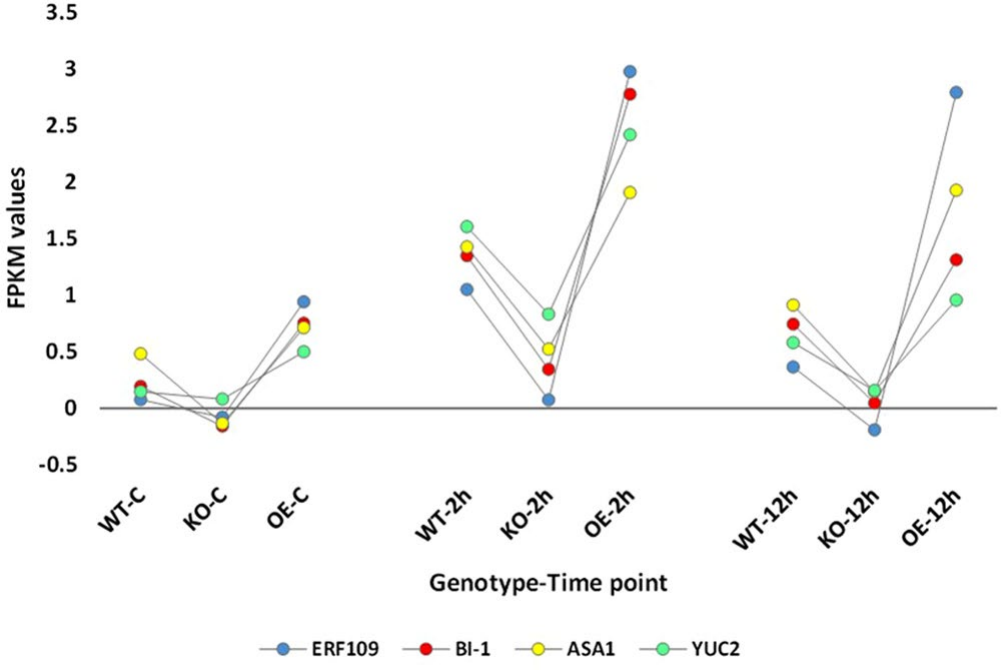
FPKM values for the transcripts upregulated at 2 h of salt (200 mM) stress treatment that were concordantly expressed with *ERF109* transcript in leaves of *Arabidopsis* genotypes (e.g., WT, KO^*ERF109*^ and OE^*ERF109*^) across time (2 and 12 h) of salt stress treatment as well as the untreated controls. Transcripts showed relatively lower levels of expression in leaves of KO^*ERF109*^ plants, while higher levels of expression in leaves of OE^*ERF109*^ plants compared with WT at any given time point. WT = wild type, C = control untreated, KO^*ERF109*^ = *ERF109* knocked out mutant, OE^*ERF109*^ = *ORF109* overexpressed plant. Description of transcripts is shown in Table S1 (cluster 1).

**Figure 4:**
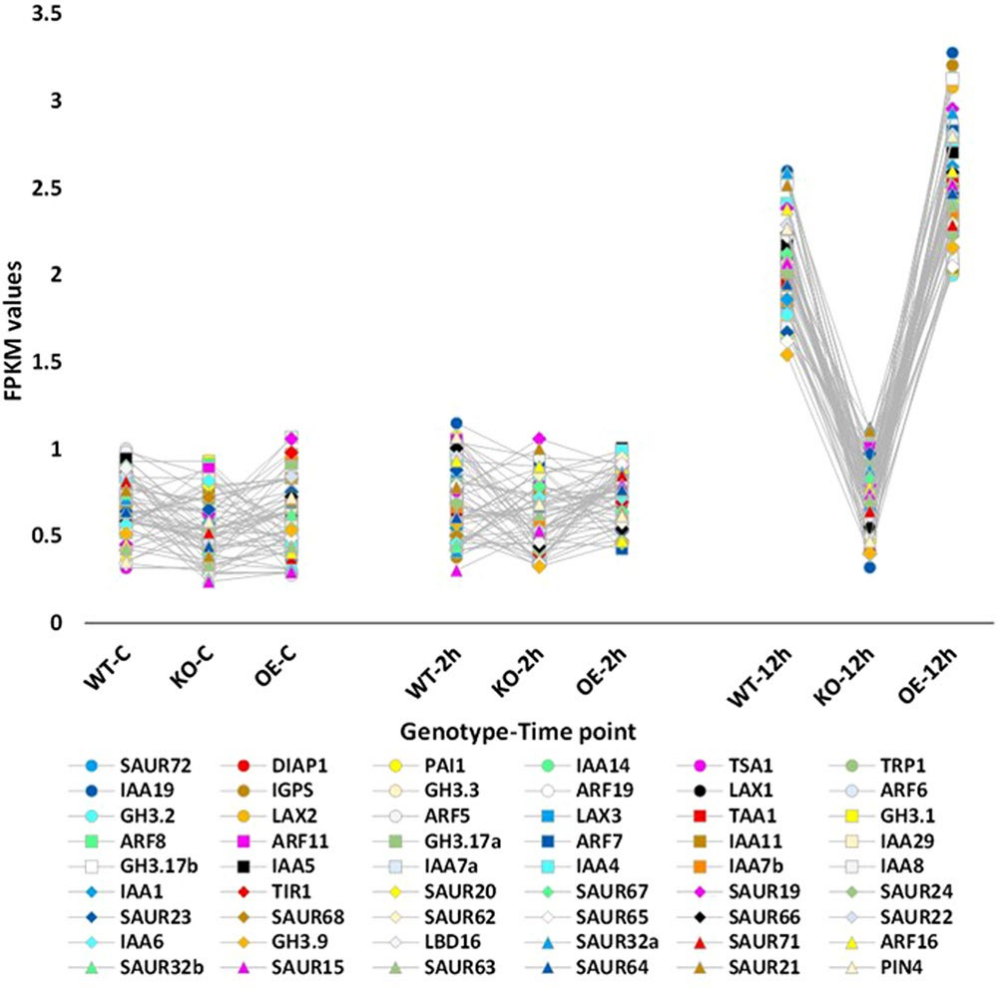
FPKM values for the transcripts upregulated at 12 h of salt (200 mM) stress treatment with relatively lower levels of expression in leaves of *Arabidopsis* KO^*ERF109*^ plants, while higher levels of expression in leaves of OE^*ERF109*^ plants compared with WT. Gene expression levels in the three genotypes (e.g., WT, KO^*ERF109*^ and OE^*ERF109*^) were not changed at earlier time points (control and 2 h) of salt stress treatment. WT = wild type, C = control untreated, KO^*ERF109*^ = *ERF109* knocked out mutant, OE^*ERF109*^ = *ORF109* overexpressed plant. Description of transcripts is shown in Table S1 (cluster 2).

**Figure 5:**
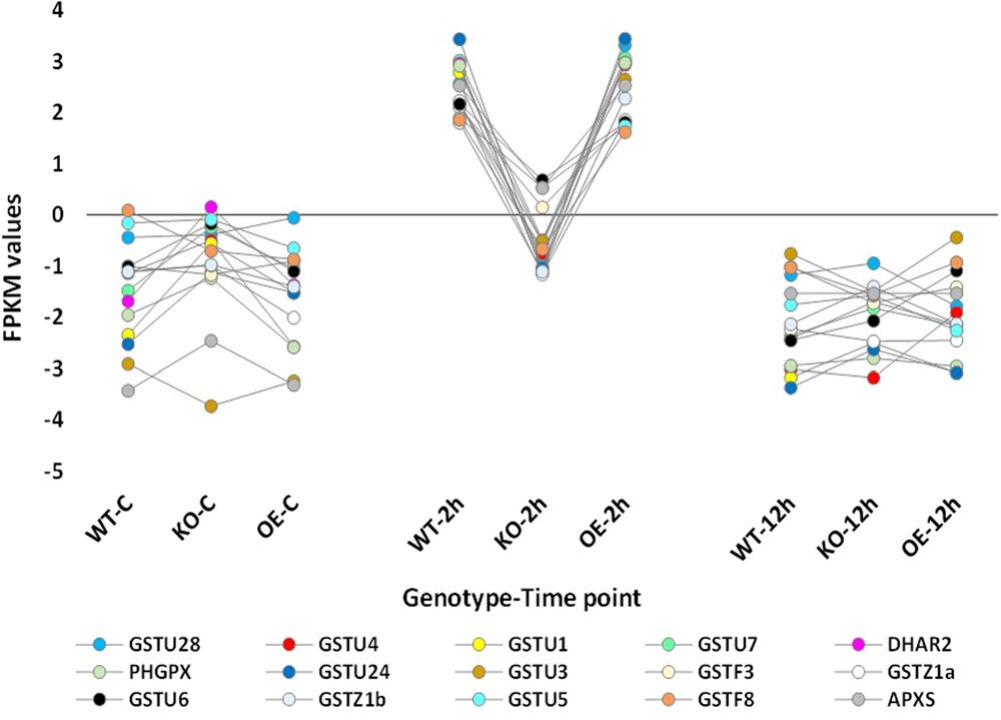
FPKM values for the transcripts upregulated at 2 h of salt (200 mM) stress treatment with relatively lower levels of expression in leaves of *Arabidopsis* KO^*ERF109*^ plants compared with OE^*ERF109*^ and WT. Gene expression levels in the three genotypes (e.g., WT, KO^*ERF109*^ and OE^*ERF109*^) were not changed at either earlier or later time point (control or 12 h, respectively) of salt stress treatment. WT = wild type, C = control untreated, KO^*ERF109*^ = *ERF109* knocked out mutant, OE^*ERF109*^ = *ORF109* overexpressed plant. Description of transcripts is shown in Table S1 (cluster 3).

**Figure 6:**
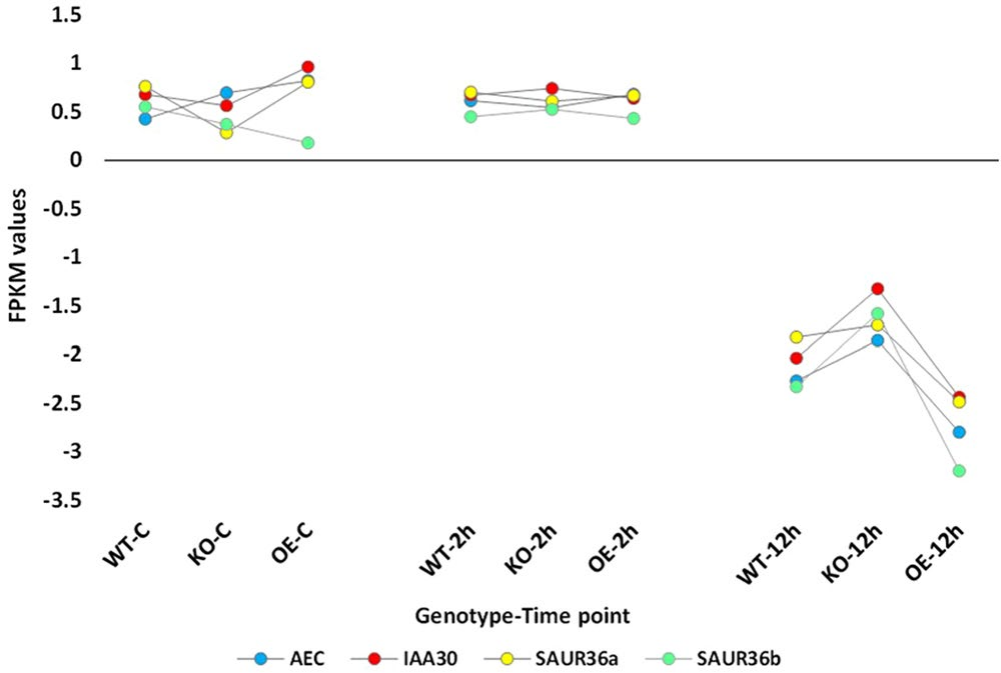
FPKM values for the transcripts downregulated at 12 h of salt (200 mM) stress treatment with relatively higher levels of expression in leaves of *Arabidopsis* KO^*ERF109*^, while lower levels of expression in leaves of OE^*ERF109*^ plants compared with WT. Gene expression levels in the three genotypes (e.g., WT, KO and OE) were not changed at earlier time points (control and 2 h) of salt stress treatment. WT = wild type, C = control untreated, KO^*ERF109*^ = *ERF109* knocked out mutant, OE^*ERF109*^ = *ORF109* overexpressed plant. Description of transcripts is shown in Table S1 (cluster 4).

Hierarchical cluster analysis of gene expression based on log ratio RPKM data and Multi-dimensional scaling plot for transcripts of the three genotypes of *Arabidopsis* indicated transcriptomic separation of the two time points of salt stress (Figs 1 and 2, respectively). Transcriptomes at 2 and 12 h time points were closer than either time point and control. Based on the transcriptomic data shown in Figs 1 and 2, we concluded that the distance between transcriptomes of 2 h salt-treated or 12 h salt-treated samples and transcriptomes of the control samples is almost the same. Therefore, we assume untreated samples at 12 h time point can be a general control of the experiment. The total number of generated clusters was 65 (Table S1) with ~1470 differentially expressed transcripts (Table S1). Of which, expression levels of transcripts of clusters 1, 2, 3 and 4 differed at the genotype (e.g., WT, KO^*ERF109*^ or OE^*ERF109*^) level. No regulation was shown across the four clusters for transcripts of the three genotypes of the control. Transcripts of OE^*ERF109*^ showed the high expression levels at 2 and 12 h time points in cluster 1 and at 12 h only in cluster 2, while transcripts of KO^*ERF109*^ showed low expression levels at the two time points in the two clusters (Figs 3 and 4). Transcripts of WT and OE^*ERF109*^ in cluster 3 showed similar expression levels at 2 h time point, while transcripts of KO^*ERF109*^ showed the lowest at this time point (Fig. 5). However, results of cluster 4 remain unexplained as transcripts of KO^*ERF109*^ showed high expression levels at 12 h time point, while OE^*ERF109*^ showed low expression levels (Fig. 6). Transcripts of these four clusters at selected time points are further called *ERF109*-regulated or *ERF109*-upregulated. Transcripts of other clusters were differentially expressed across time of salt stress treatment regardless of the genotype, e.g., *ERF109*-independent (Table S1). These clusters showed either salt-induced up- or downregulation at a given time point, e.g., 2 h (9 clusters), 12 h (28 clusters), 2 h/12 h (20 clusters) or with gradual up- or downregulation (4 clusters). RNA-Seq data was successfully validated via the use of semi-quantitative (sq)RT-PCR for five transcripts of cluster 2 (Table S2) with WT and OE^*ERF109*^ genotypes unevenly upregulated at 12 h time point (Fig. S1). The four *ERF109*-regulated clusters were further analyzed as the focus of this study. These clusters contained 3, 54, 15 and 4 *ERF109*-regulated transcripts, respectively (Table S1).

### Regulated cell death genes

One transcript in cluster 2 namely *DAL1-like* or *DAL2-like*, analogue of *DIAP1* gene in *Drosophila*, encodes a death-associated inhibitor of ROS during either PCD or biotic/abiotic stress^12,13^. This gene naturally acts as a negative regulator of PCD in *Arabidopsis*^12^. Previous reports with knockout mutants of *DAL1* or *DAL2* gene resulted in higher accumulation of ROS upon infection with the avirulent strain of *Pseudomonas syrinage* pv. *tomato* (Pst) DC3000, thus, approached PCD earlier than the WT. The idea that this gene is regulated by ERF109 aligns with our speculation that ERF109 might regulate PCD-inhibitor genes other than the recently discovered *BI-1* gene.

### Regulated genes encoding ROS scavenging enzymes

The results in Fig. 5 indicate that some ERF109-regulated transcripts of cluster 3 belong to gene families encoding four ROS scavenging enzymes, namely ascorbate peroxidase (APXS), glutathione S-transferase (GST), phospholipid hydroperoxide glutathione peroxidase (PHGPX) and dehydroascorbate reductase (DHAR). As Bax is a PCD-inducing protein stimulated by ROS, its action can be blocked not only by the action of BI-1 or DAL1/2 protein but also by the action of ROS scavenging enzymes such as APXS, GST, PHGPX and DHAR^2,14–16^. The first enzyme namely APXS is a key enzyme in the peroxide-detoxification system in chloroplasts as it converts H_2_O_2_ into H_2_O with ascorbate used as an electron donor^17^. Previous reports indicated that this enzyme is regulated under both biotic and abiotic stresses^17^ and acts in scavenging ROS in several organelles and the cytosol^18^. Under salt stress, responses of APXS gene were previously reported to be tissue- and developmental stage-specific^19^. The second ROS scavenger namely GST has a major role in glutathione-dependent isomerization and the reduction of toxic hydroperoxides as GST attaches glutathione to electrophilic xenobiotics for subsequent sequestration in the plant vacuole^20^. The enzyme also has affinity for auxins, thus, contributes to hormone homeostasis under different environmental conditions^21^. The third enzyme namely PHGPX provides an enzymatic defense mechanism against oxidative destruction of biomembranes and acts in the removal of lipid hydroperoxides^22^. The latter compounds are toxic and naturally recovered during accumulation of ROS, e.g., hydroxyl radicals and singlet oxygen. Lipid hydroperoxidation results in the decreased membrane fluidity and damage of transmembrane proteins, hence, inactivates protein receptors, important enzymes and ion channels within the membrane. Recent reports indicated that lipid hydroperoxides can be scavenged by PHGPX^22^. The forth enzyme namely DHAR reduces dehydroascorbate to non-enzymatic antioxidants ascorbic acid. This action requires the use of a reduced form of glutathione as an electron donor^23^. The enzyme participates in regulating AA pool size in symplast and apoplast towards maintaining the plant cell’s redox state under stress conditions^24^. In general, the present data supports the involvement of ERF109 in retarding PCD under salt stress due to the regulation of processes participating in ROS inhibition.

Aligning with the latter conclusion, *ERF109* gene was previously proven to express in *Arabidopsis* when plant leaves approach re-adjustment towards homeostasis under other types of abiotic stresses, ex., high light stress^25^.

### KEGG analysis

**Figure 7:**
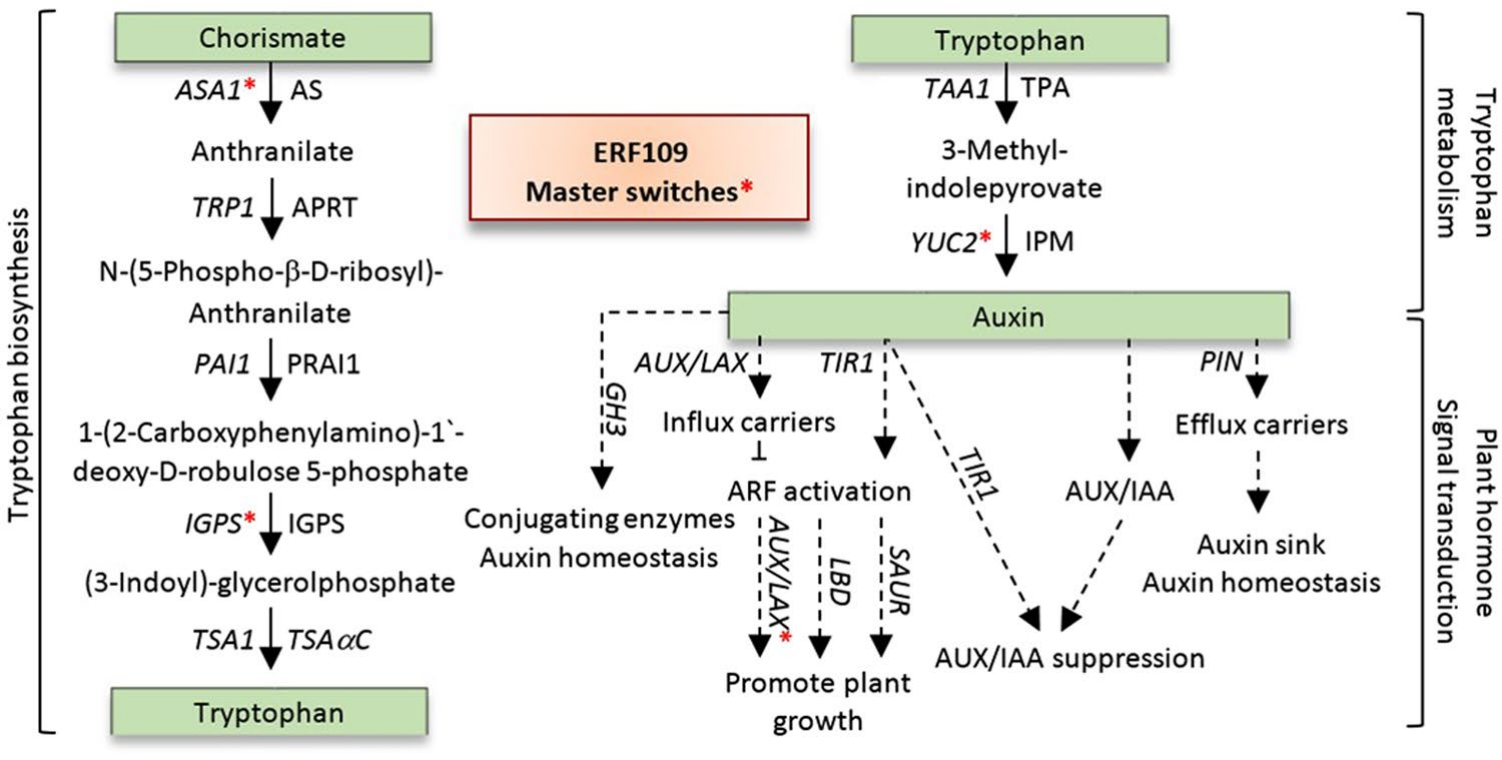
Activated steps in the pathways of tryptophan biosynthesis, tryptophan metabolism and plant hormone signal transduction due to the initial ERF109-mediated “master switch” (indicated by red asterisks) at the three pathways for *ASA1*, *IGPS*, *YUC2* and *LAX2* genes. Master switch subsequently triggered expression of other downstream genes and gene families of *TRP1*, *PAI1*, *TSA1*, *TAA1*, *YUC2*, *AUX*/*IAA* (or *IAA*), *ARF*, *TIR1*, *GH3*, *PIN4*, *AUX*/*LAX* (or *LAX*), *LBD* and *SAUR*. AS = anthranilate synthase alpha subunit 1, APRT = anthranilate phosphoribosyltransferase, PRAI = phosphoribosylanthranilate isomerase 1, IGPS = indole-3-glycerol phosphate synthase, TSAαC = tryptophan synthase alpha chain, TPA = L-tryptophan pyruvate aminotransferase, IPM = indole-3-pyruvate monooxygenase.

Other ERF109-regulated transcripts of the four clusters mainly belong to three consecutive pathways, e.g., tryptophan biosynthesis^26–28^ (Fig. S2), tryptophan metabolism^26–28^ (Fig. S3) and the downstream plant hormone signal transduction^26–28^ (Fig. S4). The first pathway results in the biosynthesis of tryptophan that is used in the second pathway as a substrate for auxin (IAA) biosynthesis. In turn, biosynthesized auxin participates in the third pathway in inducing responses of genes related to cell enlargement and plant growth. KEGG analysis was conducted in order to detect the activated enzymes after 12 h of exposure to the target salt concentration (200 mM NaCl) as compared to those in the control plantlets whose samples were concurrently harvested. We can extract that ERF109 might act as a “master switch” mediator of a cascade of consecutive events across these three pathways by directly or indirectly regulating expression of a battery of genes in these pathways (Fig. 7).

### Tryptophan biosynthesis and metabolism pathways

We speculate that the master switch is basically triggered by the ERF109-induced expression of *ASA1* and *YUC2* genes in tryptophan biosynthesis and metabolism pathways, respectively (Fig. 7). The downstream ERF109-regulated genes in the first pathway involve the consecutively functioning *TRP1*, *PAI1*, *IGPS* and *TSA1* genes (Fig. S2). The *ASA1* gene presents in cluster 1 (Fig. 3), while the rest of genes in this pathway present in cluster 2 (Fig. 4). The ERF109-regulated genes in tryptophan metabolism involve the consecutively functioning *TAA1* and *YUC2* genes (Fig. S3). The *TAA1* gene in the present study presents in cluster 2 with upregulation 12 h after salt stress (Fig. 4), while *YUC2* presents in cluster 1 with upregulation 2 h after salt stress (Fig. 3). ERF109 was recently reported to drive expression of *ASA1* gene encoding anthranilate synthase alpha subunit 1 in tryptophan biosynthesis pathway and *YUC2* gene encoding indole-3-pyruvate (or flavin) monooxygenase in tryptophan metabolism pathway^9^. Expression of these two genes participates in the biosynthesis of IAA that is required for triggering the plant hormone signal transduction pathway (Fig. 7). Auxin biosynthesis pathway with tryptophan as a precursor has more than one direction in *Arabidopsis*. The shortest is a two-step reaction due to the expression of *TAA1* gene, encoding tryptophan pyruvate aminotransferase, and *YUC2* gene, encoding flavin monooxygenase^29^. The latter gene catalyzes a rate-limiting step in the pathway^30^. We suggest that *ASA1* gene is also a rate-limiting step in tryptophan biosynthesis pathway (Fig. 7). As ERF109 is driving expression of the latter two genes, it is assumed that expression of this TF is vital for the two pathways.

### Plant hormone signal transduction pathway

The ERF109-regulated genes in the plant hormone signal transduction pathway involve a battery of gene families, e.g., *AUX*/*IAA* (or *IAA*), *ARF*, *TIR1*, *PIN*, *GH3*, *AUX*/*LAX* (or *LAX*), *LBD* and *SAUR* (Figs 7 and S4). These auxin-responsive genes mostly present in cluster 2 (Fig. 4) and pathway results in enlarged cells and improved plant growth. The influence of IAA is cell type-dependent resulting in differential responses at the transcriptional up to the post-translational levels^31^. Auxin signaling is ultimately controlled by the complex of auxin response factors (ARFs) and their interacting repressors AUX/IAA proteins. ARFs bind to promoter elements of auxin responsive genes (e.g., *LAX*, *LBD* and *SAUR*), while AUX/IAA proteins bind to ARFs to block its action, thus negatively regulate transcription of auxin-induced genes^32,33^. A large number of genes, e.g., 29 *AUX*/*IAA* and 23 *ARF*, encode these two heterogeneous, tissue-specific types of controlling elements in *Arabidopsis*^31^. These diverged elements justify the differential role in development processes and their controlling battery of genes^34^. Previous reports indicated that degradation of AUX/IAA proteins in *Arabidopsis* takes place due to the elevated levels of auxin and subsequent increased levels of ARFs^35,36^. AUX/IAA proteins differ in their expression patterns and tissue specificity. For example, five *IAA* genes, namely *IAA9*, *IAA12*, *IAA18*, *IAA19* and *IAA26* are expressed only in the lateral roots. Of which, only *IAA19* was ERF109-upregulated in the present study (Fig. 4). At least, one *IAA* gene in each tissue is required to respond rapidly after exposure to auxin in order to securely act in the different tissues. The encoding/degradation process of AUX/IAA proteins is regulated to promote the continuous expression of the downstream responsive genes^37^. Among *ARF* genes, *ARF19* is the most sensitive to auxin biosynthesis, while *ARF16* is expressed after prolonged exposure. Unlike ARF19, ARF16 is a repressor of expression of downstream responsive genes. Albeit the different functions, the two genes were previously reported to co-express in different plant organs and during different developmental stages^37^. *ARF19* gene is closely-related to *ARF5*, *ARF7* and *ARF8*^34^. The latter four genes act as activators of auxin-responsive gene expression in *Arabidopsis*. *ARF19* is speculated to act as an amplifier of the auxin signal representing a positive-feedback signaling loop^38^. In the present study, these four genes were ERF109-upregulated 12 h after salt stress treatment (Fig. 4) supporting the indirect action of ERF109 in promoting plant growth under normal and adverse conditions. The E3 ubiquitin ligase SCF^TIR1^ (or TIR1) is also auxin-induced and is the main contributor to AUX/IAA protein degradation. This enzyme allows the activation of ARF, derepresses downstream auxin responsive pathways, thus mediates plant growth and development^31^. As the highly conserved domain II of AUX/IAA proteins is a target for degradation process, auxin promotes degradation of all types of AUX/IAA proteins by allowing the participation of auxin-induced TIR1. In the present study, *TIR1* gene was also ERF109-upregulated 12 h after salt stress treatment (Fig. 4) to secure the activation of its target factors, e.g., ARFs, thus, allowing the auxin-responsive downstream genes, *AUX*/*LAX*, *LBD* and *SAUR*, to act (Fig. 7). Active form of auxin functions in its unconjugated form. Towards the homeostasis and regulation of active form of auxin in the cell, *GH3* gene family encodes a class of auxin-induced conjugating enzymes that block action of excessively available auxin^37^. This gene family comprises 19 genes in *Arabidopsis*. Genes encoding group II enzymes, e.g., GH3.1, GH3.3, GH3.4, GH3.5 and GH3.6, and group III enzymes of GH3, e.g., GH3.14 and GH3.17, are upregulated in seedling and root, respectively, to decrease the hormone active form in these tissues. In the present study, only two *GH3* genes, e.g., *GH3.1* and *GH3.3*, of group II and one, e.g., *GH3.17*, of group III in addition to *GH3.2* and *GH3.9* genes were upregulated after 12 h of salt stress treatment. The most rapidly responding gene to auxin is *GH3.3*. Localization of auxin carriers occurs asymmetrically in which influx/efflux process are genetically regulated. Of which, *PIN* gene family acts as auxin efflux carrier. The gene family comprises as little as eight members^39^. For example, *PIN4* gene generates a sink for auxin into columella cells^40^. This action secures the occurrence of auxin gradient and homeostasis to correct root patterning. The present study indicated that *PIN4* gene was ERF109-upregulated after 12 h of salt stress treatment. Interestingly, previous reports indicated that the three *PIN* genes, e.g., *PIN1*, *PIN3* and *PIN7*, are the only members that are upregulated by auxin^37^. The latter genes or gene families, e.g., *TIR1*, *GH3* and *PIN* either participate in degrading AUX/IAA proteins or secure auxin homeostasis under normal and adverse conditions. Thus, these genes allow the activated form of ARF to stimulate expression of downstream genes of growth enhancement such as *like LAX*), *LBD* and *SAUR*. As *PIN* family members act as auxin efflux carriers, *LAX* genes act as auxin influx carriers^41^. The gene family mainly comprises three genes, e.g., *LAX1, LAX2* and *LAX3*, with differential roles and tissue specificities by inducing several downstream genes towards promoting the overall plant growth. In the present study, these three genes were ERF109-upregulated 12 h after salt stress treatment (Fig. 4). The *LBD* genes act towards the induction of lateral root formation and enhancement of LR density^9^. LBD16 and LBD29 genes are considered the most sensitive to auxin presence as they overexpress after as little as 30 min of exogenous or indigenous auxin. Out of the 42 *LBD* genes in *Arabidopsis*, only *LBD16* was ERF109-upregulated in the present study after 12 h of salt stress treatment (Fig. 4). Activated ARF also stimulates the induction of *SAUR* gene family encoding highly unstable mRNAs^42^. The family comprises over 70 genes. Of which, *SAUR62* and *SAUR64-68* were reported to be strongly upregulated by auxin, while clade including *SAUR15*, *SAUR19-22*, *SAUR24* was moderately auxin-responsive. Previous reports indicated that *SAUR36* and *SAUR72* respond negatively to auxin. High expression levels of *SAUR* genes exist in leaves^37^ in accordance with the results of the present finding. *SAUR* genes act as regulators of cell elongation^43^ and stimulators of shoot elongation^37^. In the present study, all these *SAUR* genes were ERF109-upregulated after 12 h of salt stress treatment except for *SAUR36*.

### Putative cis-acting promoter elements

The data shown in Table S3 indicates putative cis-acting promoter elements of several regulated genes of clusters 3 and 4 with binding sites for ERF109 and several other transcription factors of the AP2/ERF gene family. These genes include three *GST* genes (cluster 3) namely *GSTU7*, *GSTU6* and *GSTF8*. As indicated earlier, these genes encode ROS scavenging enzymes and act in blocking Bax protein to avoid PCD and contributes to hormone homeostasis under different environmental conditions. The other two regulated genes (cluster 2) act during tryptophan biosynthesis (e.g., *IGPS*) and plant hormones and signal transduction (e.g., *LAX2*) pathways. The promoters of *GSTF8* and *LAX2* genes harbor four binding sites for ERF109, while *GSTU7* gene harbors three binding sites. Promoter sequences of the *GSTU6* and *IGPS* genes harbor only one binding site for ERF109. This data indicates that ERF109 might act as the master switch for *IGPS* and *LAX2* genes in addition to the previously published *ASA* and *YUC2* genes^10^. These four genes act during the three pathways under study.

In conclusion, we speculate that ERF109 acts under salt stress not only in regulating PCD inhibitors (e.g., BI-1 and DAL1/2) or inducing ROS scavengers, but also as a “master switch” mediator in promoting plant growth and re-adjustment to homeostasis due to the direct participation in auxin biosynthesis. These actions combine to increase the plant’s ability to tolerate salt stress. This conclusion is supported by our recent study in detecting the influence of ERF109 to confer salt stress tolerance in *Arabidopsis*^6^.

## Materials and Methods

### Plant materials

Arabidopsis WT (Col), the knockout T-DNA insertion (SALK_150614) mutant (namely KO^*ERF109*^) and over-expression lines of *ERF109* gene (CS2102255) (OE^*ERF109*^) of locus AT4G34410 were provided by the SALK Institute, Genomic Analysis Laboratory (SIGnAL) (http://signal.salk.edu/tdnaprimers.2.html). Plantlets were grown from seed in a growth chamber for two weeks under the following growth conditions. First, seeds of the three genotypes were surface sterilized, sown in Petri dishes containing MS medium. The plates were kept in the dark at 4 °C for 2 days and then shifted to 21 ± 2 °C (day/night) under light intensity of ~175 umolm-2s-1 and a 16-h-light/8-h-dark cycle where plantlets were allowed to grow for 12 more days. Knockout mutant was screened for homozygosity by standard PCR approach, while seeds of the over-expression line were homozygous. Sequences of PCR primers and reaction conditions were recovered from *Arabidopsis* database (TAIR, http://www.arabidopsis.org/, Table S2).

### Salt stress experiment

The experiment was conducted at the laboratories of the Department of Biological Sciences, KAU, Jeddah, Saudi Arabia. In order to harvest transcriptomes under salt stress, 2-wk-old plantlets of KO^ERF109^ and OE^ERF109^ lines as well as the WT (Col) with homogeneous performance were transferred to pots (9 cm) filled with soil mix (1 soil: 1 vermiculite), where salt stress experiment started (Fig. S5). Two-wk-old control untreated and salt-stressed plantlets were allowed to grow at the above mentioned growth conditions. Control plantlets were irrigated daily with deionized double distilled water and allowed to grow for two more weeks. While, salt-stressed plantlets were initially irrigated daily with salt concentration of 50 mM NaCl for one week. Then, two incremental increases of salt stress was made as recommended by Munns^44^. An increase of 75 mM NaCl was made for 3-wk-old plantlets, which were left to grow for one more week. Another increase of 75 mM NaCl was made for 4-wk-old plantlets which is the target salt concentration (200 mM NaCl) for RNA-Seq analysis. At the same day of reaching the target salt concentration, leaf samples of the 4-wk-old plantlets were harvested after 2 and 12 h. Leaf samples of the control unstressed 4-wk-old plantlets were harvested concurrently with those harvested 2 and 12 h after salt treatment.

### RNA-Seq analysis

RNA was isolated from leaves of different genotypes across time of treatment. Total RNA was extracted from three similar-sized (10 mm^2^) leaf discs per plant (approximately 50 mg tissue) collected from upper leaves using Trizol (Invitrogen) and treated with RNase-free DNase (Promega Inc.). RNA samples were then shipped to BGI, China, for deep sequencing. However, the low quality RNAs isolated from control leaf samples at 2 h time point made them unsuitable for deep sequencing. Therefore, we had to rely only on the leaf samples of control plantlets harvested concurrently with those harvested 12 h after salt stress, only. Raw data were submitted to the NCBI for reviewing and receiving accession numbers. Analysis of the RNA-Seq datasets indicated the recovery of >100 million reads per sample. Adapter sequences were trimmed-off and high quality sequences were aligned (≥2 mismatches) to *Arabidopsis thaliana* genome (http://www.arabidopsis.org/, TAIR version 10) using RSEM v1.1.6 and Bowtie aligner (Bowtie v0.12.1). Then, differential expression and cluster analysis were done by EdgeR (version 3.0.0, R version 2.1.5). Blastx was performed (with an E-value cut off of 1e^−5^) and FPKM values of differentially expressed transcripts were measured against the actin used as the house-keeping gene. Significant Pearson correlation was determined during permutation analysis. Principal component analysis (PCA) was determined using trinity-v2.3.2 PtR module with default parameters. The generated clusters were analyzed for GO terms using Blast2GO (http://www.blast2go.org/). To identify the biological pathways that are active at 12 h time point of salt stress as compared to the untreated control at 12 h time point, the detected genes were mapped to reference canonical pathways in the Kyoto Encyclopedia of Genes and Genomes (KEGG) (http://www.genome.ad.jp/kegg/)^26–28^. Analysis of promoter cis-elements for the genes in the first four clusters was done using Promoter Analysis software (http://plantpan2.itps.ncku.edu.tw/promoter.php) consulting the promoter sequence (up to −500 nt) datasets available at TAIR in order to detect putative binding sites for ERF109 and other TFs in the AP2/ERF gene family.

RNA-Seq datasets were then validated via sqRT-PCR of selected transcripts. First-strand cDNA was synthesized using 2.5 ug of total RNA, 0.5 ug oligo (dT) primer and Superscript II reverse transcriptase (Invitrogen) to a final volume of 20 ul. sqRT-PCR was performed in 20-ul reactions using 1 ul cDNA, 1 × PCR buffer (with 1.5 mM MgCl_2_), 200 uM dNTPs, 200 nM of each gene-specific primers (Table S2) and 0.2 U of Taq DNA polymerase (Promega Inc.). Primers were designed using Netprimer software (http://www.premierbiosoft.com/netprimer/index.html) with the following criteria: length 20–22 bases, GC content ~40–50%, minimal secondary structures, comparable annealing temperatures of the primer pairs, and PCR products of 261–353 bp. Forty PCR cycles for each gene product include denaturation at 94 °C for 15 sec, annealing at appropriate temperature for 30 sec, and extension at 72 °C for 45 sec. Amplicons were analyzed on a 1.2% agarose gel stained with ethidium bromide and visualized using the Gel Doc XR from Bio-Rad Laboratories (Hercules, CA, USA).

## Electronic supplementary material

Supplementary Figures

Supplementary Tables
