# Supplementary material for: Multifunctional activities of ERF109 as affected by salt stress in Arabidopsis
Source: Sci Rep. 2018 Apr 23;8:6403. doi: 10.1038/s41598-018-24452-6 (PMC5913302; doi:10.1038/s41598-018-24452-6)
Supplement: Supplementary file 1 — Supplementary Figures [file 41598_2018_24452_MOESM1_ESM.pdf]

Multifunctional activities of ERF109 as affected by salt stress in Arabidopsis

Ahmed Bahieldin[*1], Ahmed Atef[1], Sherif Edris[1,2,3], Nour O. Gadalla[4,5], Ahmed M. Ramadan[1,6], Sabah M. Hassan[1,2], Sanaa G. Al Attas[1], Magdy A. Al-Kordy[5], Abdulrahman S.M. Al-Hajar[1], Jamal S.M. Sabir[1], Mahmoud E. Nasr[7], Gamal H. Osman[*6,8] and Fotouh M. El-Domyati[2]

1. Department of Biological Sciences, Faculty of Science, King Abdulaziz University (KAU), P.O. Box 80141, Jeddah 21589, Saudi Arabia
2. Department of Genetics, Faculty of Agriculture, Ain Shams University, Cairo, Egypt
3. Princess Al-Jawhara Al-Brahim Centre of Excellence in Research of Hereditary Disorders (PACER-HD), Faculty of Medicine, King Abdulaziz University (KAU), Jeddah, Saudi Arabia
4. Department of Arid Land Agriculture, Faculty of Meteorology, Environment and Arid Land Agriculture, King Abdulaziz University, Jeddah, Saudi Arabia
5. Genetics and Cytology Department, Genetic Engineering and Biotechnology Division, National Research Center, Dokki, Egypt
6. Agricultural Genetic Engineering Research Institute (AGERI), Agriculture Research Center (ARC), Giza, Egypt
7. Faculty of Agriculture, Menofia University, Shebeen Elkom, Egypt
8. Department of Biology, Umm Al-Qura University, Makkah, KSA

Email addresses: bahieldin55@gmail.com; ahmed_atefaig2@yahoo.com; sedris@aucegypt.edu; nouromar71@yahoo.com; ahmedramadan782@yahoo.com; sabmahmoud@yahoo.com; sgalattas@kau.edu.sa; m_alkordy@yahoo.com; ahajarr@gmail.com; jsabir2622@gmail.com; nasr_mi@yahoo.com; geosman@uqu.edu.sa; fm_domyati@hotmail.com

[*] **Corresponding authors:**

Ahmed Bahieldin, Department of Biological Sciences, Faculty of Science, King Abdulaziz University (KAU), P.O. Box 80141, Jeddah 21589, Saudi Arabia, bahieldin55@gmail.com Tel:+966506329922

Gamal Osman, Department of Biology, Umm Al-Qura University, Makkah, KSA, geosman@uqu.edu.sa Tel: 966530760365

(a) *actin* gene

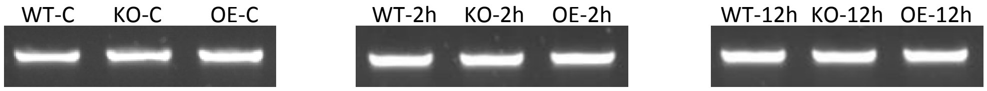

(b) *ERF109* gene

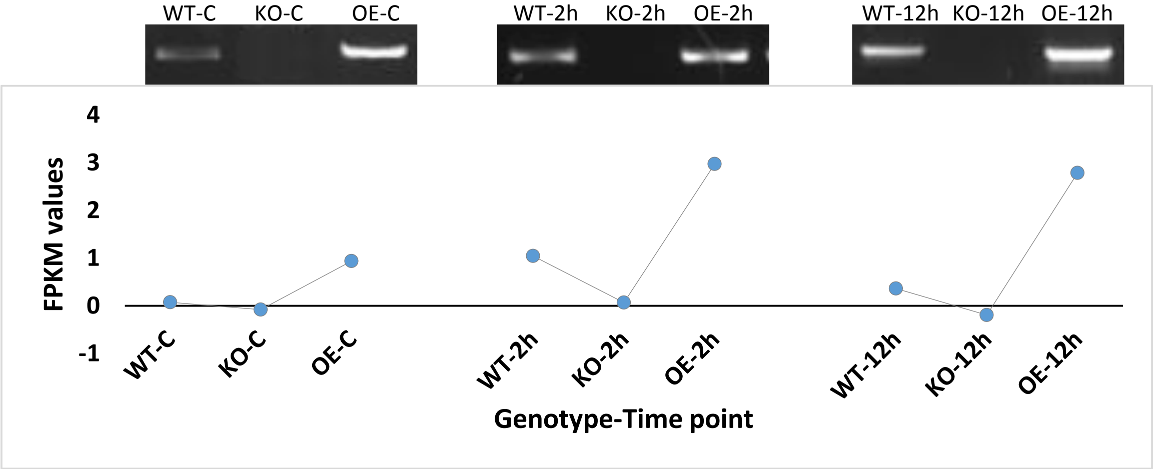

(c) Selected genes of Cluster 2

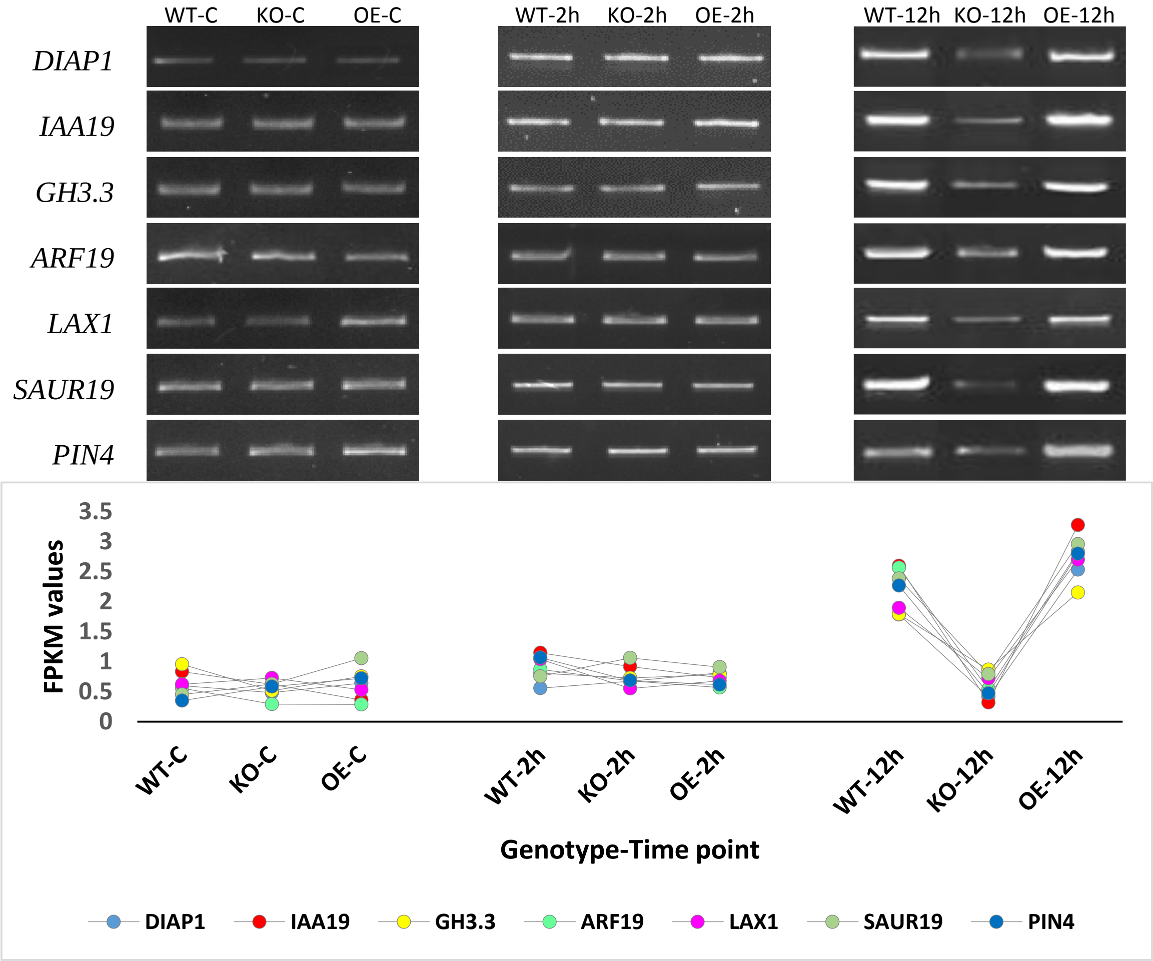

Figure S1| Semi-quantitative RT-PCR and profiles of FPKM values resulting from RNA-Seq analysis for *ERF109* transcript (b) as well as seven selected upregulated transcripts namely *DIAP1*, *IAA19*, *GH3.3*, *ARF19*, *LAX1*, *SAUR19* and *PIN4* (c) of clusters 1 and 2, respectively, used for validating RNA-Seq data of WT, KO^{ERF109} and OE^{ERF109} *Arabidopsis* leaves collected at 2 and 12 h time points of salt stress treatment as well as control (C) leaves. The "*actin*" was used as the unregulated house-keeping gene (a). WT = wild type, C = control untreated plant at 12 h time point, KO = knocked out mutant, OE = overexpressed mutant, 2 h = plants treated with salt stress (200 mM NaCl) for 2 h, while 12 h = plants treated with salt stress (200 mM NaCl) for 12 h.

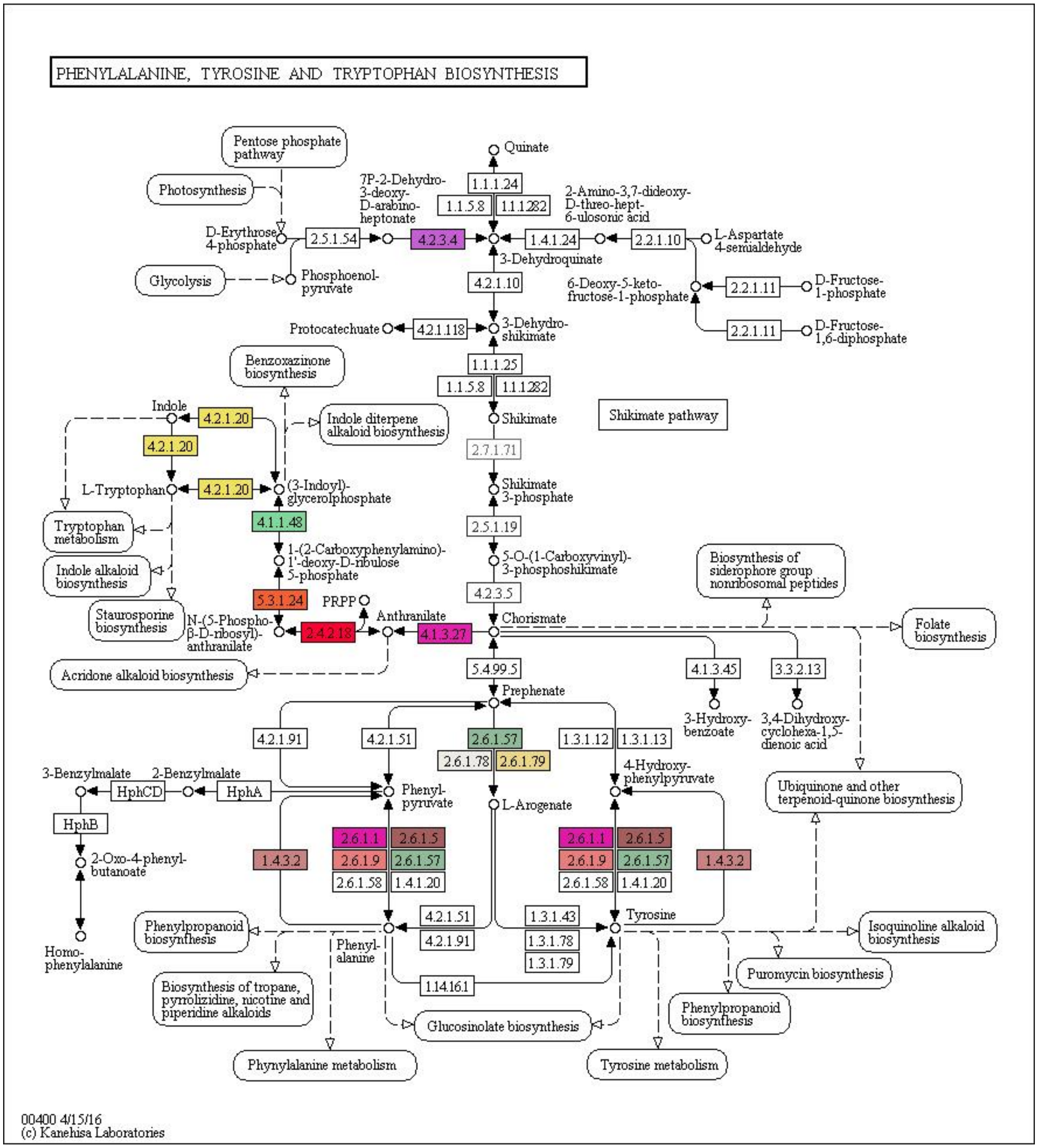

Figure S2| Enzymes in the phenylalanine, tyrosine and tryptophan biosynthesis pathway in leaves positively responded to salt stress treatment (200 mM NaCl) at 12 h time point. Highly activated enzymes are shown in colored boxes, while the enzymes with unchanged activation rates are shown in uncolored boxes. Different box colors in the pathway indicates different highly activated enzymes (Kanehisa et al., 26-28).

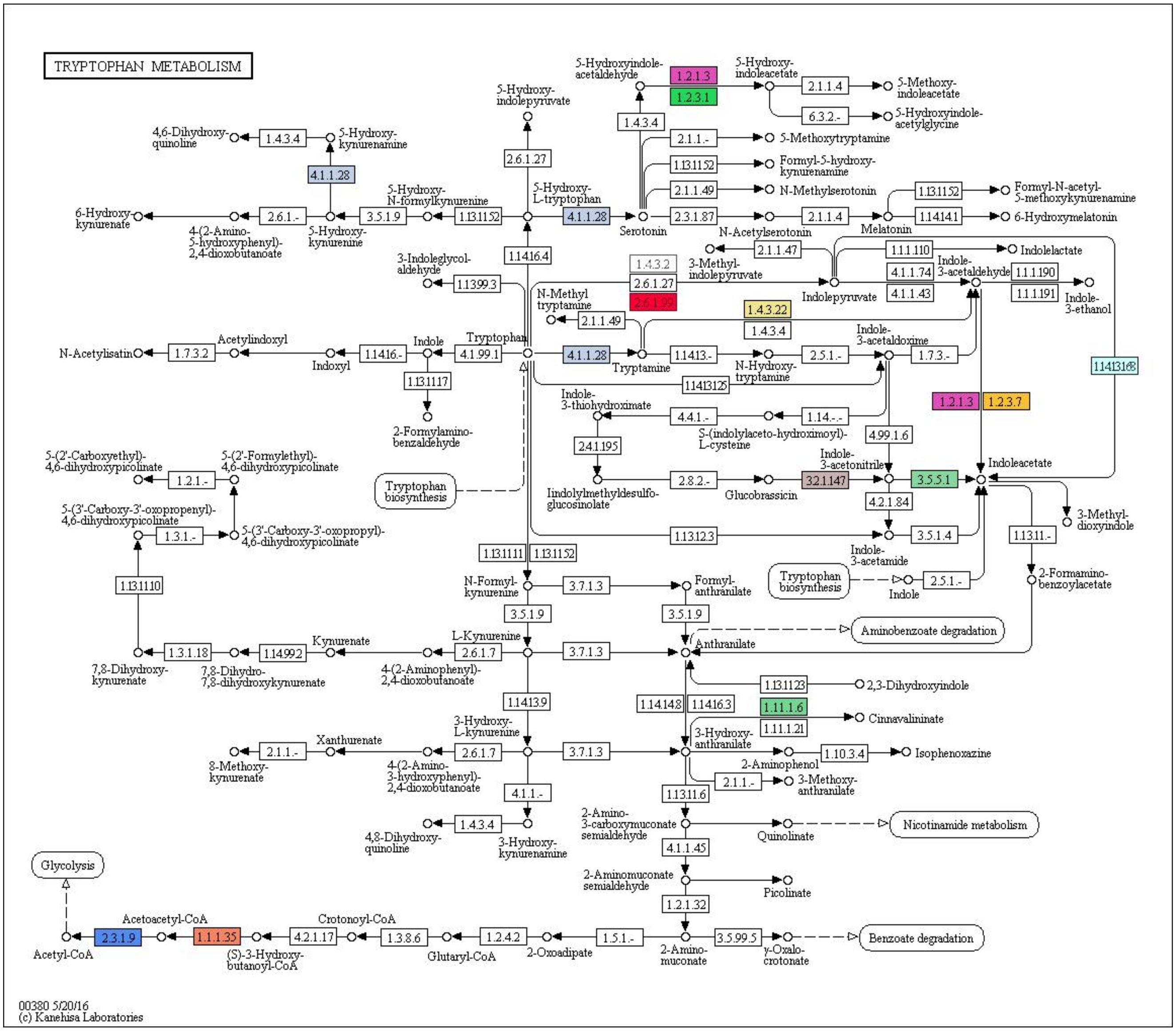

Figure S3| Enzymes in the tryptophan metabolism pathway in leaves positively responded to salt stress treatment (200 mM NaCl) at 12 h time point. Highly activated enzymes are shown in colored boxes, while the enzymes with unchanged activation rates are shown in uncolored boxes. Different box colors in the pathway indicates different highly activated enzymes (Kanehisa et al., 26-28).

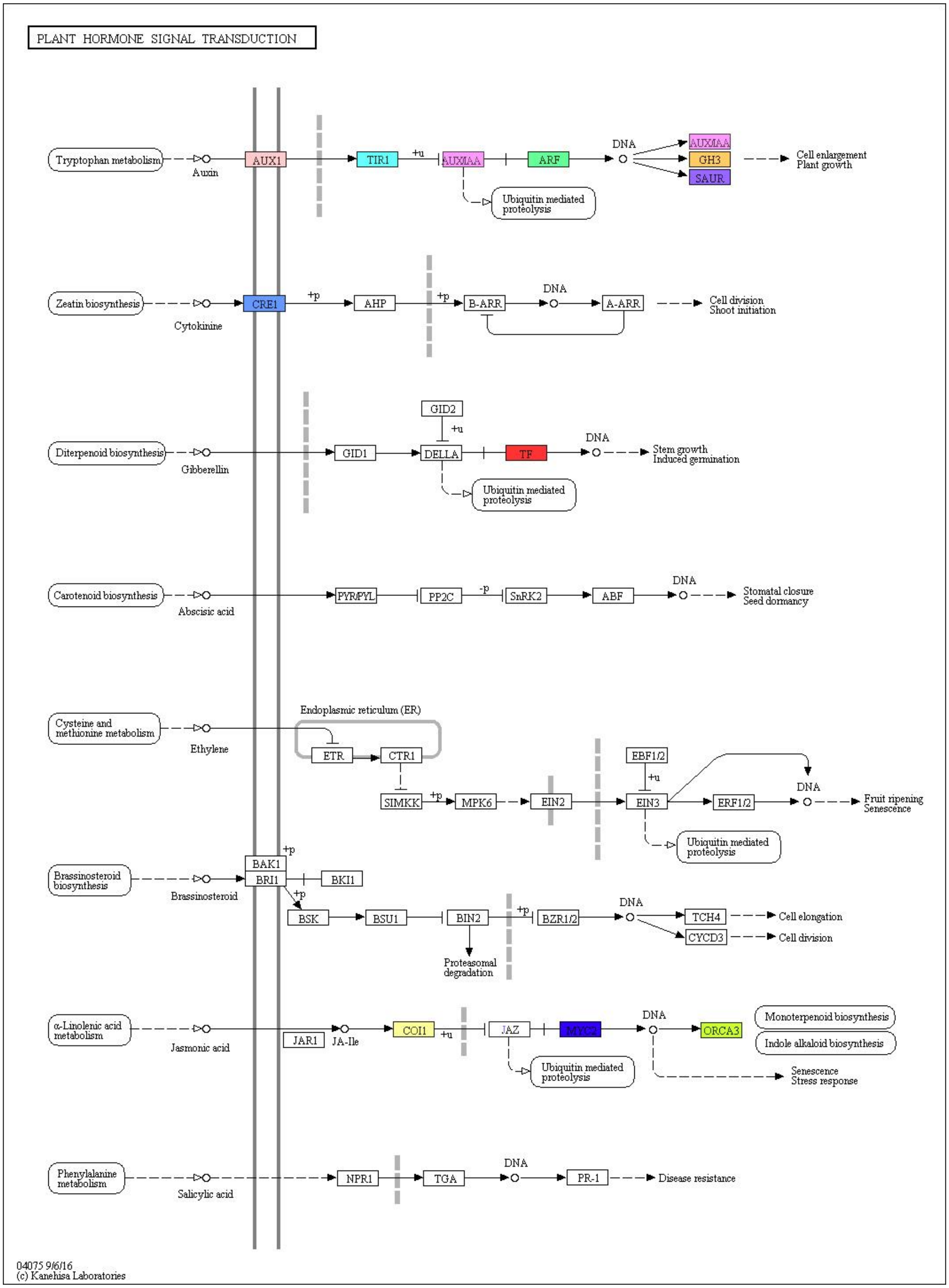

Figure S4| Enzymes in the plant hormone signal transduction pathway in leaves positively responded to salt stress treatment (200 mM NaCl) at 12 h time point. Highly activated enzymes are shown in colored boxes, while the enzymes with unchanged activation rates are shown in uncolored boxes. Different box colors in the pathway indicates different highly activated enzymes (Kanehisa et al., 26-28).

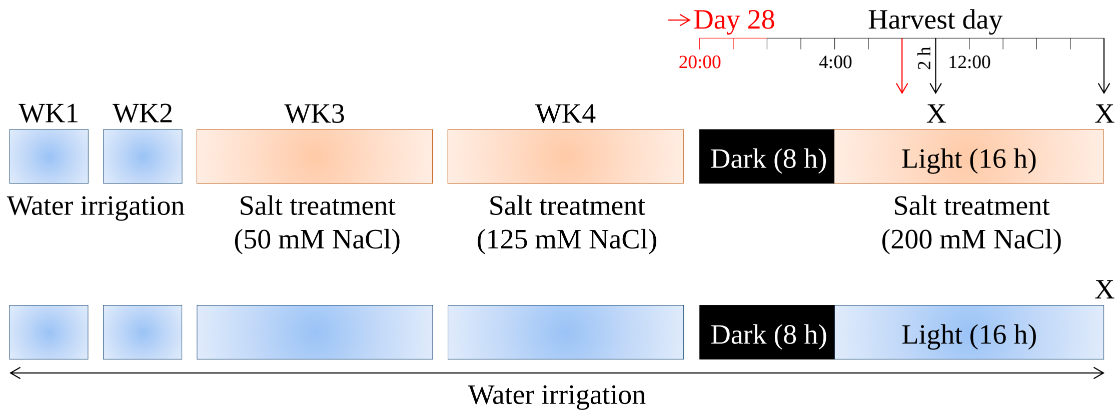

Figure S5| Schematic representation of the setup of salt stress experiment indicating the time of salt treatment at the harvest day (indicated by vertical red arrow), harvest time points (indicated by vertical black arrows) for salt-treated and untreated samples (X) across photoperiod of the harvest day.
