# Supplementary material for: Multifunctional activities of ERF109 as affected by salt stress in Arabidopsis
Source: Sci Rep. 2018 Apr 23;8:6403. doi: 10.1038/s41598-018-24452-6 (PMC5913302; doi:10.1038/s41598-018-24452-6)
Supplement: Supplementary file 2 — Supplementary Tables [file 41598_2018_24452_MOESM2_ESM.pdf]

Multifunctional activities of ERF109 as affected by salt stress in Arabidopsis

Ahmed Bahieldin[*1], Ahmed Atef[1], Sherif Edris[1,2,3], Nour O. Gadalla[4,5], Ahmed M. Ramadan[1,6], Sabah M. Hassan[1,2], Sanaa G. Al Attas[1], Magdy A. Al-Kordy[5], Abdulrahman S.M. Al-Hajar[1], Jamal S.M. Sabir[1], Mahmoud E. Nasr[7], Gamal H. Osman[*6,8] and Fotouh M. El-Domyati[2]

1. Department of Biological Sciences, Faculty of Science, King Abdulaziz University (KAU), P.O. Box 80141, Jeddah 21589, Saudi Arabia
2. Department of Genetics, Faculty of Agriculture, Ain Shams University, Cairo, Egypt
3. Princess Al-Jawhara Al-Brahim Centre of Excellence in Research of Hereditary Disorders (PACER-HD), Faculty of Medicine, King Abdulaziz University (KAU), Jeddah, Saudi Arabia
4. Department of Arid Land Agriculture, Faculty of Meteorology, Environment and Arid Land Agriculture, King Abdulaziz University, Jeddah, Saudi Arabia
5. Genetics and Cytology Department, Genetic Engineering and Biotechnology Division, National Research Center, Dokki, Egypt
6. Agricultural Genetic Engineering Research Institute (AGERI), Agriculture Research Center (ARC), Giza, Egypt
7. Faculty of Agriculture, Menofia University, Shebeen Elkom, Egypt
8. Department of Biology, Umm Al-Qura University, Makkah, KSA

Email addresses: bahieldin55@gmail.com; ahmed_atefaig2@yahoo.com; sedris@aucegypt.edu; nouromar71@yahoo.com; ahmedramadan782@yahoo.com; sabmahmoud@yahoo.com; sgalattas@kau.edu.sa; m_alkordy@yahoo.com; ahajarr@gmail.com; jsabir2622@gmail.com; nasr_mi@yahoo.com; geosman@uqu.edu.sa; fm_domyati@hotmail.com

[*] **Corresponding authors:**

Ahmed Bahieldin, Department of Biological Sciences, Faculty of Science, King Abdulaziz University (KAU), P.O. Box 80141, Jeddah 21589, Saudi Arabia, bahieldin55@gmail.com Tel:+966506329922

Gamal Osman, Department of Biology, Umm Al-Qura University, Makkah, KSA, geosman@uqu.edu.sa Tel: 966530760365

Table S1. Cluster analysis indicating FPKM values of assembled leaf transcripts of three Arabidopsis genotypes differing in ERF109 gene expression levels treated with NaCl (200 mM) for 2 and 12 h as well as the untreated control. Wild type=WT, C=control, KO=ERF109-knockout mutant, OE=ERF109-overexpressing line. Green raws in clusters 1, 2 and 3 refer to genes initially triggered by ERF109.

Cluster 1	Description
AT4G34410.1	ERF109_ARATHEthylene-responsive transcription factor ERF109 OS=Arabidopsis thaliana GN=ERF109 PE=1 SV=1
AT1G03070.1	BI-1 bax inhibitor-1 family protein
AT5G05730.2	TRPE_ARATHAnthranilate synthase alpha subunit 1 chloroplastic OS=Arabidopsis thaliana GN=ASA1 PE=1 SV=1
AT4G13260	YUC2_Indole-3-pyruvate monooxygenase OS=Arabidopsis thaliana GN=YUC2 PE=1 SV=1
**Cluster 2**	**Description**
AT3G12830.1	SAU72_ARATHAuxin-responsive SAUR72 OS=Arabidopsis thaliana GN=SAUR72 PE=2 SV=1
AT1G62370.1	DIAP1_DROMEDeath-associated inhibitor of apoptosis 1 OS=Drosophila melanogaster GN=DIAP1 (DEL1/2) PE=1 SV=2
AT1G07780.1	PAI1_ARATHN-(5 -phosphoribosyl)anthranilate isomerase chloroplastic OS=Arabidopsis thaliana GN=PAI1 PE=2 SV=1
AT4G14550.1	IAA14_ARATHAuxin-responsive IAA14 OS=Arabidopsis thaliana GN=IAA14 PE=1 SV=1
AT3G54640.1	TSA1_ARATHtryptophan synthase alpha subunit 1 OS=Arabidopsis thaliana GN=TSA1 PE=1 SV=1
AT5G17990.1	TRP1_ARATHanthranilate phosphoribosyltransferase OS=Arabidopsis thaliana GN=TRP1 PE=1 SV=2
AT3G15540.1	IAA19_ARATHAuxin-responsive IAA19 OS=Arabidopsis thaliana GN=IAA19 PE=1 SV=2
AT2G04400.1	IGPS_ARATHIndole-3-glycerol phosphate synthase, chloroplastic OS=Arabidopsis thaliana GN=IGPS PE=2 SV=1
AT2G23170.1	GH3.3_ARATHIndole-3-acetic acid-amido synthetase OS=Arabidopsis thaliana GN=GH3.3 PE=1 SV=1
AT1G19220.1	ARFB_ARATHAuxin response factor 19 OS=Arabidopsis thaliana GN=ARF19 PE=1 SV=2
AT5G01240.1	LAX1_ARATHAuxin transporter 1 OS=Arabidopsis thaliana GN=LAX1 PE=2 SV=1
AT1G30330.1	ARFF_ARATHAuxin response factor 6 OS=Arabidopsis thaliana GN=ARF6 PE=1 SV=2
AT4G37390.1	GH3.2_ARATHIndole-3-acetic acid-amido synthetase OS=Arabidopsis thaliana GN=GH3.2 PE=1 SV=3
AT2G21050.1	LAX2_ARATHAuxin transporter 2 OS=Arabidopsis thaliana GN=LAX2 PE=2 SV=1
AT1G19850.1	ARFE_ARATHAuxin response factor 5 OS=Arabidopsis thaliana GN=ARF5 PE=1 SV=3
AT1G77690.1	LAX3_ARATHAuxin transporter 3 OS=Arabidopsis thaliana GN=LAX3 PE=2 SV=1
AT1G70560.1	TAA1_ARATHtryptophan---pyruvate aminotransferase OS=Arabidopsis thaliana GN=TAA1 PE=1 SV=1
AT2G14960.1	GH3.1_ARATHProbable indole-3-acetic acid-amido synthetase OS=Arabidopsis thaliana GN=GH3.1 PE=2 SV=1
AT5G37020.2	ARFH_ARATHAuxin response factor 8 OS=Arabidopsis thaliana GN=ARF8 PE=2 SV=2
AT2G46530.1	ARFK_ARATHAuxin response factor 11 OS=Arabidopsis thaliana GN=ARF11 PE=2 SV=3
AT5G13360.3	GH3.17_ARATHIndole-3-acetic acid-amido synthetase OS=Arabidopsis thaliana GN=GH3.17 PE=1 SV=1
AT5G20730.1	ARFG_ARATHAuxin response factor 7 OS=Arabidopsis thaliana GN=ARF7 PE=1 SV=2
AT4G28640.2	IAA11_ARATHAuxin-responsive IAA11 OS=Arabidopsis thaliana GN=IAA11 PE=1 SV=1
AT4G32280.1	IAA29_ARATHAuxin-responsive IAA29 OS=Arabidopsis thaliana GN=IAA29 PE=2 SV=2
AT1G28130.2	GH3.17_ARATHIndole-3-acetic acid-amido synthetase OS=Arabidopsis thaliana GN=GH3.17 PE=1 SV=1
AT1G15580.1	IAA5_ARATHAuxin-responsive IAA5 OS=Arabidopsis thaliana GN=IAA5 PE=2 SV=3
AT3G23050.1	IAA7_ARATHAuxin-responsive IAA7 OS=Arabidopsis thaliana GN=IAA7 PE=1 SV=1
AT5G43700.1	IAA4_ARATHAuxin-responsive IAA4 OS=Arabidopsis thaliana GN=IAA4 PE=1 SV=2
AT3G23050.2	IAA7_ARATHAuxin-responsive IAA7 OS=Arabidopsis thaliana GN=IAA7 PE=1 SV=1
AT2G22670.2	IAA8_ARATHAuxin-responsive IAA8 OS=Arabidopsis thaliana GN=IAA8 PE=1 SV=1
AT4G14560.1	IAA1_ARATHAuxin-responsive IAA1 OS=Arabidopsis thaliana GN=IAA1 PE=1 SV=2
AT5G51450.2	TIR1_ARATHE3 ubiquitin ligase RIN3 OS=Arabidopsis thaliana GN=TIR1 PE=1 SV=2
AT4G38840.1	SAU20_ARATHAuxin-responsive SAUR20 OS=Arabidopsis thaliana GN=SAUR20 PE=2 SV=1
AT1G29510.1	SAU67_ARATHAuxin-responsive SAUR67 OS=Arabidopsis thaliana GN=SAUR67 PE=2 SV=1
AT5G18010.1	SAU19_ARATHAuxin-responsive SAUR19 OS=Arabidopsis thaliana GN=SAUR19 PE=2 SV=1
AT5G18080.1	SAU24_ARATHAuxin-responsive SAUR24 OS=Arabidopsis thaliana GN=SAUR24 PE=2 SV=1
AT5G18060.1	SAU23_ARATHAuxin-responsive SAUR23 OS=Arabidopsis thaliana GN=SAUR23 PE=2 SV=1
AT1G29490.1	SAU68_ARATHAuxin-responsive SAUR68 OS=Arabidopsis thaliana GN=SAUR68 PE=3 SV=1
AT1G29430.1	SAU62_ARATHAuxin-responsive SAUR62 OS=Arabidopsis thaliana GN=SAUR62 PE=2 SV=1
AT1G29460.1	SAU65_ARATHAuxin-responsive SAUR65 OS=Arabidopsis thaliana GN=SAUR65 PE=2 SV=1
AT1G29500.1	SAU66_ARATHAuxin-responsive SAUR66 OS=Arabidopsis thaliana GN=SAUR66 PE=2 SV=1
AT5G18050.1	SAU22_ARATHAuxin-responsive SAUR22 OS=Arabidopsis thaliana GN=SAUR22 PE=2 SV=1
AT1G52830.1	IAA6_ARATHAuxin-responsive IAA6 OS=Arabidopsis thaliana GN=IAA6 PE=1 SV=2
AT2G47750.1	GH3.9_ARATH indole-3-acetic acid-amido synthetase OS=Arabidopsis thaliana GN=G3.9 PE=2 SV=1
At2g42430.1	LBD16_ARATHLOB domain-containing 1 OS=Arabidopsis thaliana GN=LBD16 PE=2 SV=1
AT5G53590.1	SAU32_ARATHAuxin-responsive SAUR32 OS=Arabidopsis thaliana GN=SAUR32 PE=2 SV=1
AT5G50760.1	SAU71_ARATHAuxin-responsive SAUR71 OS=Arabidopsis thaliana GN=SAUR71 PE=2 SV=1
AT4G30080.2	ARFA_ARATHAuxin response factor 16 OS=Arabidopsis thaliana GN=ARF16 PE=1 SV=2
AT2G28085.1	SAU32_ARATHAuxin-responsive SAUR32 OS=Arabidopsis thaliana GN=SAUR32 PE=2 SV=1
AT4G38850.1	SAU15_ARATHAuxin-responsive SAUR15 OS=Arabidopsis thaliana GN=SAUR15 PE=2 SV=1
AT1G29440.1	SAU63_ARATHAuxin-responsive SAUR63 OS=Arabidopsis thaliana GN=SAUR63 PE=2 SV=1
AT1G29450.1	SAU64_ARATHAuxin-responsive SAUR64 OS=Arabidopsis thaliana GN=SAUR64 PE=2 SV=1
AT4G38820.1	SAU21_ARATHAuxin-responsive SAUR21 OS=Arabidopsis thaliana GN=SAUR21 PE=2 SV=1
AT2G01420.2	PIN4_ARATHAuxin efflux carrier component 4 OS=Arabidopsis thaliana GN=PIN4 PE=1 SV=1
**Cluster 3**	**Description**
AT1G53680.1	GSTUS_ARATHGlutathione S-transferase U28 OS=Arabidopsis thaliana GN=GSTU28 PE=3 SV=1
AT2G29460.1	GSTU4_ARATHGlutathione S-transferase U4 OS=Arabidopsis thaliana GN=GSTU4 PE=1 SV=1
AT2G29490.1	GSTU1_ARATHGlutathione S-transferase U1 OS=Arabidopsis thaliana GN=GSTU1 PE=2 SV=1
AT2G29420.1	GSTU7_ARATHGlutathione S-transferase U7 OS=Arabidopsis thaliana GN=GSTU7 PE=2 SV=1
AT1G75270.1	DHAR2_ARATHGlutathione S-transferase DHAR2 OS=Arabidopsis thaliana GN=DHAR2 PE=1 SV=1
AT4G11600.1	GPX6_ARATHProbable phospholipid hydroperoxide glutathione peroxidase mitochondrial OS=Arabidopsis thaliana GN=PHGPX6 PE=2 SV=2
AT1G17170.1	GSTUO_ARATHGlutathione S-transferase U24 OS=Arabidopsis thaliana GN=GSTU24 PE=2 SV=1
AT2G29470.1	GSTU3_ARATHGlutathione S-transferase U3 OS=Arabidopsis thaliana GN=GSTU3 PE=2 SV=1
AT2G02930.1	GSTF3_ARATHGlutathione S-transferase F3 OS=Arabidopsis thaliana GN=GSTF3 PE=2 SV=1
AT2G02390.2	GSTZ1_ARATHGlutathione S-transferase Z1 OS=Arabidopsis thaliana GN=GSTZ1 PE=1 SV=1
AT2G29440.1	GSTU6_ARATHGlutathione S-transferase U6 OS=Arabidopsis thaliana GN=GSTU6 PE=2 SV=1
AT2G02390.3	GSTZ1_ARATHGlutathione S-transferase Z1 OS=Arabidopsis thaliana GN=GSTZ1 PE=1 SV=1
AT2G29450.1	GSTU5_ARATHGlutathione S-transferase U5 OS=Arabidopsis thaliana GN=GSTU5 PE=2 SV=1
AT2G47730.1	GSTF8_ARATHGlutathione S-transferase chloroplastic OS=Arabidopsis thaliana GN=GSTF8 PE=1 SV=3
AT4G08390.3	APXS_ARATHL-ascorbate peroxidase chloroplastic mitochondrial OS=Arabidopsis thaliana GN=APXS PE=1 SV=2
**Cluster 4**	**Description**
AT1G76530.1	auxin efflux carrier (AEC) family [Medicago truncatula]
AT3G62100.1	IAA30_ARATHAuxin-responsive IAA30 OS=Arabidopsis thaliana GN=IAA30 PE=2 SV=1
AT4G12410.1	SAU36_ARATHAuxin-responsive SAUR36 OS=Arabidopsis thaliana GN=SAUR36 PE=2 SV=1
AT4G12410.1	SAU36_ARATHAuxin-responsive SAUR36 OS=Arabidopsis thaliana GN=SAUR36 PE=2 SV=1
**Cluster 5**	**Description**
AT2G34070.1	TBL37_ARATH trichome birefringence-like 37 OS=Arabidopsis thaliana GN=TBL37 PE=2 SV=2
AT3G22600.1	NLTL2_ARATHNon-specific lipid-transfer At2g13820 OS=Arabidopsis thaliana GN=At2g13820 PE=1 SV=1
AT4G05100.1	MYB39_ARATHTranscription factor MYB39 OS=Arabidopsis thaliana GN=MYB39 PE=2 SV=1
AT5G04340.1	ZAT6_ARATHZinc finger ZAT6 OS=Arabidopsis thaliana GN=ZAT6 PE=2 SV=1
AT2G21940.1	SK1_ARATHShikimate kinase chloroplastic OS=Arabidopsis thaliana GN=SK1 PE=1 SV=2
AT4G15530.5	phosphate dikinase chloroplastic OS=Arabidopsis thaliana GN=PPDK PE=1 SV=2
AT2G44530.1	KPRS5_ARATHRibose-phosphate pyrophosphokinase chloroplastic OS=Arabidopsis thaliana GN=PRS5 PE=2 SV=2
AT1G52890.1	NAC19_ARATHNAC domain-containing 19 OS=Arabidopsis thaliana GN=NAC019 PE=1 SV=1
AT2G43570.1	CHI_ARATHEndochitinase CHI OS=Arabidopsis thaliana GN=CHI PE=2 SV=1
AT1G07430.1	P2C03_ARATH phosphatase 2C 3 OS=Arabidopsis thaliana GN=AIP1 PE=1 SV=1
AT1G67360.1	Y1736_ARATHREF SRPP At1g67360 OS=Arabidopsis thaliana GN=At1g67360 PE=2 SV=1
AT5G63130.1	octicosapeptide phox Bem1p domain kinase superfamily [Medicago truncatula]
**Cluster 6**	**Description**
AT5G50360.1	PREDICTED: uncharacterized protein LOC103431109
AT3G57020.2	SSL9_ARATH STRICTOSIDINE SYNTHASE-LIKE 9 OS=Arabidopsis thaliana GN=SSL9 PE=2 SV=1
AT5G58330.2	MDHP_MEDSAMalate dehydrogenase
AT1G08110.1	LGUL_ARATHLactoylglutathione lyase OS=Arabidopsis thaliana GN=At1g08110 PE=2 SV=1
AT1G80160.1	FOSB_OCEIHMetallothiol transferase OS=Oceanobacillus iheyensis (strain DSM 14371 JCM 11309 KCTC 3954 HTE831) GN=fosB PE=3 SV=2
AT5G45428.1	CDKF4_ORYSJCyclin-dependent kinase F-4 OS=Oryza sativa japonica GN=CDKF-4 PE=2 SV=1
AT5G45630.1	nucleolin-like [Malus domestica]
**Cluster 7**	**Description**
AT4G29930.1	BH027_ARATHTranscription factor bHLH27 OS=Arabidopsis thaliana GN=BHLH27 PE=2 SV=1
AT5G65210.1	TGA1_ARATHTranscription factor TGA1 OS=Arabidopsis thaliana GN=TGA1 PE=1 SV=2

WT-C	KO-C	OE-C	WT-2h	KO-2h	OE-2h	WT-12h	KO-12h	OE-12h
0.075225631	-0.080723254	0.93973876	1.048779044	0.071185693	2.972750375	0.362227496	-0.192199475	2.789008895
0.191720869	-0.158357227	0.749818039	1.34587412	0.342960323	2.773940941	0.74208013	0.043308101	1.310303669
0.478835108	-0.134595418	0.710687579	1.423845917	0.521212231	1.904385719	0.91120372	0.145111708	1.923855152
0.144170171	0.081366679	0.498454934	1.602285638	0.830183642	2.416465146	0.577434157	0.154867515	0.954801239
**WT-C**	**KO-C**	**OE-C**	**WT-2h**	**KO-2h**	**OE-2h**	**WT-12h**	**KO-12h**	**OE-12h**
0.729835374	0.624944258	0.471906826	0.647759662	0.352929676	0.569340787	2.216905796	0.551289541	2.764488545
0.598542711	0.481876342	0.641142862	0.559312936	0.673026982	0.814833564	1.78353343	0.413557738	2.532569737
0.358076903	0.522023397	0.514028376	1.075501791	0.472904225	0.887598239	2.0269187	0.814337226	2.452074266
0.777853929	0.694557433	0.835000439	0.911331142	0.502188724	0.69571576	2.104059554	1.027570929	2.47029839
0.317083848	0.321026652	0.934524427	0.972232224	0.563021963	0.539820714	2.018898786	0.906815997	2.483773416
0.715537577	0.282055223	0.29442993	0.728057503	0.890049149	0.425260819	2.572345087	0.922667845	3.142159647
0.83220199	0.618784048	0.361606514	1.149824041	0.91686388	0.733453188	2.598903358	0.320966783	3.27839735
0.769630327	0.709846613	0.423612806	0.379360711	0.610026308	0.72503302	2.540495133	0.76747396	3.206966085
0.953449139	0.516579601	0.755572437	0.809274903	0.727952485	0.783918355	1.789155519	0.865756853	2.153148825
0.560064836	0.294429988	0.287209306	0.87201486	0.67775584	0.568185505	2.563426577	0.539221482	2.831606712
0.620787767	0.727464943	0.531015159	1.040810721	0.549862932	0.681704148	1.896698832	0.722681776	2.697718476
1.002723961	0.727242933	0.833041886	0.589646921	0.669515446	0.925157562	1.808443881	0.617326918	2.594330566
0.85546546	0.348301088	0.309602127	0.435541521	0.792426095	0.626276962	1.779605332	0.790488429	1.998316765
0.697640935	0.650158932	0.595627968	1.052582168	0.400196541	0.958969795	1.926442384	0.837987848	3.079329224
0.984507602	0.85257997	0.276454319	1.040527893	0.951105137	0.922175709	1.759061071	0.522751613	2.877534318
0.730629885	0.444913194	0.486115605	0.817842109	0.73736127	0.869501199	1.839467969	0.742860021	2.250126377
0.650336962	0.529752562	0.687517534	0.620574371	0.763039865	0.56985159	1.952986959	0.859418415	2.550992688
0.928914178	0.931299359	0.487595016	0.496175924	0.531539471	0.817737327	1.712029446	0.49454718	2.030937909
0.520917662	0.912495765	0.419616582	0.953490648	0.403662984	0.856215826	1.659948718	1.013998772	2.281462843
0.72866077	0.885552411	0.480551813	1.053604944	0.78519027	0.706502544	2.090382229	1.009036657	2.818291765
0.856309362	0.501570078	0.917969745	0.799093446	0.521458929	0.735367162	2.029868225	0.776350646	2.345873316
0.55871588	0.802703577	0.588755752	1.013879577	0.48525014	0.428557641	2.115028492	0.559320556	2.830536461
0.57743948	0.774916809	0.834752893	0.448166719	0.72945242	0.918382466	1.684118786	0.715771988	2.044275659
0.838961115	0.830337664	0.555640039	0.681494482	0.552862479	0.594644206	1.869752268	0.713216176	2.06798597
0.582956043	0.503325056	0.645153327	0.736188073	0.356706502	0.940036599	2.530795462	0.804590872	3.127524376
0.944392657	0.543865052	0.645528633	0.744906291	0.508769779	1.003962274	2.172319723	0.658586331	2.704257244
0.730527728	0.315347747	0.497872916	0.714625819	0.371808479	0.87072165	2.033668162	0.870336682	2.492380048
0.841428347	0.444507543	0.446152632	0.578691565	0.855791451	0.991268006	2.411776258	0.718794508	2.776258289
0.651206376	0.551520074	0.969875352	0.673380045	0.586963092	0.805655604	1.852400363	0.745799625	2.359321738
0.58040484	0.532980701	1.069693903	0.991871259	0.642826326	0.734566526	1.700715106	0.603853986	2.097034676
0.420657677	0.572571622	0.489969796	0.547808624	0.355629057	0.85617507	1.861085894	0.654655436	2.62235404
0.866500885	0.632223802	0.981808868	0.506322535	0.396570335	0.696106661	2.076482614	0.555429699	2.446863948
0.418280146	0.791058149	0.82718017	0.45765007	0.459099882	0.868655228	1.654199569	0.693025379	2.42367232
0.901803308	0.395769416	0.543733422	0.425520173	0.787998316	0.64045996	2.120029739	0.786369585	2.513051975
0.453443272	0.627507803	1.058934988	0.75601546	1.05974096	0.906863214	2.384595802	0.792562356	2.95688311
0.600854171	0.536546931	0.637786502	0.687145755	0.415258885	0.475866936	2.017955117	0.914462192	2.226476059
0.643030052	0.65451597	0.754778904	0.880842028	0.341031145	0.528881604	1.671948082	0.97537363	2.468628931
0.616768374	0.724797058	0.830237872	0.502788304	0.334493417	0.839139399	1.996567633	0.753867423	2.506403146
0.53476985	0.431810862	0.656029395	0.588953357	0.855075481	0.949131575	2.071538218	0.734842936	2.295851041
0.343220053	0.299478389	0.491926301	0.791797159	0.344942997	0.912909581	1.620429379	0.495356792	2.04903947
0.58592197	0.475138104	0.72730096	1.006328528	0.44087606	0.542263116	2.24065289	0.553482024	2.591385842
0.854656389	0.469462868	0.843498623	0.953241304	0.534245752	0.807197726	2.291360406	0.39615751	2.808184765
0.568854378	0.818277445	0.411749759	0.459768806	0.723367706	0.723819143	1.774161609	0.825524694	2.157947194
0.514544505	0.423191467	0.534418326	0.572505095	0.325322777	0.462203837	1.542997681	0.40320438	2.158666317
0.890635592	0.274304454	0.422926864	0.419742122	0.469720776	0.580769493	2.224484368	0.620985345	2.401852396
0.716248045	0.397788851	0.311176518	0.412479849	0.531284086	0.867997807	2.587970923	0.879709233	2.93391199
0.81521175	0.519862895	0.374742519	0.444027775	0.898856209	0.848851491	2.08032726	0.643915247	2.286644649
0.45067971	0.363853416	0.405737396	0.93352972	0.901971711	0.473507892	2.37847897	0.784789699	2.595933693
0.746717459	0.257116081	0.628408515	0.442614592	0.69837977	0.758027607	2.261947495	0.842130773	2.432480358
0.636005688	0.238675938	0.294894369	0.305037309	0.53124509	0.795779427	2.068978154	0.742538065	2.519156371
0.4276191	0.33592049	0.442211143	0.612566088	0.623852846	0.771826437	2.01969041	0.70875342	2.399072047
0.642746018	0.44068241	0.693718304	0.608316296	0.673233453	0.767903112	1.948058946	1.124244119	2.471869883
0.765062304	0.385619967	0.706325262	0.781222478	1.003407854	0.634226587	2.517829014	1.105907968	2.793609602
0.35302652	0.584099139	0.72073549	1.068378897	0.684711585	0.615623901	2.26684322	0.47183894	2.799795045
**WT-C**	**KO-C**	**OE-C**	**WT-2h**	**KO-2h**	**OE-2h**	**WT-12h**	**KO-12h**	**OE-12h**
-0.436361074	-0.383949721	-0.053432089	2.580205296	-0.862028033	3.324228986	-1.17152009	-0.941849606	-1.779349735
-1.135846534	-0.494343818	-2.56911696	2.189276801	-0.74833208	2.94170614	-3.009922156	-3.166140541	-1.902559636
-2.337067201	-0.555901597	-1.511250722	2.796112181	-0.559098257	2.980074873	-3.167964599	-2.493486549	-3.078053682
-1.475176047	-0.232891733	-1.047064243	3.018986067	-0.491407699	3.064804245	-2.34912528	-1.819918551	-2.161286203
-1.678559739	0.16062462	-1.368385741	2.965507126	-1.095870175	2.983130932	-2.419164319	-1.695381319	-2.137532695
-1.950487518	-1.230909964	-2.577992214	2.919373816	-1.035136395	2.974459372	-2.942811315	-2.794613136	-2.950934828
-2.522509798	-1.118077061	-1.516304779	3.43675161	-1.02249375	3.440562458	-3.360650269	-2.62157747	-3.074471717
-2.905545381	-3.723192869	-3.235802189	2.181486672	-0.506331273	2.653357571	-0.756993754	-1.493731236	-0.435613276
-1.000242284	-1.165028562	-0.895995416	2.103597962	0.151387652	1.823612038	-1.011613355	-1.716636057	-1.411614501
-1.112503101	-0.980189752	-2.004581529	2.229806896	-1.159476143	1.860229841	-2.228868312	-2.468401331	-2.449517619
-1.005897501	-0.164263097	-1.095074814	2.167703981	0.689000912	1.809272887	-2.450411738	-2.066499962	-1.085955894
-1.102470034	-0.969915375	-1.395546307	1.803852323	-1.100849981	2.280419205	-2.130736022	-1.396568484	-2.125748718
-0.15677669	-0.080624358	-0.646948914	1.902121721	0.537157147	1.736047052	-1.749597619	-1.568846095	-2.256882947
0.093347134	-0.7009422	-0.865091188	1.862901519	-0.65769438	1.624463752	-1.022014282	-1.551723396	-0.922024352
-3.419049539	-2.45193606	-3.306322251	2.529551308	0.529551308	2.529551308	-1.529551308	-1.529551308	-1.529551308
**WT-C**	**KO-C**	**OE-C**	**WT-2h**	**KO-2h**	**OE-2h**	**WT-12h**	**KO-12h**	**OE-12h**
0.424537813	0.695006056	0.821292048	0.61418421	0.542081572	0.68082815	-2.269414038	-1.857490882	-2.800083557
0.677481493	0.563472976	0.962369647	0.671169798	0.741266267	0.637645347	-2.037797116	-1.324687158	-2.438673078
0.759947575	0.28256686	0.80529602	0.699871174	0.60686612	0.668510161	-1.824141222	-1.696132544	-2.485906687
0.551198794	0.37099048	0.178415532	0.449099156	0.524203381	0.430602998	-2.329852733	-1.580506968	-3.196860537
**WT-C**	**KO-C**	**OE-C**	**WT-2h**	**KO-2h**	**OE-2h**	**WT-12h**	**KO-12h**	**OE-12h**
-0.05650172	-0.103420764	1.224147782	0.757733145	1.824073349	1.194189241	-0.996080934	-2.704842973	-1.139297125
-0.375232276	1.708653845	2.184115392	0.231676629	1.366064591	0.664861475	-3.218236624	-2.392860776	-0.169042256
0.136274314	0.95759186	1.74550641	1.077337468	2.164245004	1.290959143	-1.501391594	-3.346585458	-2.523937148
-0.135427645	0.26682525	1.756448161	1.068832425	2.05879998	1.148688818	-1.688022172	-3.64286514	-0.833279678
1.339628421	-0.317669254	1.147469988	-0.085276216	0.966334327	2.076693732	-2.778149724	-2.778149724	-0.029118449
0.113013551	-0.109556807	1.07362159	1.381748105	1.585834054	0.843345069	-1.476653859	-2.643369304	-0.767982399
1.486933154	0.859260352	1.607807543	-0.047470421	1.025662675	1.273131551	-3.40234568	-3.40234568	-0.099366504
0.209673173	0.558356344	1.469533315	0.988987711	1.653209192	1.480015617	-0.760490295	-3.460506474	-2.138778582
0.291940513	0.233615519	2.128994257	1.303937427	2.689598777	-0.006841812	-2.900844426	-2.919363229	-0.821037025
-0.290018609	0.805211983	2.277660077	1.269248822	2.447003376	1.224395047	-1.595079817	-4.170439923	-1.967980957
1.35274895	-0.761455399	1.82158545	1.104660461	1.812032277	1.609438048	-2.119228109	-3.718069687	-1.10171199
-0.313248728	0.416873728	1.194669241	1.092773	2.140818129	1.361155869	-1.686417939	-3.092640215	-1.113983085
**WT-C**	**KO-C**	**OE-C**	**WT-2h**	**KO-2h**	**OE-2h**	**WT-12h**	**KO-12h**	**OE-12h**
-0.464707287	0.621781528	2.453305391	0.616901509	2.579657326	0.976343533	-3.389188131	-5.080837806	-2.313256061
0.357187562	-0.142005544	3.059568176	0.084609383	1.033839756	0.695966716	-3.683907459	-3.524672797	-2.880585794
-0.507111135	0.201461683	1.751178701	0.278432593	1.104681005	0.064258904	-3.131562107	-4.86789678	-2.843432923
-0.720692	0.801826689	0.727846665	-0.158776914	1.87468308	1.221239632	-2.549598541	-4.083161889	-2.886633278
0.43434756	0.72811934	1.964107821	0.031991449	1.802176325	0.694497133	-3.048562967	-4.392975927	-3.213700733
-0.166909332	1.131634336	0.586785721	1.066252333	1.525479121	0.512561881	-2.782384734	-3.943755673	-3.070336347
-0.894887625	0.459487708	3.025425489	-0.084852096	2.019798599	0.77527634	-2.716074705	-3.815350557	-2.768824154
**WT-C**	**KO-C**	**OE-C**	**WT-2h**	**KO-2h**	**OE-2h**	**WT-12h**	**KO-12h**	**OE-12h**
-1.147404134	-1.147404134	-1.147404134	2.304795915	1.447144415	1.871408052	-1.878370988	-1.155360858	-1.147404134
-2.186118994	-2.186118994	-2.186118994	1.54951149	2.819954574	2.105926499	-2.051750607	-1.31822253	-1.34943634

AT3G09560.1	PAH1_ARATHPhosphatidate phosphatase PAH1 OS=Arabidopsis thaliana GN=PAH1 PE=1 SV=1
AT1G56170.2	NFYC2_ARATHNuclear transcription factor Y subunit C-2 OS=Arabidopsis thaliana GN=NFYC2 PE=2 SV=2
AT3G49590.1	AT13A_ARATHAutophagy-related 13a OS=Arabidopsis thaliana GN=ATG13A PE=1 SV=1
AT3G55500.1	EXP16_ARATHExpansin-A16 OS=Arabidopsis thaliana GN=EXPA16 PE=2 SV=1
AT4G35110.3	plant phospholipase [Medicago truncatula]
AT5G65890.1	ACR1_ARATHACT domain-containing ACR1 OS=Arabidopsis thaliana GN=ACR1 PE=2 SV=1
AT4G39340.1	EC14_ARATHEgg cell-secreted OS=Arabidopsis thaliana GN= PE=2 SV=1
AT1G65040.3	HRD1B_ARATHERAD-associated E3 ubiquitin- ligase HRD1B OS=Arabidopsis thaliana GN=HRD1B PE=2 SV=1
AT1G28520.2	VOZ1_ARATHTranscription factor VOZ1 OS=Arabidopsis thaliana GN=VOZ1 PE=1 SV=1
AT1G63010.2	SPXM1_ARATHSPX domain-containing membrane At1g63010 OS=Arabidopsis thaliana GN=At1g63010 PE=2 SV=1
AT2G40900.1	WTR15_ARATHWAT1-related At2g40900 OS=Arabidopsis thaliana GN=At2g40900 PE=2 SV=1
AT4G34000.3	AI5L6_ARATHABSCISIC ACID-INSENSITIVE 5 6 OS=Arabidopsis thaliana GN=ABF3 PE=1 SV=1
AT4G36640.2	RSC5_DICDIRandom slug 5 OS=Dictyostelium discoideum GN=rsc5 PE=2 SV=1
AT2G04570.1	GDL34_ARATHGDSL esterase lipase At2g04570 OS=Arabidopsis thaliana GN=At2g04570 PE=2 SV=1
AT4G39330.1	CADH9_ARATHProbable cinnamyl alcohol dehydrogenase 9 OS=Arabidopsis thaliana GN=CAD9 PE=2 SV=2
AT3G53830.1	UVR8_ARATHUltraviolet-B receptor UVR8 OS=Arabidopsis thaliana GN=UVR8 PE=1 SV=1
AT2G46830.2	CCA1_ARATH CCA1 OS=Arabidopsis thaliana GN=CCA1 PE=1 SV=1
**Cluster 8**	**Description**
AT5G12840.1	NFYA1_ARATHNuclear transcription factor Y subunit A-1 OS=Arabidopsis thaliana GN=NFYA1 PE=2 SV=1
AT3G54810.2	GATA8_ARATHGATA transcription factor 8 OS=Arabidopsis thaliana GN=GATA8 PE=2 SV=1
AT5G60250.1	DEAHC_ARATHATP-dependent RNA helicase chloroplastic OS=Arabidopsis thaliana GN=At5g10370 PE=3 SV=1
AT4G13340.1	LRX3_ARATHLeucine-rich repeat extensin 3 OS=Arabidopsis thaliana GN=LRX3 PE=1 SV=1
AT4G27970.1	SLAH2_ARATHS-type anion channel SLAH2 OS=Arabidopsis thaliana GN=SLAH2 PE=2 SV=1
AT1G51820.1	Y5182_ARATHProbable LRR receptor-like serine threonine- kinase At1g51820 OS=Arabidopsis thaliana GN=At1g51820 PE=2 SV=1
AT5G63770.2	DGK2_ARATHDiacylglycerol kinase 2 OS=Arabidopsis thaliana GN=DGK2 PE=1 SV=1
AT3G48580.1	XTH11_ARATHProbable xyloglucan endotransglucosylase hydrolase 11 OS=Arabidopsis thaliana GN=XTH11 PE=2 SV=2
AT4G38420.1	ASOL_TOBACL-ascorbate oxidase homolog OS=Nicotiana tabacum PE=2 SV=1
AT4G16563.1	NEP2_NEPGRAspartic ase nepenthesin-2 OS=Nepenthes gracilis GN=nep2 PE=1 SV=1
AT3G12520.2	SUT42_ARATHProbable sulfate transporter OS=Arabidopsis thaliana GN=SULTR4 2 PE=2 SV=2
AT5G03040.2	IQD1_ARATH IQ-DOMAIN 1 OS=Arabidopsis thaliana GN=IQD1 PE=1 SV=1
AT1G02080.1	CNOT1_DANRECCR4-NOT transcription complex subunit 1 OS=Danio rerio GN=cnot1 PE=2 SV=1
AT2G23130.2	AGP17_ARATHLysine-rich arabinogalactan 17 OS=Arabidopsis thaliana GN=AGP17 PE=2 SV=1
AT5G50915.1	BH137_ARATHTranscription factor bHLH137 OS=Arabidopsis thaliana GN=BHLH137 PE=2 SV=1
AT1G60890.2	PI5K8_ARATHPhosphatidylinositol 4-phosphate 5-kinase 8 OS=Arabidopsis thaliana GN=PIP5K8 PE=1 SV=1
AT3G14870.3	IRKI_ARATHIRK-interacting OS=Arabidopsis thaliana GN=IRKI PE=1 SV=1
AT4G31000.2	CB60F_ARATHCalmodulin-binding 60 F OS=Arabidopsis thaliana GN=CBP60F PE=2 SV=1
AT5G64060.1	NAC82_ARATHNAC domain-containing 82 OS=Arabidopsis thaliana GN=NAC082 PE=1 SV=1
**Cluster 9**	**Description**
AT3G12150.1	CD029_MOUSEUncharacterized protein C4orf29 homolog OS=Mus musculus PE=2 SV=1
AT1G56430.1	NAS4_ARATHProbable nicotianamine synthase 4 OS=Arabidopsis thaliana GN=NAS4 PE=2 SV=1
AT5G10150.1	UFC_ARATH UPSTREAM OF FLC OS=Arabidopsis thaliana GN=UFC PE=2 SV=1
AT5G02890.1	taxadien-5-alpha-ol O-acetyltransferase
AT4G01390.1	UBP13_ARATHUbiquitin carboxyl-terminal hydrolase 13 OS=Arabidopsis thaliana GN=UBP13 PE=1 SV=1
AT1G52190.1	PTR6_ARATH NRT1 PTR FAMILY OS=Arabidopsis thaliana GN= PE=1 SV=1
AT1G49130.2	COL8_ARATHZinc finger CONSTANS-LIKE 8 OS=Arabidopsis thaliana GN=COL8 PE=2 SV=2
AT1G07650.2	Y1765_ARATHProbable LRR receptor-like serine threonine- kinase At1g07650 OS=Arabidopsis thaliana GN=At1g07650 PE=1 SV=1
AT2G41090.1	CML10_ARATHCalmodulin 10 OS=Arabidopsis thaliana GN=CML10 PE=2 SV=1
AT3G29370.1	transcription factor UPBEAT1-like [Cucumis melo]
AT3G50740.1	U72E1_ARATHUDP-glycosyltransferase 72E1 OS=Arabidopsis thaliana GN=UGT72E1 PE=1 SV=1
AT5G22310.1	plant F24K9-26 [Medicago truncatula]
AT1G20190.1	EXP11_ARATHExpansin-A11 OS=Arabidopsis thaliana GN=EXPA11 PE=2 SV=1
AT1G73830.2	BEE3_ARATHTranscription factor BEE 3 OS=Arabidopsis thaliana GN=BEE3 PE=2 SV=1
AT2G37030.1	ARG7_VIGRRIndole-3-acetic acid-induced ARG7 OS=Vigna radiata radiata GN=ARG7 PE=2 SV=1
AT3G48970.1	HIP26_ARATHHeavy metal-associated isoprenylated plant 26 OS=Arabidopsis thaliana GN=HIPP26 PE=1 SV=1
AT1G64400.1	LACS3_ARATHLong chain acyl- synthetase 3 OS=Arabidopsis thaliana GN=LACS3 PE=2 SV=1
AT1G19350.5	BZR2_ARATH BRASSINAZOLE-RESISTANT 2 OS=Arabidopsis thaliana GN=BZR2 PE=1 SV=1
AT4G23300.1	CRK22_ARATHCysteine-rich receptor kinase 22 OS=Arabidopsis thaliana GN=CRK22 PE=2 SV=1
AT5G64190.1	PREDICTED: uncharacterized protein LOC103437659
AT3G58990.1	LEUD2_ARATH3-isopropylmalate dehydratase small subunit 2 OS=Arabidopsis thaliana GN=IPMI1 PE=1 SV=1
AT1G49210.1	ATL76_ARATHE3 ubiquitin- ligase ATL76 OS=Arabidopsis thaliana GN=ATL76 PE=1 SV=1
AT3G18773.1	ATL77_ARATHRING-H2 finger ATL77 OS=Arabidopsis thaliana GN=ATL77 PE=2 SV=1
AT2G32100.1	OFP16_ARATHTranscription repressor OFP16 OS=Arabidopsis thaliana GN=OFP16 PE=2 SV=1
AT1G16880.2	ACR11_ARATHACT domain-containing ACR11 OS=Arabidopsis thaliana GN=ACR11 PE=1 SV=1
AT1G54730.3	ERDL5_ARATHSugar transporter ERD6-like 5 OS=Arabidopsis thaliana GN=At1g54730 PE=2 SV=2
AT5G59780.3	MYB59_ARATHTranscription factor MYB59 OS=Arabidopsis thaliana GN=MYB59 PE=2 SV=2
AT3G62550.1	USPAL_ARATHUniversal stress A OS=Arabidopsis thaliana GN=At3g01520 PE=1 SV=2
AT5G41761.1	PREDICTED: uncharacterized protein LOC103454635
AT1G66940.2	Y1670_ARATHProbable receptor kinase At1g67000 OS=Arabidopsis thaliana GN=At1g67000 PE=2 SV=2
AT1G49200.1	ATL75_ARATHRING-H2 finger ATL75 OS=Arabidopsis thaliana GN=ATL75 PE=2 SV=1
AT5G02540.1	TIC32_PEAShort-chain dehydrogenase TIC chloroplastic OS=Pisum sativum GN=TIC32 PE=1 SV=1
AT1G49750.1	PLRX3_ARATHPollen-specific leucine-rich repeat extensin 3 OS=Arabidopsis thaliana GN=PEX3 PE=2 SV=1
AT5G38410.3	RBS3B_ARATHRibulose bisphosphate carboxylase small chain chloroplastic OS=Arabidopsis thaliana GN=RBCS-3B PE=2 SV=2
AT1G14700.2	PPA3_ARATHPurple acid phosphatase 3 OS=Arabidopsis thaliana GN=PAP3 PE=2 SV=1
AT1G06080.1	ADS1_ARATHDelta-9 acyl-lipid desaturase 1 OS=Arabidopsis thaliana GN=ADS1 PE=2 SV=1
AT1G26220.1	YCF52_PORPUUncharacterized N-acetyltransferase ycf52 OS=Porphyra purpurea GN=ycf52 PE=3 SV=1
AT5G23020.1	MAM3_ARATHMethylthioalkylmalate synthase chloroplastic OS=Arabidopsis thaliana GN=MAM3 PE=1 SV=1
AT3G53570.3	AFC1_ARATHSerine threonine- kinase AFC1 OS=Arabidopsis thaliana GN=AFC1 PE=2 SV=2
AT4G27710.1	C70B3_ARATHCytochrome P450 709B3 OS=Arabidopsis thaliana GN=CYP709B3 PE=2 SV=1
AT1G02360.1	CHI10_ORYSJChitinase 10 OS=Oryza sativa japonica GN=Cht10 PE=2 SV=1
AT3G26165.1	C71BJ_ARATHCytochrome P450 71B19 OS=Arabidopsis thaliana GN=CYP71B19 PE=2 SV=1
AT2G17780.1	MCAC2_ARATH MID1-COMPLEMENTING ACTIVITY 2 OS=Arabidopsis thaliana GN=MCA2 PE=2 SV=1
AT2G36050.1	OPF15_ARATHTranscription repressor OFP15 OS=Arabidopsis thaliana GN=OFP15 PE=1 SV=1
AT3G50470.1	HR3_ARATHRPW8 3 OS=Arabidopsis thaliana GN=HR3 PE=2 SV=1
AT3G08940.1	CB4B_ARATHChlorophyll a-b binding chloroplastic OS=Arabidopsis thaliana GN= PE=1 SV=1
AT1G11850.3	transmembrane
AT5G67060.1	HEC1_ARATHTranscription factor HEC1 OS=Arabidopsis thaliana GN=HEC1 PE=1 SV=1
AT1G19960.1	transcription factor
**Cluster 10**	**Description**
AT1G23020.1	FRO3_ARATHFerric reduction oxidase mitochondrial OS=Arabidopsis thaliana GN=FRO3 PE=2 SV=1
AT2G34430.1	CB21_SINALChlorophyll a-b binding chloroplastic OS=Sinapis alba GN=CAB1 PE=3 SV=1
AT3G54500.3	dentin sialophospho [Medicago truncatula]
AT3G54500.4	dentin sialophospho [Medicago truncatula]
AT3G54500.1	dentin sialophospho [Medicago truncatula]
**Cluster 11**	**Description**
AT1G34370.2	STOP1_ARATH SENSITIVE TO PROTON RHIZOTOXICITY 1 OS=Arabidopsis thaliana GN=STOP1 PE=2 SV=1
AT5G53620.1	kinesin-related 8 isoform X2
AT1G20560.1	AAE1_ARATHProbable acyl-activating enzyme peroxisomal OS=Arabidopsis thaliana GN=AAE1 PE=2 SV=1
**Cluster 12**	**Description**
AT1G55500.2	YTHD2_MOUSEYTH domain-containing family 2 OS=Mus musculus GN=Ythdf2 PE=1 SV=1
AT5G48880.1	FADA_ARATHacetyl-CoA acyltransferase GN=FADA PE=1 SV=2
AT1G50260.2	SYT3_ARATHSynaptotagmin-3 OS=Arabidopsis thaliana GN=SYT3 PE=2 SV=1
AT1G19640.3	JMT_ARATHJasmonate O-methyltransferase OS=Arabidopsis thaliana GN=JMT PE=1 SV=3
AT2G46370.2	JAR1_ARATHJasmonic acid-amido synthetase JAR1 OS=Arabidopsis thaliana GN=JAR1 PE=1 SV=2
AT2G39460.2	R23A1_ARATH60S ribosomal L23a-1 OS=Arabidopsis thaliana GN=RPL23AA PE=2 SV=2
AT1G72770.2	P2C16_ARATH phosphatase 2C 16 OS=Arabidopsis thaliana GN=HAB1 PE=1 SV=1
AT2G36590.1	PROT3_ARATHProline transporter 3 OS=Arabidopsis thaliana GN=PROT3 PE=1 SV=1
AT1G58520.1	CSCLA_ARATHCSC1 RXW8 OS=Arabidopsis thaliana GN=RXW8 PE=2 SV=1

-1.799009487	-1.739364199	-1.739364199	2.079895231	2.363209955	2.06551541	-1.376002038	-1.739364199	-0.86752055
-1.476938791	-1.699162958	-1.233215139	2.548516595	2.145859347	1.987196542	-1.98882225	-1.598528507	-0.849048392
-2.223643952	-1.914913566	-1.187604733	1.641867657	2.468302537	1.734940549	-2.039195053	-1.4780674	-0.68603844
-1.508455684	-1.811446871	-1.968744264	1.916014848	3.012365933	1.057969019	-1.744837202	-1.69324336	-0.646109139
-1.585627171	-1.016668199	-2.137020139	1.392675893	1.36950564	2.0457516	-1.44637956	-1.583477231	-1.205715295
-1.242438848	-1.661070186	-2.213447257	1.515452809	1.62671381	1.315499064	-1.589112368	-1.80224655	-1.038721269
-0.741209768	-1.047506628	-1.289581415	2.502042868	1.066260053	1.160661456	-1.734122909	-1.626430979	-1.271443195
-1.498764022	-1.498764022	-1.498764022	1.175922598	2.05852352	1.786638197	-2.15223256	-1.776839057	-1.149398745
-2.286997562	-1.506267525	-2.286997562	1.728250212	2.905354439	1.149963776	-1.867293994	-1.160581635	-1.731181407
-0.763564858	-1.463245749	-1.463245749	1.472968152	2.256156763	2.276494364	-1.463245749	-1.570271933	-1.463245749
-1.444422756	-1.556154333	-0.693225654	1.164427475	2.177484366	0.847695485	-1.68374979	-1.460499565	-1.272554357
-2.238595076	-2.076077065	-2.072810584	2.341172922	2.880525919	2.016602212	-2.219449469	-1.177677544	-0.892590251
-1.396752066	-1.396752066	-1.396752066	1.821254084	1.933949736	1.041806941	-2.104559292	-1.894556794	-1.396752066
-1.126186939	-1.433028335	-1.025344325	1.642470162	1.892224788	1.050967097	-2.20107602	-1.633001907	-1.433028335
-0.828562234	-1.788651512	-1.343900143	1.794991129	1.609207334	1.38347453	-1.52533189	-1.184056131	-0.852833444
-1.701641768	-1.624089538	-1.746570613	2.571760131	1.39865949	1.921150506	-2.221759503	-1.237263977	-1.360244727
-2.734456538	-2.177738689	-2.028001791	2.246698657	2.600987359	2.003096093	-2.377081775	-2.029205631	-1.316872497
**WT-C**	**KO-C**	**OE-C**	**WT-2h**	**KO-2h**	**OE-2h**	**WT-12h**	**KO-12h**	**OE-12h**
1.916740483	1.777562398	1.701976391	-2.200176285	-2.33505434	-2.33505434	1.526702566	0.882601802	1.564436693
1.804521775	1.829654749	1.894326433	-1.805193159	-2.215927956	-1.002956697	1.189709639	1.263164454	1.051551053
1.35615083	1.634388105	1.374898071	-1.529339948	-0.985106802	-1.463004325	1.214337286	1.162495214	1.560171997
1.069469414	1.802944249	1.180506623	-1.043868998	-1.589812109	-1.101490777	1.377297767	1.374693493	0.964732446
1.310153382	1.7877626	1.528006578	-1.568425822	-1.396734394	-0.715495294	1.441127811	0.705236105	1.27489548
1.578043641	0.935257454	1.629244825	-1.047139366	-1.474734103	-1.565835182	1.18359311	1.199955875	0.961525495
1.769123039	1.221533052	1.349142845	-1.157255986	-1.157255986	-0.769893445	0.94594589	1.019096841	1.245756137
1.213974854	0.919806416	1.046742381	-1.089793061	-1.598728723	-0.979507769	0.877668036	1.067118798	1.078441216
1.152045923	1.73283791	1.23990334	-1.6107116	-1.678676436	-0.839733622	0.921837677	1.54021192	1.333051532
1.089214794	1.583354223	1.668651566	-1.80623558	-1.591825193	-1.099092629	1.061310476	1.074399306	0.916426021
1.228810893	1.687828686	1.125897859	-1.5230277	-1.5230277	-1.5230277	1.025287612	1.5230277	0.748609748
1.843466046	0.899552557	1.111936957	-1.081203962	-1.081203962	-1.081203962	1.551064253	1.081203962	1.081203962
1.918558586	1.705970777	1.266450385	-0.75601851	-1.855084938	-0.880440512	0.810998617	1.658048091	1.552386314
1.521007207	1.629008829	1.376295203	-1.063887369	-1.703039045	-0.915311688	0.733301515	1.062054599	1.237270353
1.476104937	1.604692686	0.893121894	-1.380991734	-2.123200667	-1.215011116	0.956214021	1.322515175	1.588414845
1.491727501	1.37245561	1.585449333	-1.052266355	-1.387978265	-1.465780417	1.226967102	1.056688802	0.727366894
1.781934499	1.158855159	1.602820289	-0.975410731	-2.171703241	-1.151645589	1.305974087	1.004987796	1.323706334
1.355390329	1.376018925	1.484751539	-1.421980481	-1.302341806	-1.085694476	1.040499129	0.795426864	0.878370434
1.853753829	1.261575998	1.362706335	-1.551103074	-1.244816929	-1.444669491	1.210421019	0.906210093	0.781614125
**WT-C**	**KO-C**	**OE-C**	**WT-2h**	**KO-2h**	**OE-2h**	**WT-12h**	**KO-12h**	**OE-12h**
-0.233861611	-1.138141584	-1.242050389	-0.964959933	-0.989014519	-0.809382996	1.752766713	2.045237266	1.579407053
-0.792005186	-1.291385129	-0.576520985	-0.510670648	-0.454644276	-0.673772486	1.395271965	1.571243815	2.332482929
-0.612225288	-1.079052708	-1.055134081	-0.863945372	-0.995567722	-0.899899866	1.742576874	2.251139495	1.512108669
-0.401136277	-1.183131277	-0.254778069	-0.436145229	-0.776549045	-0.975618032	1.463640464	1.986589334	1.577128131
-0.969615354	-0.895109917	-0.574552554	-0.506507066	0.004179576	-0.712604736	1.621382935	1.472399212	1.790179642
-0.629772357	-1.236003474	-1.084714763	-0.735184596	-1.166294502	-0.545486803	1.114913833	2.555621505	1.726921157
-0.664383325	-0.754402973	-1.02663782	-0.36786408	-1.049538222	-0.207967584	1.146124341	2.221887454	1.702782207
-1.938807003	-0.57591636	-0.384955034	0.279664208	-0.449777939	-0.938807003	1.558166578	2.01072793	1.439704621
-0.975256325	-1.978810351	-0.535271466	-0.933122845	-0.536220969	-1.061772462	1.012960645	2.80503087	2.13191997
-0.691281836	-0.873049746	-0.761859887	-0.784831633	-1.303870462	-0.870242215	2.127351542	2.456457172	1.134954871
-0.9875116	-1.568291507	-0.14577802	-0.249947749	-0.506965545	-0.859340289	0.753457583	1.184605796	2.37977133
-0.479309905	-0.889010478	-0.767663749	-0.474599829	-1.504053302	-0.750481787	0.851835518	1.705737278	1.307546253
-1.155891924	-0.791210396	-0.493038046	-1.470491207	-0.900658231	-1.020669093	1.198684298	2.473403575	1.173794932
-0.93910796	-1.480127114	-0.605133475	-1.480127114	-1.043965274	-0.94208566	1.414593659	2.605425014	1.470527924
-0.478405697	-0.512519114	-0.789908813	-0.789908813	-0.789908813	-0.789908813	0.876393315	2.729507284	1.544659463
-0.556354174	-1.016627049	-1.142278151	-0.506291647	-0.76487672	-0.987824558	0.947558939	2.723244808	1.303448552
-0.855624329	-1.135847941	0.159480455	0.122754219	0.169065177	-0.403018686	0.810187635	1.443448713	1.309930027
-1.1488006	-1.1488006	-1.1488006	-0.387515327	-1.1488006	-0.324236388	1.150444057	2.031347261	2.125162799
-0.998404392	-1.031044985	-0.58851057	-0.301386367	-0.795786788	-0.421849229	1.038016694	1.701583869	1.397381768
-0.527603486	-0.253999838	-0.967368071	-0.522661535	-0.995457683	-0.550413368	1.125051716	1.80979979	1.882652475
-1.220212386	-1.251521153	-0.307810283	-0.44164477	-0.371175468	-1.094486325	0.949792643	1.794032367	2.057987683
-0.790647888	-0.766631007	-0.963170001	-0.766631007	-1.113729677	-0.759841841	1.794122394	2.458068464	1.908460562
-0.176799496	-0.799988033	-1.368595921	-0.733407951	-0.35506893	-1.078757193	1.426560662	2.08314876	1.84930911
-0.23134722	-0.843195272	-1.340996972	-1.057906956	-0.937919168	-0.686286417	1.130529448	2.732508343	1.234614214
-0.377951342	-0.67139507	-1.222422814	-0.46750597	-1.183498403	-0.164727511	1.191158183	1.892304614	1.004038315
-0.515428455	-1.508028105	-1.433584708	-0.023946375	-1.08581163	-0.801102272	2.180867596	1.790796732	1.875245173
-1.293525977	-1.293525977	0.180520622	0.349868476	-1.293525977	-0.935259772	0.790538288	1.648706999	2.204469523
-0.298432867	-0.52179796	-1.252737774	-0.978343061	-1.120121607	-0.978868739	1.582497715	2.548672359	1.841284335
-1.376284371	-0.77165024	-0.856965487	-0.788156329	-1.264285073	-0.463858627	1.210261769	1.754966621	2.555971736
-0.685212278	-1.439672252	-1.439672252	-0.861458086	-0.680090278	-0.582089296	0.854580885	2.353183101	2.480430456
-1.369892407	-1.977349546	0.164363498	-1.333163635	-0.371922943	-0.967036972	1.583016045	2.038248694	1.563403991
-0.657033012	-1.265700162	-0.757048581	-0.201364293	-0.994938535	-0.531416011	0.805026809	2.328295167	1.274178618
-0.394940596	-1.291499828	-0.463701516	-0.425881627	-0.581251572	-0.849960036	1.331081921	2.075098662	1.601054593
-1.07417348	-1.543932419	-0.478915013	0.35099179	-0.403872058	-0.634697883	1.353166304	2.652874192	1.778558569
-0.645498777	-0.645498777	-0.645498777	-0.645498777	-0.645498777	-0.645498777	1.645498777	2.366354628	2.152136812
-0.464289094	-1.406393428	-0.828172505	-1.012635471	-0.616438054	-0.889426158	1.282553756	2.217398552	1.717402403
-0.69082194	-1.051766404	-0.569983429	-0.35654707	-0.863039248	-0.969320862	1.27784077	1.558766595	1.192482813
-0.36191155	-1.027957979	-0.148987469	0.132921883	-1.084271028	-0.929925896	1.003926478	1.326732189	2.097326327
-1.191872247	-0.463298645	0.062117019	-0.223965877	-1.191872247	-0.609816176	1.187550641	1.64728114	1.858977675
-0.912016249	-1.387726687	-0.70168319	-0.264713337	-0.645246431	-0.698824165	1.504733239	1.932358493	1.173118328
-0.477427202	-1.273068704	-0.205014464	-0.696478117	-1.065529411	-0.74101961	0.894555831	1.623696652	2.340285025
-0.682209121	-0.816863141	-1.317436033	-1.147154379	-1.023383839	-0.926189097	1.933495865	2.322639932	1.657099814
-0.766385907	-1.015673556	-0.716153672	-0.568516249	-0.808877989	-0.776207621	1.608607661	1.726904515	1.316302818
-0.701620797	-0.767840483	-0.992694142	-0.456883506	-0.665538654	-0.70966425	1.130426511	1.864515871	1.160601625
-0.710487723	-0.885047675	-0.965920406	-1.018869286	-0.865518509	-1.016175177	0.877737564	2.087347515	2.496933698
-0.885755757	-1.291544157	-1.100648668	-0.608590863	-0.785644272	-1.092871318	1.606801077	2.437851145	1.503221086
-1.058345059	-1.058345059	0.034538906	-1.058345059	-0.790509667	-1.058345059	1.53620349	2.729923023	1.723224485
-0.586366854	-0.916252188	-1.081038466	-0.399364324	-0.897075631	-0.316352613	0.853289312	2.193074072	1.150086692
-0.727120246	-1.182348817	-0.787286017	-0.918112666	-0.835250146	-0.91211891	1.340962214	1.676427928	2.34484666
**WT-C**	**KO-C**	**OE-C**	**WT-2h**	**KO-2h**	**OE-2h**	**WT-12h**	**KO-12h**	**OE-12h**
-3.946413134	-3.978691169	-3.946413134	0.854590274	1.556712946	0.711512638	1.842507439	1.792732888	2.113461254
-4.794051736	-3.200935914	-4.023000154	1.578571399	2.075254662	1.654364113	1.936642942	3.015865022	1.757289666
-3.784659562	-2.676756098	-3.006502866	1.877182	1.643382043	2.033575889	1.361453481	1.181983889	1.370341224
-2.957262367	-2.742823728	-3.621745208	1.455754172	1.74887246	0.825238804	1.969096303	1.62788975	1.694979814
-3.537529585	-2.781382279	-3.536580235	1.428838308	1.773013155	0.82678159	2.21942576	1.991259795	1.61617349
**WT-C**	**KO-C**	**OE-C**	**WT-2h**	**KO-2h**	**OE-2h**	**WT-12h**	**KO-12h**	**OE-12h**
-1.757337788	-1.757337788	-1.416908819	1.728673916	1.31990521	1.149168037	1.335643207	0.748807115	1.757337788
-2.433874253	-2.433874253	-1.606579869	1.483175534	1.084660886	1.257324882	1.511733451	1.358148135	1.433874253
-1.488473471	-1.54775599	-1.560266616	1.194377335	1.147945566	1.185964682	1.004134692	0.637559207	1.078729645
**WT-C**	**KO-C**	**OE-C**	**WT-2h**	**KO-2h**	**OE-2h**	**WT-12h**	**KO-12h**	**OE-12h**
-1.19344507	-0.462167801	-0.711975912	1.788717874	2.125252043	1.879690225	-2.399585657	-1.337916646	-2.03685648
-1.52185246	-0.782215365	-0.112381565	1.955451683	2.871303663	1.950263039	-0.762480529	-1.135287084	-2.462801382
-1.316414951	-0.467749614	-0.59106378	1.95671764	1.863828797	1.332110302	-1.924727939	-0.266549753	-2.703777492
-2.867038895	-0.510323321	-0.868285889	2.435016667	3.486065426	2.748521645	-2.047425836	-1.724238295	-1.60740598
-2.548389279	-0.385849059	-0.466340065	2.067260632	2.021118941	2.391718402	-1.218220408	-0.914424694	-0.946874471
-1.454880145	-1.094868466	-0.795530241	2.953581506	3.511114694	2.932900171	-1.927925019	-1.11388686	-3.010505639
-1.293223734	-1.091598367	-0.776238272	1.930544645	2.610561673	1.781294505	-0.478689637	-1.068747463	-1.613903349
-1.816077168	-1.142903857	-1.392611984	3.091183674	1.937933528	3.259675457	-1.950107338	-0.715484687	-1.915543844
-1.933139441	-0.413847575	-0.398760889	2.070142569	2.527612765	2.00752449	-1.548109956	-0.523165811	-1.788256152

AT1G19650.2	SFH4_ARATHPhosphatidylinositol phosphatidylcholine transfer SFH4 OS=Arabidopsis thaliana GN=SFH4 PE=2 SV=1
AT3G46650.1	U76E6_ARATHUDP-glycosyltransferase 76E6 OS=Arabidopsis thaliana GN=UGT76E6 PE=2 SV=1
AT5G62090.1	SLK2_ARATHProbable transcriptional regulator SLK2 OS=Arabidopsis thaliana GN=SLK2 PE=1 SV=1
AT1G24070.1	CSLAA_ARATHProbable mannan synthase 10 OS=Arabidopsis thaliana GN=CSLA10 PE=2 SV=2
AT3G55580.1	UVR8_ARATHUltraviolet-B receptor UVR8 OS=Arabidopsis thaliana GN=UVR8 PE=1 SV=1
AT3G14810.1	MSL5_ARATHMechanosensitive ion channel 5 OS=Arabidopsis thaliana GN=MSL5 PE=2 SV=1
AT3G24460.1	SERC3_HUMANSerine incorporator 3 OS=Homo sapiens GN=SERINC3 PE=2 SV=2
AT2G46790.1	APRR9_ARATHTwo-component response regulator-like APRR9 OS=Arabidopsis thaliana GN=APRR9 PE=1 SV=2
AT5G54470.1	BBX32_ARATHB-box zinc finger 32 OS=Arabidopsis thaliana GN=BBX32 PE=1 SV=1
AT4G35300.3	MSSP2_ARATHMonosaccharide-sensing 2 OS=Arabidopsis thaliana GN=MSSP2 PE=1 SV=2
AT1G48100.1	PGLR4_ARATHPolygalacturonase At1g48100 OS=Arabidopsis thaliana GN=At1g48100 PE=2 SV=1
AT1G02205.2	CER1_ARATH ECERIFERUM 1 OS=Arabidopsis thaliana GN=CER1 PE=1 SV=1
AT1G45249.3	AI5L5_ARATHABSCISIC ACID-INSENSITIVE 5 5 OS=Arabidopsis thaliana GN=ABF2 PE=1 SV=1
AT2G17500.1	YB8B_YEASTUncharacterized transporter YBR287W OS=Saccharomyces cerevisiae (strain ATCC 204508 S288c) GN=YBR287W PE=1 SV=1
AT3G05380.2	ALY2_ARATH ALWAYS EARLY 2 OS=Arabidopsis thaliana GN=ALY2 PE=1 SV=1
AT5G24670.2	ADAT3_DANREProbable inactive tRNA-specific adenosine deaminase 3 OS=Danio rerio GN=adat3 PE=2 SV=2
AT1G78680.2	GGH2_ARATHGamma-glutamyl hydrolase 2 OS=Arabidopsis thaliana GN=GGH2 PE=1 SV=2
AT1G73390.3	endosomal targeting BRO1-like domain [Medicago truncatula]
AT5G08130.2	BIM1_ARATHTranscription factor BIM1 OS=Arabidopsis thaliana GN=BIM1 PE=1 SV=2
**Cluster 13**	**Description**
AT1G56060.1	CYSTM1 family A-like
AT3G29250.2	SDR4_ARATHShort-chain dehydrogenase reductase 4 OS=Arabidopsis thaliana GN=SDR4 PE=2 SV=1
AT5G57010.1	IQM5_ARATHIQ domain-containing IQM5 OS=Arabidopsis thaliana GN=IQM5 PE=2 SV=1
AT1G76210.1	DUF241 domain [Medicago truncatula]
AT3G29000.1	CML30_ARATHProbable calcium-binding CML30 OS=Arabidopsis thaliana GN=CML30 PE=2 SV=1
AT5G42800.1	DFRA_ARATHDihydroflavonol-4-reductase OS=Arabidopsis thaliana GN=DFRA PE=1 SV=2
AT2G43620.1	CHI62_ARATHEndochitinase At2g43620 OS=Arabidopsis thaliana GN=At2g43620 PE=3 SV=1
AT2G39200.1	MLO12_ARATHMLO 12 OS=Arabidopsis thaliana GN=MLO12 PE=2 SV=2
AT1G66400.1	CML23_ARATHProbable calcium-binding CML23 OS=Arabidopsis thaliana GN=CML23 PE=2 SV=1
AT1G63245.1	CLE14_ARATHCLAVATA3 ESR (CLE)-related 2 OS=Arabidopsis thaliana GN=CLE14 PE=2 SV=1
AT3G29250.1	SDR4_ARATHShort-chain dehydrogenase reductase 4 OS=Arabidopsis thaliana GN=SDR4 PE=2 SV=1
AT3G28340.1	GATLA_ARATHProbable galacturonosyltransferase-like 10 OS=Arabidopsis thaliana GN=GATL10 PE=2 SV=1
AT1G07160.1	P2C02_ARATHProbable phosphatase 2C 2 OS=Arabidopsis thaliana GN=At1g07160 PE=2 SV=1
AT2G23270.1	transmembrane [Medicago truncatula]
AT3G02240.1	RGF7_ARATHRoot meristem growth factor 7 OS=Arabidopsis thaliana GN=RGF7 PE=3 SV=1
**Cluster 14**	**Description**
AT4G33905.1	PX24D_DICDIPXMP2 4 family 4 OS=Dictyostelium discoideum GN=DDB_G0290631 PE=3 SV=1
AT2G35730.1	hypothetical protein MTR_4g101025 [Medicago truncatula]
AT2G02990.1	RNS1_ARATHRibonuclease 1 OS=Arabidopsis thaliana GN=RNS1 PE=1 SV=1
AT1G61800.1	GPT2_ARATHGlucose-6-phosphate phosphate translocator chloroplastic OS=Arabidopsis thaliana GN=GPT2 PE=2 SV=2
AT1G77120.1	ADH1_ARATHAlcohol dehydrogenase class-P OS=Arabidopsis thaliana GN=ADH1 PE=1 SV=2
AT4G21680.1	PTR47_ARATH NRT1 PTR FAMILY OS=Arabidopsis thaliana GN= PE=2 SV=2
AT4G24000.1	CSLG2_ARATHCellulose synthase G2 OS=Arabidopsis thaliana GN=CSLG2 PE=2 SV=1
AT2G33380.2	PXG3_ARATHProbable peroxygenase 3 OS=Arabidopsis thaliana GN=PXG3 PE=1 SV=1
AT5G05220.1	unnamed protein product
AT2G45570.1	C76C2_ARATHCytochrome P450 76C2 OS=Arabidopsis thaliana GN=CYP76C2 PE=2 SV=1
AT1G57590.1	PAE2_ARATHPectin acetylesterase 2 OS=Arabidopsis thaliana GN=PAE2 PE=2 SV=1
AT4G33467.1	unnamed protein product
AT1G70640.1	PB1 domain [Medicago truncatula]
**Cluster 15**	**Description**
AT1G54050.1	kDa class II heat shock OS=Arabidopsis thaliana GN= PE=2 SV=1
AT1G55110.1	IDD7_ARATH indeterminate-domain 7 OS=Arabidopsis thaliana GN=IDD7 PE=2 SV=1
AT2G29500.1	kDa class I heat shock OS=Arabidopsis thaliana GN= PE=1 SV=1
AT3G55770.3	WLI2B_ARATHLIM domain-containing WLIM2b OS=Arabidopsis thaliana GN=WLIM2B PE=1 SV=1
AT2G42980.1	CDR1_ARATHAspartic ase CDR1 OS=Arabidopsis thaliana GN=CDR1 PE=1 SV=1
AT1G19650.1	SFH4_ARATHPhosphatidylinositol phosphatidylcholine transfer SFH4 OS=Arabidopsis thaliana GN=SFH4 PE=2 SV=1
AT1G05170.2	B3GT2_ARATHProbable beta-1,3-galactosyltransferase 2 OS=Arabidopsis thaliana GN=B3GALT2 PE=2 SV=1
AT4G23100.3	GSH1_ARATHGlutamate--cysteine chloroplastic OS=Arabidopsis thaliana GN=GSH1 PE=1 SV=2
AT4G32250.2	KEG_ARATHE3 ubiquitin- ligase KEG OS=Arabidopsis thaliana GN=KEG PE=1 SV=2
AT1G73602.1	PEAM3_ARATHPhosphoethanolamine N-methyltransferase 3 OS=Arabidopsis thaliana GN=NMT3 PE=2 SV=2
AT4G00440.3	phosphatidylinositol N-acetyglucosaminlytransferase subunit P [Medicago truncatula]
AT5G48570.1	FKB65_ARATHPeptidyl-prolyl cis-trans isomerase FKBP65 OS=Arabidopsis thaliana GN=FKBP65 PE=1 SV=1
AT1G66540.1	C8D11_ARATHCytochrome P450 81D11 OS=Arabidopsis thaliana GN=CYP81D11 PE=2 SV=1
AT5G20370.1	uncharacterized serine-rich -like [Cucumis melo]
AT1G56170.1	NFYC2_ARATHNuclear transcription factor Y subunit C-2 OS=Arabidopsis thaliana GN=NFYC2 PE=2 SV=2
AT5G24780.2	VSP1_ARATHVegetative storage 1 OS=Arabidopsis thaliana GN=VSP1 PE=1 SV=2
AT4G12400.2	HSOP3_ARATHHsp70-Hsp90 organizing 3 OS=Arabidopsis thaliana GN=HOP3 PE=2 SV=1
AT1G74310.1	CLPB1_ARATHChaperone 1 OS=Arabidopsis thaliana GN=CLPB1 PE=1 SV=2
AT3G24500.1	MBF1C_ARATHMulti -bridging factor 1c OS=Arabidopsis thaliana GN=MBF1C PE=1 SV=1
AT4G12400.1	HSOP3_ARATHHsp70-Hsp90 organizing 3 OS=Arabidopsis thaliana GN=HOP3 PE=2 SV=1
AT5G20490.2	MYO17_ARATHMyosin-17 OS=Arabidopsis thaliana GN=XI-K PE=1 SV=2
AT2G30830.1	GSL_ARATHProbable 2-oxoacid dependent dioxygenase OS=Arabidopsis thaliana GN=GSL-OH PE=2 SV=1
AT1G79430.1	APL_ARATHMyb family transcription factor APL OS=Arabidopsis thaliana GN=APL PE=2 SV=2
AT1G76680.2	OPR1_ARATH12-oxophytodienoate reductase 1 OS=Arabidopsis thaliana GN=OPR1 PE=1 SV=2
AT1G19640.1	JMT_ARATHJasmonate O-methyltransferase OS=Arabidopsis thaliana GN=JMT PE=1 SV=3
AT5G59730.2	E70A1_ARATHExocyst complex component EXO70A1 OS=Arabidopsis thaliana GN=EXO70A1 PE=1 SV=1
AT3G51840.1	ACOX4_ARATHAcyl-coenzyme A oxidase peroxisomal OS=Arabidopsis thaliana GN=ACX4 PE=1 SV=1
AT1G60270.1	BGL05_ARATH beta-glucosidase 5 OS=Arabidopsis thaliana GN=BGLU5 PE=5 SV=2
AT1G33110.2	DTX21_ARATH DETOXIFICATION 21 OS=Arabidopsis thaliana GN=DTX21 PE=1 SV=1
AT1G55860.2	UPL1_ARATHE3 ubiquitin- ligase UPL1 OS=Arabidopsis thaliana GN=UPL1 PE=1 SV=3
AT1G75170.1	RSC5_DICDIRandom slug 5 OS=Dictyostelium discoideum GN=rsc5 PE=2 SV=1
AT3G24200.2	COQ6_XENTRUbiquinone biosynthesis monooxygenase mitochondrial OS=Xenopus tropicalis GN=coq6 PE=2 SV=1
AT3G28740.1	C8D11_ARATHCytochrome P450 81D11 OS=Arabidopsis thaliana GN=CYP81D11 PE=2 SV=1
AT2G36390.1	GLGB1_ARATH1,4-alpha-glucan-branching enzyme 2- chloroplastic amyloplastic OS=Arabidopsis thaliana GN= PE=2 SV=1
AT1G52510.2	DHMA2_MYCTUHaloalkane dehalogenase 2 OS=Mycobacterium tuberculosis (strain ATCC 25618 H37Rv) GN=dhmA2 PE=1 SV=1
AT1G76130.1	AMY2_ARATHProbable alpha-amylase 2 OS=Arabidopsis thaliana GN=AMY2 PE=2 SV=1
AT2G42200.1	SPL9_ARATHSquamosa promoter-binding 9 OS=Arabidopsis thaliana GN=SPL9 PE=2 SV=2
AT5G16980.1	P1_ARATHNADP-dependent alkenal double bond reductase P1 OS=Arabidopsis thaliana GN=P1 PE=1 SV=1
AT2G38170.3	CAX1_ARATHVacuolar cation proton exchanger 1 OS=Arabidopsis thaliana GN=CAX1 PE=1 SV=3
AT4G16760.1	ACOX1_ARATHPeroxisomal acyl-coenzyme A oxidase 1 OS=Arabidopsis thaliana GN=ACX1 PE=1 SV=1
AT4G27820.1	BGL09_ARATHBeta-glucosidase 9 OS=Arabidopsis thaliana GN=BGLU9 PE=2 SV=2
AT4G39140.2	C3HC4-type RING zinc finger [Medicago truncatula]
AT3G25585.4	AAPT2_ARATHCholine ethanolaminephosphotransferase 2 OS=Arabidopsis thaliana GN=AAPT2 PE=1 SV=1
AT3G16340.1	AB29G_ARATHABC transporter G family member 29 OS=Arabidopsis thaliana GN=ABCG29 PE=2 SV=2
AT3G51895.1	SUT31_ARATHSulfate transporter OS=Arabidopsis thaliana GN=SULTR3 1 PE=2 SV=1
AT4G35560.1	STXB5_MOUSESyntaxin-binding 5 OS=Mus musculus GN=Stxbp5 PE=1 SV=3
AT5G12030.1	kDa class II heat shock OS=Arabidopsis thaliana GN= PE=2 SV=1
**Cluster 16**	**Description**
AT1G13360.2	hypothetical protein AT1G13360
AT2G47890.1	COL13_ARATHZinc finger CONSTANS-LIKE 13 OS=Arabidopsis thaliana GN=COL13 PE=2 SV=1
AT5G19440.1	CCR1_ARATHCinnamoyl- reductase 1 OS=Arabidopsis thaliana GN=CCR1 PE=1 SV=1
AT5G01600.1	FRI1_ARATHFerritin- chloroplastic OS=Arabidopsis thaliana GN=FER1 PE=2 SV=1
AT5G22860.1	PCP_PONABLysosomal Pro-X carboxypeptidase OS=Pongo abelii GN=PRCP PE=2 SV=1
AT5G26340.1	STP13_ARATHSugar transport 13 OS=Arabidopsis thaliana GN=STP13 PE=1 SV=2
AT1G32880.1	IMPA1_ARATHImportin subunit alpha-1 OS=Arabidopsis thaliana GN=IMPA1 PE=1 SV=2
AT1G17745.1	SERA2_ARATHD-3-phosphoglycerate dehydrogenase chloroplastic OS=Arabidopsis thaliana GN=PGDH2 PE=1 SV=2
AT2G36800.1	U73C5_ARATHUDP-glycosyltransferase 73C5 OS=Arabidopsis thaliana GN=UGT73C5 PE=2 SV=1

-1.659049444	-0.406242016	-0.713975654	1.783449237	2.06994831	1.744106817	-1.351565086	-1.393100656	-0.88605554
-0.972298037	-1.296729034	-0.719695346	1.963134964	2.265615806	2.040827749	-1.36612237	-1.330717033	-1.236515092
-2.182613654	-0.846878021	-0.439674159	2.17225907	2.17391594	2.107997962	-0.998787836	-1.709640589	-1.308144536
-0.966349532	-1.016218781	-0.328476531	2.482383991	2.735634946	1.348717532	-2.46455072	-1.49098828	-0.957105687
-1.297700112	-0.571668894	-0.673996829	1.949493901	2.408864883	1.664441808	-1.280075346	-1.003135838	-1.196223574
-1.675395036	-0.226474806	-0.369580036	1.756231718	2.6963079	1.719787831	-1.189467041	-1.00288943	-1.708521101
-1.069446407	-0.386532412	-0.229183481	1.711437068	2.464293864	1.718438726	-1.46484816	-1.034348624	-1.709810573
-1.989949752	-0.372149397	-0.445751095	2.112419932	2.126950877	2.251740419	-0.415103278	-0.839323711	-2.428833994
-1.069277936	-0.603284658	-0.20428904	1.915781467	2.726118946	2.332275419	-1.665090956	-1.658195594	-1.774037649
-2.751826586	-0.425772343	-0.412404112	2.335084531	2.854674865	2.033148447	-0.956059638	-0.776563264	-2.751826586
-1.411248976	-1.165399365	-0.698072108	3.007693353	3.440704325	3.032627828	-2.240035279	-1.774869274	-2.191400504
-1.674172851	-1.235140021	-1.659192365	2.254309818	2.324446733	2.078937506	-1.100855705	-0.49828405	-1.486617166
-1.352929276	-0.458143569	-0.511018423	1.598779225	2.479920584	1.576719542	-1.196203772	-1.305648172	-1.321983545
-1.810384575	-0.477533453	-1.810384575	1.235407843	2.493103952	2.761595545	-0.781815423	-0.239526332	-1.810384575
-1.849276691	-0.370124191	-1.849276691	2.245466091	2.17477698	1.671522683	-1.849276691	-0.521589327	-0.392470542
-1.964692619	-0.75125949	-0.322677836	2.444989727	2.933995894	1.075297961	-1.564476444	-1.269616593	-0.581560599
-1.429637377	-0.113601938	-0.403461871	2.466420712	2.172628599	1.18878019	-1.971939855	-1.44082803	-0.46836043
-3.253226309	-0.801523356	-0.727522435	2.794921689	3.712097185	3.032882514	-2.804258648	-1.166893119	-2.668427891
-1.821630846	-0.958508285	-1.062118242	2.787866937	2.06099602	1.770554074	-1.829289497	-0.995978546	-1.006250729
**WT-C**	**KO-C**	**OE-C**	**WT-2h**	**KO-2h**	**OE-2h**	**WT-12h**	**KO-12h**	**OE-12h**
2.274303072	1.916173717	2.766418022	-0.53690064	-1.405403143	-0.352224109	-1.082281491	-1.877389457	-2.70269597
2.483699453	1.819922272	2.458210674	-0.556465865	-1.246462671	-0.535140483	-1.572971136	-2.585094695	-2.127767504
2.325487499	1.809298633	2.659037878	-0.570473536	-0.871748024	-0.836356549	-0.801086464	-1.356412731	-2.357746707
1.730165179	1.733470604	2.900757316	-1.239154796	-1.626621007	-0.749756806	-0.756610477	-1.826999805	-2.165250206
2.515141507	1.879837047	2.379047711	-1.335743749	-1.010920318	-0.21088508	-0.913760911	-1.585357639	-2.282641432
2.254683977	1.743830332	2.381505088	-0.174563245	-0.944545401	-0.910541552	-2.081435088	-1.24886587	-2.020068239
1.968293601	2.602161557	2.971077345	-0.72033767	-0.420617674	-1.023892944	-2.506798743	-2.030214855	-2.160329383
2.608772644	1.991518286	2.563905107	-0.966047966	0.183154519	-0.617107606	-1.873829075	-2.221370662	-2.331004753
2.639893816	1.927725483	2.252937515	-1.201426313	-1.38668962	-0.41412834	-1.141801085	-1.695827704	-1.745730816
2.253901569	1.990553421	2.874261362	0.172624199	-0.524309421	-0.814413951	-2.32588199	-2.32588199	-2.300853196
2.469241044	1.731125645	2.119641631	-0.899135974	-0.943322665	-1.000727783	-1.499671279	-1.867042344	-2.047904806
2.116351456	1.941756744	2.297126492	-0.884250009	-0.975125899	-0.494050212	-0.968137818	-1.806644391	-2.120554708
2.002367785	2.625919276	2.921619667	-1.061570298	-1.025391872	-0.608199948	-0.9265956	-1.665607933	-1.737458924
2.613674381	1.743136	2.467121349	-0.723616536	-1.440826835	-0.922795342	-1.440826835	-1.185023998	-2.110842185
1.657321491	2.079338698	2.396944125	-1.076787894	0.046067159	-1.155921042	-2.069961291	-2.564632903	-1.587631655
**WT-C**	**KO-C**	**OE-C**	**WT-2h**	**KO-2h**	**OE-2h**	**WT-12h**	**KO-12h**	**OE-12h**
0.501906525	1.331555062	2.163382804	1.61455558	2.526257914	1.586043938	-3.645413264	-3.755695319	-3.32259324
1.581759414	1.893482466	2.526933018	0.937623533	2.21090347	1.242454719	-3.53528355	-4.322953149	-3.534919921
1.976210505	1.543160854	2.283934636	1.061725379	1.628814842	1.73399092	-3.584086973	-4.221799388	-3.421950774
1.346273463	1.758452708	2.364653495	0.959328796	1.562203883	1.635074894	-4.947780269	-4.069364995	-2.608841976
1.920998711	2.265945002	2.779184095	0.956362715	1.951872784	1.062334905	-3.652436788	-4.04524434	-3.872217996
1.697228916	1.871652982	2.635616766	0.984679101	1.570824451	1.447459432	-2.901274996	-4.431059419	-3.188916042
1.266914303	2.105200131	2.731144975	1.017080656	2.587698364	1.338422297	-4.128291189	-4.126085233	-3.792084305
1.222921013	2.187208731	2.962239239	1.157168254	2.548648063	2.064157915	-4.385089796	-4.385089796	-3.372163622
1.110675185	2.004936264	2.568664696	0.814683646	2.354427731	1.673147255	-3.248412419	-4.152808495	-3.768777053
0.909646901	1.609818004	2.397307433	0.954429877	2.706522855	1.700412059	-2.948383692	-3.95675051	-3.873002926
0.858980685	1.738975906	2.58120661	1.00880922	2.177591395	1.603184919	-3.960398493	-4.152627269	-3.855722973
0.82820116	1.731323041	2.327973292	0.896980377	2.622474339	1.7803579	-3.29269992	-4.594872719	-3.29973747
1.219097786	1.78903104	2.576247637	1.827873752	3.092495346	2.338446613	-4.46191786	-3.830305266	-4.550969048
**WT-C**	**KO-C**	**OE-C**	**WT-2h**	**KO-2h**	**OE-2h**	**WT-12h**	**KO-12h**	**OE-12h**
-0.227298543	-0.217209816	-0.294501733	2.057581527	1.537942153	1.8304353	-1.235984675	-1.56157985	-1.859833511
-0.825585269	-0.15442497	-0.231479663	1.787686057	2.091215738	1.269439641	-1.293047068	-1.17237831	-1.471426156
-1.338555447	-0.865945894	-1.384243574	1.597914758	2.415558089	1.670663735	-1.788093202	-0.559963597	-2.156337551
-0.263065513	-0.794682075	-0.512093061	0.812652514	1.416769813	1.266851078	-0.668587416	-2.081097988	-0.766111502
-0.654905271	-0.497714079	-0.185577905	1.611643948	1.718866532	1.301712207	-0.814009124	-0.952873467	-1.527142842
-0.408610186	-0.512305762	-0.153469816	1.456315823	1.497857096	1.419741679	-1.139072387	-0.791916743	-1.368539704
-1.330136648	-0.246015474	-0.397133212	1.008492904	2.37889207	1.524205196	-1.519022258	-0.560442185	-1.35087134
-0.16537655	-0.300707226	-0.513319426	1.930307374	1.946087282	1.649738856	-1.121067246	-1.617560183	-1.808102881
-1.457576043	-0.718620743	0.020365586	1.687959597	1.817675577	1.210958202	-1.617467867	-1.28544658	-1.095089216
-1.512863943	-1.326894137	-1.188629381	2.249803783	1.815110782	1.103258462	-0.362954275	-1.124398846	-1.306220719
-1.276285849	-0.410067232	0.009894943	0.773849206	2.213033981	1.464613315	-1.251816853	-1.036373328	-1.306982646
-0.172644368	-0.830249589	-1.094835379	2.201123757	1.949072906	2.417978746	-1.683779198	-2.337053224	-2.00123595
-0.527784354	-0.230407976	-0.438082178	1.487689883	1.943738713	1.468121573	-1.27148366	-0.932107015	-1.499684985
-0.752650756	-0.923231881	-1.127736946	1.787743604	2.360826333	1.035129237	-1.68318036	-1.869058319	-2.179606186
-0.605550188	-0.716256558	-0.223195842	2.028404694	2.084717078	1.627550019	-1.277536128	-1.236392771	-1.681740304
-0.576860414	-1.401966164	-1.266606852	2.517268825	2.45410939	1.269943443	-1.266606852	-1.266606852	-1.266606852
-0.345023125	-0.308649572	-0.653582888	2.080208933	1.718075812	1.833969278	-1.18780367	-1.718850848	-1.035643064
-0.153739311	-0.708050969	-0.576914749	2.114594121	2.343978319	2.236893675	-1.481105797	-2.322301873	-2.176933976
-0.410195673	-0.281552544	-0.159302232	2.026181359	1.710559048	2.072159548	-1.742541606	-2.230312751	-2.368491582
-0.044123515	-0.431404095	-1.383045894	2.433243929	1.507540709	1.932305604	-1.166462079	-1.636271278	-2.074591571
-0.628494482	-0.489098005	-1.607939495	1.951481547	1.896112154	1.846816689	-1.57201992	-1.007607648	-2.624435814
-0.154573651	-0.234607715	-1.036627172	2.592909815	2.11236521	2.858561751	-1.427124752	-1.654865828	-1.525253089
-0.475749963	-0.566880911	0.023304593	1.294847828	2.035171438	1.266249344	-1.231961248	-1.038747396	-1.929802686
-0.975947083	-0.9203261	-0.661720602	1.875795158	2.320800852	0.671564418	-0.982400871	-1.075787477	-2.044524661
-0.784924903	-0.251301484	0.199023187	1.752884123	1.579866723	2.43643529	-2.285745483	-2.07934801	-1.932909803
-0.848097453	-0.852903589	-1.094201463	1.238363268	2.045104303	0.943180759	-1.094201463	-0.948850077	-1.094201463
-1.265103431	-0.182331675	-1.482606203	2.466788656	1.751512472	1.958279571	-1.502946651	-1.403313436	-1.488388555
-0.349569026	-0.301965414	0.125522761	2.157967249	1.872934769	1.281216531	-1.549428796	-1.255770104	-1.980907969
-0.317485491	-0.272999717	-0.977845122	2.131024372	2.403037269	1.631354402	-2.144290696	-0.977980415	-2.109785582
-0.583885826	-0.291768481	-1.113731855	2.126781724	1.400225579	1.485882299	-0.347941515	-2.107949502	-1.151149384
-0.949548716	-0.921185588	-0.324895877	1.598412164	2.560295581	1.911381672	-2.567090808	-1.636208117	-0.684464458
-0.157775842	-1.167520851	-0.136850003	1.356961264	1.917067627	1.023347366	-0.608858879	-1.780706193	-1.780706193
-0.754443028	-0.129218074	-0.701362652	1.263788007	2.44088558	1.724624832	-1.643605345	-1.246228461	-2.721763064
-0.436490725	-0.220238819	-0.06325672	1.50901012	1.637231646	0.95868454	-1.706895526	-0.943124821	-1.65553713
-0.438092931	-0.518962634	-0.193009622	2.209740454	1.859799736	1.549760454	-1.725048803	-1.631939399	-1.112247255
-0.414587981	-0.139663375	0.303968862	1.430883954	1.848474209	1.040449163	-1.922652888	-1.120660916	-1.744271637
-0.404537513	-0.253260791	0.315873181	1.874533751	1.873092276	0.897458151	-1.457124475	-1.287819481	-1.558215099
-0.654021551	-0.113666664	-0.521372146	1.939143113	2.875484886	1.345911921	-1.900570236	-1.055054959	-2.776416131
-0.652936326	-0.465973467	-0.94323433	2.137586186	1.959901188	1.51630937	-1.268885312	-1.612119314	-1.602594928
-0.14334032	-1.159046018	-1.689752163	1.608612892	2.04273413	1.759412994	-0.861120581	-0.472521447	-1.689752163
-0.083999763	-0.113120529	0.361424442	1.483969634	1.755278097	1.319064964	-1.548883634	-1.295376332	-1.878356876
-1.157703444	-1.660533596	-1.157703444	1.571305426	1.430381515	1.26156576	-1.157703444	-1.157703444	-0.292972522
-0.293354637	-0.616291019	0.194688139	1.650993656	1.390596496	1.324397799	-1.446230768	-1.386917955	-1.435789021
-0.640832371	-0.879767594	-1.371501904	1.447558087	1.555048492	1.329915503	-1.364786477	-0.865179154	-0.96998977
-0.629698292	-0.853254401	0.241671472	1.915712284	1.936152776	1.271190627	-1.584231046	-0.979990042	-2.356133219
-0.617048674	-0.310520224	-0.393670469	1.704419157	1.435797296	1.317987774	-1.147819075	-0.517973264	-1.471172523
-0.472885826	-0.320274758	-0.588751883	1.806318088	1.379833949	1.807370832	-1.311087923	-1.480890277	-2.405953369
**WT-C**	**KO-C**	**OE-C**	**WT-2h**	**KO-2h**	**OE-2h**	**WT-12h**	**KO-12h**	**OE-12h**
1.095127526	0.806501406	3.059142069	1.090765501	1.714235872	1.098260037	-2.95467747	-2.95467747	-2.95467747
1.033310893	0.662007895	1.739258193	1.05191201	1.72277988	0.714361064	-2.286347489	-2.112850253	-2.524432194
1.656391426	1.193462109	2.426419171	0.632059681	1.040577278	0.923153176	-2.569655923	-2.456166274	-2.846240644
0.702202712	0.814977421	2.950367492	0.88825865	1.06813407	0.505645472	-1.715854586	-2.378826958	-2.834904273
0.666164584	1.145655844	2.228506856	0.987157311	2.585146554	1.449773027	-3.502907509	-2.278630729	-3.280865937
0.975785698	0.915115503	2.827175053	0.716165705	1.173853533	1.037005669	-2.621001244	-2.825669083	-2.198430833
2.117840587	1.941508912	1.854548976	1.783344994	0.766231092	0.57623771	-3.349784746	-3.184536232	-2.505391292
0.88019227	0.895810939	2.80489995	0.458150104	1.262870833	0.855028787	-2.94728868	-2.597575975	-1.612088228
1.755034491	0.766629666	1.288210717	0.942555458	1.126151628	0.7090173	-2.534223112	-1.739029258	-2.314346891

AT5G16960.1	P2_ARATHNADP-dependent alkenal double bond reductase P2 OS=Arabidopsis thaliana GN=P2 PE=2 SV=2
AT1G78000.1	SUT12_ARATHSulfate transporter OS=Arabidopsis thaliana GN=SULTR1 2 PE=1 SV=1
AT4G01070.1	U72B1_ARATHUDP-glycosyltransferase 72B1 OS=Arabidopsis thaliana GN=UGT72B1 PE=1 SV=1
AT3G53232.1	rotundifolia [Medicago truncatula]
AT1G66390.1	MYB90_ARATHTranscription factor MYB90 OS=Arabidopsis thaliana GN=MYB90 PE=1 SV=1
AT3G01040.1	GAUTD_ARATHProbable galacturonosyltransferase 13 OS=Arabidopsis thaliana GN=GAUT13 PE=2 SV=1
AT1G15415.1	late embryogenesis abundant 1-like
AT3G56090.1	FRI3_ARATHFerritin- chloroplastic OS=Arabidopsis thaliana GN=FER3 PE=2 SV=1
AT2G25620.1	P2C22_ARATHProbable phosphatase 2C 22 OS=Arabidopsis thaliana GN=At2g25620 PE=2 SV=1
AT2G37770.2	AKRC9_ARATHAldo-keto reductase family 4 member C9 OS=Arabidopsis thaliana GN=AKR4C9 PE=1 SV=1
AT1G09240.1	NAS3_ARATHNicotianamine synthase 3 OS=Arabidopsis thaliana GN=NAS3 PE=2 SV=1
AT1G08650.1	PPCK1_ARATHPhosphoenolpyruvate carboxylase kinase 1 OS=Arabidopsis thaliana GN=PPCK1 PE=1 SV=1
AT1G13930.1	nodulin-related 1
AT4G34590.1	BZP53_ARAT transcription factor 53 OS=Arabidopsis thaliana GN=BZIP53 PE=1 SV=1
AT1G76690.1	OPR2_ARATH12-oxophytodienoate reductase 2 OS=Arabidopsis thaliana GN=OPR2 PE=1 SV=2
AT4G21990.1	APR3_ARATH5 -adenylylsulfate reductase chloroplastic OS=Arabidopsis thaliana GN=APR3 PE=2 SV=2
AT5G39050.1	PMAT1_ARATHPhenolic glucoside malonyltransferase 1 OS=Arabidopsis thaliana GN=PMAT1 PE=1 SV=1
AT4G34588.1	BZP53_ARAT transcription factor 53 OS=Arabidopsis thaliana GN=BZIP53 PE=1 SV=1
AT4G11590.1	FB231_ARATHF-box At4g11590 OS=Arabidopsis thaliana GN=At4g11590 PE=1 SV=1
AT5G24860.1	FPF1_ARATHFlowering-promoting factor 1 OS=Arabidopsis thaliana GN=FPF1 PE=2 SV=1
AT1G02850.1	BGL11_ARATHBeta-glucosidase 11 OS=Arabidopsis thaliana GN=BGLU11 PE=2 SV=2
AT5G49480.1	CALM_RENRECalmodulin OS=Renilla reniformis PE=1 SV=2
AT2G37770.1	AKRC9_ARATHAldo-keto reductase family 4 member C9 OS=Arabidopsis thaliana GN=AKR4C9 PE=1 SV=1
AT1G70790.1	CAR9_ARATH C2-DOMAIN ABA-RELATED 9 OS=Arabidopsis thaliana GN=CAR9 PE=2 SV=1
AT5G05410.2	DRE2A_ARATHDehydration-responsive element-binding 2A OS=Arabidopsis thaliana GN=DREB2A PE=1 SV=1
AT5G17390.1	adenine nucleotide alpha hydrolase superfamily [Medicago truncatula]
AT4G33465.1	DF233_ARATH defensin 233 OS=Arabidopsis thaliana GN=SCRL22 PE=2 SV=2
AT3G44860.1	MT799_ARATHProbable S-adenosylmethionine-dependent methyltransferase At5g37990 OS=Arabidopsis thaliana GN=At5g37990 PE=3 SV=2
**Cluster 17**	**Description**
AT2G44380.1	NRX1_ARATHProbable nucleoredoxin 1 OS=Arabidopsis thaliana GN=At1g60420 PE=1 SV=1
AT2G44370.1	probable nucleoredoxin 1-1
AT2G36690.1	DIOX2_PAPSOProbable 2-oxoglutarate Fe(II)-dependent dioxygenase OS=Papaver somniferum GN=DIOX2 PE=2 SV=1
AT1G19610.1	DEF19_ARATHDefensin 19 OS=Arabidopsis thaliana GN= PE=3 SV=2
AT1G76930.2	EXTN1_ARATHExtensin-1 OS=Arabidopsis thaliana GN=EXT1 PE=2 SV=2
AT5G64100.1	PER69_ARATHPeroxidase 69 OS=Arabidopsis thaliana GN=PER69 PE=1 SV=1
AT1G30730.1	FOX4_ARATHFlavin-dependent oxidoreductase FOX4 OS=Arabidopsis thaliana GN=FOX4 PE=2 SV=1
**Cluster 18**	**Description**
AT2G22980.1	SCP13_ARATHSerine carboxypeptidase-like 13 OS=Arabidopsis thaliana GN=SCPL13 PE=2 SV=2
AT3G56970.1	ORG2_ARATHTranscription factor ORG2 OS=Arabidopsis thaliana GN=ORG2 PE=1 SV=1
AT3G13060.1	YTHD3_PONABYTH domain-containing family 3 OS=Pongo abelii GN=YTHDF3 PE=2 SV=1
AT2G41240.2	BH100_ARATHTranscription factor bHLH100 OS=Arabidopsis thaliana GN=BHLH100 PE=2 SV=1
AT5G04150.1	BH101_ARATHTranscription factor bHLH101 OS=Arabidopsis thaliana GN=BHLH101 PE=2 SV=1
AT5G05250.1	PREDICTED: uncharacterized protein LOC103432918
AT5G56870.1	BGAL4_ARATHBeta-galactosidase 4 OS=Arabidopsis thaliana GN=BGAL4 PE=1 SV=1
AT5G49730.1	FRO6_ARATHFerric reduction oxidase 6 OS=Arabidopsis thaliana GN=FRO6 PE=2 SV=1
AT3G56980.1	ORG3_ARATHTranscription factor ORG3 OS=Arabidopsis thaliana GN=ORG3 PE=1 SV=1
AT3G47340.1	ASNS1_ARATHAsparagine synthetase
AT4G27450.1	TSJT1_TOBACStem-specific TSJT1 OS=Nicotiana tabacum GN=TSJT1 PE=2 SV=1
**Cluster 19**	**Description**
AT5G58330.3	MDHP_PEAMalate dehydrogenase
AT4G29950.2	TBC5A_DICDITBC1 domain family member 5 homolog A OS=Dictyostelium discoideum GN=tbc1d5A PE=1 SV=1
AT5G42520.3	BPC6_ARATH BASIC PENTACYSTEINE6 OS=Arabidopsis thaliana GN=BPC6 PE=1 SV=1
AT2G18190.1	AATP2_ARATHAAA-ATPase At2g18190 OS=Arabidopsis thaliana GN=At2g18190 PE=2 SV=1
AT1G12760.2	RING1_ARATHE3 ubiquitin- ligase At1g12760 OS=Arabidopsis thaliana GN=At1g12760 PE=1 SV=1
AT5G08130.1	BIM1_ARATHTranscription factor BIM1 OS=Arabidopsis thaliana GN=BIM1 PE=1 SV=2
AT3G13810.3	IDD11_ARATH indeterminate-domain 11 OS=Arabidopsis thaliana GN=IDD11 PE=2 SV=1
AT4G35785.1	SR45A_ARATHSerine arginine-rich splicing factor SR45a OS=Arabidopsis thaliana GN=SR45A PE=1 SV=1
AT4G03292.1	ribonuclease H At1g65750
AT5G48880.3	THIK5_ARATH3-ketoacyl- thiolase peroxisomal OS=Arabidopsis thaliana GN=KAT5 PE=2 SV=2
AT4G35920.3	MCAC1_ARATH MID1-COMPLEMENTING ACTIVITY 1 OS=Arabidopsis thaliana GN=MCA1 PE=1 SV=1
AT3G03790.1	IBTK_XENLAInhibitor of Bruton tyrosine kinase OS=Xenopus laevis GN=ibtk PE=2 SV=1
AT4G31240.2	NRX3_ARATHProbable nucleoredoxin 3 OS=Arabidopsis thaliana GN=At4g31240 PE=2 SV=1
AT1G74920.2	BADH1_ARATHBetaine aldehyde dehydrogenase chloroplastic OS=Arabidopsis thaliana GN=ALDH10A8 PE=2 SV=1
AT1G17990.2	ORL2B_ARATH 12-oxophytodienoate reductase 2B OS=Arabidopsis thaliana GN=At1g18020 PE=3 SV=1
AT1G14570.4	PUX7_ARATHPlant UBX domain-containing 7 OS=Arabidopsis thaliana GN=PUX7 PE=1 SV=1
AT4G08980.3	FBW2_ARATHF-box FBW2 OS=Arabidopsis thaliana GN=FBW2 PE=1 SV=1
AT1G02145.1	ALG12_ARATHDol-P-Man:Man(7) c(2)-PP-Dol alpha-1,6-mannosyltransferase OS=Arabidopsis thaliana GN=ALG12 PE=1 SV=1
AT5G09300.2	ODBA2_ARATH2-oxoisovalerate dehydrogenase subunit alpha mitochondrial OS=Arabidopsis thaliana GN=At5g09300 PE=1 SV=1
AT5G11860.4	CTSL2_DICDICTD small phosphatase 2 OS=Dictyostelium discoideum GN=ctdspl2 PE=3 SV=1
AT3G44750.2	HDT1_ARATHHistone deacetylase HDT1 OS=Arabidopsis thaliana GN=HDT1 PE=1 SV=1
AT1G25150.1	FBK14_ARATHF-box kelch-repeat At1g25211 OS=Arabidopsis thaliana GN=At1g25211 PE=4 SV=1
AT2G31060.1	TYPA_HELPYGTP-binding homolog OS=Helicobacter pylori (strain ATCC 700392 26695) GN=typA PE=3 SV=1
AT4G00500.2	calmodulin-binding heat-shock [Medicago truncatula]
AT1G77240.1	AAE4_ARATHProbable acyl-activating enzyme 4 OS=Arabidopsis thaliana GN=AEE4 PE=2 SV=1
AT2G39310.3	JAL22_ARATHJacalin-related lectin 22 OS=Arabidopsis thaliana GN=JAL22 PE=1 SV=1
AT2G39310.2	JAL22_ARATHJacalin-related lectin 22 OS=Arabidopsis thaliana GN=JAL22 PE=1 SV=1
AT3G01540.3	RH14_ARATHDEAD-box ATP-dependent RNA helicase 14 OS=Arabidopsis thaliana GN=RH14 PE=1 SV=2
AT3G01790.1	RL13_CHLPB50S ribosomal L13 OS=Chlorobium phaeobacteroides (strain BS1) GN=rplM PE=3 SV=1
AT2G28710.1	ZAT12_ARATHZinc finger ZAT12 OS=Arabidopsis thaliana GN=ZAT12 PE=2 SV=1
AT1G59890.3	SNL5_ARATHPaired amphipathic helix Sin3-like 5 OS=Arabidopsis thaliana GN=SNL5 PE=2 SV=3
AT2G15000.3	At4g34265 [Brassica napus]
AT1G70440.1	SRO3_ARATHProbable inactive poly
AT1G67610.1	FB262_ARATHF-box At5g25290 OS=Arabidopsis thaliana GN=At5g25290 PE=2 SV=1
AT2G18180.1	SFH10_ARATHPhosphatidylinositol phosphatidylcholine transfer SFH10 OS=Arabidopsis thaliana GN=SFH10 PE=3 SV=1
AT1G55870.2	PARN_ARATHPoly(A)-specific ribonuclease PARN OS=Arabidopsis thaliana GN=PARN PE=1 SV=2
AT5G65310.2	ATHB5_ARATHHomeobox-leucine zipper ATHB-5 OS=Arabidopsis thaliana GN=ATHB-5 PE=1 SV=1
AT1G60750.1	ALKR6_ARATHProbable aldo-keto reductase 6 OS=Arabidopsis thaliana GN=At1g60750 PE=3 SV=1
AT1G05180.2	ULA1_ARATHNEDD8-activating enzyme E1 regulatory subunit OS=Arabidopsis thaliana GN=AXR1 PE=1 SV=1
AT3G63510.2	DUSA_VIBV -dihydrouridine(20 20a) synthase OS=Vibrio vulnificus (strain CMCP6) GN=dusA PE=3 SV=2
AT1G50030.2	TOR_ARATHSerine threonine- kinase TOR OS=Arabidopsis thaliana GN=TOR PE=1 SV=1
AT1G80770.2	NOG1_HUMANNucleolar GTP-binding 1 OS=Homo sapiens GN=GTPBP4 PE=1 SV=3
AT5G62570.2	CB60A_ARATHCalmodulin-binding 60 A OS=Arabidopsis thaliana GN=CBP60A PE=2 SV=1
AT5G58700.2	PLCD4_ARATHPhosphoinositide phospholipase C 4 OS=Arabidopsis thaliana GN=PLC4 PE=2 SV=2
**Cluster 20**	**Description**
AT1G67360.2	Y1736_ARATHREF SRPP At1g67360 OS=Arabidopsis thaliana GN=At1g67360 PE=2 SV=1
AT2G33380.1	PXG3_ARATHProbable peroxygenase 3 OS=Arabidopsis thaliana GN=PXG3 PE=1 SV=1
AT3G09920.3	PI5K9_ARATHPhosphatidylinositol 4-phosphate 5-kinase 9 OS=Arabidopsis thaliana GN=PIP5K9 PE=1 SV=2
AT4G25480.1	DRE1A_ARATHDehydration-responsive element-binding 1A OS=Arabidopsis thaliana GN=DREB1A PE=1 SV=2
AT5G51760.1	P2C75_ARATHProbable phosphatase 2C 75 OS=Arabidopsis thaliana GN=AHG1 PE=2 SV=1
AT3G10912.1	ATL72_ARATHRING-H2 finger ATL72 OS=Arabidopsis thaliana GN=ATL72 PE=2 SV=1
AT4G05020.1	NDB2_ARATHExternal alternative NAD(P)H-ubiquinone oxidoreductase mitochondrial OS=Arabidopsis thaliana GN=NDB2 PE=1 SV=1
AT1G52855.1	PREDICTED: uncharacterized protein LOC103495535
AT3G05630.1	PLDZ2_ARATHPhospholipase D zeta 2 OS=Arabidopsis thaliana GN=PLPZETA2 PE=1 SV=2
AT4G25433.1	peptidoglycan-binding domain [Medicago truncatula]
AT4G17690.1	PER41_ARATHPeroxidase 41 OS=Arabidopsis thaliana GN=PER41 PE=3 SV=1
AT4G02360.1	DUF538 family [Medicago truncatula]
AT5G54165.1	unnamed protein product

1.923250003	1.404586826	0.556569406	1.464030165	1.839613801	0.468714191	-1.809600888	-2.822028376	-3.025135127
1.509529918	1.251165457	1.54617347	1.349831608	1.042497175	0.763401589	-3.354210347	-3.354210347	-3.754178524
0.979487737	0.66757661	1.956691984	1.445363933	1.916889109	1.377687691	-3.149679439	-2.474846784	-2.719170843
0.964891671	0.096586422	2.947254603	1.443621898	1.823642861	1.65029712	-3.556424845	-2.45918455	-2.910685182
1.261517249	0.772993895	2.402977713	0.869650471	1.187585892	0.824558803	-3.411384398	-1.919197116	-1.988702508
1.512320794	0.903574134	1.86360319	0.613447459	1.722003138	0.564141602	-2.393030105	-2.393030105	-2.393030105
1.339576065	0.894618133	2.972609436	0.807872739	0.79703493	0.699128855	-2.577704825	-2.298624979	-2.634510355
0.940104417	0.401968102	2.446738557	1.023675249	1.004290709	0.869883798	-2.318898929	-2.449328053	-2.91843385
0.463633492	1.07302345	2.651125463	0.941881871	1.873966335	1.28627626	-3.131049774	-2.456849661	-2.702007436
1.157012196	1.240171393	2.352091003	0.403366793	1.436231325	1.177339509	-3.144102151	-3.151510683	-3.470599385
0.577968503	1.520610815	2.021116513	1.028499284	0.32759332	1.217956662	-1.861050112	-3.165518997	-1.667175988
1.007014094	0.988284235	2.008356164	0.653780791	1.874750372	0.522698779	-2.105813904	-2.534358656	-2.414711875
1.253642706	1.202795469	2.099938079	1.210482796	1.052241282	0.662750914	-3.609451161	-2.030697095	-1.84170299
1.77220402	1.237171758	2.42172409	1.033237352	1.120292085	1.03968139	-3.454316522	-2.782582767	-2.387411407
1.494608009	1.000358693	1.683945918	1.108430366	1.268377556	1.107978323	-2.968936177	-1.839199069	-2.85556362
1.688040136	0.991520999	1.975648822	1.401442299	1.549659448	1.403144976	-3.45368497	-2.862392505	-2.693379206
1.436526755	0.791854634	1.349949389	0.678442424	1.418135776	1.041108158	-2.811935568	-2.601954908	-2.302126659
1.77220402	1.237171758	2.42172409	1.033237352	1.120292085	1.03968139	-3.454316522	-2.782582767	-2.387411407
1.34786124	1.333887114	2.352715535	1.135901526	0.701358656	0.757491275	-2.543071782	-2.543071782	-2.543071782
0.78453186	1.194173692	3.0508047	0.533090059	1.699665697	1.2018183	-2.749571756	-2.966181825	-2.748330728
1.635707062	0.880104438	1.626038178	0.585691883	1.610132334	0.47181468	-1.831278458	-1.998671296	-2.979538821
1.535457702	0.601215024	1.790736039	1.477272956	1.687355389	1.648271797	-3.201364017	-2.644429499	-2.894515391
0.853155952	0.592132995	2.499628578	0.867670601	1.708870318	1.048459295	-3.211864088	-3.747106507	-3.610947144
1.24701478	1.084425811	2.483163764	1.016660389	0.857287631	1.135853163	-2.630154441	-2.229775165	-2.964475932
0.625797168	0.947823302	2.60861131	1.034349309	0.753687309	1.49949065	-2.292906759	-2.253418469	-1.92343382
1.465498619	1.392444887	2.145229872	0.555954174	2.268362601	1.228588182	-2.773233925	-2.44636388	-3.836480529
0.761773293	1.491583254	1.546614941	0.584191726	2.51202096	1.542565837	-2.988940392	-2.460869227	-2.988940392
1.196034832	0.823819937	1.990694176	0.997721682	0.620981669	1.838691082	-2.187533256	-2.498133345	-1.782276777
**WT-C**	**KO-C**	**OE-C**	**WT-2h**	**KO-2h**	**OE-2h**	**WT-12h**	**KO-12h**	**OE-12h**
-0.623502355	-0.846306916	-0.716694178	-0.400580213	-0.846306916	-0.75952315	1.882007911	2.372260317	3.546353965
-0.327866753	-0.434208548	-0.25340026	-0.887882896	-0.863360577	-0.816839433	2.146735301	2.1575276	3.594350768
-0.708392873	-0.630670577	-0.235953063	-0.634458838	-0.658684954	-0.766787561	1.8103002	2.314808483	3.139456148
-0.963770618	-1.414609366	-1.308820749	-0.449810498	-0.96090407	-1.475237866	1.754672432	2.353984444	3.1123175
-0.964698951	-0.757974632	-1.389288738	-0.083642991	-0.35782371	-0.376701114	1.954783813	1.859684081	3.678859432
-0.611203516	-0.72218335	-0.775989794	-0.158691311	-0.700114927	-0.594569154	2.870633714	2.55194952	3.244067858
-0.335488281	-0.573395069	-0.291393159	-0.346917137	-1.078283978	-1.041916858	1.307244279	2.616084156	2.976234359
**WT-C**	**KO-C**	**OE-C**	**WT-2h**	**KO-2h**	**OE-2h**	**WT-12h**	**KO-12h**	**OE-12h**
-1.689427641	-2.542002449	-2.36207927	-0.12888144	0.337361313	0.239557826	3.447656709	2.438794751	2.902020245
-2.796474864	-2.51447558	-2.831648404	-0.399079786	-0.258603599	-0.084695084	3.465087487	3.350605217	4.069284615
-2.537133946	-2.231077243	-2.992362517	-0.668704226	-0.644980325	-0.242399259	3.396464173	3.131117455	2.789075887
-2.228776466	-2.369195631	-2.893259307	-0.002618063	-0.444611255	-0.479394474	3.359784488	3.35766438	4.300256198
-2.009490374	-2.055593485	-2.493886336	-0.219475567	-0.451241999	-0.666067312	3.366629584	3.062083158	3.467042331
-2.188383174	-2.108916288	-3.192018695	-0.653290498	0.21678685	-0.017248366	3.395696535	2.799970019	3.429373936
-1.400096042	-1.756411102	-3.336244927	-0.329859615	-0.962427308	-0.425877217	3.68247156	2.541739767	3.374270676
-2.955578654	-2.435684727	-2.420178141	-0.500009411	-0.508391963	0.151261214	3.109509262	3.145763198	2.994358444
-2.824704301	-2.375504252	-2.484813971	-0.817102578	-0.463465623	-0.01222545	3.063041661	3.187510157	3.727264356
-2.641571821	-2.244632805	-3.389457635	-0.048211363	-0.717309654	0.456114408	3.916361969	2.515132717	3.153574185
-1.441090541	-2.104095496	-3.203035003	-0.321673694	-0.522678947	-0.151638391	3.567645173	3.086778685	3.089788214
**WT-C**	**KO-C**	**OE-C**	**WT-2h**	**KO-2h**	**OE-2h**	**WT-12h**	**KO-12h**	**OE-12h**
3.139412939	3.517426617	3.517426617	-0.517426617	-0.517426617	-0.517426617	-0.517426617	-0.517426617	-0.517426617
3.784948087	3.473118511	3.473118511	-0.473118511	-0.473118511	-0.473118511	-0.473118511	-0.473118511	-0.473118511
3.338545227	3.646909663	3.646909663	-0.646909663	-0.646909663	-0.646909663	-0.646909663	0.189822417	-0.646909663
3.180159937	3.154117049	3.406597905	-0.290355535	-0.529417803	-0.568770631	-1.157449025	-0.138734967	-0.242951121
2.866797521	3.138418548	3.306720983	-0.416415848	-0.416415848	-0.416415848	-0.416415848	-0.416415848	-0.416415848
2.81544586	3.344126197	3.344126197	-0.344126197	-0.344126197	-0.344126197	-0.106562479	-0.344126197	-0.344126197
2.999293209	3.374911651	3.374911651	-0.374911651	-0.374911651	-0.374911651	-0.374911651	-0.374911651	-0.374911651
2.917766712	3.506390132	3.506390132	-0.226964209	-0.506390132	-0.506390132	-0.506390132	-0.506390132	-0.506390132
2.827620827	3.435469258	3.188204339	-0.408026256	-1.118519639	-0.227982858	-0.625931287	-0.280499255	-0.013926452
2.974429927	3.551469055	3.413483998	-0.413483998	-0.413483998	-0.413483998	-0.413483998	-0.413483998	-0.044994997
3.039290829	3.438386498	3.438386498	-0.029414658	-0.438386498	-0.438386498	-0.438386498	-0.438386498	-0.438386498
2.92965893	3.349120737	3.349120737	-0.349120737	-0.349120737	-0.349120737	-0.349120737	-0.349120737	-0.349120737
2.841285869	3.77670044	3.876037816	-0.524378969	-0.888218691	-0.35129881	-0.642097331	-0.374131452	-0.413898873
3.153015496	3.519126937	3.519126937	-0.519126937	-0.519126937	-0.519126937	-0.519126937	-0.519126937	-0.519126937
3.175090952	3.365355111	3.375497121	-0.985990531	-0.985990531	-0.985990531	-0.985990531	-0.985990531	-0.985990531
3.828974961	3.620587335	3.779950594	-0.779950594	-0.779950594	-0.023781266	-0.779950594	-0.779950594	-0.779950594
3.520113049	3.565014131	3.565014131	-0.565014131	-0.565014131	-0.565014131	-0.565014131	-0.565014131	-0.565014131
2.854407038	3.323180088	3.323180088	-0.323180088	-0.323180088	-0.323180088	-0.323180088	-0.323180088	-0.323180088
2.886975039	3.298587188	3.298587188	-0.298587188	-0.298587188	-0.298587188	-0.298587188	-0.298587188	-0.298587188
2.884646284	3.301105808	3.301105808	-0.301105808	-0.301105808	-0.301105808	-0.301105808	-0.301105808	-0.301105808
3.657771933	3.786218592	2.970407051	-0.830284141	-0.629474869	-0.791175104	-1.573583668	-0.016296126	-1.573583668
2.841848047	3.355231006	3.355231006	-0.355231006	-0.355231006	-0.355231006	-0.355231006	-0.355231006	-0.355231006
2.964496161	3.458005974	3.458005974	-0.241545658	-0.458005974	-0.458005974	-0.458005974	-0.458005974	-0.458005974
2.762523151	3.665834315	3.665834315	-0.363888225	-0.55511469	-0.665834315	-0.665834315	-0.665834315	-0.665834315
2.835642189	3.135715281	3.482813951	-0.482813951	-0.482813951	-0.482813951	-0.482813951	0.14759152	-0.133448674
3.310236741	3.415395364	3.415395364	-0.415395364	-0.415395364	-0.415395364	-0.40246919	-0.415395364	-0.415395364
3.36874807	3.343407462	3.343407462	-0.343407462	-0.343407462	-0.343407462	-0.343407462	0.035104162	-0.343407462
2.690451677	3.796043682	3.714918237	-0.858776241	-0.15561926	-0.584532944	-0.368571075	-0.558327874	-0.476988247
2.92872085	3.588071395	3.588071395	-0.523627678	-0.588071395	-0.588071395	-0.588071395	-0.588071395	-0.588071395
3.234966363	3.208981407	3.561236366	-0.552476127	-0.668169657	-1.117675373	-0.740740143	-0.763593228	-0.162529609
2.886506609	3.360813326	3.360813326	-0.360813326	-0.360813326	-0.360813326	-0.360813326	-0.360813326	-0.360813326
2.795485109	3.839899037	3.836408516	-0.446652535	-1.346601521	-0.573449124	-0.783931695	-0.279651277	-0.04150651
3.531540069	3.38087265	3.10840646	-0.541874554	-0.298627731	-0.608136566	-1.457311664	-0.633008035	-1.069547728
2.813305627	3.577200038	3.05387993	-0.251794695	-0.673567028	-0.658219064	-0.839639704	-0.461128081	-0.452277163
2.884122282	3.42463923	3.788920581	-0.354850981	-0.618378701	-0.712146119	-0.743012775	0.141106513	-0.536848815
2.716838933	3.341200251	3.595390578	-0.514304286	-0.595390578	-0.595390578	-0.595390578	-0.595390578	-0.595390578
3.099242556	3.095868774	3.232393255	0.059880459	-0.325251127	-0.838858155	-0.216215488	-1.095868774	-0.251884929
2.7855162	3.314409128	3.617751523	-0.904304226	-0.152866124	-0.252618929	-0.617751523	-0.617751523	-0.422403924
3.228386827	3.725169134	3.654451828	-0.654451828	-0.654451828	-0.02684499	-0.654451828	-0.654451828	-0.654451828
2.771136732	3.346392092	3.346392092	-0.346392092	-0.346392092	-0.346392092	-0.346392092	-0.346392092	-0.346392092
2.77312789	3.475482089	3.475482089	0.093036508	-0.475482089	0.152124749	-0.475482089	-0.475482089	-0.475482089
2.789395335	3.482905971	3.129256544	-0.137166966	-0.719199436	-0.459440162	-0.283010491	-0.305712388	-0.497028406
3.607118635	3.364458622	3.747861548	-0.31898034	-0.548853614	-0.599268427	-0.502715605	-1.513067392	-1.513067392
2.824013107	3.711222223	3.599699512	-0.599699512	-0.599699512	-0.599699512	-0.599699512	0.062961743	-0.599699512
**WT-C**	**KO-C**	**OE-C**	**WT-2h**	**KO-2h**	**OE-2h**	**WT-12h**	**KO-12h**	**OE-12h**
2.255591566	2.31810227	2.233678835	1.776157045	2.428099172	1.742246359	-2.873094702	-2.385825497	-2.685230575
2.04885345	2.267156003	1.412077966	1.961233806	3.08502047	2.259670867	-2.95894329	-2.03873679	-2.638625582
2.179948267	2.732794272	1.326900741	1.985402684	2.236840069	2.082385209	-2.550632653	-2.525851985	-2.692341412
2.560884782	2.316217313	2.628079111	1.920353294	2.483323873	1.103859172	-2.213099528	-2.783706736	-2.192991955
2.37872477	2.719903263	1.817698491	1.86288015	2.714541324	1.571167126	-2.889686689	-2.840187429	-2.577591465
2.820718688	2.63500231	2.017339753	1.967555385	2.548927018	1.931655073	-2.163808877	-2.330174463	-2.515772891
2.152080878	2.301597609	1.841833968	1.992446196	2.004234145	1.884298863	-2.899299138	-1.808880436	-2.468312084
2.909148877	2.356299652	2.680315267	1.621155309	2.52646763	1.215683536	-3.031666606	-2.949473981	-2.797032626
2.393572698	2.237398639	1.893477196	1.846251911	2.768097188	1.572836524	-2.497285095	-2.305365537	-2.422378128
2.117181811	2.124965228	1.700476462	1.903205006	2.340291491	0.940882163	-2.879047835	-2.636700807	-2.676889897
2.876490229	2.177244301	2.516741494	1.978098918	2.748629398	2.336811256	-2.842182178	-2.842182178	-2.842182178
2.287703347	2.162798771	2.781966874	1.908484019	2.309526714	1.452584809	-2.814708869	-2.932795525	-2.75556014
2.27377273	2.205802508	1.906203216	1.642619623	2.95754997	1.967640886	-3.057345086	-2.022351202	-2.326347185

AT3G26290.1	C71BQ_ARATHCytochrome P450 71B26 OS=Arabidopsis thaliana GN=CYP71B26 PE=2 SV=1
AT4G33040.1	GRXC6_ARATHGlutaredoxin-C6 OS=Arabidopsis thaliana GN=GRXC6 PE=2 SV=2
AT1G07900.1	LBD1_ARATHLOB domain-containing 1 OS=Arabidopsis thaliana GN=LBD1 PE=2 SV=1
AT5G13750.1	ZIFL1_ARATH ZINC INDUCED FACILITATOR-LIKE 1 OS=Arabidopsis thaliana GN=ZIFL1 PE=2 SV=1
AT2G25625.1	unnamed protein product
AT1G62570.1	GSOX4_ARATHFlavin-containing monooxygenase FMO GS-OX4 OS=Arabidopsis thaliana GN=FMOGS-OX4 PE=2 SV=1
AT3G44450.1	unnamed protein product
AT2G36640.1	LEAD8_DAUCAEmbryonic DC-8 OS=Daucus carota PE=3 SV=1
AT4G16750.1	ERF39_ARATHEthylene-responsive transcription factor ERF039 OS=Arabidopsis thaliana GN=ERF039 PE=2 SV=1
AT1G24580.1	XERIC_ARATHProbable E3 ubiquitin- ligase XERICO OS=Arabidopsis thaliana GN=XERICO PE=1 SV=1
AT5G20830.1	SUSY1_ARATHSucrose synthase 1 OS=Arabidopsis thaliana GN=SUS1 PE=1 SV=3
AT4G34710.1	SPE2_ARATHArginine decarboxylase 2 OS=Arabidopsis thaliana GN=SPE2 PE=2 SV=1
AT3G05640.1	P2C34_ARATHProbable phosphatase 2C 34 OS=Arabidopsis thaliana GN=At3g05640 PE=2 SV=1
AT2G42540.3	CR15A_ARATH COLD-REGULATED chloroplastic OS=Arabidopsis thaliana GN=COR15A PE=1 SV=1
AT3G28007.1	SWET4_ARATHBidirectional sugar transporter SWEET4 OS=Arabidopsis thaliana GN=SWEET4 PE=1 SV=1
AT5G59220.1	P2C78_ARATHProbable phosphatase 2C 78 OS=Arabidopsis thaliana GN=At5g59220 PE=2 SV=1
AT1G02820.1	LEA5_CITSILate embryogenesis abundant Lea5 OS=Citrus sinensis GN=LEA5 PE=2 SV=1
AT3G10910.1	ATL72_ARATHRING-H2 finger ATL72 OS=Arabidopsis thaliana GN=ATL72 PE=2 SV=1
AT5G29000.3	PHL1_ARATH PHR1-LIKE 1 OS=Arabidopsis thaliana GN=PHL1 PE=1 SV=1
AT2G18170.1	MPK7_ARATHMitogen-activated kinase 7 OS=Arabidopsis thaliana GN=MPK7 PE=1 SV=2
AT1G63010.4	SPXM1_ARATHSPX domain-containing membrane At1g63010 OS=Arabidopsis thaliana GN=At1g63010 PE=2 SV=1
AT5G57050.1	P2C77_ARATH phosphatase 2C 77 OS=Arabidopsis thaliana GN=ABI2 PE=1 SV=1
AT1G61255.1	GRP family [Medicago truncatula]
AT3G57680.1	CTPA3_ARATHCarboxyl-terminal-processing peptidase chloroplastic OS=Arabidopsis thaliana GN=CTPA3 PE=3 SV=1
AT4G40010.1	SRK2F_ARATHSerine threonine- kinase SRK2F OS=Arabidopsis thaliana GN=SRK2F PE=1 SV=1
AT1G19970.1	ERD2_CAEELER lumen -retaining receptor OS=Caenorhabditis elegans GN=erd-2 PE=3 SV=2
AT1G53470.1	MSL4_ARATHMechanosensitive ion channel 4 OS=Arabidopsis thaliana GN=MSL4 PE=3 SV=1
AT3G14440.1	NCED3_ARATH9-cis-epoxycarotenoid dioxygenase chloroplastic OS=Arabidopsis thaliana GN=NCED3 PE=2 SV=1
AT1G49450.1	MHCKB_DICDIMyosin heavy chain kinase B OS=Dictyostelium discoideum GN=mhkB PE=2 SV=1
AT4G35300.1	MSSP2_ARATHMonosaccharide-sensing 2 OS=Arabidopsis thaliana GN=MSSP2 PE=1 SV=2
AT4G00430.1	PIP14_ARATHProbable aquaporin PIP1-4 OS=Arabidopsis thaliana GN= PE=1 SV=1
**Cluster 21**	**Description**
AT3G06070.1	PREDICTED: uncharacterized protein LOC103403852
AT1G74670.1	GASA6_ARATHGibberellin-regulated 6 OS=Arabidopsis thaliana GN=GASA6 PE=3 SV=1
AT5G62280.1	DUF1442 family [Medicago truncatula]
AT1G13650.2	hypothetical protein AT1G13650
AT2G18300.2	HBI1_ARATHTranscription factor HBI1 OS=Arabidopsis thaliana GN=HBI1 PE=1 SV=3
AT1G68190.1	COLX_ARATH zinc finger At1g68190 OS=Arabidopsis thaliana GN=At1g68190 PE=2 SV=1
AT5G44680.1	3MG1_ECOLIDNA-3-methyladenine glycosylase 1 OS=Escherichia coli (strain K12) GN=tag PE=1 SV=1
AT5G57760.1	auxin canalization [Medicago truncatula]
AT5G48490.1	DIRL1_ARATH lipid-transfer DIR1 OS=Arabidopsis thaliana GN=DIR1 PE=1 SV=1
**Cluster 22**	**Description**
AT3G45140.1	LOX2_ARATHLipoxygenase chloroplastic OS=Arabidopsis thaliana GN=LOX2 PE=1 SV=1
AT5G17450.1	HIP26_ARATHHeavy metal-associated isoprenylated plant 26 OS=Arabidopsis thaliana GN=HIPP26 PE=1 SV=1
AT5G13550.1	SUT41_ARATHSulfate transporter chloroplastic OS=Arabidopsis thaliana GN=SULTR4 1 PE=1 SV=1
AT1G28600.2	GDL9_ARATHGDSL esterase lipase At1g28600 OS=Arabidopsis thaliana GN=At1g28600 PE=2 SV=1
AT3G44190.1	AIFA_DICDIApoptosis-inducing factor homolog A OS=Dictyostelium discoideum GN=aifA PE=3 SV=1
AT2G39780.2	RNS2_ARATHRibonuclease 2 OS=Arabidopsis thaliana GN=RNS2 PE=2 SV=1
AT1G74950.1	TI10B_ARATH TIFY 10B OS=Arabidopsis thaliana GN=TIFY10B PE=1 SV=1
AT1G28360.1	ERF81_ARATHEthylene-responsive transcription factor 12 OS=Arabidopsis thaliana GN=ERF12 PE=2 SV=1
AT1G30070.1	CYBP_MOUSECalcyclin-binding OS=Mus musculus GN=Cacybp PE=1 SV=1
AT1G71530.1	Y1960_ARATHProbable serine threonine- kinase At1g09600 OS=Arabidopsis thaliana GN=At1g09600 PE=3 SV=1
AT2G38170.2	CAX1_ARATHVacuolar cation proton exchanger 1 OS=Arabidopsis thaliana GN=CAX1 PE=1 SV=3
AT2G25140.1	CLPB4_ARATHChaperone mitochondrial OS=Arabidopsis thaliana GN=CLPB4 PE=2 SV=1
AT3G04710.1	PSD10_DICDI26S proteasome non-ATPase regulatory subunit 10 OS=Dictyostelium discoideum GN=psmD10 PE=2 SV=1
AT1G66080.1	HIKES_DANRE Hikeshi OS=Danio rerio GN=zgc:110091 PE=2 SV=2
AT5G13750.2	ZIFL1_ARATH ZINC INDUCED FACILITATOR-LIKE 1 OS=Arabidopsis thaliana GN=ZIFL1 PE=2 SV=1
AT5G56030.1	HS902_ARATHHeat shock 90-2 OS=Arabidopsis thaliana GN=HSP90-2 PE=1 SV=1
AT1G07350.1	SR45A_ARATHSerine arginine-rich splicing factor SR45a OS=Arabidopsis thaliana GN=SR45A PE=1 SV=1
AT5G09590.1	HSP7J_ARATHHeat shock 70 kDa mitochondrial OS=Arabidopsis thaliana GN=HSP70-10 PE=2 SV=1
AT5G64700.1	WTR45_ARATHWAT1-related At5g64700 OS=Arabidopsis thaliana GN=At5g64700 PE=2 SV=1
AT5G02244.1	SCE1_ARATHSUMO-conjugating enzyme SCE1 OS=Arabidopsis thaliana GN=SCE1 PE=1 SV=1
AT3G44310.1	NRL1_ARATHNitrilase 1 OS=Arabidopsis thaliana GN=NIT1 PE=1 SV=2
AT5G67460.1	E131_ARATHGlucan endo-1,3-beta-glucosidase 1 OS=Arabidopsis thaliana GN=At1g11820 PE=1 SV=3
AT4G34370.2	ARI1_ARATHProbable E3 ubiquitin- ligase ARI1 OS=Arabidopsis thaliana GN=ARI1 PE=2 SV=1
AT2G27200.1	LSG11_ARATHGTPase LSG1-1 OS=Arabidopsis thaliana GN=LSG1-1 PE=1 SV=1
AT5G17760.2	AATPI_ARATHAAA-ATPase At5g17760 OS=Arabidopsis thaliana GN=At5g17760 PE=2 SV=1
AT5G56010.1	HS903_ARATHHeat shock 90-3 OS=Arabidopsis thaliana GN=HSP90-3 PE=1 SV=2
AT5G59580.1	U76E1_ARATHUDP-glycosyltransferase 76E1 OS=Arabidopsis thaliana GN=UGT76E1 PE=2 SV=1
AT2G34660.2	AB2C_ARATHABC transporter C family member 2 OS=Arabidopsis thaliana GN=ABCC2 PE=1 SV=2
AT5G12020.1	kDa class II heat shock OS=Arabidopsis thaliana GN= PE=2 SV=1
AT4G29930.3	BH027_ARATHTranscription factor bHLH27 OS=Arabidopsis thaliana GN=BHLH27 PE=2 SV=1
AT1G76680.1	OPR1_ARATH12-oxophytodienoate reductase 1 OS=Arabidopsis thaliana GN=OPR1 PE=1 SV=2
AT1G61610.1	Y1661_ARATH G-type lectin S-receptor-like serine threonine- kinase At1g61610 OS=Arabidopsis thaliana GN=At1g61610 PE=3 SV=1
AT5G53400.1	BOB1_ARATH BOBBER 1 OS=Arabidopsis thaliana GN=BOB1 PE=1 SV=1
AT5G04370.2	BSMT1_ARATHSalicylate benzoate carboxyl methyltransferase OS=Arabidopsis thaliana GN=BSMT1 PE=1 SV=1
AT1G48720.1	U1 small nuclear ribonucleo 70 kDa- partial
AT3G59900.1	ARGOS_ARATH AUXIN-REGULATED GENE INVOLVED IN ORGAN SIZE OS=Arabidopsis thaliana GN=ARGOS PE=2 SV=1
AT1G10580.1	PRP17_HUMANPre-mRNA-processing factor 17 OS=Homo sapiens GN=CDC40 PE=1 SV=1
AT5G65280.1	GCL1_ARATH GCL1 OS=Arabidopsis thaliana GN=GCL1 PE=2 SV=1
AT1G51340.2	DTX42_ARATH DETOXIFICATION 42 OS=Arabidopsis thaliana GN=DTX42 PE=2 SV=2
AT5G10830.1	Y8948_DICDI methyltransferase DDB_G0268948 OS=Dictyostelium discoideum GN=DDB_G0268948 PE=1 SV=2
AT2G15480.1	U73B5_ARATHUDP-glycosyltransferase 73B5 OS=Arabidopsis thaliana GN=UGT73B5 PE=2 SV=1
AT5G53760.2	MLO11_ARATHMLO 11 OS=Arabidopsis thaliana GN=MLO11 PE=2 SV=1
AT4G15440.1	C74B2_ARATHLinolenate hydroperoxide chloroplastic OS=Arabidopsis thaliana GN=CYP74B2 PE=1 SV=1
AT5G33290.1	XGD1_ARATHXylogalacturonan beta-1,3-xylosyltransferase OS=Arabidopsis thaliana GN=XGD1 PE=1 SV=2
AT1G04770.1	SDI2_ARATH SULFUR DEFICIENCY-INDUCED 2 OS=Arabidopsis thaliana GN=At1g04770 PE=2 SV=1
AT5G55090.1	ANP1_ARATHMitogen-activated kinase kinase kinase ANP1 OS=Arabidopsis thaliana GN=ANP1 PE=1 SV=2
AT2G26310.1	FAP2_ARATHFatty-acid-binding 2 OS=Arabidopsis thaliana GN=FAP2 PE=2 SV=2
AT4G34550.1	FB348_ARATHF-box At2g16365 OS=Arabidopsis thaliana GN=At2g16365 PE=2 SV=2
AT1G44350.1	ILL6_ARATHIAA-amino acid hydrolase ILR1-like 6 OS=Arabidopsis thaliana GN=ILL6 PE=2 SV=2
**Cluster 23**	**Description**
AT1G05870.4	signal transducer transcription (DUF1685)
AT3G06550.1	RWA2_ARATH REDUCED WALL ACETYLATION 2 OS=Arabidopsis thaliana GN=RWA2 PE=1 SV=1
AT3G09600.1	RVE8_ARATH REVEILLE 8 OS=Arabidopsis thaliana GN=RVE8 PE=2 SV=1
AT5G08130.4	BIM1_ARATHTranscription factor BIM1 OS=Arabidopsis thaliana GN=BIM1 PE=1 SV=2
AT2G15020.1	PREDICTED: uncharacterized protein LOC103496192
AT1G78170.1	unnamed protein product
AT4G16515.1	RGF6_ARATHRoot meristem growth factor 6 OS=Arabidopsis thaliana GN=RGF6 PE=3 SV=1
AT5G47640.1	NFYB2_ARATHNuclear transcription factor Y subunit B-2 OS=Arabidopsis thaliana GN=NFYB2 PE=2 SV=1
AT1G17990.1	ORL2B_ARATH 12-oxophytodienoate reductase 2B OS=Arabidopsis thaliana GN=At1g18020 PE=3 SV=1
AT2G46450.3	CNG12_ARATHProbable cyclic nucleotide-gated ion channel 12 OS=Arabidopsis thaliana GN=CNGC12 PE=2 SV=2
AT1G69523.1	MET7A_HUMANMethyltransferase 7A OS=Homo sapiens GN=METTL7A PE=1 SV=1
AT4G32440.3	plant tudor-like RNA-binding [Medicago truncatula]
AT3G47500.1	CDF3_ARATHCyclic dof factor 3 OS=Arabidopsis thaliana GN=CDF3 PE=1 SV=2
AT4G17340.1	TIP22_ARATHProbable aquaporin TIP2-2 OS=Arabidopsis thaliana GN=TIP2-2 PE=1 SV=2
AT3G47295.1	PSY2_ARATH PSY2 OS=Arabidopsis thaliana GN=PSY2 PE=3 SV=1

2.831077516	2.495113381	1.418691276	1.840792602	2.162709276	1.737986501	-2.028899168	-1.971206688	-2.133882902
2.140462674	2.10359738	2.08359579	1.91185431	2.565097692	1.402543148	-2.722836191	-2.171709553	-1.812605249
2.51702035	2.105502909	1.409980252	1.788208256	2.184287907	1.801713653	-3.024304196	-1.915871867	-1.921490746
2.499396909	2.269195263	1.490715525	1.864944889	2.15049203	1.927937861	-2.956785265	-1.943111455	-2.303991939
2.398518001	2.030554038	1.453599504	1.821589503	2.36700585	1.3767326	-2.549136952	-1.907159459	-1.771646871
2.487726446	2.340328116	1.709646435	1.817168166	2.802729942	1.690867895	-2.664779773	-2.482917644	-2.265318233
2.042216887	2.103695869	1.698204926	1.721492363	1.553717855	1.564896969	-2.765433155	-1.826166558	-1.885233418
2.829573318	2.932999568	1.337627252	1.735271104	2.030422324	1.971140556	-2.368568826	-2.392494666	-2.416823995
2.505953697	2.155994978	2.190065107	1.795625468	2.305243492	1.155899154	-2.665093619	-1.942084885	-2.061643121
2.981896216	2.498002091	1.844120096	1.80354425	2.290920532	2.007031148	-1.494776251	-2.915793228	-1.531958416
2.730030116	2.452368664	2.558476792	1.612704421	2.882264922	1.544260915	-2.740438315	-2.920720892	-2.658886391
2.511097333	2.632174799	1.495558341	1.768824679	2.818061932	2.16104584	-3.07922843	-2.408546096	-1.876793732
2.950179648	2.006830547	1.860580536	1.882919693	2.717830646	1.711678419	-2.961398025	-2.085298645	-3.182963523
2.191272339	2.382109343	2.802726148	1.805755639	2.346340083	0.854572941	-2.62956519	-2.193403351	-2.62956519
2.077647464	2.478914708	2.465706561	1.957533609	2.269385997	1.923860945	-3.084424062	-1.74428995	-3.071082072
2.982791315	2.083786343	2.123691686	1.692314209	2.506108881	2.041027788	-1.51304097	-2.177469143	-3.022579687
2.746329704	2.382243616	1.383164001	1.908311183	2.809017005	1.899306271	-2.500025959	-2.12306739	-2.248131792
2.820718688	2.63500231	2.017339753	1.967555385	2.548927018	1.931655073	-2.163808877	-2.330174463	-2.515772891
2.938851514	2.586527891	1.498695163	1.857943118	2.156235892	1.956264066	-2.0750429	-2.0750429	-2.421524229
2.164667843	2.198004139	1.570718094	1.962124029	2.520873472	1.803854816	-2.731950981	-2.120246625	-2.502133159
2.149148839	2.765037808	1.763408997	1.835974059	2.165470303	2.11827502	-2.605515942	-2.287422519	-2.206078887
2.498879538	2.194645738	1.750879466	1.79097511	2.45947846	1.64365981	-1.611379703	-2.209776303	-1.80772544
2.308302101	2.51192984	2.097795989	2.12364225	2.793556286	1.39388957	-3.090629423	-1.75197351	-2.14604922
2.627618186	2.058922796	1.868608818	1.927007636	2.308798637	1.751743818	-2.515720522	-1.951455663	-1.885980461
2.704735392	2.456882396	2.247111527	1.913122065	3.022410175	1.899028523	-3.061774262	-2.396177533	-2.240292849
2.742502273	2.303976666	2.012776362	1.840506592	2.81521268	1.415091274	-3.207960953	-2.294495739	-2.342604609
2.154037589	2.846307937	2.386509194	1.932192543	2.971516948	2.116165956	-2.771551722	-2.962380185	-3.064723081
2.162361882	2.157156723	2.614629957	1.884445342	2.606095282	1.921104036	-1.817974462	-2.16197567	-1.726805878
2.118816549	2.621283113	2.511767209	1.969261635	2.659151781	1.774617676	-2.548920885	-3.00657482	-2.989433687
2.887132546	2.332807221	1.542156814	1.753835365	2.670572414	1.404721339	-1.649737961	-2.136857216	-2.030365429
2.314371149	2.117242684	2.228310159	1.906922631	1.845060562	1.578452378	-2.467834271	-2.496532195	-2.625993097

WT-C	KO-C	OE-C	WT-2h	KO-2h	OE-2h	WT-12h	KO-12h	OE-12h
-1.919904989	-2.398518824	-2.288431494	-1.079453478	-1.251451009	-0.815984957	2.634612402	4.547574055	3.571558294
-1.238336318	-2.865277287	-2.852523517	-1.240325817	-1.145319039	-0.046908625	2.859807852	3.847071401	3.68181135
-2.520981577	-2.319290329	-2.649274978	-1.757078268	-0.80891246	-0.438262785	2.616349444	4.854526913	4.022924041
-1.949717974	-2.57153088	-1.542611748	-0.903280015	-0.459928685	-0.390290565	2.825877869	3.999989303	3.991492695
-0.612189291	-2.379844089	-2.379844089	-1.353398109	-1.643455689	-0.720376152	2.671397485	3.868899455	3.54881048
-1.383065292	-2.258985876	-2.204791672	-0.563082219	-0.626143588	-0.17854726	2.463144722	3.120275987	3.631195198
-0.778310198	-1.875469606	-2.208760545	-1.309954005	-0.884856468	-0.188746394	2.015965852	3.456009559	3.774121804
-1.533936789	-2.463815263	-2.741204962	-1.174146336	-1.478771804	-1.448423213	2.684892028	4.423782392	3.731623947
-1.347576957	-1.128536039	-1.062367478	-1.817283326	-1.195496491	0.03088365	3.180557714	3.930585546	3.409233381

WT-C	KO-C	OE-C	WT-2h	KO-2h	OE-2h	WT-12h	KO-12h	OE-12h
0.43489626	0.354649505	0.696315646	1.354496682	0.375773685	0.886920533	-1.771385062	-1.667693634	-0.663973615
0.876290141	1.105120791	0.78412019	1.411065099	1.951934421	0.906528653	-2.215995019	-0.995394962	-2.057148742
0.493801293	0.635637278	1.140103334	1.114351314	1.259082573	0.787879208	-1.803305942	-1.789495248	-1.83805381
0.80515026	1.080196698	0.511900762	1.303998667	1.586415796	0.920917568	-1.802161196	-1.178498771	-2.067526388
0.683669163	1.014517506	1.130172651	1.328506542	0.789431112	1.160506856	-1.091454596	-1.801000312	-1.214348922
0.585327877	1.179841457	0.608607044	0.448498901	0.480397061	1.311575821	-1.108672534	-0.792831769	-1.712743858
0.581164857	0.710451913	0.652767056	0.96969631	0.587393858	1.394474947	-1.325774679	-1.966788982	-1.603385281
0.725871786	0.803998286	1.75289085	1.045732489	1.145026168	0.680214234	-1.660161935	-1.884134007	-1.609437871
0.605273136	1.277208795	0.581943439	1.356033973	0.324743745	1.159714476	-1.315329288	-1.787953371	-1.201634905
0.904356978	0.947201294	0.402671829	1.128030422	0.609783151	0.515842376	-2.20968067	-0.788524709	-2.20968067
0.543640977	1.276239655	0.69920416	1.533772902	0.462488546	1.229128834	-0.934694103	-1.457307229	-1.723189997
0.674070129	0.775338057	0.605065915	1.733436458	1.036997059	1.292913718	-1.908167734	-2.099206391	-1.765887888
0.931105123	0.752901077	0.765617312	0.908554014	0.384259936	0.789079192	-1.879748998	-1.274817436	-1.376950221
0.593985622	0.539396279	0.828651917	1.358493676	1.397723395	1.510982133	-1.694461203	-1.413119919	-1.32620511
0.74702518	1.474159527	0.740763578	1.541304288	1.93750084	1.31424299	-2.405463063	-1.840222064	-1.560992222
0.708887612	0.431015839	1.073490093	1.312395679	1.418289267	1.074124105	-1.38662008	-1.749433632	-1.045570349
0.415290523	0.327633856	0.786927369	1.336824252	0.401390193	0.996568165	-0.994843554	-1.585809545	-1.683981259
0.313204484	0.805442513	1.779787022	0.89387933	1.241873961	1.004609178	-1.605777769	-2.016210369	-1.41680835
1.026054107	0.359262694	0.322011298	1.45878386	0.609730466	1.421234328	-1.974683377	-0.602939025	-1.567346137
0.439585974	0.719397356	0.785969341	1.812066981	0.976004623	0.519227615	-1.240609103	-1.682092583	-1.682092583
0.892796837	0.617347071	1.519311906	0.409075496	0.38362856	0.900352996	-1.951269322	-1.749678844	-1.021564701
1.150700407	0.905648674	1.120422979	1.256666369	1.228821928	1.216722095	-1.604469783	-1.073955066	-0.485473795
1.030762909	0.866624004	1.12098551	1.614543178	1.498929487	0.998274492	-2.227207971	-1.738802675	-1.384147331
0.608522501	0.322108413	0.816670498	1.023031157	1.507501633	0.831006875	-1.52199095	-1.792696062	-1.794154066
0.588041493	1.156033312	1.100056341	1.755174523	2.102812342	1.454214074	-1.991033727	-1.805814336	-1.847304715
0.679428973	0.744583485	1.559267146	1.346144455	1.263170051	1.060944069	-1.762494819	-2.136630054	-1.228073204
0.322180367	0.659826273	1.030992986	0.785960569	0.563188466	1.205901211	-1.359823661	-1.468387494	-1.245995072
0.414100379	0.649765049	0.783598151	1.206027011	1.440192083	1.086342226	-1.805405927	-1.904585548	-1.870033423
1.154285142	0.490784345	1.017914318	1.595241853	0.960187614	1.51006879	-1.950904219	-1.048564611	-1.693184597
0.342797817	0.723263546	0.658907197	1.907850642	0.512549361	1.011158786	-1.536570809	-1.609604436	-1.459414913
1.092212134	0.46354857	1.146862243	0.82554631	1.327094946	0.998483294	-1.919552361	-1.519113655	-1.097887769
1.031055182	0.705593552	0.613392494	0.42299787	0.820646877	1.109642224	-1.176143566	-1.342466331	-1.184718302
0.994907815	0.534205253	1.357008501	1.144189583	1.02695666	0.653070172	-1.847452327	-1.680499438	-1.182386218
1.039430686	0.341329509	0.716197961	1.466958933	1.213646956	1.208469815	-1.530514139	-1.530514139	-0.84614421
1.283869064	0.817901613	1.00970897	1.906627547	1.308035376	1.103516509	-1.799322854	-0.889868008	-1.556659338
0.190295988	0.435770272	1.386968015	1.434141519	0.798822592	1.024345654	-0.889580331	-1.218323247	-1.388504432
1.23406398	0.471666144	0.667526545	1.558382866	0.894426508	1.132748057	-1.944341794	-2.12776443	-1.886707875
0.642717968	0.465750209	0.616285561	1.281642647	1.554959046	1.166093001	-1.821058941	-1.979664769	-1.926724722
0.139522126	0.684100859	0.730173335	1.070867687	1.990334431	1.243430843	-1.484102765	-1.038951896	-1.362527844
1.124512833	1.271508693	1.236386977	1.679343658	1.807690952	0.774984925	-2.027350998	-1.251911256	-1.599374443
0.66253113	1.286501307	0.520405603	1.235672608	1.693283574	0.926462277	-1.052761734	-1.671421359	-0.75930575
0.105804858	0.678682534	0.836859911	1.039744493	1.771631447	1.526825787	-1.39473663	-1.166483557	-1.234340644
0.358265749	1.11706524	0.430264598	1.663024388	1.513129808	1.403597158	-1.710253964	-0.94720693	-1.593755567
0.7933591	0.450371133	1.178507219	0.823396777	1.749787289	1.07261124	-1.90297622	-1.937716647	-1.227339892
1.083179165	0.922782932	0.504644302	1.323759447	1.142742048	0.776115681	-2.139703832	-2.00788987	-1.605629873
0.651637324	1.223451277	0.544757655	1.09259888	1.167545885	1.043976745	-1.577701163	-0.961312379	-0.849862454
0.430518783	0.471561933	0.663071071	0.38696079	1.560821013	0.788360881	-1.333317675	-0.973796505	-0.994180291
1.039296897	1.22388954	1.101395912	1.210717638	1.146203707	1.159776478	-1.324300069	-1.282670445	-1.195715863
0.196751711	0.961596046	0.735614081	0.620443219	1.015719425	1.174402527	-0.916475122	-1.714078883	-1.208536563

WT-C	KO-C	OE-C	WT-2h	KO-2h	OE-2h	WT-12h	KO-12h	OE-12h
-1.403824834	-2.060092369	-0.181955602	1.500744842	1.241056257	0.696503981	1.226493114	0.728753114	1.1918007
-1.481452117	-2.153152736	-0.918038416	1.113933554	1.266251317	0.755468055	1.578899337	0.616830526	1.212046425
-1.769617515	-1.25674701	-0.701440811	0.700815956	1.210610876	0.813183419	1.271980478	1.362888138	0.683264687
-2.367134782	-1.873667243	-0.562102943	0.702867625	1.203575756	1.348356322	0.613819107	0.864634124	0.696520337
-1.992878525	-1.522017655	-0.625205723	0.977776504	0.914876878	1.211368683	1.128665582	1.473506886	1.43390737
-1.313912087	-2.460684528	-1.524197394	1.659260058	1.795287715	0.812049664	1.358728583	1.706285905	1.31536075
-1.259584141	-2.273880386	-0.274965119	0.793349323	1.235825452	0.822326185	1.174380857	0.522317167	1.391007625
-2.029234216	-1.709070256	-0.365154396	1.085339289	1.881709726	1.167339192	1.258284981	1.102546223	1.186668096
-1.777957798	-1.488018854	-0.873247544	1.152117882	1.299170307	1.522609012	1.205104303	1.027708415	1.067485724
-1.943514987	-1.57648148	-0.077706304	1.815958035	1.878267724	0.973772819	1.254728191	1.131172057	1.032160963
-1.66660905	-1.403344347	-1.097859379	0.936320852	1.200965568	0.71825325	1.244513135	1.256587498	1.188827526
-1.864302977	-2.064681775	-0.550180739	1.143835485	1.961764205	0.707203803	1.760968806	1.1238774	1.533612282
-2.279290524	-1.381227982	0.072028008	1.072786893	1.429419798	0.845708307	1.406566701	1.052984705	1.113006495
-2.272315655	-2.234160914	-1.552680588	0.999087242	1.837551447	1.556118894	0.605201358	0.904003316	0.719489974
-1.406414475	-2.417924797	-0.71221645	1.705850642	1.44928562	0.847947854	1.149028035	0.570508639	1.288008998

AT4G24700.1	PREDICTED: uncharacterized protein LOC103483829
AT1G05870.2	signal transducer transcription (DUF1685)
AT2G40960.1	R3HD1_HUMANR3H domain-containing 1 OS=Homo sapiens GN=R3HDM1 PE=1 SV=3
AT3G26890.2	F214B_PONAB FAM214B OS=Pongo abelii GN=FAM214B PE=2 SV=1
AT2G37170.1	PIP22_ARATHAquaporin PIP2-2 OS=Arabidopsis thaliana GN=PIP2-2 PE=1 SV=2
AT2G32160.3	CARME_RATCarnosine N-methyltransferase OS=Rattus norvegicus GN=Carnmt1 PE=1 SV=1
AT4G39800.1	INO1_ARATHInositol-3-phosphate synthase isozyme 1 OS=Arabidopsis thaliana GN=IPS1 PE=1 SV=3
**Cluster 24**	**Description**
AT2G45180.1	14KD_DAUCA14 kDa proline-rich OS=Daucus carota PE=2 SV=1
AT2G15680.1	CML1_ARATHCalmodulin 1 OS=Arabidopsis thaliana GN=CML1 PE=2 SV=1
AT1G78460.1	HEBP2_HUMANHeme-binding 2 OS=Homo sapiens GN=HEBP2 PE=1 SV=1
AT1G76110.1	HMGB9_ARATHHigh mobility group B 9 OS=Arabidopsis thaliana GN=HMGB9 PE=2 SV=1
AT4G38470.1	STY46_ARATHSerine threonine- kinase STY46 OS=Arabidopsis thaliana GN=STY46 PE=1 SV=1
AT5G02760.1	P2C67_ARATHProbable phosphatase 2C 67 OS=Arabidopsis thaliana GN=At5g02760 PE=2 SV=1
AT2G44080.1	ARL_ARATHARGOS OS=Arabidopsis thaliana GN=ARL PE=2 SV=1
AT2G25900.2	C3H23_ARATHZinc finger CCCH domain-containing 23 OS=Arabidopsis thaliana GN=At2g25900 PE=2 SV=1
AT5G19530.1	ACL5_ARATHThermospermine synthase ACAULIS5 OS=Arabidopsis thaliana GN=ACL5 PE=1 SV=1
AT3G50560.1	DH12A_XENLAVery-long-chain 3-oxoacyl- reductase-A OS=Xenopus laevis GN=hsd17b12-a PE=2 SV=1
AT2G30770.1	C71AD_ARATHIndoleacetaldoxime dehydratase OS=Arabidopsis thaliana GN=CYP71A13 PE=1 SV=1
AT4G36410.1	UBC17_ARATHProbable ubiquitin-conjugating enzyme E2 17 OS=Arabidopsis thaliana GN=UBC17 PE=2 SV=1
AT5G25460.1	DUF642 family [Medicago truncatula]
AT3G13450.1	ODBB2_ARATH2-oxoisovalerate dehydrogenase subunit beta mitochondrial OS=Arabidopsis thaliana GN=DIN4 PE=1 SV=1
AT5G28770.3	BZP63_ARATHBasic leucine zipper 63 OS=Arabidopsis thaliana GN=BZIP63 PE=1 SV=1
AT1G25230.1	PPA4_ARATHPurple acid phosphatase 4 OS=Arabidopsis thaliana GN=PAP4 PE=2 SV=1
AT2G28630.1	KCS12_ARATH3-ketoacyl- synthase 12 OS=Arabidopsis thaliana GN=KCS12 PE=2 SV=1
AT2G41250.1	HDHD3_BOVINHaloacid dehalogenase-like hydrolase domain-containing 3 OS=Bos taurus GN=HDHD3 PE=2 SV=1
AT4G10910.1	DUF4228 domain [Medicago truncatula]
AT5G28770.2	BZP63_ARATHBasic leucine zipper 63 OS=Arabidopsis thaliana GN=BZIP63 PE=1 SV=1
AT1G13670.1	BIG GRAIN 1-like E
AT3G12710.1	3MG1_ECOLIDNA-3-methyladenine glycosylase 1 OS=Escherichia coli (strain K12) GN=tag PE=1 SV=1
**Cluster 25**	**Description**
AT3G19170.2	PREP1_ARATHPresequence protease chloroplastic mitochondrial OS=Arabidopsis thaliana GN=PREP1 PE=1 SV=2
AT5G08370.2	AGAL2_ARATHAlpha-galactosidase 2 OS=Arabidopsis thaliana GN=AGAL2 PE=1 SV=1
AT1G13608.1	DF288_ARATH defensin 288 OS=Arabidopsis thaliana GN=At1g13608 PE=5 SV=1
AT1G55450.1	Y8948_DICDI methyltransferase DDB_G0268948 OS=Dictyostelium discoideum GN=DDB_G0268948 PE=1 SV=2
AT4G37800.1	XTH7_ARATHProbable xyloglucan endotransglucosylase hydrolase 7 OS=Arabidopsis thaliana GN=XTH7 PE=2 SV=2
AT1G72416.2	DNAJ_THENNChaperone OS=Thermotoga neapolitana (strain ATCC 49049 DSM 4359 NS-E) GN=dnaJ PE=3 SV=1
**Cluster 26**	**Description**
AT5G64401.1	PPP7_ARATHSerine threonine- phosphatase 7 OS=Arabidopsis thaliana GN=PP7 PE=1 SV=1
AT2G36750.1	U73C1_ARATHUDP-glycosyltransferase 73C1 OS=Arabidopsis thaliana GN=UGT73C1 PE=2 SV=1
AT3G09440.1	HSP7C_ARATHHeat shock 70 kDa 3 OS=Arabidopsis thaliana GN=HSP70-3 PE=1 SV=1
AT3G22830.1	HFA6B_ARATHHeat stress transcription factor A-6b OS=Arabidopsis thaliana GN=HSFA6b PE=2 SV=1
AT1G29195.1	phosphatidylinositol 4-phosphate 5-kinase MSS4
AT1G56300.1	DNJB6_BOVIN homolog subfamily B member 6 OS=Bos taurus GN=DNAJB6 PE=2 SV=1
AT2G22470.1	AGP2_ARATHClassical arabinogalactan 2 OS=Arabidopsis thaliana GN=AGP2 PE=1 SV=1
AT5G51060.1	RBOHC_ARATHRespiratory burst oxidase homolog C OS=Arabidopsis thaliana GN=RBOHC PE=2 SV=2
AT2G40340.1	DRE2C_ARATHDehydration-responsive element-binding 2C OS=Arabidopsis thaliana GN=DREB2C PE=2 SV=2
AT4G28140.1	ERF54_ARATHEthylene-responsive transcription factor ERF054 OS=Arabidopsis thaliana GN=ERF054 PE=2 SV=1
AT3G16330.1	Avr9 Cf-9 rapidly elicited [Medicago truncatula]
AT3G11480.1	BSMT1_ARATHSalicylate benzoate carboxyl methyltransferase OS=Arabidopsis thaliana GN=BSMT1 PE=1 SV=1
AT2G21130.1	CP19B_ARATHPeptidyl-prolyl cis-trans isomerase CYP19-2 OS=Arabidopsis thaliana GN=CYP19-2 PE=1 SV=1
AT4G37990.1	CADH8_ARATHCinnamyl alcohol dehydrogenase 8 OS=Arabidopsis thaliana GN=CAD8 PE=1 SV=1
**Cluster 27**	**Description**
AT3G27690.1	CB21_GOSHIChlorophyll a-b binding chloroplastic OS=Gossypium hirsutum GN=CAB-151 PE=2 SV=2
AT1G13609.2	DF287_ARATHDefensin 287 OS=Arabidopsis thaliana GN=At1g13609 PE=2 SV=1
AT1G13609.1	DF287_ARATHDefensin 287 OS=Arabidopsis thaliana GN=At1g13609 PE=2 SV=1
AT4G35770.1	STR15_ARATHRhodanese-like domain-containing chloroplastic OS=Arabidopsis thaliana GN=STR15 PE=2 SV=1
**Cluster 28**	**Description**
AT1G76990.3	ACR3_ARATHACT domain-containing ACR3 OS=Arabidopsis thaliana GN=ACR3 PE=2 SV=1
AT5G16030.1	family transcriptional [Medicago truncatula]
AT2G39730.1	RCA_ARATHRibulose bisphosphate carboxylase oxygenase chloroplastic OS=Arabidopsis thaliana GN=RCA PE=1 SV=2
AT1G13650.1	hypothetical protein AT1G13650
AT3G62930.1	GRXS6_ARATHMonothiol glutaredoxin-S6 OS=Arabidopsis thaliana GN=GRXS6 PE=3 SV=1
AT3G23080.1	STAR7_HUMAN -related lipid transfer mitochondrial OS=Homo sapiens GN=STARD7 PE=1 SV=2
AT3G02380.1	COL2_ARATHZinc finger CONSTANS-LIKE 2 OS=Arabidopsis thaliana GN=COL2 PE=1 SV=1
AT1G32060.1	chloroplastic OS=Arabidopsis thaliana GN=At1g32060 PE=2 SV=1
AT1G26945.1	PRE6_ARATHTranscription factor PRE6 OS=Arabidopsis thaliana GN=PRE6 PE=1 SV=1
AT2G32880.1	UBP12_ARATHUbiquitin carboxyl-terminal hydrolase 12 OS=Arabidopsis thaliana GN=UBP12 PE=1 SV=2
AT3G61210.1	Y8948_DICDI methyltransferase DDB_G0268948 OS=Dictyostelium discoideum GN=DDB_G0268948 PE=1 SV=2
AT1G66940.1	Y1670_ARATHProbable receptor kinase At1g67000 OS=Arabidopsis thaliana GN=At1g67000 PE=2 SV=2
AT1G03130.1	PSAD2_ARATHPhotosystem I reaction center subunit II- chloroplastic OS=Arabidopsis thaliana GN=PSAD2 PE=1 SV=1
AT1G51400.1	PST2_ARATHPhotosystem II 5 kDa chloroplastic OS=Arabidopsis thaliana GN=PSBT PE=3 SV=2
AT2G24270.4	GAPN_ARATHNADP-dependent glyceraldehyde-3-phosphate dehydrogenase OS=Arabidopsis thaliana GN=ALDH11A3 PE=1 SV=2
AT5G14200.2	LEU33_ARATH3-isopropylmalate dehydrogenase chloroplastic OS=Arabidopsis thaliana GN=IMDH3 PE=1 SV=1
AT5G18600.1	GRXS2_ARATHMonothiol glutaredoxin-S2 OS=Arabidopsis thaliana GN=GRXS2 PE=3 SV=1
AT2G39730.2	RCA_ARATHRibulose bisphosphate carboxylase oxygenase chloroplastic OS=Arabidopsis thaliana GN=RCA PE=1 SV=2
AT1G15820.1	CB4A_SOLLCChlorophyll a-b binding CP24 chloroplastic OS=Solanum lycopersicum GN=CAP10A PE=3 SV=1
AT2G29290.2	TRNH6_ARATHTropinone reductase homolog At2g29290 OS=Arabidopsis thaliana GN=At2g29290 PE=2 SV=1
AT1G79700.2	AP2L3_ARATHAP2-like ethylene-responsive transcription factor At1g79700 OS=Arabidopsis thaliana GN=At1g79700 PE=2 SV=1
AT2G21195.3	hypothetical protein MTR_7g057760 [Medicago truncatula]
AT3G15356.1	LECT2_ARATHLectin LEC OS=Arabidopsis thaliana GN=LEC PE=1 SV=1
**Cluster 29**	**Description**
AT4G36800.2	RCE1_ARATHNEDD8-conjugating enzyme Ubc12 OS=Arabidopsis thaliana GN=RCE1 PE=1 SV=1
AT5G09600.1	SDH32_ARATHSuccinate dehydrogenase subunit 3- mitochondrial OS=Arabidopsis thaliana GN=SDH3-2 PE=1 SV=1
AT4G03520.2	TRXM2_ARATHThioredoxin chloroplastic OS=Arabidopsis thaliana GN=At4g03520 PE=1 SV=2
**Cluster 30**	**Description**
AT3G48520.1	C94B3_ARATHCytochrome P450 94B3 OS=Arabidopsis thaliana GN=CYP94B3 PE=2 SV=1
AT4G36950.1	YODA_ARATHMitogen-activated kinase kinase kinase YODA OS=Arabidopsis thaliana GN=YDA PE=1 SV=1
AT3G59220.1	PRN1_ARATHPirin-1 OS=Arabidopsis thaliana GN=PRN1 PE=1 SV=1
AT3G46660.1	U7E12_ARATHUDP-glycosyltransferase 76E12 OS=Arabidopsis thaliana GN=UGT76E12 PE=2 SV=1
AT1G05100.1	ANP1_ARATHMitogen-activated kinase kinase kinase ANP1 OS=Arabidopsis thaliana GN=ANP1 PE=1 SV=2
AT3G51860.1	CAX3_ARATHVacuolar cation proton exchanger 3 OS=Arabidopsis thaliana GN=CAX3 PE=1 SV=1
AT1G43160.1	RAP26_ARATHEthylene-responsive transcription factor RAP2-6 OS=Arabidopsis thaliana GN=RAP2-6 PE=2 SV=2
AT5G24080.1	Y5248_ARATHG-type lectin S-receptor-like serine threonine- kinase At5g24080 OS=Arabidopsis thaliana GN=At5g24080 PE=2 SV=1
AT1G10585.1	basic helix-loop-helix (bHLH) DNA-binding superfamily
AT2G20560.1	DJB13_MOUSE homolog subfamily B member 13 OS=Mus musculus GN=Dnajb13 PE=1 SV=1
AT3G14200.1	DNJB8_HUMAN homolog subfamily B member 8 OS=Homo sapiens GN=DNAJB8 PE=1 SV=1
AT3G12580.1	MD37C_ARATHProbable mediator of RNA polymerase II transcription subunit 37c OS=Arabidopsis thaliana GN=MED37C PE=1 SV=1
AT5G05410.1	DRE2A_ARATHDehydration-responsive element-binding 2A OS=Arabidopsis thaliana GN=DREB2A PE=1 SV=1
AT3G63060.1	EDL3_ARATHEID1-like F-box 3 OS=Arabidopsis thaliana GN=EDL3 PE=2 SV=1
AT1G07610.1	AC022464_38F22G5.2
AT3G19270.1	ABAH4_ARATHAbscisic acid 8 -hydroxylase 4 OS=Arabidopsis thaliana GN=CYP707A4 PE=2 SV=2
AT1G68620.1	CXE6_ARATHProbable carboxylesterase 6 OS=Arabidopsis thaliana GN=CXE6 PE=2 SV=1
**Cluster 31**	**Description**
AT5G52780.1	PAM68_ARATH chloroplastic OS=Arabidopsis thaliana GN=PAM68 PE=1 SV=1
AT4G24230.5	ACBP3_ARATHAcyl- -binding domain-containing 3 OS=Arabidopsis thaliana GN=ACBP3 PE=1 SV=1
AT5G03350.1	LECT6_ARATHLectin At5g03350 OS=Arabidopsis thaliana GN=At5g03350 PE=1 SV=1

-1.498030203	-2.387234906	-0.203749026	0.629364426	1.281958403	1.630288934	1.362526198	0.833563189	0.513129846
-1.607750115	-1.943938533	-0.693370541	0.986419529	1.587574063	1.368479492	0.670020771	0.849648565	0.859354648
-1.456763878	-1.77983312	-1.391666753	0.874204366	1.863295117	1.4008729	0.882780084	1.421614599	1.22000124
-2.219817953	-2.219817953	-0.512293162	1.737725248	1.667902264	1.15174091	1.043817451	0.788991603	0.561751592
-1.619162713	-1.35702642	-0.717526261	1.099099327	1.050661485	1.143358505	1.040048026	0.776216477	1.415668426
-1.581409135	-2.413286376	-0.971802896	1.269062185	1.423243745	1.577849944	1.199161571	1.614133113	0.813709897
-1.933274462	-1.872749362	0.280389831	1.047646054	1.69293465	1.559929389	1.186381068	1.358550291	1.229944741
**WT-C**	**KO-C**	**OE-C**	**WT-2h**	**KO-2h**	**OE-2h**	**WT-12h**	**KO-12h**	**OE-12h**
-0.449363614	-1.907794452	-2.056337731	-0.632186591	-1.011633061	-0.115972358	2.079560189	1.846854532	2.509042164
-0.112129935	-1.645194857	-1.645194857	-1.645194857	-1.370231347	-1.645194857	2.254947404	2.091518244	1.976212366
-0.62168161	-1.555407175	-1.527976543	-0.624663619	-0.852139643	-0.69908846	2.216381756	2.132709318	2.531865975
-0.689192071	-1.417803694	-1.310643737	-0.817371893	-1.451405873	-0.840329322	2.285330361	2.596703619	2.64471261
-0.231645587	-0.912510987	-2.026729858	-0.933581148	-1.396979435	-0.644329143	2.867982204	1.967954022	2.309839931
-0.618037701	-1.678866208	-1.413065137	-1.808804077	-1.838353804	-1.017232603	2.046967229	2.883035984	2.444356317
-0.412671237	-1.914700342	-1.6351755	-0.528595871	-0.239915814	-1.065994654	1.539303119	2.098063627	2.159686672
-0.332086988	-1.835036276	-2.096544828	-0.55346231	-1.705418887	-1.206960153	3.147484539	2.842419267	2.739605636
-1.349160954	-1.934123455	-1.731072756	-0.830450387	-0.204819563	-1.43594472	2.304335262	2.671199107	2.510037466
-0.241522729	-1.174690511	-2.214383388	-0.702484349	-1.03913243	-0.611273541	2.702908252	2.136140383	2.144438312
-0.450127077	-1.066632554	-0.83180051	-1.481278814	-0.772916796	-0.757675241	1.880883722	2.284693075	2.194854195
-1.146315756	-1.757711141	-1.371069768	-1.030729636	-0.87199922	-0.162117301	2.461146692	2.232005267	2.646790863
-0.198345222	-1.210998353	-0.657328246	-1.16874008	-0.828995633	-1.161998823	1.823430205	2.481676178	1.921299973
-0.42029741	-1.199367728	-1.659297864	-1.237449833	-0.987717089	-0.787536478	2.996023401	2.1404519	2.1551911
-0.293862459	-1.299263484	-1.430785538	-1.208132585	-1.243736957	-1.616412718	2.755060964	2.874956287	2.462176491
-0.888761933	-1.523718063	-1.457814284	-1.881069061	-0.848338194	-0.648523183	2.809423531	3.338783958	3.100017228
-0.673864641	-1.589792985	-1.430417903	-0.987687412	-1.26786489	-1.074998104	1.754430149	2.648220132	2.621975655
-1.372014777	-1.98030483	-2.154263011	-0.919523596	-0.625967602	-0.209925564	2.825471939	2.2599125	2.176614941
-0.465737911	-1.746694225	-1.746694225	-0.854497515	-1.198750914	-0.641016147	2.593155778	2.453606095	2.606629066
-0.591806324	-1.574868992	-1.166918385	-1.1271701	-1.157840549	-0.219701587	3.002737489	2.212423908	2.62314454
-1.118863527	-1.770472158	-1.935258436	-1.253584295	-1.283212774	-1.397216983	2.629119732	2.190310015	2.372752878
-0.882202189	-1.444116199	-1.323038224	-0.624216697	-0.516112441	-1.242245656	1.648014631	2.486960241	1.896956535
**WT-C**	**KO-C**	**OE-C**	**WT-2h**	**KO-2h**	**OE-2h**	**WT-12h**	**KO-12h**	**OE-12h**
-2.157056921	-1.905353341	-1.721224857	-1.91684898	-1.896791328	-1.91684898	1.312738943	1.953910269	1.490911639
-1.814223622	-1.929506754	-1.837724283	-1.341661745	-1.929506754	-1.929506754	1.1173335	1.733610113	1.627290492
-1.62381383	-1.468863632	-1.267983315	-1.323758277	-1.647341378	-0.996546887	2.00683425	2.134632576	2.650873862
-1.297692076	-1.567475884	-1.158328975	-1.972467752	-1.564504318	-1.261801152	1.921002021	1.499589719	2.085020467
-1.540753096	-1.637933572	-1.181091442	-1.410930102	-1.038460078	-0.911447177	1.371091397	1.365318505	1.622022681
-1.621502056	-1.751887442	-1.135765037	-1.418463708	-1.828167763	-1.224817106	1.801042892	2.507083667	1.988824291
**WT-C**	**KO-C**	**OE-C**	**WT-2h**	**KO-2h**	**OE-2h**	**WT-12h**	**KO-12h**	**OE-12h**
1.282844565	1.349530917	2.579131169	1.366686851	0.897587802	1.606793336	-2.637926193	-2.588792273	-4.855856175
1.810063507	1.301775893	2.742701815	1.423600648	2.069278339	1.172842863	-4.030323703	-2.6494135	-3.840525863
2.084343165	2.489640683	3.470049881	1.496410138	1.611288226	1.132440709	-2.600409001	-2.716002899	-3.319590062
0.998920022	2.136973745	3.162688485	1.809645125	1.432897969	0.881835005	-3.227495548	-3.20341663	-3.166095004
2.006647057	1.506541516	2.294541383	1.029120096	2.09266625	1.090340846	-3.885073438	-3.013912101	-3.120871608
2.073422839	1.741011056	2.326626264	1.562874763	1.408370041	1.569075892	-3.24659281	-3.675021514	-3.759766531
1.561512414	2.625509576	3.218537732	1.207657743	1.813594587	0.783764312	-2.797445301	-3.261121607	-4.152009454
0.668794456	1.812812366	2.497810274	0.806463964	0.651753548	2.255750586	-2.692414058	-3.099136289	-2.901834848
2.184369191	2.358017933	2.743807185	0.564998046	0.853138013	0.708291019	-2.977090889	-3.034280719	-3.401249778
0.642668765	2.164356308	1.87295803	1.811625735	1.242685718	1.721269738	-4.035977702	-2.519340758	-3.367652516
2.32756704	1.757196172	2.471645779	1.082015978	1.670488873	1.429184014	-4.27924873	-3.222196303	-3.236652823
0.333713041	1.339177801	3.12141987	0.915874428	1.801268066	2.274524654	-3.225743185	-3.371662723	-3.188571952
2.457929997	1.782784523	2.630221335	1.376490559	1.266065784	1.371561648	-3.968684983	-3.152178778	-3.764190085
1.756282769	1.999059137	2.34822257	0.660676136	1.858553574	1.844257586	-2.817317988	-3.341170925	-4.30856286
**WT-C**	**KO-C**	**OE-C**	**WT-2h**	**KO-2h**	**OE-2h**	**WT-12h**	**KO-12h**	**OE-12h**
-4.322263069	-4.014520281	-4.721951266	0.743338975	0.829438799	1.553507688	3.040794425	4.197569619	2.694085109
-4.507420481	-3.735674934	-5.011278014	-0.407621999	0.282975123	0.954921707	3.792630293	4.277917848	4.353550457
-4.312827263	-4.061583456	-3.994344118	-0.190230734	-0.208547762	0.400598774	3.916048773	4.242617622	4.208268164
-3.858932475	-3.863863883	-5.297655505	1.981244042	1.530890452	1.810079828	3.917098697	3.486772274	3.294366572
**WT-C**	**KO-C**	**OE-C**	**WT-2h**	**KO-2h**	**OE-2h**	**WT-12h**	**KO-12h**	**OE-12h**
-2.341372486	-1.951580937	-1.954151442	0.045848558	0.675146705	0.128801967	1.435928365	1.860901294	2.100477975
-1.840199958	-2.359427117	-1.991162124	-0.272429107	0.251763366	0.454290866	2.022184536	1.871034015	1.863945523
-3.004241443	-2.775703995	-1.770602018	-0.353310022	0.000758477	1.135252137	2.525510882	2.012199755	2.230136227
-2.661826104	-1.432238181	-2.661826104	0.31545382	0.029931371	0.41079424	2.305434745	2.603460754	1.090815459
-1.738661089	-2.600398552	-2.881184143	0.320524867	0.785717615	-0.055509037	2.20891982	2.043874925	1.916715594
-1.869850975	-2.812847142	-2.592316999	-0.109468716	0.406961473	0.035756655	1.501834601	1.886978244	1.552952858
-2.369444834	-2.227832186	-2.360324456	0.255020002	0.121252102	0.44326587	2.099085413	2.474184853	1.564793235
-2.215023147	-2.267054116	-1.792743707	-0.200027121	-0.070026108	0.799955383	2.055228279	1.745976223	1.943714314
-2.112237065	-2.255324021	-1.388645693	-0.18892871	0.414283163	-0.474088629	1.317048439	2.463991801	2.223900716
-1.865398052	-2.688130313	-2.803208996	0.146803647	0.576908104	1.063143216	2.162823919	1.825493406	1.581565068
-1.462451262	-2.924720617	-2.066108156	-0.125082311	0.535445434	0.437475302	2.132027367	1.744871017	1.728543226
-1.929182017	-2.110312473	-2.127018806	-0.882506556	-0.402590079	0.326216442	1.927547516	2.665283369	2.532562604
-1.803427854	-2.787155917	-2.716211729	0.600937579	0.424837038	-0.235944479	2.073325736	2.43481441	2.008825217
-1.188156593	-2.754639323	-2.295983856	0.492415218	0.582295495	-0.241660617	1.467694606	2.393246687	1.544788384
-2.276351546	-2.87176246	-2.343990263	0.392722837	1.115506931	0.058322619	2.91418943	1.297729156	0.713633295
-2.971409487	-2.676568215	-2.971409487	-0.397792592	0.67475317	0.221258133	1.8516265	1.855037309	2.414504669
-1.168960231	-2.2839704	-2.010947037	-0.34654381	0.822852783	0.115114379	1.811599794	1.364109286	1.696745236
-3.114115247	-2.880726659	-1.949872057	-0.333676737	0.422066011	1.17300603	2.439154038	2.070832606	2.173332015
-1.750615574	-3.008266783	-2.327041948	0.018555369	0.24276666	0.528897378	1.965288767	2.370288947	1.960127184
-2.012183922	-2.494855541	-2.901727274	-0.384872756	-0.240881423	0.209321779	2.570061273	3.037340675	2.21779719
-1.6859727	-2.82735568	-2.241037262	0.47655385	0.222270173	-0.188228038	2.606754926	2.064466578	1.572548153
-1.755141305	-1.755141305	-1.755141305	-0.289427986	0.584566202	-0.518189547	1.415584971	2.417826175	1.655064099
-1.037103517	-2.613041975	-1.617623417	-0.037026804	0.650449776	-0.438043487	1.405337278	1.268592853	2.418459292
**WT-C**	**KO-C**	**OE-C**	**WT-2h**	**KO-2h**	**OE-2h**	**WT-12h**	**KO-12h**	**OE-12h**
3.070659481	2.541486566	3.094122736	-2.541486566	-2.541486566	-2.541486566	2.132013325	3.063729067	2.541486566
3.264740609	2.410034627	3.385072375	-3.37787002	-3.37787002	-3.095726791	1.981791703	2.552890969	2.038162512
2.83424905	2.516924975	2.987859389	-2.636581791	-2.691029575	-2.308685467	1.290268798	2.199083422	2.54176115
**WT-C**	**KO-C**	**OE-C**	**WT-2h**	**KO-2h**	**OE-2h**	**WT-12h**	**KO-12h**	**OE-12h**
0.656235089	1.089318334	2.577096277	1.81238373	2.39784627	2.062042727	-3.065675091	-3.803881561	-3.725365775
1.319661825	1.462447842	1.863551399	1.119087999	1.890536137	2.667826695	-3.365854216	-3.324472928	-3.632784752
0.926414363	0.872978037	1.329648764	2.154513592	2.006175452	1.638023486	-3.310301383	-3.044618679	-2.572833633
1.310317778	1.325604316	2.06914709	1.973027515	2.677465447	2.308932899	-3.380916539	-4.29454428	-3.989034226
-0.031509496	0.577833724	2.213024475	2.027743521	3.40769027	2.564317015	-2.615800429	-4.670713111	-3.472585968
1.056676783	1.554104149	2.036185154	1.518364807	2.338812664	2.007107901	-2.933327339	-3.690002468	-3.88792165
0.646901433	0.86317583	1.707366009	1.945301119	2.787983967	1.838776359	-2.281921919	-4.520977698	-2.986605098
0.644086767	1.670174871	2.050267537	1.910315721	2.352831906	1.899646901	-3.737240526	-3.277412726	-3.512670451
1.50618177	0.300538853	1.483861416	2.120317873	2.442813837	2.320733178	-2.975859717	-3.395050293	-3.803536917
0.364359485	1.086958404	1.374362297	2.326161888	1.130664905	2.20482126	-2.327478058	-3.174766695	-2.985083485
0.751482192	1.233297017	1.75218809	2.052810027	1.33519353	1.948551588	-2.387813924	-3.619261459	-3.066447061
0.426952484	0.974378487	1.676634904	1.912770371	0.849304261	2.213881992	-2.2023049	-3.119975056	-2.731642544
0.550123661	1.62213899	1.638612378	1.593651089	1.853910544	1.762660413	-2.376497813	-2.838714799	-3.805884464
-0.130273754	0.522227467	1.851193399	1.693604628	3.198364568	2.746820919	-2.385068949	-3.740223283	-3.756644996
0.681061399	-0.070667882	1.608696993	1.663753388	2.383621168	1.995973522	-2.84719582	-2.900040546	-2.515202222
0.364849369	1.06127995	1.617052491	1.907179578	1.921940182	1.313922128	-2.716553298	-2.554390338	-2.91528006
0.305452922	0.478173315	1.355226034	1.538933727	2.930783792	1.969664244	-2.879609033	-3.459562682	-2.23906232
**WT-C**	**KO-C**	**OE-C**	**WT-2h**	**KO-2h**	**OE-2h**	**WT-12h**	**KO-12h**	**OE-12h**
0.060663871	-1.174706904	-0.880965971	-0.638307702	-1.239677751	-0.824189117	1.349222043	1.707736652	1.640224879
-0.825119558	-0.054152441	-1.556385046	-0.756199619	-0.257856239	-1.066252275	2.510858436	3.278659138	1.726447605
-0.385553336	-0.736116521	-0.740607404	-0.482830092	-0.135914797	-0.233814013	0.436421721	3.174471692	1.503942749

AT1G14600.1	MYBF_ARATH Myb family transcription factor At1g14600 OS=Arabidopsis thaliana GN=At1g14600 PE=2 SV=2
AT1G56520.1	ADR2_ARATHDisease resistance ADR2 OS=Arabidopsis thaliana GN=ADR2 PE=2 SV=1
AT1G33790.2	JAL4_ARATHJacalin-related lectin 4 OS=Arabidopsis thaliana GN=JAL4 PE=2 SV=1
AT1G71400.1	RLP12_ARATHReceptor 12 OS=Arabidopsis thaliana GN=RLP12 PE=2 SV=2
AT1G72910.1	TIR_ARATHToll interleukin-1 receptor OS=Arabidopsis thaliana GN=TIR PE=1 SV=1
AT5G57655.2	XYLA_ARATHXylose isomerase OS=Arabidopsis thaliana GN=XYLA PE=2 SV=2
AT1G19770.1	PUP14_ARATHProbable purine permease 14 OS=Arabidopsis thaliana GN=PUP14 PE=1 SV=1
AT3G50480.1	HR4_ARATHRPW8 4 OS=Arabidopsis thaliana GN=HR4 PE=2 SV=1
AT1G77060.1	CPPM_ARATHCarboxyvinyl-carboxyphosphonate chloroplastic OS=Arabidopsis thaliana GN=At1g77060 PE=2 SV=2
AT5G03310.1	ARG7_VIGRRIndole-3-acetic acid-induced ARG7 OS=Vigna radiata radiata GN=ARG7 PE=2 SV=1
AT3G48590.1	NFYC1_ARATHNuclear transcription factor Y subunit C-1 OS=Arabidopsis thaliana GN=NFYC1 PE=1 SV=1
AT1G09460.1	E13B_WHEATGlucan endo-1,3-beta-glucosidase OS=Triticum aestivum GN=GLC1 PE=2 SV=1
AT5G25830.1	GAT12_ARATHGATA transcription factor 12 OS=Arabidopsis thaliana GN=GATA12 PE=2 SV=1
AT1G19715.2	JAL3_ARATHJacalin-related lectin 3 OS=Arabidopsis thaliana GN=JAL3 PE=2 SV=1
AT4G08869.1	DF210_ARATHDefensin 210 OS=Arabidopsis thaliana GN=At4g08869 PE=2 SV=1
AT3G15620.1	UVR3_ARATH(6-4)DNA photolyase OS=Arabidopsis thaliana GN=UVR3 PE=1 SV=2
AT5G07440.3	DHE2_ARATHGlutamate dehydrogenase 2 OS=Arabidopsis thaliana GN=GDH2 PE=1 SV=1
AT4G31910.1	HHT1_ARATHOmega-hydroxypalmitate O-feruloyl transferase OS=Arabidopsis thaliana GN=HHT1 PE=1 SV=1
AT5G51480.1	SKS2_ARATHMonocopper oxidase SKS2 OS=Arabidopsis thaliana GN=SKS2 PE=1 SV=1
AT4G04040.1	PFPB2_ARATHPyrophosphate--fructose 6-phosphate 1-phosphotransferase subunit beta 2 OS=Arabidopsis thaliana GN=PFP-BETA2 PE=3 SV=1
AT5G44610.1	PCAP2_ARATHPlasma membrane-associated cation-binding 2 OS=Arabidopsis thaliana GN=PCAP2 PE=1 SV=1
AT4G11460.1	CRK30_ARATH cysteine-rich receptor kinase 30 OS=Arabidopsis thaliana GN=CRK30 PE=3 SV=1
AT4G26260.1	MIOX4_ARATHInositol oxygenase 4 OS=Arabidopsis thaliana GN=MIOX4 PE=2 SV=1
AT3G26320.1	C71BX_ARATHCytochrome P450 71B36 OS=Arabidopsis thaliana GN=CYP71B36 PE=3 SV=1
AT3G50270.1	Y3028_ARATHUncharacterized acetyltransferase At3g50280 OS=Arabidopsis thaliana GN=At3g50280 PE=3 SV=1
AT2G31110.1	TBL40_ARATH trichome birefringence-like 40 OS=Arabidopsis thaliana GN=TBL40 PE=2 SV=2
AT2G40000.1	HSPR2_ARATHNematode resistance -like HSPRO2 OS=Arabidopsis thaliana GN=HSPRO2 PE=1 SV=1
AT5G60800.2	TMV resistance N-like [Malus domestica]
AT5G19240.1	UGPI2_ARATHUncharacterized GPI-anchored At5g19240 OS=Arabidopsis thaliana GN=At5g19240 PE=2 SV=2
AT1G14860.1	NUD18_ARATHNudix hydrolase mitochondrial OS=Arabidopsis thaliana GN=NUDT18 PE=2 SV=1
AT4G03510.1	RMA1_ARATHE3 ubiquitin- ligase RMA1 OS=Arabidopsis thaliana GN=RMA1 PE=1 SV=1
AT4G27460.1	CBSX5_ARATHCBS domain-containing CBSX5 OS=Arabidopsis thaliana GN=CBSX5 PE=2 SV=2
AT1G02620.1	SAR1A_ARATHGTP-binding SAR1A OS=Arabidopsis thaliana GN=SAR1A PE=2 SV=1
AT1G13300.1	PCL1_ORYSJTranscription factor PCL1 OS=Oryza sativa japonica GN=PCL1 PE=2 SV=1
AT4G19820.1	CHIT1_HUMANChitotriosidase-1 OS=Homo sapiens GN=CHIT1 PE=1 SV=1
AT4G24230.1	ACBP3_ARATHAcyl- -binding domain-containing 3 OS=Arabidopsis thaliana GN=ACBP3 PE=1 SV=1
AT1G70850.3	MLP34_ARATHMLP 34 OS=Arabidopsis thaliana GN=MLP34 PE=2 SV=1
AT5G42680.1	MIZ1_ARATH MIZU-KUSSEI 1 OS=Arabidopsis thaliana GN=MIZ1 PE=1 SV=1
AT2G22890.1	FD4L2_ARATHFatty acid desaturase 4-like chloroplastic OS=Arabidopsis thaliana GN=FAD4L2 PE=2 SV=1
AT5G06690.1	TRL31_ARATHThioredoxin-like 3- chloroplastic OS=Arabidopsis thaliana GN=WCRKC1 PE=2 SV=3
AT3G10720.2	PME25_ARATHProbable pectinesterase pectinesterase inhibitor 25 OS=Arabidopsis thaliana GN=PME25 PE=2 SV=1
AT2G10940.1	ERLL1_ARAT 1-like lipid transfer 1 OS=Arabidopsis thaliana GN=AZI1 PE=1 SV=1
AT5G45930.1	CHLI2_ARATHMagnesium-chelatase subunit - chloroplastic OS=Arabidopsis thaliana GN=CHLI2 PE=1 SV=1
AT1G27670.1	transmembrane protein
AT5G26740.1	T184A_HUMANTransmembrane 184A OS=Homo sapiens GN=TMEM184A PE=2 SV=1
AT4G24230.2	ACBP3_ARATHAcyl- -binding domain-containing 3 OS=Arabidopsis thaliana GN=ACBP3 PE=1 SV=1
AT4G25110.1	MCA2_ARATHMetacaspase-2 OS=Arabidopsis thaliana GN=AMC2 PE=1 SV=1
AT5G65060.2	AGL31_ARATHAgamous-like MADS-box AGL31 OS=Arabidopsis thaliana GN=AGL31 PE=2 SV=2
AT2G37025.2	TBP1_ORYSJTelomere-binding 1 OS=Oryza sativa japonica GN=TBP1 PE=1 SV=2
AT1G05675.1	U74E1_ARATHUDP-glycosyltransferase 74E1 OS=Arabidopsis thaliana GN=UGT74E1 PE=3 SV=1
AT4G12690.2	phosphatase [Medicago truncatula]
AT1G73540.1	NUD21_ARATHNudix hydrolase chloroplastic OS=Arabidopsis thaliana GN=NUDT21 PE=2 SV=1
AT1G63260.2	TET10_ARATHTetraspanin-10 OS=Arabidopsis thaliana GN=TET10 PE=2 SV=1
AT4G34030.1	MCCB_ARATHMethylcrotonoyl- carboxylase beta mitochondrial OS=Arabidopsis thaliana GN=MCCB PE=2 SV=1
AT1G03580.1	MCC07_ARATHMATH domain and coiled-coil domain-containing At2g42465 OS=Arabidopsis thaliana GN=At2g42465 PE=3 SV=1
AT2G43140.2	BH129_ARATHTranscription factor bHLH129 OS=Arabidopsis thaliana GN=BHLH129 PE=2 SV=2
AT4G26630.2	DEK carboxy-terminal domain [Medicago truncatula]
AT1G14890.1	21KD_DAUCA21 kDa OS=Daucus carota PE=2 SV=1
AT2G17130.2	IDH2_ARATHIsocitrate dehydrogenase
AT2G22980.3	SCP13_ARATHSerine carboxypeptidase-like 13 OS=Arabidopsis thaliana GN=SCPL13 PE=2 SV=2
AT4G25170.2	PREDICTED: uncharacterized protein LOC103439613
AT1G76990.2	ACR3_ARATHACT domain-containing ACR3 OS=Arabidopsis thaliana GN=ACR3 PE=2 SV=1
AT4G19100.1	PAM68_ARATH chloroplastic OS=Arabidopsis thaliana GN=PAM68 PE=1 SV=1
AT5G18650.1	MIEL1_ARATHE3 ubiquitin- ligase MIEL1 OS=Arabidopsis thaliana GN=MIEL1 PE=1 SV=1
AT5G01210.1	DCR_ARATHBAHD acyltransferase DCR OS=Arabidopsis thaliana GN=DCR PE=2 SV=1
AT5G21950.1	HSAD_MYCTU4,5:9,10-diseco-3-hydroxy-5,9,17-trioxoandrosta-1(10),2-diene-4-oate hydrolase OS=Mycobacterium tuberculosis (strain ATCC 25618 H37Rv) GN=hsaD PE=1 SV=1
AT1G18270.1	YGBJ_ECOLIUncharacterized oxidoreductase OS=Escherichia coli (strain K12) GN=ygbJ PE=3 SV=1
AT2G37060.1	NFYB8_ARATHNuclear transcription factor Y subunit B-8 OS=Arabidopsis thaliana GN=NFYB8 PE=2 SV=1
AT3G43850.1	suppressor SRP40-like [Cucumis melo]
AT1G23870.1	TPS9_ARATHProbable alpha,alpha-trehalose-phosphate synthase
AT1G80460.1	GLPK_ARATHGlycerol kinase OS=Arabidopsis thaliana GN=GLPK PE=1 SV=1
AT5G63020.1	DRL40_ARATHProbable disease resistance At5g63020 OS=Arabidopsis thaliana GN=At5g63020 PE=2 SV=2
AT2G16050.1	cysteine histidine-rich C1 domain [Medicago truncatula]
AT1G33610.1	DR100_ARATHDNA-damage-repair toleration DRT100 OS=Arabidopsis thaliana GN=DRT100 PE=2 SV=2
AT1G24260.1	SEP3_ARATHDevelopmental SEPALLATA 3 OS=Arabidopsis thaliana GN=SEP3 PE=1 SV=1
AT2G29740.1	U71C2_ARATHUDP-glycosyltransferase 71C2 OS=Arabidopsis thaliana GN=UGT71C2 PE=1 SV=1
AT3G14310.1	PME3_ARATHPectinesterase pectinesterase inhibitor 3 OS=Arabidopsis thaliana GN=PME3 PE=2 SV=2
AT2G42690.1	PLA20_ARATHPhospholipase A1-IIdelta OS=Arabidopsis thaliana GN=At2g42690 PE=1 SV=1
AT2G14170.2	MMSA_ARATHMethylmalonate-semialdehyde dehydrogenase
AT1G13607.1	DF286_ARATHDefensin 286 OS=Arabidopsis thaliana GN=At1g13607 PE=3 SV=1
AT1G72820.1	S2544_PONABSolute carrier family 25 member 44 OS=Pongo abelii GN=SLC25A44 PE=2 SV=2
AT3G51710.1	KPRO_MAIZE receptor kinase 1 OS=Zea mays GN=PK1 PE=2 SV=2
AT3G14840.2	Y3148_ARATHProbable leucine-rich repeat receptor-like serine threonine- kinase At3g14840 OS=Arabidopsis thaliana GN=LRR-RLK PE=2 SV=1
AT4G01720.1	WRK47_ARATHProbable WRKY transcription factor 47 OS=Arabidopsis thaliana GN=WRKY47 PE=2 SV=2
AT1G58190.2	GSO2_ARATHLRR receptor-like serine threonine- kinase GSO2 OS=Arabidopsis thaliana GN=GSO2 PE=2 SV=2
AT3G20370.1	MCC20_ARATHMATH domain and coiled-coil domain-containing At3g58260 OS=Arabidopsis thaliana GN=At3g58260 PE=3 SV=1
AT3G53950.1	WSCD1_ARTBCWSC domain-containing ARB_07867 OS=Arthroderma benhamiae (strain ATCC MYA-4681 CBS 112371) GN=ARB_07867 PE=1 SV=1
AT1G09390.1	GDL2_ARATHGDSL esterase lipase At1g09390 OS=Arabidopsis thaliana GN=At1g09390 PE=2 SV=1
AT1G03457.1	BRN2L_ARATHRNA-binding BRN2 OS=Arabidopsis thaliana GN=BRN2 PE=2 SV=1
AT4G34460.4	GBB_ARATHGuanine nucleotide-binding subunit beta OS=Arabidopsis thaliana GN=GB1 PE=1 SV=1
AT5G49740.1	FRO7_ARATHFerric reduction oxidase chloroplastic OS=Arabidopsis thaliana GN=FRO7 PE=2 SV=1
AT4G39770.1	TPPH_ARATHProbable trehalose-phosphate phosphatase H OS=Arabidopsis thaliana GN=TPPH PE=2 SV=1
AT4G32060.1	MICU1_MOUSECalcium uptake mitochondrial OS=Mus musculus GN=Micu1 PE=1 SV=1
AT1G48300.1	PREDICTED: LOW QUALITY PROTEIN: uncharacterized protein LOC103452609 isoform X1
AT3G20680.1	PREDICTED: uncharacterized protein LOC103501617 isoform X1
AT5G04770.1	CAAT6_ARATHCationic amino acid transporter chloroplastic OS=Arabidopsis thaliana GN=CAT6 PE=2 SV=1
AT1G21100.1	OMT1_ARATHFlavone 3 -O-methyltransferase 1 OS=Arabidopsis thaliana GN=OMT1 PE=1 SV=1
AT5G64570.1	BXL4_ARATHBeta-D-xylosidase 4 OS=Arabidopsis thaliana GN=BXL4 PE=1 SV=1
AT5G24520.1	TTG1_ARATH TRANSPARENT TESTA GLABRA 1 OS=Arabidopsis thaliana GN=TTG1 PE=1 SV=1
AT4G11190.1	DIR13_ARATHDirigent 13 OS=Arabidopsis thaliana GN=DIR13 PE=2 SV=1
AT3G49260.1	IQD1_ARATH IQ-DOMAIN 1 OS=Arabidopsis thaliana GN=IQD1 PE=1 SV=1
AT1G54740.1	FAF- chloroplastic
AT3G23550.1	DTX18_ARATH DETOXIFICATION 18 OS=Arabidopsis thaliana GN=DTX18 PE=2 SV=1
AT2G34510.1	plant F18G18-200 [Medicago truncatula]
AT3G06680.1	RL292_ARATH60S ribosomal L29-2 OS=Arabidopsis thaliana GN=RPL29B PE=2 SV=2
AT3G62860.1	CSE_ARATHCaffeoylshikimate esterase OS=Arabidopsis thaliana GN=CSE PE=1 SV=1
AT1G68840.2	RAV2_ARATHAP2 ERF and B3 domain-containing transcription repressor RAV2 OS=Arabidopsis thaliana GN=RAV2 PE=2 SV=1

-0.155247764	-0.762732068	-0.926010881	-0.650088167	-1.363001821	-0.37103914	0.960720511	3.079202542	1.459479076
-0.286615697	-0.360818417	-0.727051139	-0.227056499	-0.221539494	-0.668331175	0.799415712	3.085990226	1.106006484
-1.001449022	-1.015449701	-1.189216769	-0.385989732	-0.842118098	-0.713094349	1.97202599	2.948614603	2.226677077
-0.414046192	-0.229970702	-0.684370634	0.05224411	-0.7020185	-0.317713597	1.021119953	3.366130726	1.640886287
-0.55903901	-0.399377656	-1.071995796	-1.083985997	-1.374043577	-0.78792692	2.007927748	3.106178091	3.162263118
-0.331528883	-0.95658834	-0.436633195	-1.649465382	-0.568221705	-0.041654093	2.143237729	3.154506901	1.686346967
-0.404892366	-0.277592144	-0.4067905	-1.197602045	-1.323180725	-0.835236444	1.762281638	3.308712765	2.374299822
-0.924150875	-0.981254921	-1.330425097	-0.478966601	-1.762714016	-1.109081139	1.485724858	2.845513649	2.407052392
-0.142941578	-0.86841223	-0.496793081	-0.498083481	-0.379186424	-0.20247169	1.360449026	2.664592273	0.66098023
-0.181400891	-1.036202976	-0.193473724	-0.484010809	-1.209970044	-0.352387088	1.909386133	3.065923674	1.613983073
-0.136854688	-1.25217761	-0.704855712	-0.67670183	-0.510863271	-0.485210908	1.348458769	3.08389299	1.334312259
-0.339821848	-0.818698975	-1.042851854	-0.373023943	-0.952056315	-0.955921024	1.956634274	3.353882205	2.17185748
-0.511033651	-1.11628934	-0.907923804	-0.631820796	-0.528774525	-0.874323593	1.79111208	2.819308148	1.959745482
-1.611917478	-0.812001275	-1.611917478	0.267004001	0.413111316	0.080063272	1.856405166	3.353774471	1.065478005
-0.570381348	-1.156305325	-1.156305325	-0.520318821	-1.156305325	-0.802417489	2.124799407	3.186818194	3.050416032
0.007423822	-1.194210039	-0.816808608	-0.105390006	0.132327309	-0.987566815	1.595936418	3.123672844	1.244615074
-0.368626219	-0.119039135	-1.389426348	0.004801985	-0.351802428	-0.170363288	2.015610392	3.199657595	0.578502635
-0.230486214	-0.761094896	-0.732070715	-0.665391951	-0.036890083	-0.463062408	0.850495282	3.057924358	1.080576628
-0.458503311	-0.291570311	-0.255454349	-0.429073834	-0.285278565	-0.279014139	1.153111582	3.398873597	1.244656524
0.15384787	-1.142778884	-1.310797339	-0.539937859	-0.713922098	-0.331092249	1.349448295	3.496229241	1.039003022
-0.889609805	-0.644172921	-0.991271592	0.720223315	-0.991271592	-0.991271592	1.005840129	3.36086612	1.363180569
-0.906454496	-0.825592664	-0.531145305	-1.203801867	-1.158653501	-0.866493916	1.83226941	2.594728056	2.065144283
-0.640119252	-0.640119252	-0.640119252	-0.640119252	-0.640119252	-0.640119252	2.000270308	2.640119252	2.480564457
-0.460484942	-0.508694035	-0.551338373	-0.22710381	-0.300376799	-0.35725132	0.898090942	3.389016339	1.118141999
-0.718473824	-0.989453679	-0.614658777	-0.562067146	-1.388495121	-0.459494125	1.583811129	2.457088008	1.691743534
0.148613767	-0.454192207	-1.25410841	-0.666263401	-1.25410841	-0.309249964	1.19214782	3.050110559	1.547050246
0.191145169	0.129466267	-0.864992964	-1.208045784	-1.419439622	-1.220899719	2.142362451	2.75562636	1.494777842
-0.22768912	-0.22768912	-0.22768912	-0.22768912	-0.22768912	-0.22768912	1.152486192	3.22768912	0.441337646
-1.322858641	-1.134541299	-0.607683134	-0.712415116	-0.998532165	-0.557462397	1.676782406	3.266108495	2.390601851
-0.331037934	-0.757605831	-0.981672884	0.110840772	-0.470916756	-0.941193815	1.289321911	3.234001504	1.186187165
0.015943111	-0.692698452	-0.449398744	-0.714019914	-0.384193969	-0.846563603	1.288872288	3.344365642	1.437693641
-0.404255854	-0.262847876	-0.654677984	-1.047867705	-0.563662983	-0.759805506	2.298888073	2.925937176	0.468292658
-1.146352986	-1.192156675	-1.381323473	-0.918450583	-0.659853124	-0.843442117	3.015738575	3.030947895	2.094892488
-0.560284133	-0.354763576	-0.871787248	0.012615305	-0.705714572	-0.530370724	1.026614612	3.237238928	1.746451407
-0.752831835	-0.886793138	-0.940599582	-0.391669813	-0.446952248	0.144145039	1.872309316	3.162391006	1.564783267
-0.28548107	-1.49886225	-0.855336679	-1.33337576	-1.244236582	-1.093107201	2.963392216	2.924416172	2.422591155
-1.460076375	-1.406269931	-1.460076375	0.324846327	-0.995408108	-0.766756696	2.273494896	2.815526705	2.674719557
0.014929892	-0.787088551	-0.939197877	-1.173413183	-0.684242336	-0.879879303	0.949016333	2.788865612	1.381986369
-0.214295778	-0.654476734	-0.922796488	-1.004061172	-0.953256801	-0.905790062	1.44782939	3.278731114	1.928116531
-0.201489771	-0.706621482	-1.207962174	-1.354673922	-0.941647148	-0.47681642	1.683356369	2.637410628	1.165464379
-1.163922019	-0.320140039	-0.708688643	-1.2379226	-0.986550268	-0.187332395	1.960798904	2.761210343	1.882546716
-0.82257271	-0.874543334	-0.71353506	-1.437733245	-0.652552706	-0.605190643	0.872197193	3.26212256	1.326662526
-0.665826356	-1.162229048	-0.642438429	-0.810420836	-0.731559321	0.379070182	1.337408823	3.121025333	1.174969651
-0.902644993	-1.136533053	-1.006920315	-0.366761314	0.109570957	-0.168626683	1.727999792	2.591686057	1.234747001
-0.121126593	-0.473956247	-0.393805966	-1.107239582	-0.027702288	-0.445285185	1.551313949	3.055072687	0.962729223
-0.658309515	-0.999248327	-0.324364184	-1.306956471	-0.718921136	-0.247447691	2.327270194	3.045332638	1.973309769
-0.80690651	-0.80690651	-0.80690651	-0.371811359	-0.80690651	-0.80690651	1.013578833	2.85484309	2.537921987
-0.725756416	-1.583419771	-1.583419771	0.398067597	-1.583419771	0.383748836	1.973254876	3.234612704	1.034818885
-0.570667032	-0.293397503	-0.85893848	-1.414740773	-1.092812678	-1.101396524	1.959414564	3.306397045	0.924807316
-0.204216618	-1.034403483	-0.597868622	-0.329948867	-1.156488773	0.038030046	1.234510824	3.04590485	2.096290342
-0.150330675	-0.192951352	-0.291269047	-1.033898256	-1.360745566	-0.143838683	1.47276867	2.635828417	1.064436491
-0.406019744	-0.194093975	-1.016710806	-0.549602846	-0.535434714	-0.24168592	1.529227928	3.171478891	1.242841187
-0.608213952	-0.608213952	-1.114784102	-0.069035227	-0.009122325	-0.245506695	1.660775648	3.055083781	1.049184385
-0.550273155	-0.714033109	-0.228844183	-0.771789873	-0.151385147	-0.378408758	1.646470543	3.053099459	1.095164222
-1.157458429	-1.301646622	-1.623998354	-0.901083022	-1.275621159	-0.741463029	3.39059158	3.160170444	2.450508591
-0.997251346	-1.02945836	-1.28767575	-0.001385992	-1.28767575	-1.093588698	1.782713578	3.238893911	3.675428405
-0.178647378	-0.234079769	-0.614809217	-0.245203147	-0.614809217	-0.614809217	1.37323918	3.305102871	0.824015896
-0.227862441	-1.001858311	-0.320947564	-1.409983474	0.076033563	-0.754557856	1.547531532	2.842010809	1.249633743
-0.538910129	-1.176543998	-1.176543998	-0.866450465	-1.176543998	-0.223465046	2.053043925	3.206399871	0.882926502
-0.920224297	-0.920224297	-0.920224297	-0.920224297	-0.920224297	0.185453781	1.928974355	2.673129474	0.813563872
-0.458028586	-0.458028586	-0.458028586	-0.458028586	0.253906771	-0.458028586	1.620239661	3.236183022	0.798134745
-0.883667937	-0.56289446	-0.883667937	-0.883667937	-0.883667937	-0.009461151	1.303783117	3.171527717	2.631716524
-0.199647591	-1.229010774	-1.001247789	-0.789559057	-0.274882382	-0.465160446	1.21248086	2.968822663	0.850849732
0.001413543	-0.844783619	-1.256161902	-0.744854966	-0.827303415	-0.888661039	1.570784626	2.430431519	1.559135254
-0.052351717	-1.194674297	-0.544943105	-0.965345235	-0.076166502	-0.555033644	1.154619099	2.654156037	1.579739364
-0.711426917	-0.789342549	-0.83546576	-0.612464786	-0.064976991	-0.136665729	1.675929646	2.533905487	0.9405076
-1.118028057	-0.949156004	-1.093989491	-0.059602525	-0.003065307	-0.002422251	1.943671393	3.118306704	1.400898945
-0.634628322	0.047944975	-0.634628322	-0.634628322	-0.634628322	0.058691357	1.027577177	2.634628322	2.038928102
-0.939091789	-1.02994522	-0.154951581	-0.827057386	-0.762109827	-0.422082317	1.874635215	2.865357402	1.395245502
-0.491599449	-0.660312146	-1.651332397	-0.639601197	-0.751305643	-0.063388958	1.86939982	2.663037357	1.725102812
-0.391998034	-0.97304684	-0.704741589	-0.407254146	-0.495876159	-0.604336211	1.215577705	2.718094141	1.643581133
-0.571185897	-0.388762411	-0.611097019	-0.398508035	-0.282144567	-0.289573664	1.059621297	2.427632696	1.054017601
-0.188013108	-0.635793427	-0.690024983	-0.576674862	-0.202320465	-0.683036673	0.869020143	2.501062529	1.22975463
-0.33490212	-0.897199459	-0.41390563	-1.368682105	-0.640558019	-1.037430072	1.568704558	3.153471351	1.970501495
-0.987937789	-0.884244196	-0.582660415	0.224473591	-0.782169683	0.223147381	1.206242343	3.121461923	1.461686846
-0.640765236	-0.451539679	-0.589762333	-0.010967087	-0.29714207	-0.474488012	1.139003854	3.191303906	1.516964468
-0.315107212	-0.568518325	-0.322674959	-1.182959882	-0.884423929	-0.690366123	0.982619298	2.932106958	1.41910975
-0.463219387	-1.283717495	-0.991549267	-0.526716479	-0.723512056	-0.321496254	1.297012157	2.850901343	1.162297439
-0.535946726	-1.306217663	-0.441294841	0.00419472	-0.267231895	-0.109735732	1.623196977	2.545537424	1.287588904
-1.126025861	-1.126025861	-0.522903991	-1.126025861	-1.126025861	-0.135433989	1.942816893	3.275604605	1.944019927
-0.01331261	-0.930689591	-0.784330487	-0.664741385	-0.443518979	-0.620549422	1.196101625	3.012799797	1.248241052
-0.420641039	-0.165967902	-0.458654364	-0.512159694	-0.270685774	-0.388042724	0.801751383	2.613623526	0.800776589
-0.153930881	-0.877202543	-1.239155407	-0.564879999	-1.187542664	-0.335886415	1.447515166	3.26512807	1.645954673
-0.860645915	-0.96874275	-0.776367528	-0.434800415	-0.709911829	-0.786478442	1.124085553	3.083844615	2.004476865
-0.361163974	-0.418345634	-0.248564665	-0.214402965	-0.337684907	-0.118623334	1.212542072	3.295409528	0.781652934
-0.888519218	-1.087216257	-0.630677286	0.050185869	-0.248419212	-0.526057124	0.82512919	2.643657881	1.861916158
-0.661455258	-1.074522054	-0.129868231	-0.484691272	-0.085536125	-0.271030634	1.192235256	3.3294305	1.185437818
-0.623164759	-1.099720052	-1.244998899	-1.10473159	-0.971741938	-0.353777328	2.259676334	2.856019904	1.952856242
-0.194107763	-0.581394825	-0.256627514	-0.794592535	-1.529247969	0.434226155	0.794698487	3.142045404	0.985000561
-0.332961609	-0.332961609	-0.332961609	-0.332961609	-0.332961609	-0.332961609	1.745306638	3.332961609	0.854246254
-1.259639139	-0.514021674	-0.899179806	-0.293324637	-0.845732012	0.254723098	1.516517634	3.027214841	1.013441695
-0.175074282	-0.821032474	-1.157767369	-1.180347741	-0.69724503	-0.048767531	1.119631573	2.89872383	1.061879023
-0.282237984	-1.036622965	-0.811784883	-0.939405803	-0.69780722	-0.6492567	1.560658306	2.613530491	1.242926758
-0.969604271	-0.901741798	-0.920567468	-0.718185367	0.050314067	-0.102146035	1.235182991	2.838757868	1.487990013
-0.38685723	-1.124647928	-0.753551649	-0.712346963	-0.219959921	-0.226587934	1.496917822	2.623498855	1.303534947
-0.695829275	-0.589118684	-0.780371016	-0.521561196	-0.211357558	-0.417126149	1.672695965	2.494008364	1.048659549
-1.350415343	-1.708846772	-0.801355178	-0.353495675	-1.490646828	-1.198676021	2.314422444	2.765163312	2.823850061
-1.178204211	-0.723922626	-1.674708973	-0.774183209	-0.067836379	-0.289258387	2.052655519	2.940891621	1.761369105
-0.09775739	-1.154887761	-0.679373655	-1.227509499	-0.32893146	0.023008464	1.202732251	1.964017203	1.298701848
-0.665170101	-0.97525069	-0.08671112	-0.285993105	-0.286824869	-0.369874446	0.485155723	1.740781447	1.443887161
-0.136203407	-1.203038952	-0.66424929	-0.724212289	0.130026683	-0.558516001	0.828968369	1.410788998	1.186145988
-0.917268548	-0.954662724	-0.450639093	-1.381526659	-0.955349885	-1.062952578	2.466553704	1.859183022	2.396661762
-0.101294386	-1.327048542	-0.926150604	-0.592335986	-1.101176198	-1.053318612	1.965950151	1.76969117	2.163094236
-0.118536079	-1.269056916	-0.761843196	-1.314797461	-0.672008533	-0.473326052	1.783865731	1.375409892	1.213220456
-0.549728934	-0.549728934	-0.549728934	-0.549728934	-0.549728934	-0.549728934	2.072732933	1.549728934	1.775369604
-0.142641633	-0.412040025	-0.69124737	-1.046880338	-1.247498574	-0.923068324	1.973987073	1.070498469	1.133607456
-0.468685693	-0.981671028	-0.981671028	-0.981671028	-0.981671028	-0.40249088	1.522186505	1.465114534	1.810559646

AT1G78720.1	SC61A_DICDI transport Sec61 subunit alpha OS=Dictyostelium discoideum GN=sec61a PE=3 SV=1
AT4G22690.1	C75A1_PETHYFlavonoid 3 ,5 -hydroxylase 1 OS=Petunia hybrida GN=CYP75A1 PE=2 SV=1
AT1G23020.2	FRO3_ARATHFerric reduction oxidase mitochondrial OS=Arabidopsis thaliana GN=FRO3 PE=2 SV=1
AT3G01290.1	HIR3_ARATHHypersensitive-induced response 3 OS=Arabidopsis thaliana GN=HIR3 PE=1 SV=1
AT1G08980.1	AMI1_ARATHAmidase 1 OS=Arabidopsis thaliana GN=AMI1 PE=1 SV=1
AT1G10340.1	LCTA_LATTRAlpha-latrocrustotoxin-Lt1a (Fragment) OS=Latrodectus tredecimguttatus PE=2 SV=2
AT2G06850.1	XTH4_ARATHXyloglucan endotransglucosylase hydrolase 4 OS=Arabidopsis thaliana GN=XTH4 PE=1 SV=1
AT3G49790.1	P2A10_ARATH PHLOEM PROTEIN 2-LIKE A10 OS=Arabidopsis thaliana GN=PP2A10 PE=2 SV=1
AT1G70100.3	TPX2 (targeting for Xklp2) family [Medicago truncatula]
AT5G35790.1	G6PD1_ARATHGlucose-6-phosphate 1-dehydrogenase chloroplastic OS=Arabidopsis thaliana GN=APG1 PE=2 SV=2
AT5G14470.1	GLAK2_ARATHProbable glucuronokinase 2 OS=Arabidopsis thaliana GN=GLCAK2 PE=2 SV=1
AT1G58190.1	FLS2_ARATHLRR receptor-like serine threonine- kinase FLS2 OS=Arabidopsis thaliana GN=FLS2 PE=1 SV=1
AT1G80370.1	CCA24_ARATHCyclin-A2-4 OS=Arabidopsis thaliana GN=CYCA2-4 PE=2 SV=1
AT5G02200.2	PREDICTED: uncharacterized protein LOC103423426 isoform X2 [Malus domestica]
AT1G49320.1	BURP3_ORYSJBURP domain-containing 3 OS=Oryza sativa japonica GN=BURP3 PE=2 SV=1
AT5G15210.1	ZHD8_ARATHZinc-finger homeodomain 8 OS=Arabidopsis thaliana GN=ZHD8 PE=1 SV=1
AT1G12780.1	UGE1_ARATHBifunctional UDP-glucose 4-epimerase and UDP-xylose 4-epimerase 1 OS=Arabidopsis thaliana GN=UGE1 PE=1 SV=2
AT4G24230.6	ACBP3_ARATHAcyl- -binding domain-containing 3 OS=Arabidopsis thaliana GN=ACBP3 PE=1 SV=1
AT1G55810.1	UKL3_ARATHUridine kinase 3 OS=Arabidopsis thaliana GN=UKL3 PE=2 SV=1
AT5G15580.1	LNG1_ARATH LONGIFOLIA 1 OS=Arabidopsis thaliana GN=LNG1 PE=1 SV=1
AT1G79700.1	AP2L3_ARATHAP2-like ethylene-responsive transcription factor At1g79700 OS=Arabidopsis thaliana GN=At1g79700 PE=2 SV=1
AT5G21930.1	HMA8_ARATHCopper-transporting ATPase chloroplastic OS=Arabidopsis thaliana GN=PAA2 PE=2 SV=1
AT1G24530.1	MHCKB_DICDIMyosin heavy chain kinase B OS=Dictyostelium discoideum GN=mhkB PE=2 SV=1
AT4G03510.2	RMA1_ARATHE3 ubiquitin- ligase RMA1 OS=Arabidopsis thaliana GN=RMA1 PE=1 SV=1
AT5G05420.1	FK153_ARATHPeptidyl-prolyl cis-trans isomerase FKBP15-3 OS=Arabidopsis thaliana GN=FKBP15-3 PE=2 SV=1
AT3G52060.1	core-2 I-branching enzyme [Medicago truncatula]
AT1G80920.1	DNAJ8_ARATHChaperone dnaJ chloroplastic OS=Arabidopsis thaliana GN=ATJ8 PE=2 SV=1
AT5G51550.1	EXOL3_ARATH EXORDIUM-like 3 OS=Arabidopsis thaliana GN=EXL3 PE=2 SV=1
AT5G04310.1	PLY13_ARATHProbable pectate lyase 13 OS=Arabidopsis thaliana GN=PMR6 PE=1 SV=1
AT5G21170.1	KINB1_ARATHSNF1-related kinase regulatory subunit beta-1 OS=Arabidopsis thaliana GN=KINB1 PE=1 SV=1
AT3G15570.1	RPT3_ARATHRoot phototropism 3 OS=Arabidopsis thaliana GN=RPT3 PE=1 SV=2
AT1G52100.1	JAL11_ARATHJacalin-related lectin 11 OS=Arabidopsis thaliana GN=JAL11 PE=2 SV=1
AT1G03620.1	ELMOA_DICDIELMO domain-containing A OS=Dictyostelium discoideum GN=elmoA PE=1 SV=1
AT5G45310.1	PREDICTED: uncharacterized protein LOC103409418 [Malus domestica]
AT3G01480.2	CYP38_ARATHPeptidyl-prolyl cis-trans isomerase chloroplastic OS=Arabidopsis thaliana GN=CYP38 PE=1 SV=1
AT1G72070.1	DNAJ_PROMHChaperone OS=Proteus mirabilis (strain HI4320) GN=dnaJ PE=3 SV=1
AT3G16450.2	JAL33_ARATHJacalin-related lectin 33 OS=Arabidopsis thaliana GN=JAL33 PE=1 SV=1
AT3G06080.2	TBL10_ARATH trichome birefringence-like 10 OS=Arabidopsis thaliana GN=TBL10 PE=2 SV=1
AT1G03610.1	plant (DUF789)
AT5G53980.1	ATB52_ARATHHomeobox-leucine zipper ATHB-52 OS=Arabidopsis thaliana GN=ATHB-52 PE=2 SV=1
AT5G23350.1	GEML6_ARATHGEM 6 OS=Arabidopsis thaliana GN=At5g23350 PE=2 SV=2
AT3G19550.1	PREDICTED: uncharacterized protein LOC103423045
AT4G18205.1	PUP21_ARATHProbable purine permease 21 OS=Arabidopsis thaliana GN=PUP21 PE=2 SV=1
AT1G33790.1	JAL4_ARATHJacalin-related lectin 4 OS=Arabidopsis thaliana GN=JAL4 PE=2 SV=1
AT2G41990.1	late embryogenesis abundant [Medicago truncatula]
AT2G37025.1	TBP1_ORYSJTelomere-binding 1 OS=Oryza sativa japonica GN=TBP1 PE=1 SV=2
AT4G05070.1	unnamed protein product
AT3G47570.1	Y3475_ARATHProbable LRR receptor-like serine threonine- kinase At3g47570 OS=Arabidopsis thaliana GN=At3g47570 PE=2 SV=1
AT3G03890.2	pyridoxamine 5 -phosphate oxidase
AT4G27150.1	2SS2_ARATH2S seed storage 2 OS=Arabidopsis thaliana GN=AT2S2 PE=2 SV=1
AT1G80420.4	XRCC1_ARATHDNA-repair XRCC1 OS=Arabidopsis thaliana GN=XRCC1 PE=1 SV=1
AT5G18170.1	DHE1_ARATHGlutamate dehydrogenase 1 OS=Arabidopsis thaliana GN=GDH1 PE=2 SV=1
AT4G31410.1	DUF1644 family [Medicago truncatula]
AT5G23700.1	SCD2_ARATHCoiled-coil domain-containing SCD2 OS=Arabidopsis thaliana GN=SCD2 PE=1 SV=1
AT3G26220.1	C71B3_ARATHCytochrome P450 71B3 OS=Arabidopsis thaliana GN=CYP71B3 PE=2 SV=2
AT3G23630.1	IPT7_ARATHAdenylate isopentenyltransferase mitochondrial OS=Arabidopsis thaliana GN=IPT7 PE=2 SV=2
AT5G57640.1	GCK domain [Medicago truncatula]
AT2G22300.2	CMTA3_ARATHCalmodulin-binding transcription activator 3 OS=Arabidopsis thaliana GN=CMTA3 PE=1 SV=1
AT5G21170.2	KINB1_ARATHSNF1-related kinase regulatory subunit beta-1 OS=Arabidopsis thaliana GN=KINB1 PE=1 SV=1
AT3G15510.1	NAC56_ARATHNAC transcription factor 56 OS=Arabidopsis thaliana GN=NAC056 PE=2 SV=1
AT1G19540.1	IFRH_ARATHIsoflavone reductase homolog P3 OS=Arabidopsis thaliana GN=At1g75280 PE=2 SV=1
AT2G17780.3	MCAC2_ARATH MID1-COMPLEMENTING ACTIVITY 2 OS=Arabidopsis thaliana GN=MCA2 PE=2 SV=1
AT4G01460.1	BH057_ARATHTranscription factor bHLH57 OS=Arabidopsis thaliana GN=BHLH57 PE=2 SV=1
AT4G19530.1	RPS4W_ARATHDisease resistance RPS4 OS=Arabidopsis thaliana GN=RPS4 PE=2 SV=1
AT1G13250.1	GATL3_ARATHProbable galacturonosyltransferase-like 3 OS=Arabidopsis thaliana GN=GATL3 PE=2 SV=1
AT1G68910.2	WIT2_ARATHWPP domain-interacting tail-anchored 2 OS=Arabidopsis thaliana GN=WIT2 PE=1 SV=1
AT3G19850.1	Y3985_ARATHBTB POZ domain-containing At3g19850 OS=Arabidopsis thaliana GN=At3g19850 PE=2 SV=1
AT1G42560.1	MLO9_ARATHMLO 9 OS=Arabidopsis thaliana GN=MLO9 PE=2 SV=2
AT3G26200.1	C71BM_ARATHCytochrome P450 71B22 OS=Arabidopsis thaliana GN=CYP71B22 PE=2 SV=1
AT2G42170.1	ACT5_ARATH actin-5 OS=Arabidopsis thaliana GN=ACT5 PE=5 SV=1
AT1G75380.3	BBD1_ARATHBifunctional nuclease 1 OS=Arabidopsis thaliana GN=BBD1 PE=2 SV=1
AT1G23840.1	At1g23840 F5O8_37
AT3G45300.1	IVD_ARATHIsovaleryl- mitochondrial OS=Arabidopsis thaliana GN=IVD PE=1 SV=2
AT1G72920.1	TIR_ARATHToll interleukin-1 receptor OS=Arabidopsis thaliana GN=TIR PE=1 SV=1
AT5G51790.1	BH120_ARATHTranscription factor bHLH120 OS=Arabidopsis thaliana GN=BHLH120 PE=2 SV=2
AT2G03750.1	SOT11_ARATHCytosolic sulfotransferase 11 OS=Arabidopsis thaliana GN=SOT11 PE=2 SV=1
AT3G02550.1	LBD41_ARATHLOB domain-containing 41 OS=Arabidopsis thaliana GN=LBD41 PE=2 SV=1
AT2G14080.1	RPP1_ARATHProbable disease resistance RPP1 OS=Arabidopsis thaliana GN=RPP1 PE=2 SV=1
AT4G22540.2	ORP2A_ARATHOxysterol-binding -related 2A OS=Arabidopsis thaliana GN=ORP2A PE=2 SV=1
AT1G07780.1	PAI1_ARATHN-(5 -phosphoribosyl)anthranilate isomerase chloroplastic OS=Arabidopsis thaliana GN=PAI1 PE=2 SV=1
AT5G46390.1	CTPA1_ARATHCarboxyl-terminal-processing peptidase chloroplastic OS=Arabidopsis thaliana GN=CTPA1 PE=1 SV=1
AT1G76410.1	ATL8_ARATHRING-H2 finger ATL8 OS=Arabidopsis thaliana GN=ATL8 PE=2 SV=2
AT5G16370.1	AAE5_ARATHProbable acyl-activating enzyme peroxisomal OS=Arabidopsis thaliana GN=AAE5 PE=2 SV=1
AT1G55810.3	UKL3_ARATHUridine kinase 3 OS=Arabidopsis thaliana GN=UKL3 PE=2 SV=1
AT5G52100.1	DAPB3_ARATHDihydrodipicolinate reductase chloroplastic OS=Arabidopsis thaliana GN=DAPB3 PE=2 SV=1
AT5G19230.1	UGPI1_ARATHUncharacterized GPI-anchored At5g19230 OS=Arabidopsis thaliana GN=At5g19230 PE=1 SV=1
AT3G47800.1	GALM_BOVINAldose 1-epimerase OS=Bos taurus GN=GALM PE=2 SV=1
AT3G56410.2	Y5519_ARATHUncharacterized protein At5g05190 OS=Arabidopsis thaliana GN=Y-1 PE=1 SV=1
AT1G18280.1	VAS_ARATHLipid transfer VAS OS=Arabidopsis thaliana GN=VAS PE=2 SV=1
AT1G61810.1	BGL45_ARATHBeta-glucosidase 45 OS=Arabidopsis thaliana GN=BGLU45 PE=1 SV=1
AT1G53160.1	SPL4_ARATHSquamosa promoter-binding 4 OS=Arabidopsis thaliana GN=SPL4 PE=1 SV=1
AT1G66040.1	ORTH4_ARATH E3 ubiquitin- ligase ORTHRUS 4 OS=Arabidopsis thaliana GN=ORTH4 PE=3 SV=1
AT5G45830.1	HBP1C_WHEATTranscription factor HBP-1b(c1) (Fragment) OS=Triticum aestivum PE=1 SV=2
AT4G18195.1	PUP8_ARATHProbable purine permease 8 OS=Arabidopsis thaliana GN=PUP8 PE=2 SV=1
AT3G15351.3	hypothetical protein MTR_4g093680 [Medicago truncatula]
AT3G11550.1	CASP2_ARATHCasparian strip membrane 2 OS=Arabidopsis thaliana GN=CASP2 PE=1 SV=1
AT4G12690.1	phosphatase [Medicago truncatula]
AT1G22330.1	ARP1_ARATHProbable RNA-binding ARP1 OS=Arabidopsis thaliana GN=ARP1 PE=2 SV=1
AT3G07350.1	DUF506 family [Medicago truncatula]
AT1G80180.1	Y1540_ARATHUncharacterized protein At1g15400 OS=Arabidopsis thaliana GN=At1g15400 PE=1 SV=2
AT1G21920.1	PI5K1_ARATHPhosphatidylinositol 4-phosphate 5-kinase 1 OS=Arabidopsis thaliana GN=PIP5K1 PE=1 SV=1
AT3G51330.1	ASPL1_ARATHAspartic ase 1 OS=Arabidopsis thaliana GN=At5g10080 PE=1 SV=1
AT2G39870.1	homeobox OTX1-like
AT1G15040.2	NTPR_ENTHA OS=Enterococcus hirae (strain ATCC 9790 DSM 20160 JCM 8729 LMG 6399 NBRC 3181 NCIMB 6459 NCDO 1258) GN=ntpR PE=3 SV=2
AT1G58180.2	BCA6_ARATHBeta carbonic anhydrase mitochondrial OS=Arabidopsis thaliana GN=BCA6 PE=2 SV=1
AT5G63180.1	PLY22_ARATHProbable pectate lyase 22 OS=Arabidopsis thaliana GN=At5g63180 PE=2 SV=1
AT2G30010.1	TBL45_ARATH trichome birefringence-like 45 OS=Arabidopsis thaliana GN=TBL45 PE=2 SV=1

-0.276080999	-0.151183572	-0.233645732	-0.351482223	-0.387973879	-0.04269278[illegible]	1.367217765	1.364890266	1.440731689
-1.059968289	-1.343092573	0.228168035	-1.025803373	-0.793290912	-0.749408206	1.580248426	1.733522886	2.429624007
-0.462568692	-1.132480089	-0.771398272	-0.501872501	0.210917388	-1.041685815	1.547460911	0.912741063	1.238886008
-0.239572899	-0.116516596	-0.149483538	-1.142736219	-1.393056296	-0.630429433	1.589365119	2.095087879	2.177517741
-0.082417795	-0.822583239	-0.637337666	-0.713392088	0.208316631	-0.487378626	1.236071632	1.433170573	0.865550579
-0.667880554	-0.667880554	-0.667880554	-0.667880554	-0.351734811	-0.224273902	0.589130065	1.291889602	2.366511261
-0.328341372	-0.624186495	-0.327110252	-1.722151523	-0.906715003	-0.75135373	1.332882522	1.390791114	1.279501995
-0.665715189	-1.070382992	-1.020555238	-1.04995511	-0.456805718	-0.617814269	1.845005705	1.283409691	1.752813119
-1.417437366	-0.509008716	-1.417437366	-0.744785736	-0.413835129	-1.223350314	2.53926984	1.245907254	1.940677532
-0.183947319	-1.161535128	-1.718004132	-0.654559261	-0.514582735	-0.233275505	1.519390861	1.975556684	0.970956534
-0.506052916	-0.582848366	-1.319684449	-0.903514218	-1.229329672	-0.703125706	3.060805762	2.071348027	2.255097593
-0.312641888	-0.482470611	-0.355640589	-0.591320035	-0.346937713	-0.212233519	1.476441716	1.555331533	1.380134172
-0.445612746	-0.470087247	-0.673594849	0.223329808	-0.247330094	-0.466686668	0.790462507	1.430377079	0.85914221
-0.477926151	-1.118531989	-0.794297426	-0.307060958	-0.020247193	-1.118531989	1.116582332	1.78373458	0.980426491
-0.172350652	-0.659460086	-0.339300843	-0.645775884	-0.097929725	-0.849161888	0.684107378	1.663038298	1.053241316
-0.451407192	-0.859892644	-0.419781607	-0.979549203	-0.974793865	-0.735106683	1.03575805	1.317357975	1.164600783
-0.22903964	-0.560977198	-1.571921644	-1.203218831	-1.164326323	-0.326863494	2.490828551	1.985202793	1.580315786
-0.641350538	-0.376651438	-1.426216846	-0.622908757	-0.452279143	-0.607486214	2.209597902	1.363637793	1.55365724
-0.252240203	-0.667548613	-0.445100899	-0.59840207	-0.927690776	-0.372261895	1.898008812	1.2750068	1.090228843
-0.476171746	-0.363877882	-0.781786417	0.133721364	0.003411657	-0.335946244	0.542056336	1.436769047	0.841823887
-0.881520854	-0.881520854	-0.881520854	-0.881520854	-0.881520854	0.675766688	1.839414227	1.804195082	1.088228274
-0.112613336	-1.097668533	-1.841971595	-1.072153365	-0.974031697	-0.451074163	2.045853581	1.440549676	1.83788276
-0.202986835	-0.630032567	0.436537335	-1.266104171	-1.044438577	-0.53778473	1.23933799	1.239184221	2.244655777
-0.84823862	-0.400325872	-1.550708965	-0.793267266	-0.849810468	-0.429017877	2.228929923	1.482459852	1.59419316
-0.183536827	-1.305536288	-1.504477736	-0.546845024	0.008633403	-0.51648359	1.432883503	1.487767994	1.127594566
-0.400150301	-0.923702139	-0.969573397	-0.797008074	-0.052643373	-0.371139958	1.485110963	1.752055153	1.277051126
-0.299437746	-0.779504693	-1.892324232	-0.863997849	-0.638641842	-0.455354912	1.924465438	1.186093849	1.818701987
-0.228949981	-1.051024287	-0.537546202	-1.77087992	-1.041554508	-0.739086018	1.832280245	1.219734723	1.859125985
-0.573417814	-0.651309473	-0.001819883	-0.310549703	-0.381271679	-0.314713328	1.24755557	2.11344209	1.098968402
-0.399562504	-1.085566439	-1.693382638	-1.029379832	-0.795541519	-0.24377802	2.368135006	1.56451764	1.87343077
-0.231078352	-0.601204949	-0.830191874	-0.687290608	-1.160254639	-0.789316236	1.323687725	1.635181593	1.34046734
-0.394633166	-1.304598232	-1.069483912	-0.326768815	-1.178947131	0.169188679	2.229338823	1.664045524	2.211858231
-0.36564979	-0.603212672	-1.745545754	-0.535708914	-0.761598986	-0.997506918	2.039936193	1.215044952	1.02294231
-1.218635388	-1.068033649	-1.059645864	0.251649442	0.462400178	-0.532029096	1.998494918	1.591828792	1.573970665
-0.085764883	-1.196118889	-1.576848337	-0.508521476	-0.322254294	-0.527914692	1.477305535	1.624315003	1.115802033
0.093063783	-0.803056099	-0.584414524	-1.079111576	-0.391114115	-1.227137355	1.897561211	1.370565635	1.723643039
-1.223247921	-0.272741854	-1.04030112	0.08567615	-0.233055539	-0.656793909	1.292150406	1.330980413	1.717333375
-0.739437888	-1.152096851	-0.822253498	-0.852093387	-0.287414073	-0.223233296	1.718519336	0.927132144	1.430877513
-0.970382573	-0.951262739	-0.331988058	-0.402335147	0.406159777	-0.113803846	1.347163448	1.31973252	0.696716618
-0.245638753	-0.984622707	-0.625101537	-0.744613743	-0.984622707	-0.739735648	0.913779153	2.005061296	1.405494646
-0.285281484	-1.010983438	-1.045258429	-0.831096247	-1.050688224	-0.401185471	1.526638066	1.361694821	1.736160407
-0.694755568	-0.725755795	-0.928643629	0.364138121	-0.101637619	-0.563511035	0.895513215	2.056856802	1.697795508
-0.934019925	-0.961307832	0.21811396	-0.912383421	-0.977841919	-0.730245609	1.641794147	1.214676749	2.441213851
-0.568721828	-1.233200347	-0.483141571	-0.671719279	-0.537088137	-1.15325219	1.836830106	1.098830887	1.71146236
-0.909231256	-0.985225581	-0.739586689	-0.308373433	-0.491462373	-0.046818904	1.611365092	1.766209434	1.103123709
-0.198768434	-0.153202276	-0.595497375	-0.832747755	-1.422543409	0.218340416	1.658068473	1.459607225	0.866743134
-0.261501292	-0.660427443	-0.973115686	-0.133071004	-0.328708637	-0.468195923	1.001636286	1.487339325	1.251647182
-0.572412268	-0.812435323	-0.31227121	-0.498465883	-0.747359121	-0.592605416	1.083919916	1.857854906	1.593774399
-0.668952221	-0.962369824	-0.857223391	-1.225037645	-1.225037645	-0.430938819	2.311091089	1.232993785	1.825474671
-0.48252539	-0.48252539	-0.48252539	-0.48252539	-0.48252539	-0.48252539	1.545336385	1.269644824	1.619460782
-0.294047401	-0.221842892	-0.846837745	-0.118896591	-0.037759828	-0.854538004	0.833289769	1.543440754	0.997191938
-1.282696593	-1.629600396	-0.859349352	-0.547398707	-1.144639537	-0.696345807	2.394603836	1.424654871	2.340771686
-0.21110527	-0.684199538	-0.899493873	-0.176030249	0.367996673	-0.489872059	0.931007268	1.286566399	0.87513065
-0.419112243	-0.603969018	-0.569267927	-0.573229563	-0.447919092	-0.585180166	2.066017458	1.768874334	1.209547985
-0.275018068	-0.824481888	-1.258568841	-1.076040533	-0.941386086	-0.669476306	1.733513565	1.402108702	1.909349456
-0.592048626	-0.587337781	-0.539918284	-0.692174238	-0.375566475	-0.649812273	1.723969198	1.626670193	2.339558673
-0.563589495	-0.670951055	-0.891088316	-0.514164278	-0.428901674	-0.466376379	1.720324335	1.08196753	1.732779333
-0.436438745	-0.177716916	-1.015163903	0.202776027	-0.093166415	-0.576131725	0.657237031	1.501943274	0.936661371
-1.129302002	-0.287731365	-0.380411459	-0.941534255	-0.396815709	-0.82479096	2.101439001	1.003768267	1.855378482
-0.416560644	-0.376763693	-0.418549746	-0.619636331	-0.96783008	-0.534989346	1.542564504	0.898397264	1.70192561
-0.587186926	-0.594399632	-0.860140253	-0.302009309	-0.877632289	-0.308183477	2.282225671	1.03055848	1.216767733
-0.12729955	-0.678858445	-1.632682399	-0.543862365	-1.139035065	-0.479525611	2.083210972	1.070418301	1.447634162
-0.40518015	-0.872963742	-0.933476827	-0.678514556	-1.32754805	-0.665948579	1.348111946	1.86904768	0.856111977
-0.640013211	-0.732956956	-0.231756411	-0.822556414	-1.059382992	-0.476267969	1.4460684	1.239917631	1.276947923
-0.405534306	-0.685139386	-0.402018109	-0.304262904	-0.476240512	-0.412592515	0.994640861	1.495382583	1.195764288
-0.736314612	-0.013848587	-0.736314612	0.670222058	-0.736314612	-0.736314612	0.878630755	1.142606868	1.267647354
-0.694804001	-0.518676542	-0.761502783	-1.26292849	-0.981746738	-0.545266526	1.28243519	2.032971192	1.449518698
-0.62984159	-0.598193219	-0.682257484	-0.193742475	-0.606382617	-0.437370425	1.878946437	1.19066089	1.459502263
-0.69184394	-0.598383045	-0.308796555	-0.944231102	-0.834606611	-0.523065134	1.618413019	1.460159153	1.822354214
-0.22481195	-1.159950077	-1.2351655	-0.989150706	-0.376090363	-1.145097681	2.059336851	1.524511905	1.096793621
-1.017775445	-0.970497749	-0.564383803	-0.39105619	-0.078081252	-0.366330574	1.453060315	1.540301946	1.394762751
-0.92383265	-0.349226943	-0.397849839	-0.483097126	-0.434320586	-0.347668676	0.78610164	1.515191154	1.634703026
-0.432630579	-0.957322722	-0.843269404	-0.901464091	-0.367565183	-0.616438384	2.108121143	0.974836062	1.313085614
-0.329661321	-0.297824574	-0.169798523	-0.540867504	-0.670106349	-0.498698579	1.226223548	1.738606583	2.019339886
-0.39955582	-0.614430846	-0.523282958	-1.043618833	-0.754112216	-1.150003781	1.847093995	1.570633643	2.067276815
-0.500813889	-1.220280573	-0.47052084	-0.719230939	-0.990586948	-0.439721753	1.332568852	1.728159542	1.280426547
0.026253986	-1.251638705	-0.922838803	0.575443522	-1.321549765	-0.656929659	1.740607215	1.256029772	1.554622438
-0.674003309	-0.275349077	-0.791146749	-0.31442898	-0.409593908	-0.063066286	0.818728005	0.962017685	1.04684262
-0.222181339	-0.479030819	-1.178512671	-0.72138521	-1.286319661	-0.437603411	1.507625244	1.265264406	1.107780783
-0.129360915	-0.776445127	-0.776445127	-0.776445127	-0.756387475	0.048119085	1.121956733	1.203214425	1.841793528
-0.681361282	-0.681361282	-0.681361282	-0.142326579	0.398614095	-0.681361282	1.824529648	1.140756993	0.503870972
-1.52369408	-1.653690799	-1.078943755	-0.463650752	-0.492739157	-0.824946673	3.009469021	1.512424765	2.51577143
-0.13018635	-0.691432781	0.095676298	-1.488820253	-0.654906905	-0.830847493	1.609780458	0.865412153	1.904195811
-0.94801234	-0.948863236	-1.710148509	0.408211217	0.582338782	-0.168138154	2.188350095	1.016382797	0.612644943
-0.290427136	-1.132203684	-0.911666819	-0.536847914	-0.464512912	-0.175138954	1.227675704	1.445807549	0.837314163
-0.924768743	-1.162943461	-0.448283232	-0.762798162	-1.658466359	-0.562808988	2.004544672	0.651442784	2.864081489
-0.480445463	-0.210843146	-0.862638327	-0.727291255	-0.097379957	-0.736458065	1.150408688	1.030122998	1.033879598
-0.340974199	-0.340974199	-0.340974199	-0.340974199	-0.340974199	-0.340974199	1.378428313	1.340974199	1.008391079
-0.356455382	-0.356455382	-0.356455382	-0.356455382	-0.356455382	-0.136125428	1.201322289	1.154821521	0.871901572
-0.221160329	-0.283053894	-0.743175114	-0.447277702	-1.155685685	-1.155685685	2.446367302	0.843415842	0.733008864
-0.143614619	-0.952074731	-1.315100135	-0.753353465	0.687093856	-0.856946557	1.281622079	1.049062951	1.00331062
-0.260782912	-0.389076313	-0.08339757	-0.211477384	-0.389076313	-0.207655673	1.292597829	1.220434277	0.469302612
-0.599617869	-0.554144202	-0.72791127	-0.451712405	-0.527532472	-0.273735377	1.470268537	0.743632844	1.196948729
-0.62738572	-0.843658535	-0.628152499	-0.319941709	-0.723257439	-0.347458183	1.13367793	0.864433306	1.491742848
-0.270727979	-0.589060383	-0.691691459	-0.300310769	-0.588228139	-0.492469157	1.259907428	1.100782145	1.231906645
-0.876479533	-0.771362827	-1.144314925	0.206182322	-0.384732952	0.117516734	1.696450412	1.137234968	1.019505801
-0.605624579	-0.487382517	-1.28729872	-0.596658142	-0.100164429	-0.833122827	1.484798072	0.978851459	1.827567997
-0.00688773	-0.035079657	-0.559026383	-1.588835193	-1.146757442	-1.079618053	1.647074104	1.353360948	1.415769407
-0.108494642	-0.524034885	-1.257779571	-0.253247416	-0.090440649	-0.799593716	1.789863917	1.347060668	0.896666294
-0.57450602	-1.414807307	-1.16802277	-0.50831799	-0.413430633	-0.935600416	1.749380728	1.431605964	1.833698444
-0.272191424	-0.760502432	-1.911708957	-0.801632507	-0.596514844	-0.466616836	1.744832883	1.549064643	1.515269474
-0.560731184	-0.872104536	-1.142387025	-0.311445283	-0.531889432	-0.172929341	1.852277237	1.251439493	1.48777007
0.04574164	-0.614852685	-0.831630286	-0.572556333	-1.006193743	-0.877637078	1.113020593	1.260070969	1.484036924
-0.8933553	-0.202270067	-1.173123722	-0.74873905	-0.826025051	-0.796833338	2.240876468	1.161628083	2.561098144
-0.220220808	-1.494831627	-1.186218706	-0.530636706	-0.849979879	-0.276710133	1.893404167	1.732484997	0.932708696
0.064365228	-1.364740037	-0.76289582	-0.743557349	-1.106979329	-0.503109846	1.072100877	1.669367222	1.675449053
-0.410209621	-0.764606489	-0.67544557	-1.266315262	-1.249729302	-0.591475684	1.769098635	1.165008934	2.023674358

AT1G16390.1	OCT3_ARATHOrganic cation carnitine transporter 3 OS=Arabidopsis thaliana GN=OCT3 PE=2 SV=1
AT1G03850.2	GRS13_ARATHMonothiol glutaredoxin-S13 OS=Arabidopsis thaliana GN=GRXS13 PE=2 SV=2
AT5G03040.3	IQD1_ARATH IQ-DOMAIN 1 OS=Arabidopsis thaliana GN=IQD1 PE=1 SV=1
AT5G06570.1	CXE15_ARATHProbable carboxylesterase 15 OS=Arabidopsis thaliana GN=CXE15 PE=2 SV=1
AT2G41100.1	CML12_ARATHCalmodulin 12 OS=Arabidopsis thaliana GN=CML12 PE=1 SV=3
AT3G52520.1	F-box only 11
AT4G34970.1	ADF9_ARATHActin-depolymerizing factor 9 OS=Arabidopsis thaliana GN=ADF9 PE=2 SV=2
**Cluster 32**	**Description**
AT2G21385.1	PREDICTED: uncharacterized protein LOC103490346 isoform X2
AT5G53450.1	PAP14_ARATHProbable plastid-lipid-associated chloroplastic OS=Arabidopsis thaliana GN=PAP14 PE=1 SV=1
AT1G18660.2	LONF1_HUMANLON peptidase N-terminal domain and RING finger 1 OS=Homo sapiens GN=LONRF1 PE=1 SV=2
AT5G51140.2	PUS7_ARATHRNA pseudouridine synthase 7 OS=Arabidopsis thaliana GN=At5g51140 PE=2 SV=1
AT3G14420.2	GLO1_ARATHPeroxisomal (S)-2-hydroxy-acid oxidase GLO1 OS=Arabidopsis thaliana GN=GLO1 PE=1 SV=1
AT5G53300.3	UBC10_ARATHUbiquitin-conjugating enzyme E2 10 OS=Arabidopsis thaliana GN=UBC10 PE=1 SV=1
**Cluster 33**	**Description**
AT4G39150.2	DNJ10_ARATHChaperone dnaJ 10 OS=Arabidopsis thaliana GN=ATJ10 PE=2 SV=2
AT4G13940.3	SAHH1_ARATHAdenosylhomocysteinase 1 OS=Arabidopsis thaliana GN=SAHH1 PE=1 SV=1
**Cluster 34**	**Description**
AT5G57930.1	APO2_ARATHAPO chloroplastic OS=Arabidopsis thaliana GN=APO2 PE=2 SV=1
AT3G44300.1	NRL2_ARATHNitrilase 2 OS=Arabidopsis thaliana GN=NIT2 PE=1 SV=1
AT3G50770.1	CML41_ARATHProbable calcium-binding CML41 OS=Arabidopsis thaliana GN=CML41 PE=2 SV=2
AT4G02170.1	neuronal acetylcholine receptor subunit alpha-2-like
AT1G64220.1	TOM72_ARATHMitochondrial import receptor subunit TOM7-2 OS=Arabidopsis thaliana GN=TOM7-2 PE=2 SV=1
AT5G57510.1	PREDICTED: uncharacterized protein LOC103494271
AT3G54810.1	GATA8_ARATHGATA transcription factor 8 OS=Arabidopsis thaliana GN=GATA8 PE=2 SV=1
AT5G50915.2	BH137_ARATHTranscription factor bHLH137 OS=Arabidopsis thaliana GN=BHLH137 PE=2 SV=1
**Cluster 35**	**Description**
AT4G30280.1	XTH18_ARATHProbable xyloglucan endotransglucosylase hydrolase 18 OS=Arabidopsis thaliana GN=XTH18 PE=2 SV=1
AT2G19800.1	MIOX2_ARATHInositol oxygenase 2 OS=Arabidopsis thaliana GN=MIOX2 PE=2 SV=2
AT1G35140.1	EXOL1_ARATH EXORDIUM-like 1 OS=Arabidopsis thaliana GN=EXL1 PE=2 SV=1
AT5G39580.1	PER62_ARATHPeroxidase 62 OS=Arabidopsis thaliana GN=PER62 PE=2 SV=1
AT4G25810.1	XTH23_ARATHProbable xyloglucan endotransglucosylase hydrolase 23 OS=Arabidopsis thaliana GN=XTH23 PE=2 SV=1
AT1G77640.1	ERF13_ARATHEthylene-responsive transcription factor ERF013 OS=Arabidopsis thaliana GN=ERF013 PE=2 SV=1
**Cluster 36**	**Description**
AT1G05680.1	U74E2_ARATHUDP-glycosyltransferase 74E2 OS=Arabidopsis thaliana GN=UGT74E2 PE=1 SV=1
AT3G05400.1	EDL12_ARATHSugar transporter ERD6-like 12 OS=Arabidopsis thaliana GN=SUGTL5 PE=2 SV=1
AT5G59510.1	rotundifolia [Medicago truncatula]
AT3G49570.1	bicaudal D-related 2-like
AT5G24660.1	golgin subfamily A member 6 3
AT5G26220.1	CHAC2_MOUSE glutathione-specific gamma-glutamylcyclotransferase 2 OS=Mus musculus GN=Chac2 PE=1 SV=1
AT5G48850.1	SDI1_ARATH SULFUR DEFICIENCY-INDUCED 1 OS=Arabidopsis thaliana GN=SDI1 PE=2 SV=1
AT4G30650.1	RC23_ARATHUPF0057 membrane At4g30650 OS=Arabidopsis thaliana GN=At4g30650 PE=3 SV=1
AT3G49580.1	bicaudal D-related 2-like
**Cluster 37**	**Description**
AT1G02640.1	BXL2_ARATHProbable beta-D-xylosidase 2 OS=Arabidopsis thaliana GN=BXL2 PE=2 SV=1
AT5G22920.1	MIEL1_ARATHE3 ubiquitin- ligase MIEL1 OS=Arabidopsis thaliana GN=MIEL1 PE=1 SV=1
AT1G11260.1	STP1_ARATHSugar transport 1 OS=Arabidopsis thaliana GN=STP1 PE=1 SV=2
AT4G21870.1	kDa class I heat shock OS=Oryza sativa japonica GN= PE=2 SV=1
AT4G36850.1	YPQ1_YEASTProbable vacuolar amino acid transporter YPQ1 OS=Saccharomyces cerevisiae (strain ATCC 204508 S288c) GN=YPQ1 PE=1 SV=1
AT2G20670.1	plant F8B4-180 [Medicago truncatula]
AT1G77210.2	STP14_ARATHSugar transport 14 OS=Arabidopsis thaliana GN=STP14 PE=2 SV=2
AT3G47340.3	ASNS_TRIVSAsparagine synthetase
AT1G03090.2	MCCA_ARATHMethylcrotonoyl- carboxylase subunit mitochondrial OS=Arabidopsis thaliana GN=MCCA PE=1 SV=2
AT3G15450.1	TSJT1_TOBACStem-specific TSJT1 OS=Nicotiana tabacum GN=TSJT1 PE=2 SV=1
AT3G62950.1	GRC11_ARATHGlutaredoxin-C11 OS=Arabidopsis thaliana GN=GRXC11 PE=3 SV=1
AT3G20395.1	NIPL1_ARATHNEP1-interacting -like 1 OS=Arabidopsis thaliana GN=ATL27 PE=2 SV=1
AT3G47340.2	ASNS1_ARATHAsparagine synthetase
**Cluster 38**	**Description**
AT5G49450.1	BZIP1_ARATHBasic leucine zipper 1 OS=Arabidopsis thaliana GN=BZIP1 PE=1 SV=1
AT5G05440.1	PYL5_ARATHAbscisic acid receptor PYL5 OS=Arabidopsis thaliana GN=PYL5 PE=1 SV=1
AT3G06850.2	ODB2_ARATHLipoamide acyltransferase component of branched-chain alpha-keto acid dehydrogenase mitochondrial OS=Arabidopsis thaliana GN=BCE2 PE=1 SV=1
AT5G23210.2	SCP34_ARATHSerine carboxypeptidase-like 34 OS=Arabidopsis thaliana GN=SCPL34 PE=2 SV=2
AT1G58180.4	BCA6_ARATHBeta carbonic anhydrase mitochondrial OS=Arabidopsis thaliana GN=BCA6 PE=2 SV=1
AT3G58030.2	RNF5_HUMANE3 ubiquitin- ligase RNF5 OS=Homo sapiens GN=RNF5 PE=1 SV=1
AT2G45170.1	ATG8E_ARATHAutophagy-related 8e OS=Arabidopsis thaliana GN=ATG8E PE=1 SV=2
AT5G63470.1	NFYC4_ARATHNuclear transcription factor Y subunit C-4 OS=Arabidopsis thaliana GN=NFYC4 PE=2 SV=1
AT1G75500.1	WAT1_ARATH WALLS ARE THIN 1 OS=Arabidopsis thaliana GN=WAT1 PE=1 SV=1
AT5G64460.6	PGML1_ARATHPhosphoglycerate mutase 1 OS=Arabidopsis thaliana GN=At5g64460 PE=2 SV=1
AT5G08350.1	GEML4_ARATHGEM 4 OS=Arabidopsis thaliana GN=At5g08350 PE=2 SV=1
AT3G18980.2	FB162_ARATHF-box ETP1 OS=Arabidopsis thaliana GN=ETP1 PE=1 SV=1
AT4G34770.1	ARG7_VIGRRIndole-3-acetic acid-induced ARG7 OS=Vigna radiata radiata GN=ARG7 PE=2 SV=1
AT4G01330.1	Y1154_ARATHProbable serine threonine- kinase At1g01540 OS=Arabidopsis thaliana GN=At1g01540 PE=1 SV=2
AT3G10120.1	PREDICTED: uncharacterized protein LOC103433264
AT5G49448.1	BZIP1_ARATHBasic leucine zipper 1 OS=Arabidopsis thaliana GN=BZIP1 PE=1 SV=1
AT4G01330.2	Y1154_ARATHProbable serine threonine- kinase At1g01540 OS=Arabidopsis thaliana GN=At1g01540 PE=1 SV=2
**Cluster 39**	**Description**
AT1G71000.1	DNAJ_THESQChaperone OS=Thermotoga (strain RQ2) GN=dnaJ PE=3 SV=1
AT1G16850.1	transmembrane protein
**Cluster 40**	**Description**
AT1G51090.1	heavy-metal-associated domain [Medicago truncatula]
AT2G42530.1	CR15B_ARATH COLD-REGULATED chloroplastic OS=Arabidopsis thaliana GN=COR15B PE=1 SV=1
AT5G52310.1	LTI78_ARATHLow-temperature-induced 78 kDa OS=Arabidopsis thaliana GN=LTI78 PE=1 SV=2
AT2G42540.2	CR15A_ARATH COLD-REGULATED chloroplastic OS=Arabidopsis thaliana GN=COR15A PE=1 SV=1
AT1G56650.1	MYB75_ARATHTranscription factor MYB75 OS=Arabidopsis thaliana GN=MYB75 PE=1 SV=1
AT1G09500.2	TKPR1_ARATHTetraketide alpha-pyrone reductase 1 OS=Arabidopsis thaliana GN=TKPR1 PE=1 SV=1
AT5G15960.1	KIN1_ARATHStress-induced KIN1 OS=Arabidopsis thaliana GN=KIN1 PE=2 SV=1
AT1G02205.1	CER1_ARATH ECERIFERUM 1 OS=Arabidopsis thaliana GN=CER1 PE=1 SV=1
AT3G50970.1	XERO2_ARATHDehydrin Xero 2 OS=Arabidopsis thaliana GN=XERO2 PE=2 SV=1
**Cluster 41**	**Description**
AT5G24770.2	VSP2_ARATHVegetative storage 2 OS=Arabidopsis thaliana GN=VSP2 PE=2 SV=1
AT1G07400.1	kDa class I heat shock OS=Arabidopsis thaliana GN= PE=1 SV=1
AT2G26150.1	HSFA2_ARATHHeat stress transcription factor A-2 OS=Arabidopsis thaliana GN=HSFA2 PE=1 SV=1
**Cluster 42**	**Description**
AT3G07360.3	PUB9_ARATHU-box domain-containing 9 OS=Arabidopsis thaliana GN=PUB9 PE=1 SV=1
AT1G53570.3	YODA_ARATHMitogen-activated kinase kinase kinase YODA OS=Arabidopsis thaliana GN=YDA PE=1 SV=1
AT5G11500.2	CCD25_BOVINCoiled-coil domain-containing 25 OS=Bos taurus GN=CCDC25 PE=2 SV=1
AT1G10570.2	ULP1C_ARATHUbiquitin-like-specific protease 1C OS=Arabidopsis thaliana GN=ULP1C PE=1 SV=1
AT2G43160.3	EPN2_ARATHClathrin interactor EPSIN 2 OS=Arabidopsis thaliana GN=EPSIN2 PE=1 SV=1
AT4G24820.2	PSMD6_ARATH26S proteasome non-ATPase regulatory subunit 6 homolog OS=Arabidopsis thaliana GN=RPN7 PE=1 SV=1
**Cluster 43**	**Description**
AT2G24270.2	GAPN_ARATHNADP-dependent glyceraldehyde-3-phosphate dehydrogenase OS=Arabidopsis thaliana GN=ALDH11A3 PE=1 SV=2
AT4G10120.2	SPSA4_ARATHProbable sucrose-phosphate synthase 4 OS=Arabidopsis thaliana GN=SPS4 PE=1 SV=1
**Cluster 44**	**Description**
AT5G20250.1	RFS6_ARATHProbable galactinol--sucrose galactosyltransferase 6 OS=Arabidopsis thaliana GN=RFS6 PE=2 SV=2
AT3G15450.2	TSJT1_TOBACStem-specific TSJT1 OS=Nicotiana tabacum GN=TSJT1 PE=2 SV=1
AT5G49360.1	BXL1_ARATHBeta-D-xylosidase 1 OS=Arabidopsis thaliana GN=BXL1 PE=1 SV=1
AT5G20250.2	RFS6_ARATHProbable galactinol--sucrose galactosyltransferase 6 OS=Arabidopsis thaliana GN=RFS6 PE=2 SV=2

-0.932428278	-0.861906587	0.413938845	-0.992983057	-1.370818425	-0.58060535	1.861227754	0.707084957	1.756490142
-0.369080297	-0.693963041	-1.62008639	-0.395565025	0.113067279	-1.158800682	1.475709499	1.139686233	2.050244295
-1.094422529	-0.198318997	-0.439393271	0.471906701	-0.637332648	-0.785831395	1.242866431	0.723921103	0.716604604
-0.776190325	-0.653020646	-1.066614729	-0.323315201	-0.644381728	-1.066614729	1.710962793	1.06530395	1.753870614
-0.887318622	-0.647866748	-0.137064693	-0.594137761	-1.373896115	-0.43012191	1.356952045	1.117342166	1.596111638
-0.061112406	-0.939002645	-0.885173229	-0.721592453	-0.631357353	-0.553719941	0.947942466	0.703460254	2.140555306
-0.172294931	-1.242344121	-0.409905254	-0.92901205	-0.193541655	-1.554485163	2.092807409	0.886530721	1.522245044
**WT-C**	**KO-C**	**OE-C**	**WT-2h**	**KO-2h**	**OE-2h**	**WT-12h**	**KO-12h**	**OE-12h**
-3.849458457	-3.765224073	-3.026728309	-0.286178276	0.759705503	1.365909515	1.611415136	0.986819176	1.203739786
-4.401388354	-3.427960756	-3.631828169	0.290313297	1.462327135	1.128432593	0.619513825	1.503649594	1.456940836
-4.331620573	-3.836309157	-3.948123629	0.976682048	1.27890233	1.119359689	1.58107719	1.642954626	0.517077474
-3.894862778	-4.491879204	-3.289842625	0.561811972	1.02966472	0.440491844	1.040172585	0.866794824	0.753890255
-3.615603115	-3.942838356	-3.338194818	0.929847928	0.963810884	0.709783299	2.204176816	2.150780026	1.938237336
-3.955879061	-3.713162242	-3.356731662	1.511412648	2.413572154	1.199895465	0.914929496	0.38077031	0.891729568
**WT-C**	**KO-C**	**OE-C**	**WT-2h**	**KO-2h**	**OE-2h**	**WT-12h**	**KO-12h**	**OE-12h**
2.115149257	1.941302367	2.485188925	-2.206518987	-3.482947919	-3.354654518	0.110883882	0.594643024	0.573202042
2.444670774	1.921086244	3.129552317	-2.502178372	-2.904697763	-3.138269033	-0.105286617	0.232697296	0.41806841
**WT-C**	**KO-C**	**OE-C**	**WT-2h**	**KO-2h**	**OE-2h**	**WT-12h**	**KO-12h**	**OE-12h**
3.174034971	2.507341546	2.898029521	-1.265388702	-1.265388702	-1.265388702	-1.252462528	-1.265388702	-1.265388702
2.31593931	2.696094716	1.906506217	-1.709738968	-1.481809853	-0.670217289	-1.116477887	-0.847803223	-0.325448798
1.951430467	1.799092249	1.651220269	-1.50678678	-2.3625016	-0.433006456	-0.375089264	-1.388605354	-0.624362131
3.038202674	2.743875415	2.68646465	-2.493709346	-2.710953401	-1.043620632	-0.667574589	-0.731568143	-0.821116629
3.048743903	2.51771851	2.73469809	-1.466993819	-1.632009267	-1.865799331	-1.014404354	-0.529512105	-0.792441626
1.949881017	1.846152178	3.297584894	-1.54334862	-2.062114378	-1.19184379	-0.076554852	-1.056618391	-0.163138058
2.976315041	3.082828171	3.224890499	-1.215637224	-2.559613484	-1.043093986	-0.862729393	-0.559613484	-0.04334614
2.124736751	2.539051108	2.781759512	-1.352925284	-2.529836715	-1.123315896	-0.481796963	-1.505875169	-0.451797344
**WT-C**	**KO-C**	**OE-C**	**WT-2h**	**KO-2h**	**OE-2h**	**WT-12h**	**KO-12h**	**OE-12h**
1.024018771	1.075357954	1.695427355	-2.949429857	-2.874010506	-1.983112983	2.009844278	2.395203468	2.397108456
1.447714158	0.822283507	1.124947337	-2.454050774	-3.025435647	-1.56341942	2.408339412	2.706212874	1.945834302
1.917773767	1.909278707	1.773607833	-3.66543127	-3.465012529	-2.089978329	1.698942574	2.093287985	2.014107231
1.360684491	0.970464946	1.24102137	-1.990293422	-3.227037695	-2.407224787	2.485735761	2.318526094	2.885175429
1.476786219	1.573618145	1.345825609	-2.650540469	-2.758847836	-1.744926308	1.695191389	2.341854646	1.856311797
1.45748828	2.106952701	1.584683643	-2.856373123	-2.724808595	-1.954820126	1.576235732	2.057338701	2.367980189
**WT-C**	**KO-C**	**OE-C**	**WT-2h**	**KO-2h**	**OE-2h**	**WT-12h**	**KO-12h**	**OE-12h**
1.902546569	1.537041643	2.480724253	0.661405471	1.436556375	1.089964406	-3.508953248	-2.84946972	-3.749815749
1.266550686	1.183207902	1.691645911	1.336990549	1.876087258	0.696670035	-3.990393226	-2.726427415	-2.3343317
2.265507307	1.290017576	2.879340715	1.232230055	0.698919691	1.213317417	-3.699795828	-2.398678436	-3.480858437
1.54275555	1.064987028	1.954593609	2.089531601	2.067454863	1.043664089	-4.780566574	-2.492555649	-2.489864518
2.165910508	1.550679994	1.920043979	1.793049366	1.484880833	1.256669711	-4.823743881	-2.593706647	-2.753783863
1.293803082	1.076180422	1.559830587	1.537987867	0.861475616	0.850801008	-4.200912471	-3.048477027	-1.930689083
1.582957997	1.815626959	2.252310542	1.552914159	1.167534802	0.822056389	-4.491834387	-2.80189726	-1.8996692
1.956658664	0.687248289	1.645518261	0.93246568	0.914623177	0.598603423	-3.84081684	-2.513640512	-3.183453296
2.016434669	1.245043825	1.978584683	1.945748598	1.616905122	0.328298074	-5.467695771	-2.349903386	-2.313415815
**WT-C**	**KO-C**	**OE-C**	**WT-2h**	**KO-2h**	**OE-2h**	**WT-12h**	**KO-12h**	**OE-12h**
-1.240616122	-1.511010417	-1.521636274	-1.172051836	-1.34740857	-0.075664711	3.188059027	2.514862334	2.165466569
-0.616113354	-2.441404207	-2.274197596	-2.566212437	-2.643038069	0.179189386	4.251028184	2.723686378	3.387061716
-1.038769885	-1.717876532	-2.115315483	-1.97353759	-1.91517449	-0.641182124	3.468515936	2.816446371	3.116893797
-0.724513768	-2.137966107	-1.648966905	-1.610211396	-1.403397405	-1.392967341	2.67923577	2.799828512	3.43895864
-0.550830899	-1.72346417	-1.07879961	-1.943148322	-1.662499358	-0.261858499	2.897028728	2.975021183	2.226070011
-0.621182963	-1.388867739	-2.266992172	-1.811581435	-2.051259861	-1.260038471	3.497849767	3.031615563	2.870457311
-1.327892898	-1.698080052	-2.015027925	-1.130965552	-1.099386835	-0.707348329	3.311014311	2.832150373	2.835536908
-1.227844545	-1.593774905	-2.41180738	-1.600336349	-1.540752305	-0.34029892	4.042450782	2.107482899	1.884282883
-1.971495524	-1.683070326	-2.481447048	-0.605823866	-1.039515997	-0.013114613	3.684462532	2.575794724	2.534210118
-0.364102522	-2.435041661	-2.755095875	-1.240797683	-2.469804176	-0.27544509	3.93412854	2.979678636	3.626479831
-2.037237177	-2.153601933	-2.153601933	-1.33393375	-0.966467642	-0.248636215	3.830600034	2.162543809	2.900334806
-0.656671336	-2.16855451	-1.648981502	-1.433300153	-2.277632605	-0.422698774	2.939385591	2.84739039	2.821062899
-1.234815762	-1.575464308	-2.921428339	-1.931562716	-1.77803492	-0.367085073	4.47104365	2.539674526	2.797672941
**WT-C**	**KO-C**	**OE-C**	**WT-2h**	**KO-2h**	**OE-2h**	**WT-12h**	**KO-12h**	**OE-12h**
-0.815055189	-0.862467166	-1.376615549	-1.011267649	-0.876358756	-1.594137577	2.009643504	1.553157974	1.976539701
-0.807366983	-0.558397021	-0.936324878	-1.696111871	-1.314713662	-1.482995797	1.71613935	1.80755038	1.657486515
-0.618584804	-1.541730931	-0.648311301	-1.134113147	-0.882390609	-2.128871742	2.431798873	1.289676661	1.995357391
-1.223037394	-2.521339013	-1.471708245	-1.653442549	-0.943382379	-0.113170642	1.793357513	2.039009108	1.847638812
-1.052268179	-0.809666296	-2.057593809	-2.057593809	-0.968095658	-2.057593809	2.524359942	2.096940795	2.276974466
-1.02452423	-1.742321028	-1.837232675	-1.837232675	-1.325621853	-0.69776318	1.109311053	1.46552504	1.189567384
-0.883908049	-1.25111317	-1.784620337	-1.40223229	-0.914662355	-1.201644494	2.239262079	1.971960576	2.054659187
-1.302683037	-1.548346654	-0.687955643	-1.24622704	-1.46706901	-0.747564386	1.910637779	1.779035871	1.704806045
-0.611319209	-1.363009827	-0.693583088	-0.782015683	-0.744362676	-1.273694935	1.387401463	1.923230455	1.702147484
-0.635894345	-2.047352951	-2.047352951	-2.047352951	-1.141364458	-0.146244708	1.986510501	1.2398238	2.284710458
-0.974514653	-1.056827125	-1.046951251	-1.128352393	-1.366011571	-1.314256904	2.238051339	1.378912896	2.492886474
-1.501772431	-1.662852966	-1.662852966	-1.662852966	-0.416748956	-1.662852966	1.866468259	1.726438496	1.973481636
-0.013370859	-1.605862407	-0.555466029	-1.72089565	-0.984507249	-1.72089565	1.406902689	3.086562318	2.107532836
0.050346402	-0.637117231	-1.11101308	-0.889504392	-1.597588867	-1.720253075	2.323303604	1.74181025	1.84001639
-0.182035722	-1.602648578	-0.30103171	-1.20684927	-0.479839832	-1.803253779	1.44620222	2.250447687	1.879008983
0.181505519	-0.862467166	-1.376615549	-1.011267649	-0.876358756	-1.594137577	2.009643504	1.553157974	1.976539701
0.084565408	-0.855464841	-1.026887191	-1.776123912	-1.704285097	-0.468859938	1.810902826	2.1600254	1.776127345
**WT-C**	**KO-C**	**OE-C**	**WT-2h**	**KO-2h**	**OE-2h**	**WT-12h**	**KO-12h**	**OE-12h**
2.026793053	2.841026325	3.229939794	2.610566283	2.967108432	1.268695117	-4.84914722	-2.823143492	-5.271838292
1.753482892	2.297952661	2.742186775	1.895020761	3.390569472	1.557879419	-5.398582119	-4.345993079	-4.892516782
**WT-C**	**KO-C**	**OE-C**	**WT-2h**	**KO-2h**	**OE-2h**	**WT-12h**	**KO-12h**	**OE-12h**
1.498046402	1.865522655	2.741350713	2.183159945	2.556718007	1.08395248	-4.341109178	-2.792826509	-4.794814514
1.191147407	0.742573545	0.960142827	2.378926761	4.31017373	2.883571837	-5.383791491	-2.222041507	-4.860703109
0.636064658	1.543613771	2.570360015	2.299735914	2.985063016	1.828227181	-4.856227913	-2.563696944	-4.443139698
0.917338245	1.183325479	3.207439934	1.920864808	3.220693749	2.255655807	-5.567532984	-2.417718656	-4.720066382
1.239681671	1.27744701	2.447406358	2.340382556	2.302836419	1.831564738	-4.906994034	-2.879162295	-3.653162422
0.477547468	1.144944844	1.323047627	2.325933303	3.881452278	2.769513569	-3.754691629	-3.567343477	-4.600403982
0.694208261	1.097782086	2.993622208	2.730433772	4.104000799	2.347076765	-5.887297362	-3.850856601	-4.228969928
0.309754168	0.701287358	1.566711504	2.792511787	3.710584892	1.787140032	-4.589887172	-1.910196383	-4.367906185
0.960210106	1.762758799	3.403499602	2.359256493	3.550716185	1.619921162	-5.484147393	-3.328323225	-4.843891727
**WT-C**	**KO-C**	**OE-C**	**WT-2h**	**KO-2h**	**OE-2h**	**WT-12h**	**KO-12h**	**OE-12h**
0.969939155	1.527630137	1.005782936	3.160251926	3.122640195	1.886650006	-3.033254441	-3.615254528	-3.024385387
1.18376946	1.545911965	0.973043008	2.94489068	2.705947796	2.856753095	-2.842105966	-3.055558733	-4.312651306
1.562270973	1.931686589	1.273272046	3.050736925	3.158694131	2.858271771	-4.003112951	-4.003112951	-2.828706533
**WT-C**	**KO-C**	**OE-C**	**WT-2h**	**KO-2h**	**OE-2h**	**WT-12h**	**KO-12h**	**OE-12h**
-3.422844827	-3.329475602	-3.422844827	0.602272582	0.762347775	0.787466102	1.617258551	0.791202245	-0.044333204
-3.617002623	-3.063434264	-3.617002623	1.701531148	2.287529644	1.395968233	1.139380232	1.053844865	0.200006591
-3.633572965	-3.492672909	-2.799270369	1.099347693	1.770531195	0.902590412	1.164861829	0.305722834	-0.302883537
-3.294232814	-3.368770164	-3.294232814	1.286575965	1.533586211	0.652123758	0.430963003	1.490742219	0.825704309
-3.515413675	-3.34877247	-3.155892505	0.949189251	0.759071643	1.256049383	0.981943211	0.546794403	0.829485819
-4.144195412	-3.386187514	-4.144195412	0.637374133	1.763608601	1.24829375	1.669637074	0.646316	0.936973754
**WT-C**	**KO-C**	**OE-C**	**WT-2h**	**KO-2h**	**OE-2h**	**WT-12h**	**KO-12h**	**OE-12h**
1.09916991	1.015547027	1.520008306	-1.520008306	-1.520008306	-1.594358719	-1.520008306	-0.62005932	-1.391016888
1.277004531	0.855027135	1.383148284	-1.383148284	-1.605207973	-1.308832467	-1.383148284	-1.383148284	-1.231208204
**WT-C**	**KO-C**	**OE-C**	**WT-2h**	**KO-2h**	**OE-2h**	**WT-12h**	**KO-12h**	**OE-12h**
-0.175037076	-0.375828008	-2.91337258	-2.467645877	-2.91337258	-0.461040724	3.455203723	2.804139346	3.046953777
-0.435602921	-1.068006332	-2.110339247	-2.133475067	-3.50173744	-2.202683811	4.208484879	3.211360788	3.160793309
-0.472618416	-0.653231271	-3.560960704	-2.593176598	-2.70346805	-0.704010845	3.79645699	2.955839149	2.989932913
-0.342279452	-0.34015057	-2.135631392	-1.407443218	-2.007475041	-2.338448275	3.764555751	2.678437083	3.443876212

AT1G08930.1	ERD6_ARATHSugar transporter ERD6 OS=Arabidopsis thaliana GN=ERD6 PE=1 SV=3
AT4G34530.1	BH063_ARATHTranscription factor bHLH63 OS=Arabidopsis thaliana GN=BHLH63 PE=1 SV=1
AT5G19190.1	unnamed protein product
AT1G73830.1	BEE3_ARATHTranscription factor BEE 3 OS=Arabidopsis thaliana GN=BEE3 PE=2 SV=1

Cluster 45	Description
AT3G17790.1	PPA17_ARATHPurple acid phosphatase 17 OS=Arabidopsis thaliana GN=PAP17 PE=2 SV=1
AT1G15100.1	RHA2A_ARATHE3 ubiquitin- ligase RHA2A OS=Arabidopsis thaliana GN=RHA2A PE=1 SV=1
AT1G05560.1	U75B1_ARATHUDP-glycosyltransferase 75B1 OS=Arabidopsis thaliana GN=UGT75B1 PE=1 SV=1
AT3G21700.3	SPG1_SCHPOSeptum-promoting GTP-binding 1 OS=Schizosaccharomyces pombe (strain 972 ATCC 24843) GN=spg1 PE=1 SV=1
AT1G02220.1	NAC3_ARATHNAC domain-containing 3 OS=Arabidopsis thaliana GN=NAC003 PE=2 SV=1
AT5G17760.1	AATPI_ARATHAAA-ATPase At5g17760 OS=Arabidopsis thaliana GN=At5g17760 PE=2 SV=1
AT1G16300.1	G3PP2_ARATHGlyceraldehyde-3-phosphate dehydrogenase chloroplastic OS=Arabidopsis thaliana GN=GAPCP2 PE=2 SV=1
AT4G21215.2	transmembrane [Medicago truncatula]
AT1G52080.1	CHUP1_ARATH chloroplastic OS=Arabidopsis thaliana GN=CHUP1 PE=1 SV=1
AT5G16980.2	P1_ARATHNADP-dependent alkenal double bond reductase P1 OS=Arabidopsis thaliana GN=P1 PE=1 SV=1
AT5G53120.5	SPSY_ARATHSpermine synthase OS=Arabidopsis thaliana GN=SPMS PE=1 SV=1
AT4G33540.1	YQJP_BACSUProbable metallo-hydrolase OS=Bacillus subtilis (strain 168) GN=yqjP PE=3 SV=1
AT4G30830.1	MYOB7_ARATHMyosin-binding 7 OS=Arabidopsis thaliana GN=MYOB7 PE=1 SV=1
AT4G00900.1	ECA2_ARATHCalcium-transporting ATPase endoplasmic reticulum-type OS=Arabidopsis thaliana GN=ECA2 PE=1 SV=1
AT4G09020.1	ISOA3_ARATHIsoamylase chloroplastic OS=Arabidopsis thaliana GN=ISA3 PE=2 SV=2
AT4G34860.1	INVB_ARATHProbable alkaline neutral invertase B OS=Arabidopsis thaliana GN=INVB PE=1 SV=1
AT1G78600.1	BBX22_ARATHB-box zinc finger 22 OS=Arabidopsis thaliana GN=BBX22 PE=1 SV=2
AT1G04570.1	FBT8_ARATHProbable folate-biopterin transporter chloroplastic OS=Arabidopsis thaliana GN=At1g04570 PE=2 SV=1
AT5G04250.1	Y4757_DICDIOTU domain-containing DDB_G0284757 OS=Dictyostelium discoideum GN=DDB_G0284757 PE=3 SV=2
AT4G15120.1	VQ22_ARATHVQ motif-containing 22 OS=Arabidopsis thaliana GN=VQ22 PE=2 SV=1
AT5G13700.1	PAO1_ARATHPolyamine oxidase 1 OS=Arabidopsis thaliana GN=PAO1 PE=1 SV=1
AT5G59845.1	GASAA_ARATHGibberellin-regulated 10 OS=Arabidopsis thaliana GN=GASA10 PE=2 SV=1
AT4G37980.2	CADH7_ARATHCinnamyl alcohol dehydrogenase 7 OS=Arabidopsis thaliana GN=CAD7 PE=1 SV=2
AT1G63840.1	RHA1B_ARATHE3 ubiquitin- ligase RHA1B OS=Arabidopsis thaliana GN=RHA1B PE=2 SV=1
AT1G10530.1	D-ribose-binding periplasmic
AT1G67856.1	XERIC_ARATHProbable E3 ubiquitin- ligase XERICO OS=Arabidopsis thaliana GN=XERICO PE=1 SV=1
AT1G52565.1	PREDICTED: uncharacterized protein LOC103494703
AT2G31560.1	DUF1685 family [Medicago truncatula]
AT5G09610.1	PUM21_ARATH pumilio homolog 21 OS=Arabidopsis thaliana GN=APUM21 PE=3 SV=1
AT1G51760.1	ILL4_ARATHIAA-amino acid hydrolase ILR1-like 4 OS=Arabidopsis thaliana GN=ILL4 PE=1 SV=2
AT1G32870.1	NAC13_ARATHNAC domain-containing 13 OS=Arabidopsis thaliana GN=NAC13 PE=1 SV=1
AT4G34230.1	CADH5_ARATHCinnamyl alcohol dehydrogenase 5 OS=Arabidopsis thaliana GN=CAD5 PE=1 SV=1
AT5G57050.2	P2C77_ARATH phosphatase 2C 77 OS=Arabidopsis thaliana GN=ABI2 PE=1 SV=1
AT2G28320.1	EDR2L_ARATH ENHANCED DISEASE RESISTANCE 2-like OS=Arabidopsis thaliana GN=EDR2L PE=2 SV=1
AT5G60650.1	PREDICTED: uncharacterized protein LOC103422926
AT2G15970.1	CRPM1_ARATHCold-regulated 413 plasma membrane 1 OS=Arabidopsis thaliana GN=COR413PM1 PE=2 SV=1
AT1G17420.1	LOX3_ARATHLipoxygenase chloroplastic OS=Arabidopsis thaliana GN=LOX3 PE=2 SV=1
AT1G79270.1	YTHD2_MOUSEYTH domain-containing family 2 OS=Mus musculus GN=Ythdf2 PE=1 SV=1
AT5G04830.1	PREDICTED: uncharacterized protein LOC103489375
AT1G72120.1	PTR22_ARATH NRT1 PTR FAMILY OS=Arabidopsis thaliana GN= PE=2 SV=2
AT5G64750.1	ABR1_ARATHEthylene-responsive transcription factor ABR1 OS=Arabidopsis thaliana GN=ABR1 PE=2 SV=1
AT1G70782.1	Y4276_ARATHUncharacterized protein At4g22758 OS=Arabidopsis thaliana GN=At4g22758 PE=2 SV=1
AT5G24150.1	ERG11_ARATHSqualene epoxidase 5 OS=Arabidopsis thaliana GN=SQE5 PE=2 SV=2
AT4G22590.1	TPPG_ARATHProbable trehalose-phosphate phosphatase G OS=Arabidopsis thaliana GN=TPPG PE=2 SV=1
AT1G24280.1	G6PD3_ARATHGlucose-6-phosphate 1-dehydrogenase chloroplastic OS=Arabidopsis thaliana GN=At1g24280 PE=1 SV=2
AT5G16970.1	P1_ARATHNADP-dependent alkenal double bond reductase P1 OS=Arabidopsis thaliana GN=P1 PE=1 SV=1
AT4G32920.1	transmembrane [Medicago truncatula]
AT1G63820.1	COL3_ARATHZinc finger CONSTANS-LIKE 3 OS=Arabidopsis thaliana GN=COL3 PE=1 SV=1
AT5G67350.1	probable membrane-associated kinase regulator 1
AT4G31860.2	P2C60_ARATHProbable phosphatase 2C 60 OS=Arabidopsis thaliana GN=At4g31860 PE=2 SV=1
AT5G55120.1	GGAP2_ARATHGDP-L-galactose phosphorylase 2 OS=Arabidopsis thaliana GN=VTC5 PE=1 SV=1
AT2G28400.1	senescence regulator [Medicago truncatula]
AT1G36370.1	GLYC7_ARATHSerine hydroxymethyltransferase 7 OS=Arabidopsis thaliana GN=SHM7 PE=2 SV=1
AT3G05640.2	P2C34_ARATHProbable phosphatase 2C 34 OS=Arabidopsis thaliana GN=At3g05640 PE=2 SV=1
AT1G48370.1	YSL8_ARATHProbable metal-nicotianamine transporter YSL8 OS=Arabidopsis thaliana GN=YSL8 PE=1 SV=2
AT1G47510.1	IP5PB_ARATHType IV inositol polyphosphate 5-phosphatase 11 OS=Arabidopsis thaliana GN=IP5P11 PE=1 SV=1
AT1G77450.1	NAC2_ARATHNAC domain-containing 2 OS=Arabidopsis thaliana GN=NAC002 PE=2 SV=2
AT5G61960.1	AML1_ARATH MEI2-like 1 OS=Arabidopsis thaliana GN=ML1 PE=1 SV=1
AT5G09390.2	CD2B2_MOUSECD2 antigen cytoplasmic tail-binding 2 OS=Mus musculus GN=Cd2bp2 PE=1 SV=1
AT3G09910.3	RAC2B_ARATHRas-related RABC2b OS=Arabidopsis thaliana GN=RABC2B PE=2 SV=1
AT5G59480.1	SDT1_YEASTSuppressor of disruption of TFIIS OS=Saccharomyces cerevisiae (strain ATCC 204508 S288c) GN=SDT1 PE=1 SV=1
AT3G22560.1	YOAA_BACSUUncharacterized N-acetyltransferase OS=Bacillus subtilis (strain 168) GN=yoaA PE=3 SV=2
AT1G78070.1	YGI3_SCHPOUncharacterized WD repeat-containing OS=Schizosaccharomyces pombe (strain 972 ATCC 24843) GN= PE=3 SV=2
AT1G24600.1	hypothetical protein AT1G24600
AT4G19230.1	ABAH1_ARATHAbscisic acid 8 -hydroxylase 1 OS=Arabidopsis thaliana GN=CYP707A1 PE=2 SV=1
AT1G02390.1	GPAT2_ARATHProbable glycerol-3-phosphate acyltransferase 2 OS=Arabidopsis thaliana GN=GPAT2 PE=2 SV=1
AT1G66760.2	DTX9_ARATH DETOXIFICATION 9 OS=Arabidopsis thaliana GN=DTX9 PE=2 SV=1
AT5G50100.1	Y5010_ARATHUncharacterized protein At5g50100, mitochondrial OS=Arabidopsis thaliana GN=At5g50100 PE=2 SV=1
AT1G27760.2	IFRD1_PIGInterferon-related developmental regulator 1 OS=Sus scrofa GN=IFRD1 PE=2 SV=1
AT5G64250.1	2NPD_BACSUProbable nitronate monooxygenase OS=Bacillus subtilis (strain 168) GN=yrpB PE=3 SV=1
AT5G61810.1	SCMC1_XENTRCalcium-binding mitochondrial carrier S -1 OS=Xenopus tropicalis GN=slc25a24 PE=2 SV=1
AT5G60790.1	AB1F_ARATHABC transporter F family member 1 OS=Arabidopsis thaliana GN=ABCF1 PE=1 SV=1
AT5G17000.1	P1_ARATHNADP-dependent alkenal double bond reductase P1 OS=Arabidopsis thaliana GN=P1 PE=1 SV=1
AT1G77920.1	TGA7_ARATHTranscription factor TGA7 OS=Arabidopsis thaliana GN=TGA7 PE=1 SV=1
AT1G17830.1	hypothetical protein (DUF789)
AT2G33590.1	CCR2_ARATHCinnamoyl- reductase 2 OS=Arabidopsis thaliana GN=CCR2 PE=1 SV=1
AT3G29575.4	AFP3_ARATHNinja-family AFP3 OS=Arabidopsis thaliana GN=AFP3 PE=1 SV=1
AT1G33110.1	DTX21_ARATH DETOXIFICATION 21 OS=Arabidopsis thaliana GN=DTX21 PE=1 SV=1
AT4G21215.1	transmembrane [Medicago truncatula]
AT1G63940.3	MDAR5_ARATHMonodehydroascorbate reductase mitochondrial OS=Arabidopsis thaliana GN=MDAR5 PE=1 SV=3
AT1G13740.1	AFP2_ARATHNinja-family AFP2 OS=Arabidopsis thaliana GN=AFP2 PE=1 SV=1
AT1G54100.2	AL7B4_ARATHAldehyde dehydrogenase family 7 member B4 OS=Arabidopsis thaliana GN=ALDH7B4 PE=2 SV=3
AT4G01870.1	TOLB_BURXL OS=Burkholderia xenovorans (strain LB400) GN=tolB PE=3 SV=1
AT5G59570.1	PCLL_ARATHTranscription factor BOA OS=Arabidopsis thaliana GN=BOA PE=2 SV=1
AT1G20440.1	COR47_ARATHDehydrin COR47 OS=Arabidopsis thaliana GN=COR47 PE=1 SV=2
AT3G62590.1	triacylglycerol lipase [Medicago truncatula]
AT1G68570.1	PTR18_ARATH NRT1 PTR FAMILY OS=Arabidopsis thaliana GN= PE=2 SV=1
AT3G13910.1	DUF3511 domain [Medicago truncatula]
AT3G22620.1	non-specific lipid-transfer At2g13820
AT3G04240.1	SEC_ARATHProbable UDP-N-acetylglucosamine--peptide N-acetylglucosaminyltransferase SEC OS=Arabidopsis thaliana GN=SEC PE=2 SV=1
AT5G51990.1	DRE1D_ARATHDehydration-responsive element-binding 1D OS=Arabidopsis thaliana GN=DREB1D PE=2 SV=1
AT3G04010.1	E138_ARATHGlucan endo-1,3-beta-glucosidase 8 OS=Arabidopsis thaliana GN=At1g64760 PE=1 SV=2
AT5G14640.1	KSG5_ARATHShaggy-related kinase epsilon OS=Arabidopsis thaliana GN=ASK5 PE=2 SV=1
AT4G13800.1	NIPA2_ARATHProbable magnesium transporter NIPA2 OS=Arabidopsis thaliana GN=At4g13800 PE=2 SV=1
AT5G51070.1	CLPD_ARATHChaperone chloroplastic OS=Arabidopsis thaliana GN=CLPD PE=1 SV=1
AT3G07090.1	DESI1_XENLADesumoylating isopeptidase 1 OS=Xenopus laevis GN=desi1 PE=2 SV=1
AT1G50260.1	SYT3_ARATHSynaptotagmin-3 OS=Arabidopsis thaliana GN=SYT3 PE=2 SV=1
AT4G21440.1	MYB39_ARATHTranscription factor MYB39 OS=Arabidopsis thaliana GN=MYB39 PE=2 SV=1
AT3G59140.1	AB10C_ARATHABC transporter C family member 10 OS=Arabidopsis thaliana GN=ABCC10 PE=2 SV=2
AT5G16450.1	RRAA2_ARATH 4-hydroxy-4-methyl-2-oxoglutarate aldolase 2 OS=Arabidopsis thaliana GN=At5g16450 PE=1 SV=1
AT3G03440.1	PUB7_ARATHU-box domain-containing 7 OS=Arabidopsis thaliana GN=PUB7 PE=2 SV=1
AT4G01895.1	NIMI1_ARATH NIM1-INTERACTING 1 OS=Arabidopsis thaliana GN=NIMIN-1 PE=1 SV=1

-0.610018771	-0.710666371	-3.248210943	-1.600205764	-3.248210943	-1.284736819	3.633710578	3.123957625	2.724343867
-0.88492031	-1.306409389	-1.86748065	-2.394550986	-2.394550986	-1.77631233	2.48848325	3.79379059	2.572110191
-0.448193097	-2.139056883	-2.322215619	-1.975935196	-2.636892497	-1.080142429	3.37378911	4.343502546	2.885144066
-0.297189689	-2.161491669	-2.06550106	-2.078594241	-2.890065272	-1.292653284	2.682794088	4.613180092	3.489521036

WT-C	KO-C	OE-C	WT-2h	KO-2h	OE-2h	WT-12h	KO-12h	OE-12h
0.393368558	0.457187917	2.308248567	0.76244067	1.806739412	0.871603922	-2.183061588	-2.480183962	-1.936343496
-0.022460409	-0.018126579	1.841663897	0.868140578	1.854433115	0.992806348	-1.821071698	-2.078016939	-1.617368312
1.203792842	0.878330689	1.227715659	1.045542341	1.808611849	1.376262866	-2.673683107	-2.160120316	-2.706452823
-0.188213856	0.299915216	2.477537493	0.840803317	2.074592834	1.479936394	-2.62744651	-2.118813201	-2.238311687
0.274812658	0.420989897	0.61627831	1.783762915	2.27556083	1.507169375	-2.117759386	-2.222768693	-2.538045908
0.466728119	0.587608134	1.619452701	0.99177343	1.710576607	1.505852445	-2.517469185	-2.432580288	-1.931941963
0.499846418	0.457420751	1.385678982	1.110454275	2.052412061	1.180662002	-2.348157259	-1.936780495	-2.401536734
0.87965248	1.067047768	1.434945938	1.009645724	1.372656079	0.975154152	-2.440830753	-1.88076983	-2.417501558
0.197281191	0.164991502	1.908017197	0.750679459	2.452599746	1.284890834	-2.593071033	-1.872036827	-2.293352069
1.106871639	-0.121350428	0.486709572	1.352154796	2.193132061	1.474190893	-2.102387598	-1.80806161	-2.581259326
1.432435347	0.022367695	1.265521314	0.620114389	1.42325996	0.702199995	-1.571964074	-1.848354053	-2.045580573
0.225510234	-0.024206463	1.642332104	0.746988366	1.612377412	0.799742604	-1.510627401	-1.568032651	-1.924084206
0.367088882	0.58962702	1.896050033	0.663519902	1.610688636	0.532733294	-1.712619876	-1.25415437	-1.958755757
0.20939539	0.669818027	1.376960636	0.889859977	1.907429935	1.421689686	-2.454773599	-1.843432299	-2.176947752
0.564248084	1.006134941	1.911226281	1.215528943	1.176375541	0.904533156	-2.402172836	-2.288897695	-2.086976416
-0.217314107	1.007636107	1.532979713	0.595467132	1.570026194	1.421014432	-1.655013089	-2.60305224	-1.651744141
0.540478803	0.680072489	1.955336372	0.509628594	1.061504022	0.4114415	-1.785328048	-1.470058377	-1.903075355
0.588429246	0.655200915	2.735402871	0.267227485	1.727727174	1.365995737	-2.053966227	-2.056415625	-2.052743086
0.448900365	0.809493917	0.989522774	0.991350185	2.473018516	1.845425165	-2.697478792	-1.814134041	-3.046098089
0.157144713	0.655072559	1.736081671	1.043842065	1.185290181	0.549881585	-2.248554019	-1.67176545	-1.406993306
0.776671519	1.57538562	1.315185805	1.18836667	1.735800323	0.969741218	-2.172790276	-2.479940896	-2.908419985
0.501383712	0.912061857	1.6414279	-0.260146336	1.484083368	0.69488482	-1.499330034	-1.291699287	-2.182666002
-0.13208905	0.672193397	1.968042474	0.93219803	1.882315051	1.152059932	-2.397597758	-1.503261685	-2.57386039
0.318024071	-0.192607337	1.405499225	0.447946864	1.854129902	1.150761066	-1.819730657	-1.861948252	-1.302074883
0.127822138	-0.206906421	1.188193815	1.221821041	1.655561097	1.68901571	-1.863706884	-1.852791106	-1.95900939
0.501177119	1.496696679	1.930504717	0.456140271	1.638788026	0.776966582	-2.79175339	-2.367579299	-1.640940705
-0.029031792	-0.15860093	1.337968303	1.435611597	2.573720454	1.415144829	-2.697122193	-1.45239473	-2.425295537
0.632019599	1.094407383	1.458118922	0.649455224	1.421687126	0.720380232	-2.243328001	-1.25372431	-2.479016176
0.452111195	0.374317702	2.4605501	-0.208406326	1.749033212	0.596069922	-1.538746661	-1.488993626	-1.491713127
0.405012783	0.07215981	0.675743952	1.140976288	1.58237207	1.949185135	-1.847556714	-2.211306637	-1.766586686
0.795270098	0.96768192	1.750839188	1.060042372	1.031112865	1.213247794	-2.788547981	-2.279814373	-1.749831882
0.378428091	0.816138598	1.919119614	1.23624073	1.600298269	1.340763822	-2.57582178	-2.403444588	-2.311722757
-0.182842488	0.588730071	1.510635596	1.275959618	2.184911593	1.169908071	-1.573456627	-1.768935739	-3.204910095
-0.258833281	0.568984128	0.984420071	0.763023278	1.710582359	1.006140212	-1.646688976	-1.158235085	-1.969392706
0.438991328	0.022113757	1.376171506	0.171315438	1.366590945	0.981135048	-1.872414697	-0.940908018	-1.542995308
0.28742872	-0.076200131	1.54811783	1.068989454	2.805495197	1.231104057	-1.841251133	-2.043097439	-2.405729116
0.30017658	1.363266426	0.932535795	0.797190307	0.73727458	1.894972964	-2.046793004	-2.417175456	-1.561448192
-0.222112369	0.220192253	0.664698366	1.638874717	2.525024949	1.540191941	-1.849530706	-1.6706949	-2.846644251
0.288631199	0.455105163	1.558638632	0.745259548	1.434187123	0.777447338	-1.863047657	-1.24051501	-2.155706335
0.376328307	0.763711159	2.380455578	0.868320496	1.698265958	1.259299317	-2.13927597	-2.780658066	-2.426446779
0.221569846	0.59191641	1.504662699	1.396095108	1.631905496	1.122085955	-1.884401344	-2.203523346	-2.380310825
1.409448797	0.74874672	1.906044568	0.738303469	0.974863429	0.847191383	-2.089361583	-2.236118981	-2.299117801
0.698575127	0.694978359	0.991376164	1.288566733	1.63691959	1.488604502	-1.732839247	-1.616935806	-2.052095167
0.074181335	0.714913698	2.041197528	0.03863811	1.38185395	1.102824574	-2.133669009	-1.440493053	-1.779447132
0.928123338	0.214022736	0.672422861	0.390969657	0.988803479	0.563298981	-1.649102612	-0.890362337	-1.218176103
1.052461854	0.382569724	1.241571906	0.734698126	1.473418434	1.219593496	-2.011947706	-1.979191242	-2.113174593
0.135907271	0.624990703	0.549342087	1.300977992	1.663226741	1.022445361	-1.847590571	-1.646195635	-1.803103949
0.303473941	0.492432458	2.322186869	0.612228005	2.054799225	1.007472993	-2.51264036	-2.248563921	-2.031389209
0.914660947	0.148540122	1.363098544	0.842574516	1.260846001	0.833417117	-2.29462476	-1.551794543	-1.516717943
0.797123102	0.15518481	0.852907872	1.877357107	2.352223395	1.300827811	-2.520797746	-2.009115642	-2.805710711
0.134826916	0.055596293	0.668412293	1.002396872	1.327673102	1.77004646	-1.854563699	-1.633034431	-1.471353804
0.351122537	0.523844285	2.091141415	0.536716374	1.599806049	1.009772035	-1.715915305	-2.457411656	-1.939075732
0.747102573	1.279510061	1.238891768	1.03692442	0.689588766	0.827569304	-2.16558674	-1.891514312	-1.76248584
0.263153279	0.430369745	2.123658531	1.011732071	2.00358136	1.316668284	-2.737335426	-1.137698431	-3.274129415
0.31393773	0.719332389	1.540100846	0.862477029	1.800845964	1.078251351	-2.339231503	-1.69636493	-2.279348877
0.248101214	1.04962063	1.62389691	0.565344316	1.326338486	1.282824885	-2.200438792	-1.388546774	-2.507140875
0.021596782	-0.170584494	1.828277568	1.466219233	2.387876281	1.670051116	-2.458342672	-2.044065924	-2.70102789
0.637010877	0.147778982	1.093820156	1.194564396	1.171700287	0.761895179	-2.305669338	-1.724738826	-0.976361713
0.657436984	0.983807824	0.653724216	0.928624909	1.627900577	0.191895648	-1.681130053	-1.681130053	-1.681130053
-0.160934245	0.353027738	1.518097426	1.043033481	1.91681587	0.428914108	-1.469052499	-1.469052499	-2.160849378
0.478600898	-0.061948778	1.270125399	1.297164457	1.921794524	1.660002297	-2.017826596	-0.97856014	-2.612150265
0.21011303	0.436427391	1.921231254	0.724809183	2.003239153	0.951427206	-1.98098264	-1.583561495	-2.682703083
0.203638149	0.095564381	1.174332225	0.952925062	1.997181567	1.133781074	-1.485150329	-1.095730222	-2.569265609
0.37351288	0.119497506	1.184370932	1.121217186	3.041511529	1.291737658	-2.271588046	-1.696187308	-2.417046577
0.724552298	1.007167936	1.995427775	0.73570423	1.29612192	1.111757647	-1.91640241	-2.529301788	-2.425027608
0.402965808	0.968470936	2.786266859	0.183734316	1.750023309	0.343650491	-1.6438888	-2.188485933	-1.79680537
0.020399503	-0.10290198	1.527224358	0.397204522	1.768196315	1.680704424	-1.304361734	-1.946158402	-2.040307007
-0.186919375	0.527444907	1.406085094	0.863737659	1.666863431	1.046126007	-1.81564913	-1.689929721	-1.817758872
0.225958081	0.921676794	0.528616651	1.091514947	1.532614032	1.109330486	-1.807338329	-1.566409957	-2.035962704
0.512608956	0.22088743	0.405248483	1.775762333	2.035863845	1.771252486	-2.145678808	-1.976082271	-2.599862454
0.144907638	0.674347667	1.988623285	0.874602149	1.720844435	1.337212823	-2.553929064	-1.693847566	-2.492761368
0.182467506	0.141405139	2.350879697	0.695590887	1.366075958	1.127715953	-2.26615429	-1.769688273	-1.828292578
0.916409758	0.473042215	1.299802508	1.202964373	1.280570187	1.661983873	-2.647754693	-1.771540774	-2.415477447
0.075918261	0.485614966	1.610802439	0.589790851	1.585569781	0.808363007	-2.237319808	-1.628235829	-1.290503668
0.884969738	0.770825931	1.686548648	0.9876327	1.228290922	0.695536385	-2.058189742	-2.033829247	-2.161785336
-0.042567067	0.268564651	2.596193263	0.422004948	1.773142274	1.033287291	-1.678542164	-2.46164701	-1.910436185
-0.056927694	-0.028078098	0.578791318	0.483415323	2.07340874	1.439216783	-1.054093322	-0.751741023	-2.683992027
0.011180739	0.095822379	0.983504882	1.186792156	2.181395044	1.5729627	-1.985218223	-2.171358532	-1.875081145
0.710243877	0.331496987	1.133101729	0.57019744	1.217726544	1.198848645	-2.491233161	-2.179148899	-2.491233161
1.109668439	1.125439733	1.7625766	1.15138754	0.474689653	0.452161999	-2.165099541	-2.125507362	-1.785317061
0.435385086	-0.077998291	0.93286518	1.138699361	2.397655436	1.714533739	-1.502155286	-2.381104551	-1.787110501
-0.027980673	0.298354437	1.700185267	0.684783931	1.852986449	0.922318794	-0.931368	-2.552576477	-1.94670373
0.378249032	-0.130669523	1.56314292	0.561314483	1.253473272	1.501091052	-1.711138477	-2.156273503	-1.259189256
0.992657566	0.647736359	1.432787098	0.17452799	1.47303942	0.402807469	-1.799046724	-1.624998127	-1.699511051
0.282849351	0.581594232	0.609812032	1.2546975	1.960115667	1.061032482	-1.584882556	-1.26143592	-2.338084086
0.42640279	0.392335443	1.639645737	0.711213308	1.924385808	1.403211618	-1.477834025	-2.339654006	-1.826901092
0.358659464	0.812469324	1.596352906	1.157512561	1.990559403	0.802558052	-2.576857584	-1.6155133	-2.525740827
0.199423836	0.193045375	2.122033366	0.428153585	1.337462725	0.826084698	-2.033943908	-1.580344704	-1.491914974
0.825853933	1.466776041	0.94363229	1.003069205	1.772455008	0.734563069	-2.118669485	-1.405923364	-1.570048832
0.809825649	0.421559041	0.695461922	1.134532063	2.047605679	1.165978772	-1.385857095	-1.457551585	-1.811903148
0.164139222	0.581818536	0.527766608	0.243032149	1.661778431	1.83399062	-1.382209968	-1.868666924	-1.761648674
0.251009261	0.240024597	1.037939917	1.250269683	1.303571742	1.225422675	-1.964891169	-1.44360108	-1.899745627
0.489269534	0.376295149	1.554557749	0.826976127	1.607725262	1.05574593	-1.820454087	-1.068533429	-2.043043166
0.549833717	0.572348243	1.910763528	1.26264475	2.192202732	1.42611558	-2.065765905	-1.685079911	-3.063395301
0.094937004	0.839552611	1.129891085	1.141630003	1.736147744	1.245210489	-1.671568	-2.624096016	-1.891704921
0.130493457	0.382391924	0.66382104	1.65787925	1.012966546	1.708208434	-1.634524668	-2.079310829	-1.841925155
0.296772607	-0.029089595	0.866393509	1.177589286	1.992649064	1.48696934	-1.774340346	-1.625940547	-1.797458103
-0.20620323	1.257289461	2.010856344	0.546196866	2.014621395	1.257230969	-1.491474396	-2.331734112	-3.056783298
0.229668442	0.295866536	1.231518679	1.322202565	2.234452423	1.534660448	-2.622145265	-1.665642892	-2.560580936
0.570737505	0.345545737	1.292222409	0.448995408	1.459000855	1.274714949	-2.040773725	-1.871534726	-1.478908413
0.254560526	-0.1507308	0.89169782	1.482799283	2.030316467	1.427933274	-1.700258317	-1.976238229	-1.750958973
1.396052575	0.301886419	1.651168862	0.558967242	1.108885107	0.496362427	-2.05527395	-1.544399864	-1.913648818

AT4G10960.1	GDL62_ARATHGDSL esterase lipase At4g10955 OS=Arabidopsis thaliana GN=At4g10955 PE=2 SV=1
AT5G13820.1	TRP4_ARATHTelomere repeat-binding 4 OS=Arabidopsis thaliana GN=TRP4 PE=1 SV=1
AT2G39050.1	ricin-type beta-trefoil lectin domain [Medicago truncatula]
AT2G47870.1	GRC12_ARATH glutaredoxin-C12 OS=Arabidopsis thaliana GN=GRXC12 PE=3 SV=1
AT5G67310.1	C81D1_ARATHCytochrome P450 81D1 OS=Arabidopsis thaliana GN=CYP81D1 PE=2 SV=1
AT1G27760.3	IFRD1_PIGInterferon-related developmental regulator 1 OS=Sus scrofa GN=IFRD1 PE=2 SV=1
AT4G12290.1	AMO_ARATHPrimary amine oxidase OS=Arabidopsis thaliana GN=At1g62810 PE=2 SV=1
AT3G46670.1	U7E11_ARATHUDP-glycosyltransferase 76E11 OS=Arabidopsis thaliana GN=UGT76E11 PE=2 SV=1
AT4G36830.1	ELO3L_ARATHElongation of fatty acids 3-like OS=Arabidopsis thaliana GN=HOS3 PE=2 SV=1
AT2G36270.1	ABI5_ARATH ABSCISIC ACID-INSENSITIVE 5 OS=Arabidopsis thaliana GN=ABI5 PE=1 SV=1
AT4G02280.1	SUS3_ARATHSucrose synthase 3 OS=Arabidopsis thaliana GN=SUS3 PE=1 SV=1
AT1G24330.1	PUB6_ARATHU-box domain-containing 6 OS=Arabidopsis thaliana GN=PUB6 PE=2 SV=2
AT1G54570.1	Y1457_ARATHAcyltransferase chloroplastic OS=Arabidopsis thaliana GN=At1g54570 PE=2 SV=1
AT4G19960.2	POT9_ARATHPotassium transporter 9 OS=Arabidopsis thaliana GN=POT9 PE=2 SV=2
AT1G14130.1	DAO_ORYSJ2-oxoglutarate-dependent dioxygenase DAO OS=Oryza sativa japonica GN=DAO PE=2 SV=2
AT3G55940.1	PLCD7_ARATHPhosphoinositide phospholipase C 7 OS=Arabidopsis thaliana GN=PLC7 PE=1 SV=1
AT3G08860.1	AGT23_ARATHAlanine--glyoxylate aminotransferase 2 homolog mitochondrial OS=Arabidopsis thaliana GN=At3g08860 PE=2 SV=1
AT4G15490.1	U84A3_ARATHUDP-glycosyltransferase 84A3 OS=Arabidopsis thaliana GN=UGT84A3 PE=1 SV=1
AT5G40850.1	CYSG_ACIADSiroheme synthase OS=Acinetobacter baylyi (strain ATCC 33305 BD413 ADP1) GN=cysG PE=3 SV=1
AT5G14700.1	CCR2_ARATHCinnamoyl- reductase 2 OS=Arabidopsis thaliana GN=CCR2 PE=1 SV=1
AT3G51440.1	SSL6_ARATH STRICTOSIDINE SYNTHASE-LIKE 6 OS=Arabidopsis thaliana GN=SSL6 PE=2 SV=1
AT1G29330.1	ERD2A_ARATHER lumen -retaining receptor A OS=Arabidopsis thaliana GN=ERD2A PE=2 SV=1
AT3G15990.1	SUT34_ARATHProbable sulfate transporter OS=Arabidopsis thaliana GN=SULTR3 4 PE=2 SV=1
AT1G76980.1	unnamed protein product
AT1G70790.2	CAR9_ARATH C2-DOMAIN ABA-RELATED 9 OS=Arabidopsis thaliana GN=CAR9 PE=2 SV=1
AT5G47880.2	ERF1X_ARATHEukaryotic peptide chain release factor subunit 1-1 OS=Arabidopsis thaliana GN=ERF1-1 PE=1 SV=2
AT2G34080.1	SAG39_ORYSJSenescence-specific cysteine protease SAG39 OS=Oryza sativa japonica GN=SAG39 PE=2 SV=2
AT1G20030.1	TLP1_PRUPEThaumatin 1 OS=Prunus persica PE=2 SV=1
AT5G54840.2	SPG1_SCHPOSeptum-promoting GTP-binding 1 OS=Schizosaccharomyces pombe (strain 972 ATCC 24843) GN=spg1 PE=1 SV=1
AT1G10760.1	GWD1_ARATHAlpha-glucan water dikinase chloroplastic OS=Arabidopsis thaliana GN=GWD1 PE=1 SV=2
AT4G05390.1	FNRR1_ARATHFerredoxin--NADP root isozyme chloroplastic OS=Arabidopsis thaliana GN=RFNR1 PE=2 SV=2
AT3G56620.1	WTR27_ARATHWAT1-related At3g56620 OS=Arabidopsis thaliana GN=At3g56620 PE=2 SV=1
AT4G21910.1	DTX39_ARATH DETOXIFICATION 39 OS=Arabidopsis thaliana GN=DTX39 PE=2 SV=1
AT3G23920.1	BAM1_ARATHBeta-amylase chloroplastic OS=Arabidopsis thaliana GN=BAM1 PE=1 SV=1
AT5G61570.1	TMKL1_ARATH kinase TMKL1 OS=Arabidopsis thaliana GN=TMKL1 PE=1 SV=1
AT5G53290.1	CRF3_ARATHEthylene-responsive transcription factor CRF3 OS=Arabidopsis thaliana GN=CRF3 PE=1 SV=1
AT1G69295.1	PDCB4_ARATHPLASMODESMATA CALLOSE-BINDING PROTEIN 4 OS=Arabidopsis thaliana GN=PDCB4 PE=1 SV=1
AT1G72770.1	P2C16_ARATH phosphatase 2C 16 OS=Arabidopsis thaliana GN=HAB1 PE=1 SV=1
AT4G27830.1	BGL10_ARATHBeta-glucosidase 10 OS=Arabidopsis thaliana GN=BGLU10 PE=2 SV=1
AT1G63010.1	SPXM1_ARATHSPX domain-containing membrane At1g63010 OS=Arabidopsis thaliana GN=At1g63010 PE=2 SV=1
AT2G17840.1	senescence dehydration-associated [Medicago truncatula]
AT3G52740.1	PREDICTED: uncharacterized protein LOC103486607
AT2G25530.1	N2B_HAEIR ATPase N2B OS=Haematobia irritans PE=2 SV=1
AT5G35110.1	TMV resistance N-like
AT1G58340.1	DTX48_ARATH DETOXIFICATION 48 OS=Arabidopsis thaliana GN=DTX48 PE=2 SV=1
AT5G43860.1	CLH2_ARATHChlorophyllase- chloroplastic OS=Arabidopsis thaliana GN=CLH2 PE=1 SV=1
AT1G04990.2	C3H3_ARATHZinc finger CCCH domain-containing 3 OS=Arabidopsis thaliana GN=At1g04990 PE=2 SV=1
AT3G21560.1	U84A2_ARATHUDP-glycosyltransferase 84A2 OS=Arabidopsis thaliana GN=UGT84A2 PE=1 SV=1
AT3G24170.1	GSHRC_ARATHGlutathione cytosolic OS=Arabidopsis thaliana GN=At3g24170 PE=2 SV=1
AT1G02850.4	BGL11_ARATHBeta-glucosidase 11 OS=Arabidopsis thaliana GN=BGLU11 PE=2 SV=2
AT3G17609.4	HYH_ARATHTranscription factor HY5-like OS=Arabidopsis thaliana GN=HYH PE=1 SV=1
AT1G01830.1	armadillo repeat-containing 3-like
AT4G24130.1	plant F25P12-18 [Medicago truncatula]
AT5G64230.1	PREDICTED: uncharacterized protein LOC103411982
AT1G55500.3	YTHD2_MOUSEYTH domain-containing family 2 OS=Mus musculus GN=Ythdf2 PE=1 SV=1
AT5G64510.1	nicotiana tabacum ORF [Medicago truncatula]
AT2G16630.1	pollen Ole e I family allergen [Medicago truncatula]
AT2G31560.2	DUF1685 family [Medicago truncatula]
AT1G09510.1	TKPR1_ARATHTetraketide alpha-pyrone reductase 1 OS=Arabidopsis thaliana GN=TKPR1 PE=1 SV=1
AT2G46240.1	BAG6_ARATHBAG family molecular chaperone regulator 6 OS=Arabidopsis thaliana GN=BAG6 PE=1 SV=1
AT1G60610.1	BRG3_ARATHProbable BOI-related E3 ubiquitin- ligase 3 OS=Arabidopsis thaliana GN=BRG3 PE=1 SV=1
AT5G09980.1	PEP4_ARATHElicitor peptide 4 OS=Arabidopsis thaliana GN=PEP4 PE=3 SV=1
AT3G05820.1	INVH_ARATHProbable alkaline neutral invertase chloroplastic OS=Arabidopsis thaliana GN=INVH PE=2 SV=1
AT2G37760.2	AKRC8_ARATHAldo-keto reductase family 4 member C8 OS=Arabidopsis thaliana GN=AKR4C8 PE=1 SV=2
AT3G55640.1	MCFB_DICDIMitochondrial substrate carrier family B OS=Dictyostelium discoideum GN=mcfB PE=3 SV=1
AT3G02875.1	ILR1_ARATHIAA-amino acid hydrolase ILR1 OS=Arabidopsis thaliana GN=ILR1 PE=1 SV=2
AT4G04610.1	APR1_ARATH5 -adenylylsulfate reductase chloroplastic OS=Arabidopsis thaliana GN=APR1 PE=1 SV=2
AT3G08970.1	DNJ63_ARATH ERDJ3A OS=Arabidopsis thaliana GN=ERDJ3A PE=1 SV=1
AT5G13200.1	GEML5_ARATHGEM 5 OS=Arabidopsis thaliana GN=At5g13200 PE=1 SV=1
AT2G19450.1	DGAT1_ARATHDiacylglycerol O-acyltransferase 1 OS=Arabidopsis thaliana GN=DGAT1 PE=1 SV=2
AT5G11110.1	SPSA2_ARATHProbable sucrose-phosphate synthase 2 OS=Arabidopsis thaliana GN=SPS2 PE=1 SV=1
AT2G21620.2	Y1101_SYNY3Universal stress Slr1101 OS=Synechocystis (strain PCC 6803 Kazusa) GN=slr1101 PE=3 SV=1
AT4G18270.1	MRAY_ARATHPhospho-N-acetylmuramoyl-pentapeptide-transferase homolog OS=Arabidopsis thaliana GN=ATTRANS 11 PE=2 SV=3
AT1G62180.1	APR2_ARATH5 -adenylylsulfate reductase chloroplastic OS=Arabidopsis thaliana GN=APR2 PE=1 SV=2
AT1G64950.1	C89A2_ARATHCytochrome P450 89A2 OS=Arabidopsis thaliana GN=CYP89A2 PE=2 SV=2
AT1G72125.1	PTR23_ARATH NRT1 PTR FAMILY OS=Arabidopsis thaliana GN= PE=2 SV=2
AT3G16050.1	PDX12_ARATHPyridoxal 5 -phosphate synthase-like subunit OS=Arabidopsis thaliana GN=PDX12 PE=1 SV=1
AT1G08920.1	ERDL3_ARATHSugar transporter ERD6-like 3 OS=Arabidopsis thaliana GN=SUGTL2 PE=2 SV=1
AT1G22400.1	U85A1_ARATHUDP-glycosyltransferase 85A1 OS=Arabidopsis thaliana GN=UGT85A1 PE=2 SV=1
AT1G62710.1	VPEB_ARATHVacuolar-processing enzyme beta-isozyme OS=Arabidopsis thaliana GN=bVPE PE=2 SV=3
AT4G23630.1	RTNLA_ARATHReticulon B1 OS=Arabidopsis thaliana GN=RTNLB1 PE=1 SV=1
AT5G35320.1	PREDICTED: uncharacterized protein LOC103494211
AT5G55400.1	FIMB3_ARATHFimbrin-3 OS=Arabidopsis thaliana GN=FIM3 PE=3 SV=1
AT1G01720.1	NAC2_ARATHNAC domain-containing 2 OS=Arabidopsis thaliana GN=NAC002 PE=2 SV=2
AT1G79410.1	OCT5_ARATHOrganic cation carnitine transporter 5 OS=Arabidopsis thaliana GN=OCT5 PE=2 SV=1
AT2G15620.1	NIR_ARATHFerredoxin--nitrite chloroplastic OS=Arabidopsis thaliana GN=NIR1 PE=1 SV=1
AT2G23000.1	SCP10_ARATHSerine carboxypeptidase-like 10 OS=Arabidopsis thaliana GN=SCPL10 PE=2 SV=1
AT3G46450.1	SEC14_SCHPOSec14 cytosolic factor OS=Schizosaccharomyces pombe (strain 972 ATCC 24843) GN=sec14 PE=3 SV=1
AT5G52320.1	C96AF_ARATHAlkane hydroxylase MAH1 OS=Arabidopsis thaliana GN=CYP96A15 PE=2 SV=1
AT4G17650.1	CQ10X_DANRECoenzyme Q-binding COQ10 mitochondrial OS=Danio rerio GN=zgc:73324 PE=2 SV=2
AT4G22980.1	MOCOS_BOTFBMolybdenum cofactor sulfurase OS=Botryotinia fuckeliana (strain) GN=hxB PE=3 SV=1
AT4G22592.1	TPPG_ARATHProbable trehalose-phosphate phosphatase G OS=Arabidopsis thaliana GN=TPPG PE=2 SV=1
AT5G51830.1	SCRK7_ARATHProbable fructokinase-7 OS=Arabidopsis thaliana GN=At5g51830 PE=1 SV=1
AT4G11350.1	transferring glycosyl group transferase [Medicago truncatula]
AT2G43018.1	PAO2_ARATHProbable polyamine oxidase 2 OS=Arabidopsis thaliana GN=PAO2 PE=2 SV=1
AT4G27560.1	U79B2_ARATHUDP-glycosyltransferase 79B2 OS=Arabidopsis thaliana GN=UGT79B2 PE=2 SV=1
AT5G11100.1	SYT4_ARATHSynaptotagmin-4 OS=Arabidopsis thaliana GN=SYT4 PE=2 SV=1
AT1G20450.1	ERD10_ARATHDehydrin ERD10 OS=Arabidopsis thaliana GN=ERD10 PE=1 SV=1
AT5G39090.1	PMAT1_ARATHPhenolic glucoside malonyltransferase 1 OS=Arabidopsis thaliana GN=PMAT1 PE=1 SV=1
AT3G50760.1	GATL2_ARATHProbable galacturonosyltransferase-like 2 OS=Arabidopsis thaliana GN=GATL2 PE=2 SV=1
AT2G47800.1	AB4C_ARATHABC transporter C family member 4 OS=Arabidopsis thaliana GN=ABCC4 PE=2 SV=2
AT1G11170.1	lysine ketoglutarate reductase trans-splicing (DUF707)
AT5G20190.1	TPR repeat [Medicago truncatula]
AT3G22200.1	GATP_ARATHGamma-aminobutyrate transaminase mitochondrial OS=Arabidopsis thaliana GN=POP2 PE=1 SV=1
AT4G02410.1	LRK43_ARATHL-type lectin-domain containing receptor kinase OS=Arabidopsis thaliana GN=LECRK43 PE=2 SV=1
AT5G43380.1	PP17_ARATHSerine threonine- phosphatase PP1 isozyme 7 OS=Arabidopsis thaliana GN=TOPP7 PE=2 SV=3
AT4G02940.1	2OG-Fe(II) oxygenase family oxidoreductase [Medicago truncatula]

-0.139200038	-0.109949728	1.467246533	1.510644675	2.267210405	1.205080438	-2.456484407	-1.490053009	-2.254494867
0.195184483	1.067358827	0.944242682	0.643662286	1.427162725	1.071647045	-1.327437364	-2.36505793	-1.656762753
0.035994978	0.731641324	2.519880519	0.758117411	1.933049612	0.926917855	-2.369082644	-2.032536715	-2.50398234
0.774248776	0.875132133	1.68383348	0.293309339	1.596295871	0.052372518	-1.74804969	-1.355783499	-2.171358928
0.646822686	1.180943772	0.637888223	1.536083333	1.415467433	0.989999887	-1.193927744	-2.096130087	-1.823502133
0.571564823	0.844616863	1.50762116	1.039937267	2.123446507	1.433569006	-2.733664611	-2.214678531	-2.572412484
0.03721122	0.512560036	1.29094049	1.28694708	1.649630486	1.188758021	-2.196893994	-1.837103908	-1.932049431
0.517067739	0.302255479	0.985956008	1.041836246	1.845950369	1.236482293	-2.213800597	-1.00471148	-2.711036055
0.244891315	0.103762109	1.294178453	1.290674693	1.503772472	1.418340288	-1.891535557	-2.074995537	-1.889088237
0.198512413	0.8071753	0.630960743	1.093468841	2.761142677	1.461918739	-2.704460933	-1.716652795	-2.532064986
-0.13639875	0.769988689	2.230160621	0.66786928	2.300458392	0.958343971	-2.091648379	-2.149266663	-2.549507161
0.700073128	0.144993704	0.882936651	1.172432166	1.571594735	1.272687109	-1.337381091	-1.529457171	-1.477732977
0.660713552	1.180448245	2.061361632	0.690849941	1.749471885	1.154156649	-2.54249391	-2.318881904	-2.63562609
0.534387286	0.814153465	0.429753344	1.071738468	1.177491754	0.518186033	-1.641358342	-1.031997937	-1.872354071
1.096762563	0.141212664	0.71729477	0.722613948	1.282478162	0.864577646	-1.847846901	-1.227255463	-1.749837387
0.505450372	0.431241081	2.126219762	0.812584295	1.680113076	0.680907949	-1.951717905	-1.599313496	-1.674584391
0.26652686	0.983248876	1.423448832	1.419536307	2.615328343	0.427659625	-3.011813864	-1.957194279	-2.166740701
0.378316317	0.191913692	1.336172987	0.809827235	1.416086781	1.143173326	-1.716999306	-1.576345219	-1.982145813
1.387842108	0.225167371	0.987729689	0.96036397	0.842729456	0.578772194	-1.78584584	-1.593902633	-1.602856314
0.253522613	0.415710659	2.563434548	0.441300762	1.38828944	0.93119574	-2.627065627	-1.640721387	-1.725666749
-0.168041813	0.240718623	1.568485275	0.968648789	2.147815083	1.309338107	-2.051955901	-1.976023214	-2.038984948
-0.005574012	-0.23476116	1.673805158	0.679132422	2.040621291	1.391297487	-1.819309152	-1.919925757	-1.805286277
0.119965101	-0.075506732	0.765249677	1.275409869	1.855914738	1.010452761	-1.982033948	-1.336341496	-1.63310997
-0.086623784	0.863951784	0.971883496	1.082553025	1.424630801	1.122916597	-1.842938881	-1.636455044	-1.899917994
1.544216923	0.347373143	1.129494038	0.641459557	0.936583381	0.233577282	-1.683319159	-1.049654718	-2.099730447
1.419571243	0.150367263	0.965099339	0.240824467	1.232568953	0.465512346	-1.672974498	-1.127994615	-1.672974498
-0.011883674	-0.031495966	0.923923978	1.262112517	1.797798943	1.300099108	-1.464699809	-1.938225771	-1.837629327
0.299223376	-0.190874158	1.267619754	1.110318973	1.920994422	0.91747463	-0.882139708	-1.26378022	-2.580390318
0.542410179	0.447410368	1.22174098	0.826180655	1.329015667	0.525688968	-2.254779132	-0.668649874	-1.96901781
0.579971371	0.458976653	1.044640206	1.5163886	1.564891016	1.135298824	-1.662391155	-1.810551641	-1.667281133
0.848037408	0.085463925	0.923516029	0.361432431	1.388674989	1.287131196	-2.0478733	-1.462827558	-1.383555118
0.573839432	0.101703575	1.46762628	1.020021999	1.2346784	1.287610358	-2.104606476	-1.621494968	-1.959378599
0.335644945	-0.049104271	1.507842308	1.089452228	1.637426101	0.933170372	-2.225190595	-0.561550795	-1.996400402
0.409245677	0.420249087	0.566866401	0.628275282	1.295646879	1.51412519	-1.045860731	-1.475384065	-1.494672366
0.293221012	-0.163505345	1.379291962	0.560591839	1.629021481	1.157761932	-2.531399293	-0.533068328	-1.79191526
0.399452872	0.258329922	1.51480825	1.333105992	2.118031695	1.617019151	-2.271203377	-2.716614526	-2.252929978
0.040764112	0.249868287	1.765584364	0.529008277	1.603476731	0.671375398	-1.458616155	-1.491172358	-1.910288656
0.306327701	0.175165573	1.235088266	0.737425597	2.395962788	1.560555567	-1.866211864	-2.180369146	-1.751289081
0.20957217	0.341972879	0.905241115	1.120305761	1.910386147	1.028291704	-1.94975658	-1.345914767	-2.220098429
0.400732993	0.264791483	1.509677803	1.27907217	2.063101108	0.926440725	-2.352134572	-1.926227346	-2.165454363
-0.127580248	0.89024197	1.760964861	1.127767382	1.70754297	1.200644847	-2.514078826	-1.492262926	-2.553240021
0.110156212	0.157515102	1.568359085	1.069604052	1.716787151	0.965109638	-2.073222817	-1.4128684	-2.101440023
0.497559677	0.257723731	0.731216635	1.019164521	1.727636898	1.362840437	-1.661717351	-1.391470262	-1.547834932
0.334270297	0.441896748	2.265315952	0.826667921	1.543924437	0.386187315	-1.708612486	-1.948687708	-1.472421883
0.302357027	-0.101892444	1.548082352	0.947420596	1.880159095	1.557987913	-1.335502119	-2.355622177	-1.838276189
0.649171036	0.105358386	0.826671861	0.412407376	1.37576027	0.784442274	-1.429311648	-0.78716411	-1.937335445
0.905947173	1.258889165	1.147740475	0.675177356	1.52018593	0.830677733	-2.228911422	-1.849654669	-2.260051741
0.688147435	0.709184662	2.298287421	0.515196378	1.534599178	1.075306206	-2.828822885	-2.143500459	-1.848397936
0.826350772	0.898548638	0.94251437	1.295377486	1.918992713	1.76300279	-1.825466839	-2.20129277	-1.965325617
1.156986499	0.943024013	0.59530642	0.863763742	1.541799784	0.465964007	-1.506889298	-1.673973254	-2.385981913
0.432018992	-0.343343357	1.136461892	0.582377199	1.683516067	1.014157224	-1.657225499	-1.430012413	-1.417950105
0.889370865	0.785097075	0.366510805	0.412735992	2.040300549	0.063964094	-1.312766057	-1.077645182	-2.167568142
0.203633231	0.251784302	2.4057625	0.917650254	1.589982051	0.907263486	-2.937166806	-0.915315525	-2.423593494
0.365844127	0.229699258	1.005119781	1.524349608	2.413682256	1.583823966	-2.096883768	-2.538586733	-2.487048496
1.008204826	0.203688323	1.137584132	0.798926575	1.354243039	1.248169524	-2.405292071	-1.290361007	-2.055163313
0.273028453	0.136018312	0.672026853	1.443726179	1.090193874	1.531303646	-0.954220994	-2.09583867	-1.550180747
0.000340351	0.234964508	2.413567278	0.959638928	1.79127338	0.994114901	-2.503393293	-1.529019101	-2.361486952
0.519613022	0.412554764	2.097013993	0.34233094	1.02567928	0.676177852	-1.652974709	-0.987915293	-2.432479849
1.052647363	0.886819825	1.77767979	1.18420802	1.70835531	1.081750078	-2.265960374	-2.743119585	-2.682380427
-0.2041018	0.846938781	0.501718162	1.447810944	1.651365912	1.431984169	-1.36952541	-2.195198361	-2.110992396
0.604271613	1.115178426	0.740166185	0.994422584	1.06531039	0.648390182	-1.639549565	-1.529533622	-1.998656192
0.522692042	-0.185923686	1.167276392	1.292364022	1.032403357	1.264380621	-2.218632239	-2.289648111	-0.584912398
0.760986094	0.484180107	2.715297544	0.412276605	1.500938009	0.951498916	-1.826015027	-1.516545048	-1.960645013
0.821650548	0.179439679	1.657892262	0.163967719	1.244674731	0.381286247	-1.557421959	-0.622081753	-2.269407474
0.283870482	0.298762411	0.749138427	1.155162971	1.765110964	1.241890855	-1.861709048	-1.63451995	-1.997707112
0.738847639	0.640076655	0.901142453	0.763136532	1.521447532	1.614170101	-1.722815332	-2.470778454	-1.985227126
0.729776816	0.873316877	1.350342663	1.237776269	1.148899243	1.313934893	-2.884471012	-2.322161774	-1.447413975
-0.120076571	0.729963165	1.786898613	1.060663603	0.905911085	1.317621526	-1.664996396	-2.126434867	-1.889550158
0.364010505	0.992184476	1.862941121	0.495753509	1.330353016	0.601654447	-1.7042569	-1.92269673	-2.019943445
1.031910828	0.624882646	1.106006733	0.93627688	1.516830406	1.074150758	-2.070835731	-1.79930782	-2.4199147
0.282795987	0.426676401	1.208216343	1.48797368	2.163939359	1.429812158	-2.080457513	-2.112110715	-2.241253725
0.797621713	1.031122015	1.581653201	0.730043881	1.488787043	-0.137317097	-1.718393434	-1.462548202	-2.310969119
-0.110179772	0.115724902	1.619500471	1.096749071	1.823376056	0.892172856	-2.520942616	-1.558855453	-2.357545514
0.75926467	0.34959975	1.75380595	1.040644634	1.424837499	1.3806474	-2.461477663	-1.935281487	-2.312040752
0.145890095	0.135922728	1.7845987	0.325470469	1.822556647	1.21153046	-1.736650534	-2.03476019	-1.654558375
0.740135554	1.221357006	0.961822329	0.51483993	0.817952403	0.343402165	-1.509556724	-1.44076595	-1.649186714
0.394629884	0.35189108	1.530546126	1.259370825	0.548578622	1.44765347	-1.887962801	-2.310974899	-1.333732309
0.516553047	0.76787647	1.176797769	0.214787953	1.04023316	0.680449304	-1.617536455	-0.923720924	-1.855440325
0.798534763	0.351908182	0.727596229	0.490974544	1.138805729	1.264609759	-1.597345478	-1.524577037	-1.650506691
-0.071824116	0.239009945	2.12703566	1.115767552	1.741578985	0.36269046	-2.440230337	-1.24418291	-1.829845239
0.026282258	0.311310436	2.073936235	0.341869519	1.424438467	0.886662191	-1.967733396	-1.569365603	-1.527400108
0.292712375	0.206717694	1.1100946	1.531473626	1.408309498	1.276723931	-2.097148968	-2.047242554	-1.6816402
0.469785876	0.376618749	1.345604681	1.071069813	1.592590546	0.832021616	-1.789770233	-1.193959248	-1.764390048
-0.026315096	-0.13342445	0.701459544	1.157339412	1.877573739	1.50385944	-1.161307455	-2.051150136	-1.868034999
0.3863837	-0.418603832	1.409884585	0.90826543	1.809462127	1.268862652	-2.265212659	-1.435978357	-1.663063647
0.397819913	0.103486923	0.995088308	0.948525634	1.328936421	1.422670475	-1.902312625	-1.608722932	-1.685492118
0.489369431	0.482266588	2.2591348	0.664315753	1.671794227	0.808971783	-2.374610395	-1.524314372	-2.476927817
0.278773774	0.571082188	2.177749493	0.645003447	1.46354449	0.947937303	-1.785670226	-1.957277802	-1.783595119
0.416528052	-0.078068181	1.178304693	1.183165095	1.514784818	1.546153219	-1.653095703	-1.728585903	-1.546129986
0.182917586	0.280137423	1.912806125	0.318741035	1.331616345	0.959994606	-1.74443577	-1.586155117	-1.655622234
0.090119892	-0.190943899	1.650473503	0.606015205	1.903132617	1.713643561	-2.073655441	-1.69545471	-2.00333026
0.074181335	0.714913698	2.041197528	0.03863811	1.38185395	1.102824574	-2.133669009	-1.440493053	-1.779447132
0.960357261	-0.002455544	1.433296158	0.832384139	1.013558752	1.052749788	-1.97650656	-1.565876034	-1.747507959
0.279401961	0.536818392	2.089987906	0.60689367	1.513086131	1.250554416	-2.035741597	-1.886792751	-1.795404206
0.344845948	0.359883822	1.516022728	1.270951946	1.066458174	1.715323039	-1.934335933	-2.020880627	-2.318269078
0.511597197	0.5864867	1.990993737	1.414328298	1.24404891	1.831285845	-2.931439539	-2.436165341	-2.211135807
0.965521838	0.927655269	0.647008049	0.500358814	0.909132763	0.90710917	-1.742167772	-1.281509586	-1.833108545
0.749586473	0.34992116	1.067686044	1.700823621	2.418178877	1.178035697	-2.058617955	-1.254638426	-2.651802545
0.241190815	0.768426843	1.702794156	0.836178069	1.231760535	1.106895532	-2.003698913	-1.854656062	-1.546509345
0.302238607	0.113699864	1.239053685	0.711747101	1.042630501	0.910624798	-1.709893133	-1.815975559	-0.794125863
-0.170093233	0.513826949	0.869583607	1.459861363	1.870385112	1.49123232	-1.912399808	-2.251069578	-1.871326732
0.437570129	0.492357549	1.015910217	0.928565016	2.851372866	1.41539703	-2.426581346	-2.089120094	-2.625471367
0.48493115	0.302946902	1.921534457	0.37972709	1.07190356	1.1185243	-2.083054587	-1.637473902	-1.55903897
0.33106663	0.226192871	1.601292689	0.943779056	2.038284462	1.11029206	-1.823510035	-1.815676903	-1.94958757
0.318741385	0.165813756	2.094877443	0.444890984	2.043436246	1.442541048	-2.145787419	-2.394201514	-1.33282916
0.798816077	0.429586545	1.208801159	1.064466152	1.233660307	1.188255919	-2.07315694	-2.254731666	-1.595697552
0.549936081	0.871321137	1.846168779	1.028591153	1.685335931	1.205766684	-2.796293595	-2.008118153	-2.382708016

AT5G55400.1	FIMB3_ARATHFimbrin-3 OS=Arabidopsis thaliana GN=FIM3 PE=3 SV=1
AT5G55400.1	FIMB3_ARATHFimbrin-3 OS=Arabidopsis thaliana GN=FIM3 PE=3 SV=1
AT4G00440.1	phosphatidylinositol N-acetyglucosaminlytransferase subunit P [Medicago truncatula]
AT1G78610.1	MSL6_ARATHMechanosensitive ion channel 6 OS=Arabidopsis thaliana GN=MSL6 PE=1 SV=1
AT1G29640.1	senescence regulator (Protein of unknown function, DUF584)
AT5G61820.1	stress up-regulated Nod 19 [Medicago truncatula]
AT2G25625.2	unnamed protein product
AT4G17550.1	GLPT4_ARATH glycerol-3-phosphate transporter 4 OS=Arabidopsis thaliana GN=At4g17550 PE=3 SV=2
AT4G34131.1	U73B3_ARATHUDP-glycosyltransferase 73B3 OS=Arabidopsis thaliana GN=UGT73B3 PE=2 SV=1
AT5G24155.1	ERG11_BRANASqualene monooxygenase 1,1 OS=Brassica napus GN=SQP1,1 PE=2 SV=1
AT1G01470.1	LEA14_ARATHProbable desiccation-related LEA14 OS=Arabidopsis thaliana GN=LEA14 PE=1 SV=1
AT1G70780.1	Y4276_ARATHUncharacterized protein At4g22758 OS=Arabidopsis thaliana GN=At4g22758 PE=2 SV=1
AT3G20300.1	extracellular ligand-gated ion channel [Medicago truncatula]
AT1G07500.1	hypothetical protein AT1G07500
AT3G23210.1	BH034_ARATHTranscription factor bHLH34 OS=Arabidopsis thaliana GN=BHLH34 PE=2 SV=1
AT3G51450.1	SSL7_ARATH STRICTOSIDINE SYNTHASE-LIKE 7 OS=Arabidopsis thaliana GN=SSL7 PE=2 SV=1
**Cluster 46**	**Description**
AT5G29000.2	PHL1_ARATH PHR1-LIKE 1 OS=Arabidopsis thaliana GN=PHL1 PE=1 SV=1
AT1G12650.4	RRP36_NEMVERibosomal RNA processing 36 homolog OS=Nematostella vectensis GN=v1g245966 PE=3 SV=1
AT5G40850.2	CYSG_ACIADSiroheme synthase OS=Acinetobacter baylyi (strain ATCC 33305 BD413 ADP1) GN=cysG PE=3 SV=1
AT2G43500.2	NLP8_ARATH NLP8 OS=Arabidopsis thaliana GN=NLP8 PE=2 SV=1
**Cluster 47**	**Description**
AT2G41240.1	BH100_ARATHTranscription factor bHLH100 OS=Arabidopsis thaliana GN=BHLH100 PE=2 SV=1
AT4G35770.2	STR15_ARATHRhodanese-like domain-containing chloroplastic OS=Arabidopsis thaliana GN=STR15 PE=2 SV=1
**Cluster 48**	**Description**
AT4G37520.2	PER50_ARATHPeroxidase 50 OS=Arabidopsis thaliana GN=PER50 PE=1 SV=1
AT2G40140.2	C3H29_ARATHZinc finger CCCH domain-containing 29 OS=Arabidopsis thaliana GN=At2g40140 PE=2 SV=1
**Cluster 49**	**Description**
AT3G16670.1	pollen Ole e I family allergen [Medicago truncatula]
AT4G36690.3	U2A2A_ARATHSplicing factor U2af large subunit A OS=Arabidopsis thaliana GN=U2AF65A PE=2 SV=2
AT5G65930.2	KCBP_ARATHKinesin-like calmodulin-binding OS=Arabidopsis thaliana GN=KCBP PE=1 SV=1
AT4G21730.1	NSF_ARATHVesicle-fusing ATPase OS=Arabidopsis thaliana GN=NSF PE=2 SV=2
AT4G21910.4	DTX39_ARATH DETOXIFICATION 39 OS=Arabidopsis thaliana GN=DTX39 PE=2 SV=1
AT1G73870.1	COL7_ARATHZinc finger CONSTANS-LIKE 7 OS=Arabidopsis thaliana GN=COL7 PE=2 SV=1
AT3G05730.1	DF205_ARATHDefensin 205 OS=Arabidopsis thaliana GN=At3g05730 PE=3 SV=1
AT1G52710.1	CX5BL_ARATH cytochrome c oxidase subunit 5b-like OS=Arabidopsis thaliana GN=At1g52710 PE=2 SV=2
AT4G28150.1	plant F9H3-4 [Medicago truncatula]
AT1G29600.1	C3H10_ARATH zinc finger CCCH domain-containing 10 OS=Arabidopsis thaliana GN=At1g29600 At1g29610 PE=3 SV=2
**Cluster 50**	**Description**
AT4G34135.1	U73B2_ARATHUDP-glucosyl transferase 73B2 OS=Arabidopsis thaliana GN=UGT73B2 PE=1 SV=1
AT1G51780.1	ILL5_ARATHIAA-amino acid hydrolase ILR1-like 5 OS=Arabidopsis thaliana GN=ILL5 PE=3 SV=1
AT3G09350.3	FES1_ASPTNHsp70 nucleotide exchange factor fes1 OS=Aspergillus terreus (strain NIH 2624 FGSC A1156) GN=fes1 PE=3 SV=1
AT4G36010.2	TP1A_MALDOThaumatin 1a OS=Malus domestica GN=TL1 PE=1 SV=1
AT2G26150.2	HSFA2_ARATHHeat stress transcription factor A-2 OS=Arabidopsis thaliana GN=HSFA2 PE=1 SV=1
AT5G22140.2	AIFA_DICDIApoptosis-inducing factor homolog A OS=Dictyostelium discoideum GN=aifA PE=3 SV=1
AT3G09350.1	FES1_EMENIHsp70 nucleotide exchange factor fes1 OS=Emericella nidulans (strain FGSC A4 ATCC 38163 CBS NRRL 194 M139) GN=fes1 PE=3 SV=1
AT5G52640.1	HS901_ARATHHeat shock 90-1 OS=Arabidopsis thaliana GN=HSP90-1 PE=1 SV=3
AT1G59860.1	kDa class I heat shock OS=Arabidopsis thaliana GN= PE=1 SV=1
AT1G20560.2	AAE1_ARATHProbable acyl-activating enzyme peroxisomal OS=Arabidopsis thaliana GN=AAE1 PE=2 SV=1
AT5G51440.1	kDa heat shock mitochondrial OS=Arabidopsis thaliana GN= PE=2 SV=1
AT1G71520.1	ERF20_ARATHEthylene-responsive transcription factor ERF020 OS=Arabidopsis thaliana GN=ERF020 PE=2 SV=1
AT4G25200.1	kDa heat shock mitochondrial OS=Arabidopsis thaliana GN= PE=2 SV=1
AT3G09440.2	HSP7C_ARATHHeat shock 70 kDa 3 OS=Arabidopsis thaliana GN=HSP70-3 PE=1 SV=1
**Cluster 51**	**Description**
AT2G32870.1	UBP13_ARATHUbiquitin carboxyl-terminal hydrolase 13 OS=Arabidopsis thaliana GN=UBP13 PE=1 SV=1
AT5G45820.1	CIPKK_ARATHCBL-interacting serine threonine- kinase 20 OS=Arabidopsis thaliana GN=CIPK20 PE=1 SV=1
AT5G64040.1	PSAN_ARATHPhotosystem I reaction center subunit chloroplastic OS=Arabidopsis thaliana GN=PSAN PE=1 SV=2
AT2G34420.1	CB1C_ARATHChlorophyll a-b binding chloroplastic OS=Arabidopsis thaliana GN= PE=1 SV=1
AT3G01500.3	BCA1_ARATHBeta carbonic anhydrase chloroplastic OS=Arabidopsis thaliana GN=BCA1 PE=1 SV=2
AT1G79790.1	ACD10_HUMANAcyl- dehydrogenase family member 10 OS=Homo sapiens GN=ACAD10 PE=1 SV=1
AT1G21500.1	hypothetical protein ARALYDRAFT_472386
AT5G54270.1	CB23_SOLLCChlorophyll a-b binding chloroplastic OS=Solanum lycopersicum GN=CAB13 PE=1 SV=1
AT3G15353.2	MT3_ARATHMetallothionein 3 OS=Arabidopsis thaliana GN=MT3 PE=1 SV=1
AT3G08940.2	CB4B_ARATHChlorophyll a-b binding chloroplastic OS=Arabidopsis thaliana GN= PE=1 SV=1
AT5G38420.1	RBS2B_ARATHRibulose bisphosphate carboxylase small chain chloroplastic OS=Arabidopsis thaliana GN=RBCS-2B PE=2 SV=2
AT1G66100.1	THN24_ARATHProbable thionin- OS=Arabidopsis thaliana GN=At1g66100 PE=2 SV=1
AT5G02160.1	PREDICTED: uncharacterized protein LOC103433437
AT4G38240.1	MGAT1_ARATHAlpha-1,3-mannosyl-glyco 2-beta-N-acetylglucosaminyltransferase OS=Arabidopsis thaliana GN=GNTI PE=1 SV=1
AT4G26530.1	ALF_ARATHFructose-bisphosphate cytoplasmic isozyme OS=Arabidopsis thaliana GN=At4g26520 PE=2 SV=2
AT3G54890.1	CAB6_ARATHChlorophyll a-b binding chloroplastic OS=Arabidopsis thaliana GN=LHCA1 PE=1 SV=1
AT5G09660.1	MDHG1_ARATHMalate glyoxysomal OS=Arabidopsis thaliana GN=At5g09660 PE=2 SV=1
AT2G39470.1	PNSL1_ARATHPhotosynthetic NDH subunit of lumenal location chloroplastic OS=Arabidopsis thaliana GN=PNSL1 PE=1 SV=2
AT1G51402.1	PST2_ARATHPhotosystem II 5 kDa chloroplastic OS=Arabidopsis thaliana GN=PSBT PE=3 SV=2
AT5G36790.3	PGP1B_ARATHPhosphoglycolate phosphatase chloroplastic OS=Arabidopsis thaliana GN=PGLP1B PE=1 SV=1
AT3G54500.2	dentin sialophospho [Medicago truncatula]
AT3G47470.1	CA4_ARATHChlorophyll a-b binding chloroplastic OS=Arabidopsis thaliana GN=LHCA4 PE=1 SV=1
AT1G75690.1	LQY1_ARATH disulfide-isomerase chloroplastic OS=Arabidopsis thaliana GN=LQY1 PE=1 SV=1
AT2G05070.1	CB21_GOSHIChlorophyll a-b binding chloroplastic OS=Gossypium hirsutum GN=CAB-151 PE=2 SV=2
AT4G05180.1	PSBQ2_ARATHOxygen-evolving enhancer 3- chloroplastic OS=Arabidopsis thaliana GN=PSBQ2 PE=1 SV=2
AT1G29910.1	CB1B_ARATHChlorophyll a-b binding chloroplastic OS=Arabidopsis thaliana GN= PE=1 SV=2
AT3G21870.1	CCU11_ARATHCyclin-U1-1 OS=Arabidopsis thaliana GN=CYCU1-1 PE=1 SV=1
AT4G25080.1	CHLM_ARATHMagnesium protoporphyrin IX chloroplastic OS=Arabidopsis thaliana GN=CHLM PE=1 SV=1
AT2G05100.1	CB21_GOSHIChlorophyll a-b binding chloroplastic OS=Gossypium hirsutum GN=CAB-151 PE=2 SV=2
AT1G09340.1	CP41B_ARATHChloroplast stem-loop binding of 41 kDa chloroplastic OS=Arabidopsis thaliana GN=CSP41B PE=1 SV=1
AT1G29920.1	CB1B_ARATHChlorophyll a-b binding chloroplastic OS=Arabidopsis thaliana GN= PE=1 SV=2
AT1G70760.1	NDHL_ARATHNAD(P)H-quinone oxidoreductase subunit chloroplastic OS=Arabidopsis thaliana GN=ndhL PE=2 SV=1
**Cluster 52**	**Description**
AT3G22190.1	IQD1_ARATH IQ-DOMAIN 1 OS=Arabidopsis thaliana GN=IQD1 PE=1 SV=1
AT4G16880.1	RPP4_ARATHDisease resistance RPP4 OS=Arabidopsis thaliana GN=RPP4 PE=1 SV=1
AT3G61590.1	FBK77_ARATHF-box kelch-repeat At3g61590 OS=Arabidopsis thaliana GN=At3g61590 PE=1 SV=1
AT5G52030.1	traB domain-containing isoform X1
AT1G21630.2	EHD2_ARATHEH domain-containing 2 OS=Arabidopsis thaliana GN=EHD2 PE=1 SV=1
AT2G47180.1	GOLS1_ARATHGalactinol synthase 1 OS=Arabidopsis thaliana GN=GOLS1 PE=1 SV=1
AT5G64210.1	AOX2_ARATHUbiquinol oxidase mitochondrial OS=Arabidopsis thaliana GN=AOX2 PE=1 SV=2
AT1G18120.1	GDL3_ARATHGDSL esterase lipase At1g18120 OS=Arabidopsis thaliana GN=At1g18120 PE=3 SV=1
AT1G27360.4	SPL11_ARATHSquamosa promoter-binding 11 OS=Arabidopsis thaliana GN=SPL11 PE=2 SV=2
AT1G10060.3	BCAT1_ARATHBranched-chain-amino-acid aminotransferase mitochondrial OS=Arabidopsis thaliana GN=BCAT1 PE=1 SV=2
AT2G26980.3	CIPK3_ARATHCBL-interacting serine threonine- kinase 3 OS=Arabidopsis thaliana GN=CIPK3 PE=1 SV=2
AT1G75170.3	RSC5_DICDIRandom slug 5 OS=Dictyostelium discoideum GN=rsc5 PE=2 SV=1
AT1G73920.1	LICH_CROAD lysosomal acid lipase cholesteryl ester hydrolase OS=Crotalus adamanteus PE=2 SV=1
AT5G50950.1	FUM2_ARATHFumarate hydratase chloroplastic OS=Arabidopsis thaliana GN=FUM2 PE=2 SV=1
AT4G14690.1	ELIP2_ARATHEarly light-induced chloroplastic OS=Arabidopsis thaliana GN=ELIP2 PE=1 SV=1
AT1G77800.1	BRPF1_HUMANPeregrin OS=Homo sapiens GN=BRPF1 PE=1 SV=2
AT4G32590.2	PNSB3_ARATHPhotosynthetic NDH subunit of subcomplex B chloroplastic OS=Arabidopsis thaliana GN=PNSB3 PE=2 SV=1
AT5G43930.4	AMRA1_MOUSEActivating molecule in BECN1-regulated autophagy 1 OS=Mus musculus GN=Ambra1 PE=1 SV=1
AT2G35390.1	KPRS1_ARATHRibose-phosphate pyrophosphokinase chloroplastic OS=Arabidopsis thaliana GN=PRS1 PE=2 SV=2
AT1G50030.1	TOR_ARATHSerine threonine- kinase TOR OS=Arabidopsis thaliana GN=TOR PE=1 SV=1

0.469785876	0.376618749	1.345604681	1.071069813	1.592590546	0.83202161[illegible]	-1.789770233	-1.193959248	-1.764390048
0.559876378	0.915220658	0.800783889	0.982008461	1.564127774	1.260953808	-1.314135685	-0.671687689	-2.097147593
0.408810166	0.532325525	1.545304256	0.853812855	1.298750032	1.02038364	-2.018091923	-1.082320052	-1.741354167
-0.155620537	0.35536123	1.957838352	1.06798825	1.809863984	1.214023236	-1.985510692	-2.019745334	-2.244198489
0.812852678	0.367211939	2.515611575	0.482566451	1.62790662	0.618715614	-1.904766787	-2.298234269	-2.221863821
0.398684252	0.130903376	1.39371475	1.100350227	2.250804048	1.614224972	-2.165708799	-2.326682036	-2.396290791
-0.109293286	0.111961142	1.338345932	0.974003869	2.372278571	1.026464278	-1.6015104	-2.440342017	-1.671908087
-0.344981468	0.191863075	2.240653244	0.5930377	2.400566154	1.578856315	-1.969743892	-2.468341718	-2.22190941
0.929717954	0.648378844	1.654811359	0.785582111	1.216473037	0.867334194	-2.091071609	-2.15658902	-1.85463687
0.136564543	-0.217621858	2.15045889	0.700346487	1.193805523	0.822947284	-2.156708746	-0.608922099	-2.020870024
-0.049818546	0.036358577	1.287924173	1.391903021	2.383135974	1.398474373	-1.931407167	-1.809437177	-2.707133226
1.409448797	0.74874672	1.906044568	0.738303469	0.974863429	0.847191383	-2.089361583	-2.236118981	-2.299117801
-0.346426212	0.096162265	2.339458909	0.466225268	1.783347463	1.114827229	-1.552148321	-2.243965979	-1.657480623
0.696054169	-0.045077584	2.084132552	0.605246124	2.340642116	1.14488224	-1.75653947	-2.30766518	-1.369566629
-0.068645933	0.360186805	1.47197876	0.879659941	1.32007625	1.558315391	-1.954107331	-1.656244507	-1.911219375
0.655568415	0.589669567	0.812967628	1.294064835	1.150284071	1.908721536	-2.104052332	-2.412245673	-1.894978047

WT-C	KO-C	OE-C	WT-2h	KO-2h	OE-2h	WT-12h	KO-12h	OE-12h
-1.386823281	-1.528985029	-2.058791069	1.793064288	3.074899586	2.3401352	-1.247618689	-1.099635235	-2.14421583
-2.149035513	-2.673388731	-2.149035513	2.451055796	2.512942055	2.058857339	-2.149035513	-2.149035513	-1.100101868
-1.168841803	-1.302200158	-1.955875914	1.948345092	2.91091846	2.558551819	-1.949765379	-2.581830355	-1.063702078
-2.063460999	-1.543454943	-2.019416667	2.619224653	3.2010379	2.883690819	-2.050534825	-2.063460999	-2.050534825

WT-C	KO-C	OE-C	WT-2h	KO-2h	OE-2h	WT-12h	KO-12h	OE-12h
-3.387628496	-2.384026259	-3.387628496	-1.207162334	-0.754662371	-0.601328801	3.600419902	3.684884667	4.234474586
-1.284010534	-2.711282697	-4.160183648	-1.487758306	-2.204126996	-0.25984814	4.980934888	3.404941284	4.721334149

WT-C	KO-C	OE-C	WT-2h	KO-2h	OE-2h	WT-12h	KO-12h	OE-12h
2.906331763	2.613933321	2.265700902	-2.613933321	-2.613933321	-2.613933321	1.776046417	2.427791531	3.611264474
2.707742857	2.318968691	1.86586699	-2.318968691	-2.318968691	-2.318968691	1.434849752	2.259487031	3.007928136

WT-C	KO-C	OE-C	WT-2h	KO-2h	OE-2h	WT-12h	KO-12h	OE-12h
-1.800174701	-2.421733452	-2.041598695	2.402761218	1.459405882	0.653423461	0.452855969	1.783529352	0.417242904
-1.397525419	-1.228700793	-2.534876649	1.456141423	1.777463376	0.739252677	0.362242061	0.492658269	0.057829179
-2.09593738	-2.032434438	-1.790258637	1.272970623	1.588769168	1.158656663	0.144682249	0.964110003	0.789441748
-2.127279369	-1.569010287	-1.971357064	1.280541566	1.41252452	0.71018849	0.538192242	0.775225006	-0.457013638
-2.529832245	-1.870400204	-2.39556794	2.318325596	1.996893123	1.388654713	0.388949952	0.667602992	0.56321896
-2.482641324	-2.457813187	-1.692656225	1.76034938	1.203658338	0.992737277	0.642810163	0.90515818	1.128397397
-1.439913061	-2.986685651	-3.361957918	2.145930418	1.005817387	1.167289555	0.462029075	1.415334321	1.592155875
-1.529410938	-1.646757808	-2.266810169	1.173997041	1.662807299	-0.022848342	0.408384188	0.593823018	-0.432006165
-1.53078961	-3.014931701	-3.014931701	1.770880374	1.854347506	1.207331902	0.421957886	1.195067446	-0.406595551
-2.038007445	-2.295524846	-2.493529007	1.528722188	1.8758678	0.311658621	0.301263385	2.193829822	0.715719483

WT-C	KO-C	OE-C	WT-2h	KO-2h	OE-2h	WT-12h	KO-12h	OE-12h
1.508130694	0.867230952	1.103458952	1.518526874	2.161567271	1.438323147	-3.439965378	-2.363023666	-2.794248846
0.540394784	1.202356353	0.756632406	2.391950757	2.163479532	3.202011782	-3.21899492	-3.206394883	-3.137001548
0.529468509	1.178552335	1.374874946	3.256103399	1.889063903	2.925623285	-2.647317092	-2.278262979	-2.895941055
1.236786464	1.979053868	1.693980849	1.843860047	2.571950551	1.715001304	-1.570508509	-3.352078054	-2.730084822
0.925997464	1.132562409	0.507378426	3.112859796	1.531849126	2.868922009	-2.522724361	-2.595215735	-3.016371695
2.056279088	0.541981141	0.381414324	2.436341753	0.459538026	2.204724614	-2.408375579	-2.295306626	-2.613768092
0.744433034	0.402547788	0.584811509	2.097330302	1.06430741	1.773705532	-1.863125717	-2.910145891	-1.893863968
0.981380565	1.078548123	0.579901416	2.070120879	0.468489692	1.980518139	-2.066181907	-2.737497556	-2.355279352
0.874281848	0.788262874	0.91211026	2.273443352	0.674997317	2.150762367	-2.219836807	-2.102114848	-2.430675001
2.091210636	1.53587561	0.051055386	1.611332465	2.363288365	1.637412392	-3.541581043	-3.541581043	-2.207012767
1.850264671	1.262167775	0.669348311	2.453124333	0.244544639	2.038962994	-2.897478921	-3.082616829	-2.538316974
1.188735909	0.597913839	0.915364238	1.76074581	2.516358833	2.177246648	-3.845634046	-2.790664375	-2.520066855
1.597601408	0.875670209	0.461879724	2.059831871	1.052129299	2.225003848	-2.945636998	-1.91228547	-3.083597058
0.766933387	1.833461172	0.079577236	2.223055461	0.453680042	2.405056721	-2.814760431	-3.008242704	-1.938760885

WT-C	KO-C	OE-C	WT-2h	KO-2h	OE-2h	WT-12h	KO-12h	OE-12h
-2.712213733	-2.232645574	-1.288950276	0.453156584	1.370969453	0.990192314	1.064076956	1.526204559	0.829209716
-2.047991309	-2.297373885	-2.396805577	1.138238563	0.493351869	1.361197898	1.351075305	0.861591111	1.536716024
-1.827489837	-3.376913531	-1.771077361	0.718733306	0.922256069	0.970225666	1.392068325	1.714795132	1.25740223
-2.317092277	-3.383494527	-1.642929038	0.760635839	1.218343499	0.693212262	1.568083726	1.598925259	1.504315257
-2.823512531	-2.345139853	-1.468401775	0.546008519	0.934655719	1.290122654	1.106188268	1.796285956	0.963793053
-2.428104645	-2.612342599	-1.471873399	0.370793873	0.502439166	0.505868312	1.587052032	2.012825813	1.533341446
-1.933192487	-3.132293569	-2.47200886	0.724234825	1.163977619	0.683233564	1.429886632	1.875124879	1.247176834
-2.908014869	-3.652153213	-2.077915494	0.487884555	0.800707741	0.889105387	1.908517911	2.471613543	2.080254439
-1.894756317	-2.94066277	-2.243086413	0.752593195	0.981990054	1.255437746	0.947257287	1.515159497	0.820787037
-2.54225405	-3.429627692	-2.659388988	0.183191846	0.290154815	0.53805276	2.44868783	2.79931462	2.371868859
-2.504467928	-2.73555142	-2.850951638	1.244185033	1.139862813	0.746074388	1.266006245	2.327848434	1.366994072
-2.630918787	-2.727620675	-2.575813157	1.512640439	1.160542468	1.62523564	1.514206515	1.042991404	1.078736133
-1.930773429	-2.939362946	-2.148751199	0.482133947	1.04176623	0.840664358	1.863525806	1.65209967	1.138697562
-2.464340185	-2.50418045	-2.50418045	0.833530642	1.678273497	0.892527071	1.479953145	1.455218785	1.133197945
-2.368918063	-2.349511209	-2.392325976	1.422124014	1.284859802	1.152561788	1.21859062	1.099136952	0.933482072
-2.414575002	-2.565472289	-1.482518313	0.586602207	0.902434168	0.898999262	1.162410652	1.623092374	1.28902694
-2.216254494	-2.264651209	-1.462000626	0.614314226	0.856920841	0.898410387	1.271232699	1.296190206	1.00583797
-1.993737618	-2.203274014	-2.263898591	0.553024356	1.050188586	0.992399878	1.14930569	1.514927137	1.201064576
-1.725228072	-2.049789223	-3.186041446	0.804271124	1.239161993	0.62943695	1.337389322	1.186823978	1.763975374
-2.265588147	-3.177854775	-2.650784439	1.031988456	1.963210102	1.940504951	1.400480174	0.997989148	0.760054531
-2.597762545	-2.111504518	-2.189717501	1.19061397	1.327204927	0.819765594	1.570021132	1.116789431	0.87458951
-2.540982131	-3.073042816	-1.285589183	0.276382882	0.732436522	0.737421462	1.703937346	2.151937171	1.297498747
-1.991684382	-2.750853995	-2.06709989	1.01691744	1.019020935	0.836136355	1.358660279	1.378679862	1.200223395
-2.865671469	-3.874188084	-2.510777141	0.275632515	0.440155243	0.509123506	2.529374607	3.256031378	2.240319445
-2.020855936	-3.147593889	-1.578259574	0.492517446	1.028486364	0.937677044	1.035676475	2.075640864	1.176711205
-2.474829197	-3.463749765	-1.836350852	0.989193975	1.088746828	0.530745974	1.157134219	2.220085934	1.789022883
-1.999604634	-2.609585191	-2.338536963	0.441368999	1.407939817	0.872242043	1.626383409	1.072321241	1.527471279
-3.603201869	-3.603201869	-1.300151784	0.368295702	1.221820495	0.871428857	2.871947882	2.5769062	0.596156386
-2.708472274	-3.400689095	-2.212122229	0.348481785	0.464669093	0.486399746	2.293232768	2.847442929	1.881057277
-2.23847339	-2.488728071	-1.851860022	0.001105596	0.48405408	1.279477418	1.596453756	1.797370373	1.420600259
-3.453715243	-3.419822806	-2.236984934	0.632104434	1.027950728	0.66761977	2.007313758	2.617821854	2.157712439
-2.277661903	-2.606649288	-1.734229538	0.807094737	1.532851295	0.973772421	0.904536106	1.373253692	1.027032478

WT-C	KO-C	OE-C	WT-2h	KO-2h	OE-2h	WT-12h	KO-12h	OE-12h
-1.764655559	0.153791256	-0.71679592	1.553374288	2.058236417	0.593454873	0.584943373	-0.954032923	-1.508315805
-0.800738434	-1.159516728	-0.664752036	1.693739465	1.52492399	1.644913981	-0.634055239	-1.146413991	-0.458101007
-1.181683479	-0.268987261	-0.511998299	0.78689042	0.937759721	1.166187883	-0.051735778	-0.512455499	-0.363977708
0.088272775	-0.096427633	-0.912594316	1.38707438	2.23323014	1.33359745	0.430115468	-2.231634132	-2.231634132
-1.381293445	-0.395231707	-0.649241371	0.87722548	1.366953086	0.69184126	0.098971677	-1.114656802	-0.285031592
-0.885763931	-1.01641685	-1.241875059	1.817109267	1.591764739	2.039193152	-0.058201946	-1.328344148	-0.917465225
-0.724171136	-0.416776318	-1.099349615	1.264382167	2.494123678	1.543120922	-0.981654572	-1.099349615	-0.980325512
-0.57497614	-0.511473198	-0.57497614	0.78116767	1.488181618	1.11700461	-0.57497614	-0.57497614	-0.57497614
-1.421440641	-0.119267841	-0.675557952	1.315597087	0.945091405	1.044272679	-0.233039716	-0.956772374	0.101117352
-0.35599025	-0.543166584	-0.578013932	1.011529466	1.506561543	1.041795917	-0.711308214	-0.644923313	-0.726484633
-1.630046282	-0.432754505	-1.218068583	0.389183829	2.344902954	2.421042299	-0.928144338	-1.333886179	-0.477737565
-0.743640989	-0.743640989	-0.743640989	0.967853917	2.435232969	1.058759051	-0.743640989	-0.743640989	-0.743640989
-0.597869035	-0.466814684	-1.021448439	1.248538641	1.774068829	1.227079532	-0.335563797	-0.524401955	-1.303589092
-1.430004181	-0.056007366	-0.749666764	2.237433399	1.787046491	1.029554273	-0.290789313	-0.519212148	-2.008354391
-1.600908051	-1.136575824	-0.447313765	0.61501882	2.226669151	2.355260728	-0.353196794	-0.921158844	-0.737795421
-1.066139649	-0.141669859	-0.720015649	1.129870211	1.532528237	0.810616251	-0.195725276	-0.749255269	-0.600208997
-1.101878607	-1.101878607	-1.101878607	1.602107997	2.541053962	1.516594996	-1.101878607	-1.015094841	-0.237147685
-0.921088183	-0.764996242	-0.7693708	1.218454486	1.054733441	0.826206528	0.041914959	-1.142528645	-1.073318027
-0.347848954	-0.214903178	-1.063206916	1.251943693	1.604821647	1.263152256	-0.849725903	-0.816999647	-0.827232998
-1.587040339	-0.857840094	-0.113202835	1.136064282	1.122021088	0.661281569	-0.338718431	-0.808340223	-0.929905204

AT3G53780.2	RBL4_ARATHRHOMBOID 4 OS=Arabidopsis thaliana GN=RBL4 PE=2 SV=1
AT1G45249.2	AI5L5_ARATHABSCISIC ACID-INSENSITIVE 5 5 OS=Arabidopsis thaliana GN=ABF2 PE=1 SV=1
AT3G08690.2	UBC11_ARATHUbiquitin-conjugating enzyme E2 11 OS=Arabidopsis thaliana GN=UBC11 PE=1 SV=2
AT1G23450.1	PP121_ARATHPentatricopeptide repeat-containing chloroplastic OS=Arabidopsis thaliana GN=PCMP-E69 PE=3 SV=1
AT2G16990.1	BMR1_BACSUMultidrug resistance 1 OS=Bacillus subtilis (strain 168) GN=bmr PE=3 SV=2
AT1G67140.1	HTR5B_XENTRHEAT repeat-containing 5B OS=Xenopus tropicalis GN=heatr5b PE=2 SV=1
AT3G53090.2	UPL7_ARATHE3 ubiquitin- ligase UPL7 OS=Arabidopsis thaliana GN=UPL7 PE=2 SV=1
AT1G80160.2	lactoylglutathione lyase glyoxalase I family [Medicago truncatula]
AT5G62130.2	PGAP3_XENTRPost-GPI attachment to s factor 3 OS=Xenopus tropicalis GN=pgap3 PE=2 SV=1
AT5G03240.2	UBQ3_ARATHPolyubiquitin 3 OS=Arabidopsis thaliana GN=UBQ3 PE=1 SV=1
AT1G78600.2	BBX22_ARATHB-box zinc finger 22 OS=Arabidopsis thaliana GN=BBX22 PE=1 SV=2
AT3G58070.1	GIS_ARATHZinc finger GIS OS=Arabidopsis thaliana GN=GIS PE=2 SV=1
AT1G62610.3	GSXL3_ARATHFlavin-containing monooxygenase FMO GS-OX-like 3 OS=Arabidopsis thaliana GN=At1g62620 PE=2 SV=2
AT4G21060.2	B3GTK_ARATHProbable beta-1,3-galactosyltransferase 20 OS=Arabidopsis thaliana GN=B3GALT20 PE=2 SV=1
AT3G02140.1	AFP4_ARATHNinja-family AFP4 OS=Arabidopsis thaliana GN=AFP4 PE=1 SV=1
AT3G10420.1	SP3AA_BACSUStage III sporulation AA OS=Bacillus subtilis (strain 168) GN=spoIIIAA PE=4 SV=1
AT3G58640.1	CTR1_ARATHSerine threonine- kinase CTR1 OS=Arabidopsis thaliana GN=CTR1 PE=1 SV=1
AT1G55500.1	YTHD2_MOUSEYTH domain-containing family 2 OS=Mus musculus GN=Ythdf2 PE=1 SV=1
AT5G53120.2	SPSY_ARATHSpermine synthase OS=Arabidopsis thaliana GN=SPMS PE=1 SV=1
AT1G29950.2	BH144_ARATHTranscription factor bHLH144 OS=Arabidopsis thaliana GN=BHLH144 PE=1 SV=1
AT5G50670.1	SP13B_ARATHSquamosa promoter-binding 13B OS=Arabidopsis thaliana GN=SPL13B PE=3 SV=1
AT1G77760.1	NIA1_ARATHNitrate reductase
AT2G46375.1	glycosyltransferase family 28 [Medicago truncatula]
AT1G30410.1	AB12C_ARATHABC transporter C family member 12 OS=Arabidopsis thaliana GN=ABCC12 PE=2 SV=1
AT1G18830.1	SC31A_ARATH transport SEC31 homolog A OS=Arabidopsis thaliana GN=SEC31A PE=1 SV=1
AT4G27410.3	NAC72_ARATHNAC domain-containing 72 OS=Arabidopsis thaliana GN=NAC072 PE=2 SV=1
AT4G25450.2	AB28B_ARATHABC transporter B family member 28 OS=Arabidopsis thaliana GN=ABCB28 PE=2 SV=1
AT5G55470.1	NHX4_ARATHSodium hydrogen exchanger 4 OS=Arabidopsis thaliana GN=NHX4 PE=2 SV=2
AT5G59210.2	synaptonemal complex 1 isoform X1 [Cucumis melo]
AT5G01850.1	HT1_ARATHSerine threonine- kinase HT1 OS=Arabidopsis thaliana GN=HT1 PE=1 SV=1
AT5G52570.2	BCH2_ARATHBeta-carotene 3-hydroxylase chloroplastic OS=Arabidopsis thaliana GN=BETA-OHASE 2 PE=2 SV=1
AT5G15950.1	DCAM2_ARATHS-adenosylmethionine decarboxylase proenzyme 2 OS=Arabidopsis thaliana GN=SAMDC2 PE=2 SV=1
AT2G02710.1	TLOV1_ARATH TWIN LOV 1 OS=Arabidopsis thaliana GN=TLP1 PE=1 SV=2
AT5G02950.1	ATM_ARATHSerine threonine- kinase ATM OS=Arabidopsis thaliana GN=ATM PE=2 SV=1
AT1G55980.1	ULP2A_ARATHProbable ubiquitin-like-specific protease 2A OS=Arabidopsis thaliana GN=ULP2A PE=2 SV=2
AT5G07680.1	NC100_ARATHNAC domain-containing 100 OS=Arabidopsis thaliana GN=NAC100 PE=2 SV=1
AT5G16840.3	BPA1_ARATHBinding partner of ACD11 1 OS=Arabidopsis thaliana GN=BPA1 PE=1 SV=1
AT1G71960.1	AB25G_ARATHABC transporter G family member 25 OS=Arabidopsis thaliana GN=ABCG25 PE=2 SV=1
AT2G44280.1	MFS12_HUMANMajor facilitator superfamily domain-containing 12 OS=Homo sapiens GN=MFSD12 PE=1 SV=2
AT5G66600.1	electron [Medicago truncatula]
AT1G18750.1	AGL65_ARATHAgamous-like MADS-box AGL65 OS=Arabidopsis thaliana GN=AGL65 PE=1 SV=1
AT1G29952.1	BH144_ARATHTranscription factor bHLH144 OS=Arabidopsis thaliana GN=BHLH144 PE=1 SV=1
AT1G07640.3	DOF11_ARATHDof zinc finger OS=Arabidopsis thaliana GN= PE=1 SV=2
AT1G72670.1	IQD1_ARATH IQ-DOMAIN 1 OS=Arabidopsis thaliana GN=IQD1 PE=1 SV=1
AT5G15600.1	SP1L4_ARATH SPIRAL1-like 4 OS=Arabidopsis thaliana GN=SP1L4 PE=2 SV=1
AT3G58510.2	RH11_ARATHDEAD-box ATP-dependent RNA helicase 11 OS=Arabidopsis thaliana GN=RH11 PE=1 SV=1
AT3G02410.1	ICML2_ARATHProbable isoprenylcysteine alpha-carbonyl methylesterase ICMEL2 OS=Arabidopsis thaliana GN=ICMEL2 PE=2 SV=1
AT2G14170.1	MMSA_ARATHMethylmalonate-semialdehyde dehydrogenase
**Cluster 53**	**Description**
AT4G33985.1	DUF1685 family [Medicago truncatula]
AT2G21170.2	TPIC_ARATHTriosephosphate chloroplastic OS=Arabidopsis thaliana GN=TIM PE=1 SV=1
AT1G06475.1	transmembrane protein
AT1G17665.1	CA-responsive protein
AT3G46970.1	PHS2_ARATHAlpha-glucan phosphorylase cytosolic OS=Arabidopsis thaliana GN=PHS2 PE=1 SV=1
AT2G41730.1	Hop-interacting [Medicago truncatula]
AT1G28050.1	COL15_ARATHZinc finger CONSTANS-LIKE 15 OS=Arabidopsis thaliana GN=COL15 PE=2 SV=1
AT5G55050.1	GDL87_ARATHGDSL esterase lipase At5g55050 OS=Arabidopsis thaliana GN=At5g55050 PE=2 SV=1
AT3G53600.1	ZAT11_ARATHZinc finger ZAT11 OS=Arabidopsis thaliana GN=ZAT11 PE=2 SV=1
AT4G33980.1	At4g33980 [Brassica napus]
AT2G21580.2	RS252_ARATH40S ribosomal S25-2 OS=Arabidopsis thaliana GN=RPS25B PE=2 SV=1
AT1G47510.2	IP5PB_ARATHType IV inositol polyphosphate 5-phosphatase 11 OS=Arabidopsis thaliana GN=IP5P11 PE=1 SV=1
AT5G24640.1	Hop-interacting [Medicago truncatula]
AT5G23240.1	DNAJ_CALS4Chaperone OS=Caldanaerobacter subterraneus tengcongensis (strain DSM 15242 JCM 11007 NBRC 100824 MB4) GN=dnaJ PE=3 SV=1
AT5G48250.1	COL10_ARATHZinc finger CONSTANS-LIKE 10 OS=Arabidopsis thaliana GN=COL10 PE=1 SV=1
AT4G34950.1	NFD4_ARATH NUCLEAR FUSION DEFECTIVE 4 OS=Arabidopsis thaliana GN=NFD4 PE=3 SV=1
AT1G66240.3	ATOX1_ARATHCopper transport ATX1 OS=Arabidopsis thaliana GN=ATX1 PE=1 SV=2
AT1G66570.1	SUC7_ARATHSucrose transport SUC7 OS=Arabidopsis thaliana GN=SUC7 PE=2 SV=2
AT3G46640.2	PCL1_ARATHTranscription factor LUX OS=Arabidopsis thaliana GN=LUX PE=1 SV=1
AT2G28900.1	OP161_ARATHOuter envelope pore 16- chloroplastic OS=Arabidopsis thaliana GN=OEP161 PE=1 SV=1
AT1G68050.1	ADO3_ARATHAdagio 3 OS=Arabidopsis thaliana GN=ADO3 PE=1 SV=1
**Cluster 54**	**Description**
AT5G20250.4	RFS6_ARATHProbable galactinol--sucrose galactosyltransferase 6 OS=Arabidopsis thaliana GN=RFS6 PE=2 SV=2
AT2G41100.4	CML12_ARATHCalmodulin 12 OS=Arabidopsis thaliana GN=CML12 PE=1 SV=3
**Cluster 55**	**Description**
AT1G07280.2	Tetratricopeptide repeat (TPR)-like superfamily
AT5G46610.3	ALMTE_ARATHAluminum-activated malate transporter 14 OS=Arabidopsis thaliana GN=ALMT14 PE=2 SV=1
AT4G28460.1	unnamed protein product
AT3G19615.1	PREDICTED: uncharacterized protein LOC103494810
AT4G14240.2	Y4424_ARATHDUF21 domain-containing At4g14240 OS=Arabidopsis thaliana GN=CBSDUF1 PE=1 SV=1
AT1G54830.2	NFYC3_ARATHNuclear transcription factor Y subunit C-3 OS=Arabidopsis thaliana GN=NFYC3 PE=2 SV=1
AT1G04870.1	ANM10_ARATH arginine N-methyltransferase PRMT10 OS=Arabidopsis thaliana GN=PRMT10 PE=1 SV=1
**Cluster 56**	**Description**
AT5G43650.1	BH092_ARATHTranscription factor bHLH92 OS=Arabidopsis thaliana GN=BHLH92 PE=2 SV=1
AT5G13490.2	ADT2_ARATHADP,ATP carrier mitochondrial OS=Arabidopsis thaliana GN=AAC2 PE=2 SV=2
AT1G70800.1	CAR6_ARATH C2-DOMAIN ABA-RELATED 6 OS=Arabidopsis thaliana GN=CAR6 PE=1 SV=1
AT5G02580.1	PREDICTED: uncharacterized protein LOC103433368
**Cluster 57**	**Description**
AT1G10070.3	BCAT2_ARATHBranched-chain-amino-acid aminotransferase chloroplastic OS=Arabidopsis thaliana GN=BCAT2 PE=1 SV=1
AT1G36060.1	ERF55_ARATHEthylene-responsive transcription factor ERF055 OS=Arabidopsis thaliana GN=ERF055 PE=2 SV=1
AT1G19530.1	DNA polymerase epsilon catalytic subunit A
AT1G10070.2	BCAT2_ARATHBranched-chain-amino-acid aminotransferase chloroplastic OS=Arabidopsis thaliana GN=BCAT2 PE=1 SV=1
AT4G36670.1	PLT6_ARATHProbable polyol transporter 6 OS=Arabidopsis thaliana GN=PLT6 PE=2 SV=2
AT2G47270.1	BH151_ARATHTranscription factor UPBEAT1 OS=Arabidopsis thaliana GN=UPB1 PE=2 SV=1
AT1G08630.2	THA1_ARATHProbable low-specificity L-threonine aldolase 1 OS=Arabidopsis thaliana GN=THA1 PE=1 SV=1
AT4G26260.2	MIOX4_ARATHInositol oxygenase 4 OS=Arabidopsis thaliana GN=MIOX4 PE=2 SV=1
AT1G15040.1	NTPR_ENTHA OS=Enterococcus hirae (strain ATCC 9790 DSM 20160 JCM 8729 LMG 6399 NBRC 3181 NCIMB 6459 NCDO 1258) GN=ntpR PE=3 SV=2
AT1G15380.1	GLOD5_XENTRGlyoxalase domain-containing 5 OS=Xenopus tropicalis GN=glod5 PE=2 SV=1
**Cluster 58**	**Description**
AT3G13460.4	YTHD2_MOUSEYTH domain-containing family 2 OS=Mus musculus GN=Ythdf2 PE=1 SV=1
AT3G13040.1	PHL1_ARATH PHR1-LIKE 1 OS=Arabidopsis thaliana GN=PHL1 PE=1 SV=1
AT5G60170.2	CNOT4_MOUSECCR4-NOT transcription complex subunit 4 OS=Mus musculus GN=Cnot4 PE=1 SV=2
AT4G13020.5	MHK_ARATHSerine threonine- kinase MHK OS=Arabidopsis thaliana GN=MHK PE=2 SV=2
AT2G37500.1	ARGJ_ARATHArginine biosynthesis bifunctional chloroplastic OS=Arabidopsis thaliana GN=At2g37500 PE=1 SV=2
AT1G69420.2	ZDHC1_ARATHProbable S-acyltransferase 22 OS=Arabidopsis thaliana GN=PAT22 PE=2 SV=2
AT2G02800.2	APK2B_ARATH kinase chloroplastic OS=Arabidopsis thaliana GN=APK2B PE=2 SV=1
AT1G04080.1	PRP39_XENLAPre-mRNA-processing factor 39 OS=Xenopus laevis GN=prpf39 PE=2 SV=1
AT2G29650.1	ANTR1_ARATHSodium-dependent phosphate transport chloroplastic OS=Arabidopsis thaliana GN=ANTR1 PE=1 SV=1

-0.63813627	0.068922807	-0.012821002	1.069364246	1.926746475	1.478190899	-1.550291907	-1.812332006	-0.529643151
-0.92690922	-0.65574468	-0.174786613	1.57497774	2.019167248	0.931210035	-0.285075342	-1.538990086	-0.943849082
-0.359392351	-0.120505416	-1.238987902	1.772104604	1.298986712	1.520933695	-0.692186396	-1.354284069	-0.826668879
-0.787326954	-0.151667212	-0.64268434	1.239619617	2.04756354	0.8108431	-0.637094039	-0.634704811	-1.2445489
-0.949066757	-0.154598726	-0.949066757	0.423885341	2.486828484	1.092176225	-0.361221748	-0.949066757	-0.949066757
-0.886743519	-0.556202728	-0.768041904	0.947440631	0.790921349	0.483283608	-0.320716208	-0.53417651	-0.752136975
-1.399082359	-0.168033285	-0.84102655	0.890933082	1.317425988	1.090945036	-0.354979076	-0.330878047	-0.205304788
0.166119716	-0.409638411	-0.556202728	1.089452747	1.913475843	0.964162747	0.209088135	-2.047445207	-2.148289663
-0.682411147	-0.235791888	-0.885890031	1.521879468	1.315563701	1.172465006	-1.074841004	-0.810233321	-0.320740784
-0.52113047	0.0713607	-0.688356033	1.828042181	1.970100326	1.241846875	-1.660199409	-1.25152341	-0.99014076
-0.608540835	-0.197810944	-0.534164927	0.602095663	1.86802184	1.068416242	-0.557828472	-0.242013885	-1.398174682
-0.764141962	-0.36563855	-0.489821749	1.85101213	1.496706233	0.651117678	-0.812484788	-0.57806548	-0.988683512
-1.438400426	-0.784227518	-0.174450314	1.70632967	1.543487011	0.259458101	-0.549167956	-0.560188907	-1.571294697
-0.68684559	-0.376368642	-0.778290642	0.830246917	1.282911119	1.303934369	-0.389348786	-0.160397158	-1.025841587
-0.79629804	-0.061124732	-0.413174907	1.079328984	2.230227124	1.547467699	-0.796734978	-1.974803909	-0.814887242
-1.103041373	-0.241610796	-0.990353558	1.575097125	2.195036428	0.687266613	-0.035394522	-1.462570357	-0.624429559
-1.505904227	-0.622144135	-0.326468338	0.807972354	0.943653659	0.893004329	-0.303541436	-0.879368397	-0.25149208
-0.744326143	-0.046397926	-1.199554715	1.390927544	1.470832442	1.516997271	-0.794651592	-0.781634707	-0.812192174
0.272697096	-0.572961332	-1.891445553	1.192448572	1.276554571	1.867070835	0.492604253	-1.891445553	-1.891445553
-0.304823775	-0.199665365	-0.919375825	1.46873839	2.170631613	1.488686504	-1.819324811	-1.296984383	-0.46487265
-1.364177302	-0.620084603	-0.275781936	1.035397015	1.229822028	1.539887022	-0.780311099	-0.916892387	-1.088027943
-0.853138995	-0.37565637	-0.60662532	1.310800891	2.00799101	1.558555571	-0.802427723	-1.365008546	-0.874490519
-0.284392493	-0.593638505	-0.354970544	1.45549516	1.454082	0.705190892	-0.587804712	-0.896980899	-0.896980899
-0.417105267	-0.36530354	-0.966592616	1.771868123	1.500123616	1.449116238	-0.71915237	-1.331596946	-0.92135724
-0.86948565	0.03500332	-0.97542836	0.814471966	1.223061232	1.199605998	-0.116076153	-0.689543015	-0.621609339
-1.35440724	0.014508504	-0.247296435	0.687295563	1.919574165	1.344430561	0.514224333	-1.279174814	-1.599154637
-0.253186837	-0.803998926	-0.168633041	0.826889098	1.754465416	0.841917307	-0.433975345	-0.584690584	-1.178787087
-0.912121265	-0.474123986	-0.711437424	1.232268644	2.204055887	0.793988144	-0.948460459	-0.261085099	-0.923084443
-0.107317936	-0.571702383	-1.296984383	0.946380043	1.179138037	1.236828114	0.064222508	-1.296984383	-1.296984383
-0.890846048	-0.373188361	-0.720044526	1.802901104	1.765730583	1.251211822	-0.885196134	-0.835813858	-1.114754582
-0.268963974	-0.26179297	-1.290393479	1.297797161	2.253801379	2.413302507	-0.973792609	-1.889922185	-1.280035828
-1.816743447	-0.570234899	-0.164567273	1.240718737	1.846050571	1.967987239	-0.838554067	-1.030985894	-0.633670967
-0.245903174	-0.622413176	-0.570179825	0.66626775	2.240908819	1.062987161	-0.53295767	-1.064799148	-0.933910738
-0.855920651	-0.677185441	-0.752966029	1.102329497	1.600028851	1.261259318	-0.329166842	-0.521362754	-0.827015949
-1.218015032	-0.081539191	-0.972667127	1.357288871	1.458172228	0.518196999	0.29749954	-0.692434596	-0.666501692
-0.557508089	-0.050768946	-1.349372276	1.692679254	2.318698237	1.907613023	-1.328522186	-1.682646515	-0.950172503
-1.292141133	-0.496962084	-1.292141133	1.352868751	2.101412356	0.376885632	-0.840435709	-1.292141133	-1.292141133
-0.86149605	-0.415822114	-0.826500073	1.241103041	1.919145276	1.350702928	-0.687188661	-0.856408071	-0.863536277
-0.738121557	-0.368683347	-0.472039285	1.608518713	2.09327302	1.403440813	-1.160453135	-1.773041541	-0.59289368
-0.908840765	0.05092939	-0.305718895	0.633664937	0.969296002	1.117605216	-0.431681554	-0.908840765	-0.216413567
-1.095880894	-0.25968787	-0.371696906	0.883660479	1.246595776	0.979173343	-0.632969043	-0.499211861	-0.769360663
-0.304823775	-0.199665365	-0.919375825	1.46873839	2.170631613	1.488686504	-1.819324811	-1.819324811	-0.46487265
-0.463813287	-0.310154694	-0.558539157	0.798749837	1.755691317	1.301864001	-0.626536759	-0.756059982	-1.761510665
-0.837671783	-0.258669081	-0.423590449	0.837449906	1.8923312	1.236939351	-0.9198341	-0.688858377	-0.838096667
-1.056119458	-0.848117253	-0.886724875	1.331016649	2.610443712	1.48086845	-0.726875979	-0.753717156	-1.15077409
-1.102531188	-0.916458987	-0.227537549	1.061933311	1.064505534	0.725287836	-0.233054554	-1.102531188	-1.102531188
-1.268197884	-0.144531687	-1.268197884	1.095533899	2.789165738	1.270836819	-1.268197884	-1.268197884	0.061786766
-0.982027486	-0.625835417	-0.622506315	0.38534358	2.077327792	1.462109471	-0.982027486	-0.982027486	-0.982027486
**WT-C**	**KO-C**	**OE-C**	**WT-2h**	**KO-2h**	**OE-2h**	**WT-12h**	**KO-12h**	**OE-12h**
1.687531595	1.369483294	2.594949159	-0.558676661	0.20380209	-0.23776106	-1.439228109	-2.06805674	-1.552043568
1.813448499	1.421014898	2.791186802	0.613472284	-1.521405771	-0.5534994	-1.521405771	-1.521405771	-1.521405771
1.676751964	1.458978419	3.183077576	-0.507600393	0.158559046	-0.437039141	-2.424714192	-1.26709231	-1.84092097
1.990239779	1.538093099	2.748296595	-0.638274392	-0.063736644	-0.5136913	-1.9459556	-1.585355862	-1.529615677
1.030056468	1.469540354	3.017074746	0.229580427	-0.178792539	0.152142535	-1.90098106	-1.95356153	-1.865059401
2.601524521	2.105961667	3.842010134	0.190865968	-1.146964347	-0.689470206	-3.246837479	-1.94962838	-1.707461878
1.440690351	1.660931542	2.302347356	-0.196815213	0.049346806	0.060572152	-1.967002591	-1.84546326	-1.504607142
1.449816937	2.055789193	3.005731469	-0.70132028	-0.626247548	0.062258688	-2.029089253	-1.653281417	-1.56365779
1.50394824	1.747776774	3.286888702	0.354816834	-0.242423996	0.174796428	-1.786040765	-2.371852659	-1.994910462
2.967314057	2.324716553	2.88345263	-1.379203112	-0.184881456	-0.094661433	-2.32326494	-2.092138636	-2.101333665
1.018365221	1.928090849	3.324898341	0.658966477	-0.451786478	-0.33771571	-1.515156207	-1.745512172	-1.88015032
2.417548484	1.650830854	3.186536863	0.400728319	-0.805801435	-0.819273555	-1.67685651	-1.67685651	-1.67685651
2.6448479	1.960476443	4.272910063	-0.280020325	-1.570982026	-1.175402386	-2.99951952	-1.197312451	-1.654997697
2.098746302	2.882098968	3.487566877	-0.663037856	0.288188041	0.150148615	-2.394714166	-2.825658056	-3.023338724
1.550046592	1.655418356	2.683161019	-0.31350205	0.273755097	-0.093152383	-2.127057997	-1.384899627	-2.243769007
2.033535463	1.565467378	2.77841063	-0.400930939	-0.442128847	-0.129035266	-1.540827812	-1.641947666	-2.222542941
1.184594345	1.465460303	3.201947796	0.800803732	-1.193662333	-0.317252984	-1.550562425	-0.87080171	-1.720526724
1.981748917	1.913703262	3.56096531	-1.555319661	0.132811592	-0.159783905	-1.958041838	-1.958041838	-1.958041838
2.317024207	1.616370033	2.503359204	-0.705825874	-0.235649282	-0.452328502	-1.754697446	-1.846720729	-1.44153161
2.02909237	1.361082263	3.06112399	-0.117553136	0.359949007	-0.580072684	-2.336449065	-1.214726022	-2.562446723
1.652865224	2.682007696	4.294015155	-0.880916996	-0.063397148	-0.235073283	-2.591580321	-2.224123886	-2.633796441
**WT-C**	**KO-C**	**OE-C**	**WT-2h**	**KO-2h**	**OE-2h**	**WT-12h**	**KO-12h**	**OE-12h**
-4.110335327	-2.857591679	-2.280378047	-2.373746465	-2.244452683	0.850972049	3.917190569	3.490729109	3.607612473
-2.344234178	-3.094485462	-2.344234178	-2.344234178	-1.852226721	0.935088532	2.384557075	2.221119279	2.594945375
**WT-C**	**KO-C**	**OE-C**	**WT-2h**	**KO-2h**	**OE-2h**	**WT-12h**	**KO-12h**	**OE-12h**
2.566390131	2.316043105	2.880712181	-1.700096597	-1.700096597	-1.700096597	-1.448528977	-1.700096597	-1.485769948
2.403292149	2.771449503	3.790041584	-0.983936571	-1.462131829	-1.16935008	-1.462131829	-1.462131829	-1.425101098
2.239322003	2.116903418	3.605845223	-1.535485442	-2.436293226	-1.635364573	-1.115391413	-1.266225743	-1.026689751
1.936688372	2.483774487	3.044156789	-1.244145968	-1.681726036	-1.251503991	-1.276678253	-1.926417861	-0.624959552
1.364218618	1.842091162	2.270417676	-1.835342618	-1.919406883	-1.44348376	-1.1739544	-0.729740436	-0.617668777
1.438015672	2.192618967	2.72194916	-1.436387867	-1.436387867	-1.403974652	-1.436387867	-0.280638634	-0.66756216
2.46344771	1.942759669	3.216509346	-0.650975708	-2.779269109	-1.312769147	-1.437283362	-1.200571872	-0.86738582
**WT-C**	**KO-C**	**OE-C**	**WT-2h**	**KO-2h**	**OE-2h**	**WT-12h**	**KO-12h**	**OE-12h**
2.701307096	2.561922785	3.477182344	0.054465973	-0.975281986	-0.146578942	-2.999106225	-2.752407429	-2.721503616
2.806315136	2.411640179	3.379108071	-0.100340691	-1.063689723	-0.546089278	-3.763460152	-2.990212282	-2.625449816
2.928260342	2.093991174	3.014057582	-0.139274525	-0.07300577	-0.291642258	-3.193436272	-2.453566409	-2.885383863
2.14343105	2.684460969	3.552510097	-0.259574379	-0.340350741	-0.250758382	-3.870875547	-3.069679257	-2.58916381
**WT-C**	**KO-C**	**OE-C**	**WT-2h**	**KO-2h**	**OE-2h**	**WT-12h**	**KO-12h**	**OE-12h**
-1.849753532	-0.913162702	-0.624478602	-0.36845459	-1.849753532	-0.24283759	3.654580836	4.085917073	3.279776783
-0.403527603	-0.931421291	-1.610667551	-0.378106784	-1.357851132	-1.38733557	3.530569243	3.973707641	3.512048329
-1.579363575	-1.889444164	-1.995122242	0.656790503	-1.092855673	-1.446192472	4.273312152	3.468552581	3.541428054
-1.405699068	-1.656143433	-1.461634809	-0.857074117	-0.733878344	-1.776653886	4.167222435	4.112149237	3.611708286
-0.501777245	-1.320773284	-1.0602918	-1.56660538	-1.775797032	-1.594086116	3.261759135	3.704028435	3.853543288
-0.896683017	-1.952470795	-2.311720517	0.473192561	-0.673846485	-0.25860918	3.008672862	4.171187095	3.440277476
-1.710950955	-2.058935169	-1.470091492	-0.20398699	-0.562462267	0.015218538	3.735172321	3.350720472	3.353714833
-0.924762108	-1.080412978	-1.034201713	-0.580523851	-0.925933602	-0.505048121	2.976574991	4.012883797	3.871911783
-0.31882491	-1.760339408	-1.703553382	-0.886827937	-1.807434525	-1.269656855	4.023927655	3.665585531	4.388294893
-1.672910805	-1.507018682	-1.61807415	0.1329432	-1.162442224	0.143849728	3.562302659	4.053804044	3.175154318
**WT-C**	**KO-C**	**OE-C**	**WT-2h**	**KO-2h**	**OE-2h**	**WT-12h**	**KO-12h**	**OE-12h**
-2.931031486	-3.204113436	-3.029827697	0.596820807	-0.451154237	1.102611758	0.958612405	0.215134936	0.275064685
-2.674029148	-2.513760547	-2.763885967	0.533058687	1.336386091	0.870080479	0.180509555	-0.213888762	-0.282242322
-3.338163712	-2.501493604	-3.211012567	1.477770297	1.326290684	0.994114572	-0.009255459	-0.181524401	0.018261848
-3.594216134	-3.122884467	-3.150079178	0.784295489	1.359700918	0.888374041	0.247958183	0.550972189	-0.209889975
-3.283388434	-3.305416133	-3.226434065	0.521387943	0.495766206	0.402103254	0.117832428	0.261344221	-0.046895816
-2.249087399	-3.112961613	-2.3632651	0.673681324	0.712906278	0.55496616	0.014548005	-0.363903533	0.180662452
-3.441161323	-2.705738237	-2.463419112	0.472733359	1.491750243	0.87418753	-0.792925994	-0.055592236	0.281851073
-3.834894568	-3.098073251	-2.140643911	1.089347869	1.510856618	1.088492347	-0.187004477	0.249254759	0.041376792
-2.870672757	-3.014619211	-2.512073904	0.931651626	1.446890802	1.531585107	-0.586901289	-0.060980818	0.110972636

AT4G18020.3	APRR2_ARATHTwo-component response regulator-like APRR2 OS=Arabidopsis thaliana GN=APRR2 PE=2 SV=2
AT3G03790.2	IBTK_XENLAInhibitor of Bruton tyrosine kinase OS=Xenopus laevis GN=ibtk PE=2 SV=1
AT3G12570.3	HSP20-like chaperone superfamily [Medicago truncatula]
AT3G14010.1	CID4_ARATHPolyadenylate-binding -interacting 4 OS=Arabidopsis thaliana GN=CID4 PE=2 SV=1
AT1G01320.1	TSS_ARATH TSS OS=Arabidopsis thaliana GN=TSS PE=1 SV=1
AT1G73350.2	PREDICTED: uncharacterized protein LOC103484557
AT2G31370.1	POF21_ARATHProbable transcription factor 21 OS=Arabidopsis thaliana GN=POSF21 PE=2 SV=1
AT2G47850.1	C3H32_ARATHZinc finger CCCH domain-containing 32 OS=Arabidopsis thaliana GN=At2g47850 PE=2 SV=2
AT1G53190.1	HIP1_ORYSJProbable E3 ubiquitin- ligase HIP1 OS=Oryza sativa japonica GN=HIP1 PE=1 SV=2
AT4G02570.2	CUL1_ARATHCullin-1 OS=Arabidopsis thaliana GN=CUL1 PE=1 SV=1
AT3G25585.1	AAPT2_ARATHCholine ethanolaminephosphotransferase 2 OS=Arabidopsis thaliana GN=AAPT2 PE=1 SV=1
AT1G53310.2	CAPP1_ARATHPhosphoenolpyruvate carboxylase 1 OS=Arabidopsis thaliana GN=PPC1 PE=1 SV=1
AT4G20380.3	LSD1_ARATH LSD1 OS=Arabidopsis thaliana GN=LSD1 PE=1 SV=1
AT3G60590.4	PREDICTED: uncharacterized protein LOC103482552 [Cucumis melo]
AT1G04300.1	Y5436_ARATHMATH domain-containing At5g43560 OS=Arabidopsis thaliana GN=At5g43560 PE=1 SV=1
AT5G24300.2	SSY1_ARATHStarch synthase chloroplastic amyloplastic OS=Arabidopsis thaliana GN=SS1 PE=2 SV=1
AT5G61140.2	DEXHE_ARATHD -box ATP-dependent RNA helicase D 14 OS=Arabidopsis thaliana GN=At5g61140 PE=2 SV=1
AT1G31600.1	ALKB8_XENTRAlkylated DNA repair alkB homolog 8 OS=Xenopus tropicalis GN=alkbh8 PE=2 SV=2
AT4G01290.1	PREDICTED: uncharacterized protein LOC103408445
AT5G58140.2	PHOT2_ARATHPhototropin-2 OS=Arabidopsis thaliana GN=PHOT2 PE=1 SV=2
AT5G57410.1	ADIPA_XENLAAfadin- and alpha-actinin-binding A OS=Xenopus laevis GN=ssx2ip-a PE=1 SV=1
AT2G01450.3	MPK17_ARATHMitogen-activated kinase 17 OS=Arabidopsis thaliana GN=MPK17 PE=2 SV=1
AT5G08450.1	RXT3_SCHPOTranscriptional regulatory rxt3 OS=Schizosaccharomyces pombe (strain 972 ATCC 24843) GN=rxt3 PE=1 SV=1
**Cluster 59**	**Description**
AT1G32350.1	AOX3_ARATHUbiquinol oxidase mitochondrial OS=Arabidopsis thaliana GN=AOX3 PE=2 SV=2
AT5G42900.2	At4g33980 [Brassica napus]
AT3G25520.2	RL51_ARATH60S ribosomal L5-1 OS=Arabidopsis thaliana GN=ATL5 PE=2 SV=2
AT2G21660.1	RBG7_ARATHGlycine-rich RNA-binding 7 OS=Arabidopsis thaliana GN=RBG7 PE=1 SV=1
AT1G07050.1	CIA2_ARATH CHLOROPLAST IMPORT APPARATUS 2 OS=Arabidopsis thaliana GN=CIA2 PE=2 SV=1
**Cluster 60**	**Description**
AT3G22370.1	AOX1A_ARATHUbiquinol oxidase mitochondrial OS=Arabidopsis thaliana GN=AOX1A PE=1 SV=2
AT1G22470.1	At1g22470 F12K8_18
AT1G62045.1	ankyrin repeat [Medicago truncatula]
AT5G66400.1	DHR18_ARATHDehydrin Rab18 OS=Arabidopsis thaliana GN=RAB18 PE=2 SV=1
AT4G39670.1	ACDH_ARATHACD11 homolog OS=Arabidopsis thaliana GN=At4g39670 PE=2 SV=1
AT3G53160.1	U73C7_ARATHUDP-glycosyltransferase 73C7 OS=Arabidopsis thaliana GN=UGT73C7 PE=2 SV=1
AT1G23800.1	AL2B7_ARATHAldehyde dehydrogenase family 2 member mitochondrial OS=Arabidopsis thaliana GN=ALDH2B7 PE=2 SV=2
AT5G64310.1	AGP1_ARATHClassical arabinogalactan 1 OS=Arabidopsis thaliana GN=AGP1 PE=2 SV=2
AT4G20970.1	BH036_ARATHTranscription factor bHLH36 OS=Arabidopsis thaliana GN=BHLH36 PE=2 SV=1
AT5G04010.1	FB336_ARATHProbable F-box At5g04010 OS=Arabidopsis thaliana GN=NSFBx PE=2 SV=1
AT4G01360.1	BPS1_ARATH chloroplastic OS=Arabidopsis thaliana GN=BPS1 PE=2 SV=1
AT5G24470.1	APRR5_ARATHTwo-component response regulator-like APRR5 OS=Arabidopsis thaliana GN=APRR5 PE=1 SV=2
AT1G14520.1	MIOX1_ARATHInositol oxygenase 1 OS=Arabidopsis thaliana GN=MIOX1 PE=2 SV=1
AT2G29350.2	SAG13_ARATHSenescence-associated 13 OS=Arabidopsis thaliana GN=SAG13 PE=1 SV=1
AT1G01480.1	1A12_ARATH1-aminocyclopropane-1-carboxylate synthase 2 OS=Arabidopsis thaliana GN=ACS2 PE=1 SV=1
AT1G68795.1	CLE12_ARATHCLAVATA3 ESR (CLE)-related 12 OS=Arabidopsis thaliana GN=CLE12 PE=2 SV=1
AT2G19900.1	MAOP1_ARATHNADP-dependent malic enzyme 1 OS=Arabidopsis thaliana GN=NADP-ME1 PE=1 SV=1
AT2G21510.1	DNJ10_ARATHChaperone dnaJ 10 OS=Arabidopsis thaliana GN=ATJ10 PE=2 SV=2
AT5G59320.1	NLTP3_ARATHNon-specific lipid-transfer 3 OS=Arabidopsis thaliana GN=LTP3 PE=3 SV=1
AT2G37580.1	ATL33_ARATHRING-H2 finger ATL33 OS=Arabidopsis thaliana GN=ATL33 PE=2 SV=2
AT1G79450.1	ALIS5_ARATHALA-interacting subunit 5 OS=Arabidopsis thaliana GN=ALIS5 PE=1 SV=1
AT2G30550.2	PLA16_ARATHPhospholipase A1- chloroplastic OS=Arabidopsis thaliana GN=At2g30550 PE=1 SV=2
AT5G15240.1	AVT1_YEASTVacuolar amino acid transporter 1 OS=Saccharomyces cerevisiae (strain ATCC 204508 S288c) GN=AVT1 PE=1 SV=1
AT3G05880.1	RCI2A_ARATHHydrophobic RCI2A OS=Arabidopsis thaliana GN=RCI2A PE=2 SV=1
AT4G37370.1	C8D11_ARATHCytochrome P450 81D11 OS=Arabidopsis thaliana GN=CYP81D11 PE=2 SV=1
AT4G19390.1	structural constituent of ribosome [Medicago truncatula]
AT4G29670.2	TRL22_ARATHThioredoxin-like 2- chloroplastic OS=Arabidopsis thaliana GN=At4g29670 PE=2 SV=2
AT2G34850.1	ARAE2_ARATH UDP-arabinose 4-epimerase 2 OS=Arabidopsis thaliana GN=At2g34850 PE=2 SV=3
AT5G62520.1	SRO5_ARATHProbable inactive poly
AT4G37390.1	GH32_ARATHIndole-3-acetic acid-amido synthetase OS=Arabidopsis thaliana GN= PE=1 SV=3
AT1G17020.1	SRG1_ARATH SRG1 OS=Arabidopsis thaliana GN=SRG1 PE=2 SV=1
AT5G46010.1	WOX8_ARATHWUSCHEL-related homeobox 8 OS=Arabidopsis thaliana GN=WOX8 PE=2 SV=1
AT5G39520.1	DUF1997 family [Medicago truncatula]
AT4G02380.1	ARG2_VIGRRIndole-3-acetic acid-induced ARG2 OS=Vigna radiata radiata GN=ARG2 PE=2 SV=1
AT1G05340.1	cysteine-rich transmembrane domain A
AT2G23170.1	GH33_ARATHIndole-3-acetic acid-amido synthetase OS=Arabidopsis thaliana GN= PE=1 SV=1
AT5G66400.2	DHR18_ARATHDehydrin Rab18 OS=Arabidopsis thaliana GN=RAB18 PE=2 SV=1
AT5G22460.1	alpha beta fold hydrolase [Medicago truncatula]
AT1G72800.1	NUCL2_ARATHNucleolin 2 OS=Arabidopsis thaliana GN=NUCL2 PE=2 SV=1
AT2G38465.1	PREDICTED: uncharacterized protein LOC103410924
AT2G34500.1	C7101_ARATHCytochrome P450 710A1 OS=Arabidopsis thaliana GN=CYP710A1 PE=1 SV=1
AT3G46640.1	PCL1_ARATHTranscription factor LUX OS=Arabidopsis thaliana GN=LUX PE=1 SV=1
AT1G12845.1	AC012187_10Contains weak similarity to LIP1 gene product gi
AT4G18980.1	nucleolin-like [Malus domestica]
AT2G30140.2	U87A2_ARATHUDP-glycosyltransferase 87A2 OS=Arabidopsis thaliana GN=UGT87A2 PE=1 SV=1
**Cluster 61**	**Description**
AT3G25572.1	DCAM3_ARATHS-adenosylmethionine decarboxylase proenzyme 3 OS=Arabidopsis thaliana GN=SAMDC3 PE=2 SV=1
AT5G53550.2	YSL3_ARATHMetal-nicotianamine transporter YSL3 OS=Arabidopsis thaliana GN=YSL3 PE=2 SV=1
AT1G11870.2	SYSM_ARATHSerine--tRNA chloroplastic mitochondrial OS=Arabidopsis thaliana GN=OVA7 PE=2 SV=1

-2.936241562	-2.550344108	-2.658306987	0.601054505	0.53715573	0.578006968	0.144416101	0.712683997	-0.845726833
-2.80558078	-2.518509736	-3.126007733	0.5046139	1.113568583	0.520381216	-0.079185489	-0.524624466	-0.373690432
-2.452668789	-3.200391228	-2.892159708	0.564718689	-0.476671831	-0.049946612	-0.15606455	0.298366388	1.179715768
-2.801786198	-2.544111568	-2.453439676	1.092221813	1.371995317	0.345887657	-0.308664595	-0.512469691	-0.184735547
-3.127926388	-2.635807313	-2.63329821	1.541073763	0.945644853	0.963246412	0.264391035	-0.470179084	-0.118759693
-2.1024299	-2.604874008	-3.153674976	0.731472176	-0.505971761	1.091972392	0.7739397	0.341972136	-1.089503726
-2.68557417	-2.349290783	-3.297921124	0.488033015	0.488832248	0.967256465	0.378963828	0.44156382	-0.027705547
-2.786642751	-3.296230118	-3.120920945	0.725331231	0.650984497	0.932978799	-0.27846767	-0.095331931	-0.566003238
-2.562477576	-2.508770244	-3.369336656	0.454909902	0.01573659	0.551889449	0.739987712	0.628927153	0.031593181
-2.765292623	-2.63279392	-2.431067169	0.963104379	0.826495281	0.726934665	0.795880148	0.064195679	0.186955721
-3.504744013	-3.2260158	-3.284672255	0.646627763	1.508628913	1.482713293	-0.161620494	-0.443140604	0.412878688
-3.016128723	-2.990618109	-3.681311513	0.865535896	0.264976009	0.770572178	-0.618052031	-0.600099712	-0.33873324
-3.635603764	-2.273233226	-3.039737463	0.958027763	1.032368263	0.568009714	-0.056915376	0.360390924	-0.539248212
-2.553374293	-2.319454467	-3.217031924	0.515124554	1.183771627	0.519246052	-0.507581874	-0.092631729	0.398959274
-3.239369766	-3.215727263	-3.001194347	1.08803018	1.300719516	0.662028146	-0.151567507	0.175171722	-0.049545207
-3.207154807	-2.425462405	-2.409790953	0.886743356	1.242538616	0.183787966	0.263382058	-0.416799793	0.212249247
-2.660175695	-2.845597181	-3.186047695	1.180246015	0.916471537	0.768752575	-0.007091607	-0.935482885	-0.294364817
-2.882487609	-3.136371418	-3.037127228	1.464777724	1.173456029	0.891018427	0.130702303	-1.066032506	0.115066985
-2.816965934	-3.058514625	-3.171422951	1.560418227	1.129197319	1.053220413	0.387892857	0.281462135	0.174837408
-2.938102138	-3.197822156	-2.145281449	1.022224219	0.578669468	1.134346712	1.008254433	-1.056437519	0.303585532
-2.549850891	-3.162524782	-2.661628622	0.809248541	1.224988869	0.312501308	0.133171774	-0.828697004	0.074483998
-2.314881434	-3.019856356	-3.370259843	0.337137697	0.655951544	0.840732739	0.603378824	0.06668582	0.1613983
-3.261772812	-2.989762381	-2.859275459	0.786472362	-0.009712252	0.270404495	-0.628536329	0.323666219	0.670440477

WT-C	KO-C	OE-C	WT-2h	KO-2h	OE-2h	WT-12h	KO-12h	OE-12h
2.947090746	2.728399544	3.466562727	-1.116406	-1.235990075	-0.172989528	-2.569327228	-2.44602529	-1.601314896
4.340507861	2.270310445	5.172803702	-0.359082156	-2.377004064	-2.377004064	-2.377004064	-2.377004064	-1.916523594
4.284859083	2.834338187	3.203449103	-0.513875154	-2.70733152	-2.70733152	-1.971809342	-1.842600597	-0.57969824
3.684870777	2.018172145	3.913696376	-1.431934095	-0.304095082	-1.280193115	-2.976371471	-1.216479019	-2.407666516
3.740333137	2.68791812	3.115783317	-1.642549355	-1.689882787	-0.580698471	-2.12830883	-1.579106093	-1.923489038

WT-C	KO-C	OE-C	WT-2h	KO-2h	OE-2h	WT-12h	KO-12h	OE-12h
2.452659566	2.346412372	2.816812322	0.084232755	0.855737694	0.901665458	-2.98445522	-2.064827106	-2.408237841
3.025557394	2.978070762	3.100706585	0.035998391	0.896717126	-0.043962289	-2.640122943	-2.755641415	-2.597323611
3.40667644	2.73137539	3.469230176	-0.256984179	1.663614875	0.417046898	-2.62399634	-2.058269015	-2.748694245
3.233282723	2.142737082	2.596122364	0.625668994	1.487672756	0.185237264	-0.968165792	-3.726845961	-2.57570943
2.444425963	2.530166427	4.172038854	-0.271868727	1.679073705	0.788523383	-2.599079528	-4.013684708	-2.729595368
2.519726924	2.570106412	2.845584884	-0.319805735	0.845938856	0.649051958	-2.579830483	-2.050854677	-2.479918138
3.185630863	2.697521764	3.094849124	-0.41673913	0.383363842	0.012823184	-1.783706332	-1.925595181	-2.248148134
2.724388011	3.212607596	2.408729269	-0.230740795	1.345246253	0.414442568	-1.679901173	-2.530956061	-1.66381567
2.594411092	3.237948559	3.31269961	0.899835903	0.984246527	-0.195010848	-2.684016931	-2.752155405	-2.397958506
2.427732298	3.009791017	3.280669659	-0.298045983	1.635302795	0.012372445	-1.620443938	-1.861452037	-1.954167639
2.666106393	3.07449446	2.15239491	-0.068249849	1.282409697	0.720266077	-1.933109604	-2.378144756	-2.516167328
2.935245438	2.835763645	2.61708822	0.110219692	0.578781489	0.525576722	-2.251769475	-2.05547002	-2.29543571
2.779077777	2.729577547	3.086048505	0.600498609	0.796934632	0.274705672	-2.376794548	-1.944948761	-1.744102216
2.697451827	2.971893418	3.842775274	0.507781688	0.738442466	0.637253486	-2.940678813	-3.681184765	-2.861560096
3.090607746	2.623571433	3.148536099	-0.278701932	1.12612729	1.071822985	-2.895530098	-2.596871783	-2.289561739
2.735364428	3.170281271	3.037824347	0.187470352	1.338718237	0.757941311	-3.011237264	-1.927212336	-2.289150347
3.291173303	2.778168779	3.415680752	-0.362041465	1.367142266	-0.152090723	-1.591726165	-2.806278235	-2.239676611
2.84010194	2.762896805	2.9749389	-0.248465689	0.913534451	0.221178021	-1.745886407	-1.99496887	-2.723329152
2.93078199	2.760455184	3.972414548	-0.29809342	1.938321832	0.094660367	-2.504841747	-2.12713027	-2.766568483
2.787160782	3.094938793	2.603738531	0.249888148	1.589826534	1.022851781	-2.457985648	-2.550426984	-2.339991938
2.777162103	3.443119466	2.56746227	0.428525376	1.715070833	0.50203262	-2.181950827	-2.932128533	-1.462242556
2.468224224	2.926202141	2.555906826	0.069748556	1.294125634	0.232815453	-1.791285849	-2.407646652	-1.348090333
3.121921795	2.703801346	3.066703491	0.331642835	1.965268608	1.11411835	-2.618349198	-2.264954075	-2.420153151
2.970749119	3.103010013	2.619818986	0.287858651	1.991806486	0.671268458	-2.905949847	-1.721380411	-3.017181457
2.776490919	2.940533953	2.781519189	0.300965334	1.096882063	0.904618238	-3.533997063	-2.630244678	-2.636767953
2.485720386	2.889144707	3.385984793	-0.406761587	0.90281649	0.232963724	-1.580702977	-2.053390692	-1.855774845
2.743552849	2.999178066	3.087268561	0.388716323	1.19228877	0.65111265	-2.55608265	-2.699799118	-2.806235452
3.236215723	2.59985536	3.288469663	0.43047866	1.91807534	0.253215987	-3.140619997	-1.739664024	-2.846026712
3.189196835	2.633514993	3.091593261	-0.151807162	0.561903909	0.550224274	-1.848982386	-2.496112843	-2.529530882
2.928002171	3.052322512	2.939886084	-0.386660965	1.931974362	1.155736058	-2.231150083	-2.657501084	-2.332609054
2.59459172	2.592970148	3.461572379	-0.056005509	0.452830648	-0.012979335	-1.562778531	-2.683068931	-2.287132588
3.010896913	2.736492747	2.360094791	-0.052575611	0.176183193	0.47563542	-2.147763263	-2.147763263	-1.858518977
3.319803643	2.655355962	2.174372967	-0.191799103	2.114936176	1.035790531	-1.255937541	-3.364846339	-1.487676296
2.858049955	3.205313708	2.061092618	0.368227831	1.517358065	0.793790662	-1.88216965	-2.588169183	-1.333494006
2.902984123	2.690186402	3.391777339	-0.059145915	1.505551184	0.144139587	-1.894746779	-2.524636493	-2.556109448
3.121982558	3.079548673	2.509753399	-0.382061631	0.867863903	1.302113613	-1.971758596	-2.812292636	-1.715149283
3.317171512	2.779652354	3.591372934	-0.094510139	0.84989636	0.420942909	-0.844475803	-2.749845517	-2.27020461
3.36682077	2.502347179	3.804128971	-0.478278188	1.214586998	-0.196912437	-1.977972148	-1.970658089	-1.530421516
3.488681435	2.64445782	3.131262924	-0.421626073	0.086844614	0.413673809	-2.138644471	-1.983913966	-2.220736091
2.724156537	3.275318114	3.127153626	0.391828644	1.562671525	0.664367008	-3.183710021	-2.7441421	-2.817643333
3.100323301	2.450136186	2.611806372	0.079909443	1.050701789	0.906448005	-1.914094932	-2.762989596	-1.522240568
3.075462547	2.736663433	2.647328233	0.296004871	1.084002036	0.102799632	-1.361823814	-2.846735301	-1.733701636
2.905092166	2.907125138	4.194563564	0.25225691	0.956963446	0.007374848	-2.585426469	-2.191714629	-2.446234975
2.896714117	3.053830109	3.724415176	-0.051167224	1.875344864	0.110561704	-2.748889735	-2.115076159	-2.745732851
2.825412122	2.670081038	2.393788072	0.837728078	0.544057735	0.729925693	-1.110793111	-2.945099814	-2.945099814

WT-C	KO-C	OE-C	WT-2h	KO-2h	OE-2h	WT-12h	KO-12h	OE-12h
-3.879622699	-4.166037492	-3.034724257	1.201538737	1.743221414	1.463718106	-1.269973827	0.603001901	0.120863084
-3.741165532	-3.559077625	-2.309684129	0.358207996	0.517931684	1.297586996	-0.343622874	1.09167328	1.688150203
-3.599369047	-4.001242088	-2.599369047	0.876623721	0.282491311	1.193798543	0.663965889	1.412305486	0.770795233

AT4G39710.2	PNSL4_ARATHPhotosynthetic NDH subunit of lumenal location chloroplastic OS=Arabidopsis thaliana GN=PNSL4 PE=1 SV=1
AT5G13710.2	SMT1_ARATHCycloartenol-C-24-methyltransferase OS=Arabidopsis thaliana GN=SMT1 PE=1 SV=1
AT5G36700.2	PGP1B_ARATHPhosphoglycolate phosphatase chloroplastic OS=Arabidopsis thaliana GN=PGLP1B PE=1 SV=1
AT5G22770.1	AP2A1_ARATHAP-2 complex subunit alpha-1 OS=Arabidopsis thaliana GN=ALPHA-ADR PE=1 SV=1
AT4G25080.5	CHLM_ARATHMagnesium protoporphyrin IX chloroplastic OS=Arabidopsis thaliana GN=CHLM PE=1 SV=1
AT4G22490.1	ERLL1_ARAT 1-like lipid transfer 1 OS=Arabidopsis thaliana GN=AZI1 PE=1 SV=1
AT1G19715.3	JAL3_ARATHJacalin-related lectin 3 OS=Arabidopsis thaliana GN=JAL3 PE=2 SV=1
AT3G04630.3	WDL1_ARATH WVD2-like 1 OS=Arabidopsis thaliana GN=WDL1 PE=1 SV=1
AT3G14420.1	GLO1_ARATHPeroxisomal (S)-2-hydroxy-acid oxidase GLO1 OS=Arabidopsis thaliana GN=GLO1 PE=1 SV=1
AT3G17040.1	CF107_ARATH high chlorophyll fluorescent 107 OS=Arabidopsis thaliana GN=HCF107 PE=1 SV=1
AT3G54050.2	F16P1_ARATHFructose-1,6- chloroplastic OS=Arabidopsis thaliana GN=FBP PE=1 SV=2
AT1G19720.1	PPR52_ARATHPentatricopeptide repeat-containing At1g19720 OS=Arabidopsis thaliana GN=DYW7 PE=2 SV=1
**Cluster 62**	**Description**
AT1G26770.2	EXP10_ARATHExpansin-A10 OS=Arabidopsis thaliana GN=EXPA10 PE=2 SV=1
AT4G16146.1	cAMP-regulated phospho [Medicago truncatula]
AT4G35655.1	RIN4_ARATHRPM1-interacting 4 OS=Arabidopsis thaliana GN=RIN4 PE=1 SV=1
AT5G67480.2	BT4_ARATHBTB POZ and TAZ domain-containing 4 OS=Arabidopsis thaliana GN=BT4 PE=1 SV=1
**Cluster 63**	**Description**
AT3G17390.1	METK4_ARATHS-adenosylmethionine synthase 4 OS=Arabidopsis thaliana GN=METK4 PE=1 SV=1
AT3G15880.2	TPR4_ARATHTopless-related 4 OS=Arabidopsis thaliana GN=TPR4 PE=1 SV=2
AT4G08950.1	EXO_ARATH EXORDIUM OS=Arabidopsis thaliana GN=EXO PE=2 SV=1
AT1G10550.1	XTH33_ARATHProbable xyloglucan endotransglucosylase hydrolase 33 OS=Arabidopsis thaliana GN=XTH33 PE=2 SV=2
AT3G45970.1	EXLA1_ARATHExpansin-like A1 OS=Arabidopsis thaliana GN=EXLA1 PE=2 SV=1
AT2G23130.1	AGP17_ARATHLysine-rich arabinogalactan 17 OS=Arabidopsis thaliana GN=AGP17 PE=2 SV=1
AT1G15750.3	TPL_ARATH TOPLESS OS=Arabidopsis thaliana GN=TPL PE=1 SV=1
AT4G37450.1	AGP18_ARATHLysine-rich arabinogalactan 18 OS=Arabidopsis thaliana GN=AGP18 PE=2 SV=1
AT1G21910.1	ERF12_ARATHEthylene-responsive transcription factor ERF012 OS=Arabidopsis thaliana GN=ERF012 PE=2 SV=1
AT5G57560.1	XTH22_ARATHXyloglucan endotransglucosylase hydrolase 22 OS=Arabidopsis thaliana GN=XTH22 PE=1 SV=1
AT1G58110.1	BZP34_ARATHBasic leucine zipper 34 OS=Arabidopsis thaliana GN=BZIP34 PE=1 SV=1
AT5G57550.1	XTH25_ARATHProbable xyloglucan endotransglucosylase hydrolase 25 OS=Arabidopsis thaliana GN=XTH25 PE=2 SV=2
**Cluster 64**	**Description**
AT1G68840.1	RAV2_ARATHAP2 ERF and B3 domain-containing transcription repressor RAV2 OS=Arabidopsis thaliana GN=RAV2 PE=2 SV=1
AT3G13750.1	BGAL1_ARATHBeta-galactosidase 1 OS=Arabidopsis thaliana GN=BGAL1 PE=1 SV=1
AT5G56550.1	suppressor SRP40-like [Cucumis melo]
AT1G25275.3	thionin
AT2G17880.1	DNJ11_ARATHChaperone dnaJ chloroplastic OS=Arabidopsis thaliana GN=ATJ11 PE=1 SV=2
AT1G13700.1	6PGL1_ARATHProbable 6-phosphogluconolactonase 1 OS=Arabidopsis thaliana GN=At1g13700 PE=2 SV=1
AT3G58120.1	BZP61_ARATHBasic leucine zipper 61 OS=Arabidopsis thaliana GN=BZIP61 PE=1 SV=1
AT1G33760.1	ERF22_ARATHEthylene-responsive transcription factor ERF022 OS=Arabidopsis thaliana GN=ERF022 PE=2 SV=1
AT4G26288.1	SYRM_ARATHArginine--tRNA chloroplastic mitochondrial OS=Arabidopsis thaliana GN=EMB1027 PE=1 SV=1
AT2G29340.1	TRNHB_ARATHTropinone reductase homolog At2g29340 OS=Arabidopsis thaliana GN=At2g29340 PE=2 SV=1
AT1G23390.1	FBK9_ARATHF-box kelch-repeat At1g23390 OS=Arabidopsis thaliana GN=At1g23390 PE=2 SV=1
AT5G19120.1	7SB1_SOYBNBasic 7S globulin OS=Glycine max GN=BG PE=1 SV=2
AT4G37610.1	BT5_ARATHBTB POZ and TAZ domain-containing 5 OS=Arabidopsis thaliana GN=BT5 PE=1 SV=1
AT1G15125.1	MT810_ARATHProbable S-adenosylmethionine-dependent methyltransferase At5g38100 OS=Arabidopsis thaliana GN=At5g38100 PE=2 SV=1
AT4G17460.1	HAT1_ARATHHomeobox-leucine zipper HAT1 OS=Arabidopsis thaliana GN=HAT1 PE=2 SV=1
AT2G30600.3	Y2060_ARATHBTB POZ domain-containing At2g30600 OS=Arabidopsis thaliana GN=At2g30600 At2g30610 PE=2 SV=1
AT1G68520.1	COL6_ARATHZinc finger CONSTANS-LIKE 6 OS=Arabidopsis thaliana GN=COL6 PE=2 SV=2
AT2G25200.1	DUF868 family [Medicago truncatula]
AT3G15630.1	PREDICTED: uncharacterized protein LOC103502747
AT4G37520.1	PER50_ARATHPeroxidase 50 OS=Arabidopsis thaliana GN=PER50 PE=1 SV=1
AT4G39070.1	BBX20_ARATHB-box zinc finger 20 OS=Arabidopsis thaliana GN=BBX20 PE=1 SV=1
AT1G33055.1	hypothetical protein AT1G33055
AT2G41100.3	CML12_ARATHCalmodulin 12 OS=Arabidopsis thaliana GN=CML12 PE=1 SV=3
AT1G03870.1	FLA9_ARATHFasciclin-like arabinogalactan 9 OS=Arabidopsis thaliana GN=FLA9 PE=1 SV=1
AT5G20250.3	RFS6_ARATHProbable galactinol--sucrose galactosyltransferase 6 OS=Arabidopsis thaliana GN=RFS6 PE=2 SV=2
**Cluster 65**	**Description**
AT2G23110.2	PREDICTED: uncharacterized protein LOC103407164
AT2G03850.1	CR15B_ARATH COLD-REGULATED chloroplastic OS=Arabidopsis thaliana GN=COR15B PE=1 SV=1
AT3G59350.2	PTI13_ARATHPTI1-like tyrosine- kinase 3 OS=Arabidopsis thaliana GN=PTI13 PE=1 SV=1
AT3G56891.1	HIP26_ARATHHeavy metal-associated isoprenylated plant 26 OS=Arabidopsis thaliana GN=HIPP26 PE=1 SV=1
AT5G03970.2	FB251_ARATHF-box At5g03970 OS=Arabidopsis thaliana GN=At5g03970 PE=2 SV=1
AT1G11925.1	STGL3_ARATHStigma-specific STIG1 3 OS=Arabidopsis thaliana GN=At1g50720 PE=2 SV=1
AT2G42500.2	PP2A4_ARATHSerine threonine- phosphatase PP2A-4 catalytic subunit OS=Arabidopsis thaliana GN=PP2A4 PE=2 SV=2
AT2G32190.1	PCC1_ARATHCysteine-rich and transmembrane domain-containing PCC1 OS=Arabidopsis thaliana GN=PCC1 PE=1 SV=1
AT5G65390.1	AGP7_ARATHClassical arabinogalactan 7 OS=Arabidopsis thaliana GN=AGP7 PE=1 SV=2

Table S2. (a) Primer sequences (LP, RP and BP) used for detecting homozygous lines of knockout T-DNA insertion (SALK_150614) mutant. (b) Loci and primer sequences along with the expected amplicon sizes (bp) and annealing for ERF109 of cluster 1 and seven selected genes of cluster 2 used in conducting semi-quantitative RT-PCR for validating the RNA-Seq datasets of Arabidopsis leaves of three genotypes (e.g., WT, KOERF109 and OEERF109) treated with high salt stress at two time points (2 and 12 h) as well as the untreated control. Actin was used as the house-keeping gene. WT = wild type, KO = knockout, OE = overexpression.

Locus	Description
AT4G34410.1	SALK_150614

Locus	Description
AT4G34410.1	ERF109_ARATHEthylene-responsive transcription factor ERF109 OS=Arabidopsis thaliana GN=ERF109 PE=1 SV=1
AT1G62370.1	DIAP1_DROMEDeath-associated inhibitor of apoptosis 1 OS=Drosophila melanogaster GN=DIAP1 (DEL1/2) PE=1 SV=2
AT3G15540.1	IAA19_ARATHAuxin-responsive IAA19 OS=Arabidopsis thaliana GN=IAA19 PE=1 SV=2
AT2G23170.1	GH3.3_ARATHIndole-3-acetic acid-amido synthetase OS=Arabidopsis thaliana GN=GH3.3 PE=1 SV=1
AT1G19220.1	ARFB_ARATHAuxin response factor 19 OS=Arabidopsis thaliana GN=ARF19 PE=1 SV=2
AT5G01240.1	LAX1_ARATHAuxin transporter 1 OS=Arabidopsis thaliana GN=LAX1 PE=2 SV=1
AT5G18010.1	SAU19_ARATHAuxin-responsive SAUR19 OS=Arabidopsis thaliana GN=SAUR19 PE=2 SV=1
AT2G01420.2	PIN4_ARATHAuxin efflux carrier component 4 OS=Arabidopsis thaliana GN=PIN4 PE=1 SV=1
AT3G18780	House-keeping gene

Table S3. Regulated cell death genes (e.g., GSTs) as well as genes in the tryptophan biosynthesis (e.g., IPTS) and plant hormone signal transduction (e.g., LAX2) pathways that have putative binding sites for ERF109 (locus AT4G34410) and other TFs of the AP2/ERF gene family.

Locus	Gene
AT2G04400	IGPS
AT2G21050	LAX2
AT2G21050	LAX2
AT2G21050	LAX2
AT2G21050	LAX2
AT2G29420	GSTU7
AT2G29420	GSTU7
AT2G29420	GSTU7
AT2G29440	GSTU6

-4.068529526	-3.41810351	-3.222983119	1.06330664	0.748330505	1.829867468	1.160513826	0.169883798	1.737713921
-3.881460769	-3.386655317	-4.072707559	0.373879526	1.212121167	1.048370026	2.307415792	1.537593865	1.861443269
-3.774030934	-4.019998615	-2.774030934	1.036000794	1.331982304	1.487424661	0.315297718	0.626097081	1.771257924
-3.563412496	-4.137249159	-2.563412496	1.247647452	0.066293826	0.571465559	1.124096165	1.08654779	0.893525041
-3.648593372	-3.871133824	-3.055954117	0.381900541	1.124013461	1.449156034	1.150159056	-0.455681877	1.926134098
-3.758247801	-3.583110343	-3.034764007	1.113420282	1.794592804	0.631103586	0.443136688	0.599885885	0.793982905
-4.256377933	-4.023496361	-2.731462816	1.210113046	0.628198907	0.239022757	1.169548858	1.488092953	1.276360588
-3.55585767	-3.403130929	-3.35825771	1.491391338	1.925960273	1.443314859	0.260803094	0.747504165	-0.551727421
-4.276105732	-3.986420007	-3.350011633	0.380981482	1.014525782	1.817998523	2.25536417	0.815438087	1.328229328
-3.514425173	-4.134548217	-3.218258444	0.954400044	0.289772416	0.803898886	1.108936747	1.333425079	1.376798662
-3.024474288	-3.506167518	-3.788310747	0.534590838	1.169209238	1.568924534	2.181691576	1.566211938	2.298324429
-3.934236653	-3.369667962	-3.442093394	0.975036593	0.285964147	0.655072171	1.112684394	1.457404698	1.259836006

WT-C	KO-C	OE-C	WT-2h	KO-2h	OE-2h	WT-12h	KO-12h	OE-12h
1.243300892	0.872521559	1.873736073	0.870678978	0.913222658	-0.105846449	-2.233427986	-3.290924045	-3.14326168
1.82013824	1.146189243	2.51160498	0.278180627	0.972253201	0.262568665	-3.33837636	-2.755679838	-2.896878758
1.202007057	1.576265218	1.197191878	1.544878923	1.818158793	1.126206901	-3.77582236	-3.038933123	-3.475405732
1.980346778	1.462518817	1.622426237	0.216739819	0.706072944	0.075940842	-1.978255879	-2.973251173	-3.112538385

WT-C	KO-C	OE-C	WT-2h	KO-2h	OE-2h	WT-12h	KO-12h	OE-12h
1.619762709	1.039912372	1.809889709	-1.621626902	-2.079260263	-2.179605112	0.308792525	-0.338160173	0.440295134
1.350835945	1.073154869	1.327019054	-1.719454748	-1.870803904	-3.050432096	0.785992379	-0.236334637	0.340023139
1.865659806	1.81388686	1.468950748	-3.268121203	-2.64252315	-2.751248983	0.447559118	1.005091887	1.060744917
1.603764865	1.526003762	1.667853634	-2.44925956	-2.165930391	-2.330235456	0.877996656	0.09473116	0.17507533
2.014228104	1.842380964	1.756954068	-2.734604673	-1.893858455	-2.692189304	0.920654856	-0.63563443	0.42206887
1.770494719	0.994778469	2.462492648	-2.210835655	-2.596907109	-1.661057867	0.564506984	0.418027794	0.258500016
0.956430129	1.334444571	2.06041469	-2.610692463	-2.441260047	-1.607504577	1.140401075	-0.163416708	1.33118333
1.945931236	1.318203375	1.730860871	-2.236882031	-1.937450479	-1.972622198	0.792869316	-0.58120332	0.940293229
2.103172863	2.068304779	1.52949482	-3.177654519	-3.133144473	-1.723473586	0.946876303	0.5969544	1.389469411
1.123417133	1.475778963	2.184942629	-2.191239183	-2.032513568	-1.597903043	0.767722413	-0.443967831	0.713762487
1.930479849	2.056102194	2.287449476	-2.04890533	-2.04890533	-2.04890533	1.179451624	0.210819105	0.482413742
1.424451732	1.416482923	1.342191547	-2.238415175	-2.092014788	-1.362252554	1.071283445	-0.494754928	0.933027799

WT-C	KO-C	OE-C	WT-2h	KO-2h	OE-2h	WT-12h	KO-12h	OE-12h
-0.311068401	-1.363130579	0.104516148	-1.696240807	-2.527394111	-1.997614525	2.564368085	2.52091726	2.705646931
-0.546723078	-0.250944773	-0.818036874	-1.894401197	-2.029959615	-0.291076666	2.633137269	1.596047401	1.601957533
-0.153899705	-0.01570511	0.390401857	-2.625502362	-2.674734087	-1.857289473	2.50424963	2.410789317	2.021689933
0.363409406	-1.316078063	-1.412380201	-1.019037773	-1.940474513	-0.992841309	2.649218695	1.424357908	2.24382585
-1.070506281	-1.617804709	-0.952002165	-1.278947017	-1.589306102	-1.201418686	2.673611945	2.372871055	2.66350196
-0.554335192	-1.012512661	-1.24362757	-1.359724635	-1.256369592	-1.873688376	3.061094209	1.844967039	2.394196778
0.359759499	-0.024471953	-0.341482463	-1.781485348	-2.571154765	-1.615357767	1.957217011	2.473871546	1.54310424
-0.052044755	-0.004421623	-0.239245377	-1.800206852	-1.759785278	-1.693197197	2.003649021	0.661026859	2.884225203
0.251945539	-0.905477229	-0.437137942	-1.771038679	-1.970378409	-1.691060142	2.437014998	1.765784133	2.320347731
-0.628689248	-1.360395432	-0.716848881	-1.633764519	-1.364776961	-0.916125775	2.558381585	1.793927837	2.268291376
-0.391946607	-1.368988429	-1.396355929	-1.935021202	-1.462979534	-1.100409455	2.706009667	2.380213312	2.569478177
0.736114644	-0.366506361	-0.683741771	-1.764723267	-2.544894262	-1.637248678	2.455985914	1.681377787	2.123635994
-0.079152909	-0.603309027	-2.306132946	-1.992875839	-2.387996961	-0.223614283	3.317541638	1.505682858	2.769857469
0.11001233	-0.289428886	-0.905861885	-1.138772572	-1.751281399	-1.520717852	1.76120444	1.234818786	2.500027039
0.245161404	-0.60015768	-0.881196833	-1.650278928	-1.80462183	-1.422277722	1.429684332	3.137751951	1.545935306
-0.091029643	-0.384577641	-0.53351606	-1.762539481	-2.071639499	-0.69391778	2.83781687	0.86048367	1.838919565
0.552187127	0.199555187	-1.698427778	-2.395375655	-2.755896914	-1.088464498	2.134779905	2.890979699	2.160662929
0.469559043	0.046940067	0.40241347	-1.976847799	-2.165686678	-1.885888391	2.205196826	0.787584979	2.116728485
0.092165778	-1.249319241	-1.09419338	-1.434518135	-2.18142054	-1.441964319	2.902437413	1.351536092	3.055276332
-0.200283909	0.128072627	-1.86880852	-1.655007599	-1.48813123	-1.641830795	2.618469961	1.236036272	2.871483193
0.376234334	-0.417770072	0.099872396	-1.572343754	-2.239288617	-1.581156054	2.336052132	0.562647458	2.435752178
0.433974023	-1.155485552	-1.567753962	-0.665388965	-1.964920785	-1.230690158	2.749914459	1.022501149	2.377849791
-0.148711887	-0.278441984	-0.419943446	-0.719874149	-2.720307934	-1.205272062	2.568901203	0.668313593	2.255336666
0.773806141	-0.163844191	0.052727713	-2.581868577	-3.13436791	-1.747344787	2.538686719	1.603537469	2.658667423
-0.176107316	-0.227221188	-1.887602223	-1.431321742	-1.887602223	-1.079423139	2.349043458	1.97445236	2.365782013

WT-C	KO-C	OE-C	WT-2h	KO-2h	OE-2h	WT-12h	KO-12h	OE-12h
1.709900023	2.110196777	2.522928154	-1.509993438	-1.434669404	-0.783232625	-0.36071527	-1.359262001	-1.616582756
1.961138355	2.025798673	1.99413168	-1.361405618	-0.750799957	-0.591312361	-0.666948846	-1.290488378	-1.320113547
1.149942052	1.478991307	1.46321413	-1.132699069	-0.677582204	-0.19124709	-0.786341541	-0.929519136	-1.082465836
1.4887206	2.537324851	2.144836096	-1.61916792	-1.554220361	-1.005660844	-0.552566453	-1.312106607	-1.232292267
1.601046772	1.114407087	2.548467094	-1.135341628	-1.115283976	-1.135341628	-0.392729536	-1.135341628	-1.135341628
1.995238832	1.923555026	1.445426786	-1.232194206	-1.082934841	-0.415799297	-0.417438723	-1.232194206	-0.98365937
2.396621901	0.940313264	2.247992913	-0.496184785	-0.89648442	-0.938924078	-0.996141144	-0.871363264	-1.65910522
1.252406628	1.764451811	2.094189054	-0.829804514	-0.739990273	-0.36887901	-0.665244798	-1.021354664	-1.485774235
2.410307336	1.520294284	2.040941184	-1.931279724	-0.820131881	-1.56570452	-0.968687202	-0.827126862	-0.797612945

Gene	roduct_length (b	F primer	R primer	nnealing temp (°C)
ERF109 (WT)	1003	LP: CGCGATGC	RP: GATCTCAG	60
ERF109 (KO)	~710	BP: GCGTGGAC	RP: GATCTCAG	

Gene	roduct_length (b	F primer	R primer	nnealing temp (°C)
ERF109	520	GGTGCTTTACA	TTTCCCCAAGG	51
DIAP1	315	TGGACTACGTT	CGAATCACAAC	52.7
IAA19	312	AGCTTTCACGG	CGGAAACCGA/	52.4
GH3.3	314	TGCTTTGTGGT	CATGGCTCCAG	52.4
ARF19	289	TACTCTGTTAT(	CATGGCTCGCT	55.2
LAX1	308	TATTTGGGGCT	TGTGAAGACGT	55
SAUR19	311	ATCAACCAACA	ACCCATCGGAT	49.2
PIN4	301	AGATAGGCCAA	AAAAGATGAGA	52.5
actin	353	CCAGTGTTGTT	GGTTGTACGAC	50

Matrix ID	Position (-)	strand (+/-)	Hit Sequence	TF loci
TFmatrixID_0060	406	-	tCGCCGtc	AT1G03800;AT1G04370;AT1G43160;AT1G53910;AT2G40220;AT2G46310;AT2G47520;AT3G16770;AT3G61630;AT4G11140;AT4G13620;AT4G34410;AT5G07310;AT5G44210;AT5G50080;AT5G61890;AT5G64750
TFmatrixID_0060	221	+	gcCGGCGa	AT1G03800;AT1G04370;AT1G43160;AT1G53910;AT2G40220;AT2G46310;AT2G47520;AT3G16770;AT3G61630;AT4G11140;AT4G13620;AT4G34410;AT5G07310;AT5G44210;AT5G50080;AT5G61890;AT5G64750
TFmatrixID_0081	219	+	taGCCGGcga	AT4G17490;AT4G18450;AT4G34410;AT5G50080;AT5G61590;AT5G61600
TFmatrixID_0081	219	-	tagCCGGCga	AT4G17490;AT4G18450;AT4G34410;AT5G50080;AT5G61590;AT5G61600
TFmatrixID_0607	91	+	tcGCCGCtaa	AT4G34410
TFmatrixID_0060	229	-	aCGCCGgc	AT1G03800;AT1G04370;AT1G43160;AT1G53910;AT2G40220;AT2G46310;AT2G47520;AT3G16770;AT3G61630;AT4G11140;AT4G13620;AT4G34410;AT5G07310;AT5G44210;AT5G50080;AT5G61890;AT5G64750
TFmatrixID_0081	229	+	acGCCGGcct	AT4G17490;AT4G18450;AT4G34410;AT5G50080;AT5G61590;AT5G61600
TFmatrixID_0081	229	-	acgCCGGCct	AT4G17490;AT4G18450;AT4G34410;AT5G50080;AT5G61590;AT5G61600
TFmatrixID_0081	409	+	tgGCCGGcaa	AT4G17490;AT4G18450;AT4G34410;AT5G50080;AT5G61590;AT5G61600

AT2G47730	GSTF8
AT2G47730	GSTF8
AT2G47730	GSTF8
AT2G47730	GSTF8

TFmatrixID_0060	146	-	tCGCCGgc	AT1G03800;AT1G04370;AT1G43160;AT1G53910;AT2G40220;AT2G46310;AT2G47520;AT3G16770;AT3G61630;AT4G11140;AT4G13620;AT4G34410;AT5G07310;AT5G44210;AT5G50080;AT5G61890;AT5G64750
TFmatrixID_0060	148	+	gcCGGCGt	AT1G03800;AT1G04370;AT1G43160;AT1G53910;AT2G40220;AT2G46310;AT2G47520;AT3G16770;AT3G61630;AT4G11140;AT4G13620;AT4G34410;AT5G07310;AT5G44210;AT5G50080;AT5G61890;AT5G64750
TFmatrixID_0081	146	+	tcGCCGGcgt	AT4G17490;AT4G18450;AT4G34410;AT5G50080;AT5G61590;AT5G61600
TFmatrixID_0081	146	-	tcgCCGGCgt	AT4G17490;AT4G18450;AT4G34410;AT5G50080;AT5G61590;AT5G61600
